# Modulation of Epigenetic Control by Advanced Molecular Tweezers

**DOI:** 10.1002/chem.71588

**Published:** 2026-08-19

**Authors:** My‐Hue‐Le, Bianca Kamba, Anja Matena, Christine Beuck, Victoria Momand, Spiros Salvanos, Jessica Vossen, Mike Blueggel, Thomas Schrader, Peter Bayer

**Affiliations:** ^1^ Institute of Organic Chemistry I Faculty of Chemistry University of Duisburg‐Essen Essen Germany; ^2^ Structural and Medicinal Biochemistry Faculty of Biology University of Duisburg‐Essen Essen Germany; ^3^ Functional Biochemistry Faculty of Biology Institute of Molecular Biology II University of Duisburg‐Essen Essen Germany

**Keywords:** epigenetics, histone protein, molecular tweezers, posttranslational modifications, supramolecular chemistry

## Abstract

This work addresses the question if supramolecular systems can be tailored to interfere with posttranslational modifications (PTM) and epigenetics. As a prominent example, the efficient epigenetic control operative in nucleosome‐DNA interactions was selected. Conceptually, transcription activation by specific acetylation of strategic lysine residues on the Histone H4 protein is replaced by noncovalent inclusion of the respective lysine residues inside the cavities of designed molecular tweezers. To this end, novel dimeric molecular tweezers with functional linkers were designed, which exhibit nanomolar affinity to the DNA‐binding fragment of histone H4. Fluorescence titrations and ITC measurements provide experimental evidence for high selectivity over other lysine‐containing H4 areas. Notably, rigid short linkers with hydrogen bond donors outperform flexible peptidic connections. MD simulations identified new binding motifs involving attractive interactions between H4 residues and linker functionalities. The ability of modified tweezers to interrupt the essential histone‐DNA interaction was demonstrated in a fluorescence anisotropy assay. In addition, the most powerful tweezers with high H4 affinity were subjected to a competitive enzymatic HAT experiment, where they successfully competed for lysine binding sites and impaired HAT function. Thus, powerful new histone binders were developed which act as supramolecular PTM on a histone H4 peptide.

## Graphical abstract

Tailored dimeric molecular tweezers recognize the DNA‐binding part of Histone H4 and interrupt the essential histone‐DNA interaction.

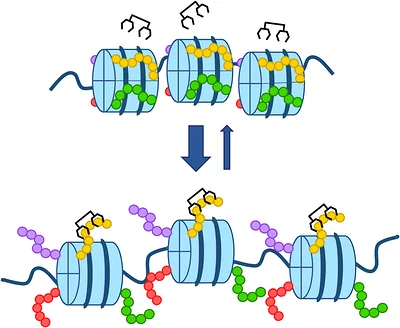

## 1 Introduction

Since Lehn, Pedersen, and Cram were awarded the Nobel Prize over 30 years ago [1], Supramolecular Chemistry has greatly matured: today, most noncovalent interactions are reasonably well understood, and artificial receptor molecules can be synthesized for almost any given target. However, applications in the field of biology are just beginning to emerge, even though our understanding of (patho)biochemical processes is increasingly reaching the molecular level. To address, e.g., protein‐protein interactions, the supramolecular ligands need to bind in shallow grooves or large pores on the protein surface. This is often more challenging than the development of traditional enzyme inhibitors, which bind in deep pockets at active sites of proteins [2].

Clearly, there is an urgent need for a deeper understanding of the underlying principles of protein recognition, manipulation, and functional modulation by external agents. If supramolecular chemists and biologists learn how to address a specific epitope on a protein target, tailored ligands may be used to modulate protein function in a predictable way. Various powerful innovative concepts have been pursued in recent years; thus, secondary structure elements have been imitated with α‐helix and β‐sheet mimetics and successfully applied to interfere with problematic protein‐protein interactions [3, 4]. Macrocyclic highly preorganized aromatic ligands have been used to block ion channels (calixarenes) [5], induce protein dimerization (cucurbiturils) [6] or counteract pathologic protein aggregation (tweezers) [7]. Molecular imprinting has led to polymer materials with extremely high selectivity for a given protein target [8], and combinatorial dendrimer structures have been developed into novel enzyme inhibitors as well as protein pore binders (GPC peptides) [9, 10].

This research project goes one step further and asks if supramolecular systems can be tailored to interfere with two other related fundamental biological mechanisms/processes: posttranslational modifications and epigenetics. Both events lead to different protein species as obtained from classical gene expression, and in both, cationic residues play a key role. A biological example of prime importance is the posttranslational acetylation of histone proteins in nucleosomes [11].

Nucleosomes are the basic entities of chromosome architecture. They consist of a stretch of DNA wrapped into a superhelix around a histone octamer [12, 13, 14]. In eukaryotes, this octamer is composed of four different histone proteins (two H2A, H2B, H3, and H4) whose N‐ and C‐termini (histone tails) comprise short sequences of intrinsically disordered regions (IDR) which undergo a highly dynamic exchange of conformational states [15]. These extremities protrude from the nucleosome core but can adopt several conformations to interact with chromosomal and linker DNA.

Histone tails participate in a variety of chromatin functions, among which are DNA accessibility, transcription or nucleosome stability and dynamics [16, 17]. These functions are coordinated in time and space by epigenetic regulation of histone tails and their interaction with a multitude of cofactors and enzymes. To cope with the particular function, histone tails are enriched with basic lysine and arginine residues (among others) and are targeted by special enzymes that either donate a chemical group to these amino acids or remove it. The plethora of these post‐translational modifications (PTMs), including acetylation, methylation, phosphorylation, and ubiquitylation (among others)—termed the histone code—enable the tight control of both heterochromatin and euchromatin structure and function [18]. An effective cross‐talk between different modifications certainly obtains a fine‐tuning of the overall control. The covalent but reversible PTMs provide an efficient potential for the cell to react to external stimuli. Thus, histone modifications are a versatile cellular toolkit to control chromatin architecture and regulate gene transcription. External stimuli either can lead to a short‐term response or be retained over cellular divisions even in germline cells, thereby providing the basis for epigenetic heredity. Epigenetic changes play a significant role in regular cell functioning and disease progression [19].

Among those PTMs, acetylation, which occurs only in lysine residues, has cumulative effects on the tail structures. It eliminates the positive charge of the side chain, thereby reducing conformational heterogeneity while inducing helical propensity [20]. Along with these structural changes, acetylation enables and promotes active transcription by reducing histone binding to DNA when neutralizing the positive charges on lysines and by limiting intermolecular hydrogen bonding [17]. The liberated DNA stretches are subsequently exposed to the cellular enzymatic machinery. Acetylating and deacetylating enzymes in the cell regulate these important post‐translational processes [21]. Histone acetylation is usually considered a mark that triggers transcription activation, whereas histone deacetylation inhibits gene expression. Prominent examples of modified lysines in histone tails for H3 are K4, K9, K14, K18, and K27, for H4 are K5, K8, K12, and K16, and for H2A/H2B are K6, K7, K16, and K17. When considering epigenetics, gene silencing in mammalian cells is usually associated with hypoacetylated and hypermethylated histones in conjunction with methylated DNA stretches (CpG islands) [17].

It appears that the interaction between densely packed histone units and the surrounding supercoiled DNA very much hinges on the lysine phosphate contacts and is therefore largely electrostatic, making it fully reversible and only moderately strong [22]. This feature opens up the opportunity for deliberate interference with external agents directed towards the histone lysine residues, or better, their arrangement in small characteristic peptide motifs. Antibodies have been produced for many lysine positions on histones, and are available on the market as biochemical tools, but even their selectivity is sometimes poor [23]. It would be highly desirable to develop small molecules that could be targeted to key areas on histones, but such a toolbox has remained elusive until today.

Although several artificial binding motifs have been invented for the recognition of lysines and arginines, applications with sequence selectivity on peptides and protein surfaces are very rare [24, 25, 26]. Crescent‐shaped annulated heterocycles [27], calixarene sulfonates [28], cucurbiturils [29] and others have been used for amino acid, peptide and protein recognition, albeit combinations with selectivity for a given peptide fragment are very hard to make.

Recognition of histone tails with simple sulfonato‐calixarenes has been accomplished by Hof et al. [30]. The authors combined three such supramolecular sensors with appropriate dye molecules and produced an elegant chemical sensor array that was able to distinguish between short histone peptide fragments with single histone code elements. Later, Kanai et al. covalently connected similar sulfonato‐calixarenes with peptide‐like linkers to a dimeric and trimeric ensemble in order to increase affinity and selectivity [31]. Submicromolar Ka values were achieved with the trimeric ligand, especially when the histone carried a trimethylated lysine; no selectivity was observed between H4 and H3. The dimeric calixarene was used in pull‐down experiments with nuclear extracts and pulled down endogenous histones, enriched in H3K9M3. This is an important first step towards deliberate external interference with posttranslational modification of histones. In our concept, we employed established highly selective lysine binders and connected them with flexible peptidic as well as rigid linkers to dimers, which showed nanomolar affinities to H4 histone fragments and successfully competed with histone‐DNA complexation as well as enzymatic histone methylation.

### 1.1 Molecular Tweezers

Several decades ago, the Klärner group developed a rigid skeleton composed of alternating benzene and norbornadiene rings forming a torus‐shaped cavity, whose inward‐directed π‐systems render it exceptionally electron‐rich, ready to accept cationic guests inside [32]. For water solubility, we added two flanking phosphate groups to its central hydroquinone element and obtained a water‐soluble molecular tweezer with exquisite selectivity for the two basic proteinogenic amino acids lysine and arginine [33]. In aqueous buffer, lysine and arginine are bound with moderate affinity (K_D_ ∼10 µM vs. 30 µM), in a unique combination of electrostatic attraction, dispersive interactions, and the hydrophobic effect. Binding is highly dynamic and fully reversible, and micromolar concentrations do not compromise any protein function [34]. The parent tweezer was shown to dock onto every basic amino acid residue in a short peptide, as long as it is sterically accessible [35]. Transient binding to the Alzheimer's peptide Amyloid β leads to full disaggregation of preformed toxic Aβ oligomers and fibrils and is currently being developed as a disease‐modifying therapy against AD, PD, and other neurodegenerative diseases [36, 37, 38].

Selective interference with histone‐DNA interactions would allow to modify the related processes of fundamental importance and may have medicinal implications. Molecular tweezers with their specificity for lysines and arginines are, in principle, well‐suited to dock onto histone tails, but lack selectivity and affinity for a certain peptide stretch. Since histone proteins are composed of many similar residues, mainly basic and nonpolar amino acids, it becomes highly challenging to tailor a tweezer derivative for a desired peptide stretch. Moreover, the dynamic nature of the histone tails makes selective complexation even more demanding. On the other hand, the introduction of peptide signals or the multiplication of single tweezer units has, in the recent past, allowed us to address hot spots on various proteins and led to powerful inhibition of essential protein‐protein interactions (PPI) [39, 40]. Thus, we decided to use these approaches to develop an advanced tweezer derivative for the selective recognition of the DNA‐binding site on histone H4. With these new chemical tools, we set out to study whether entrapment of lysine sidechains in histone peptides and the concomitant charge neutralization caused by these supramolecular hosts can weaken the histone‐DNA interaction as observed under epigenetic control (Figure 1). Moreover, we intended to modify our tweezers in such a way that they would selectively address a preferred region at the H4 N‐terminus without impairing other acetylation sites therein. We tested this hypothesis in a model with histone peptides derived from the core histone H4 and synthetically designed nucleosomal DNA.

**FIGURE 1:**
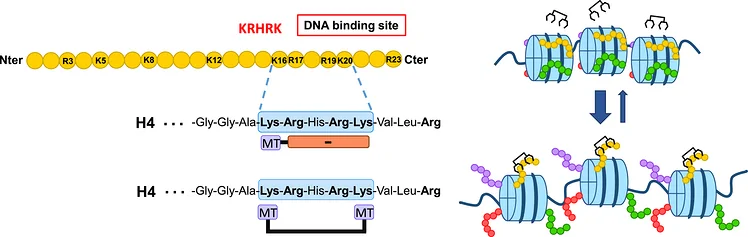
Mimicking epigenetic modulation by supramolecular lysine caps. The natural process of covalent acetylation of critical areas within the histone protein H4 is imitated by noncovalent complexation by advanced molecular tweezers (MT) (left). These are optimized with respect to high affinity for competing with the DNA‐binding site on H4. Several rounds of optimization furnish specific interfering tweezers that are able to imitate epigenetic modulation (right).

With this in vitro system, we set out to investigate the potential suitability of tweezer molecules as reversible epigenetic modulators. More specifically, we measured the dose‐dependent inhibitory influence of optimized tweezers on both the stability of histone‐DNA complexes and on histone‐acetyltransferase (HAT) activity. The project led to the development of specific binding motifs with nanomolar affinity for the DNA binding site on the histone peptide, which strongly perturb the DNA‐histone interaction and competitively inhibit HAT.

To give a proof‐of‐principle for our hypothesis, we had to reduce the tremendously complex system of the histone tail hub to a simplified but physiologically genuine one. The latter should allow demonstrating that lysine‐specific tweezers are able to interfere with the noncovalent interaction between histones and chromosomal DNA by lysine capping. The most easily accessible and technically feasible model of histone‐DNA interaction serving our purpose was described by Hong and coworkers [41], who determined the affinity of the H4 amino‐terminal sequence for a double‐stranded DNA from erythrocyte nucleosome core particles in the absence and presence of acetyl groups on lysine residues.

Given this groundwork, we used peptides derived from the N‐terminal tail of the core histone H4 (H4_1‐22_) and synthetically designed nucleosomal DNA as an in vitro system to investigate the potential suitability of our tweezer molecules as reversible epigenetic modulators.

## 2 Formation of Histone Peptide:DNA complexes

Hong and coworkers reported DNA affinities towards non‐acetylated and acetylated peptides within the range of mM down to nM K_D_ [38]. In a first attempt, we monitored the binding of our H4 peptides to the selected double‐stranded DNA by fluorescence anisotropy and determined K_D_ values in the low micro‐molar regime (∼ 2 µM).

Concurrently, multidimensional NMR spectroscopy was applied to assign the H4 H_N_ resonances in the HSQC spectrum (red peaks in Figure 2A) of the histone tail peptide [42]. Addition of DNA led to a perturbation of a number of those resonances, resulting in the appearance of a second conformation. The corresponding peak intensities decline (with respect to the original peaks) (Figure 2B). Given our fluorescence anisotropy data, the drop in intensities of ^15^N‐resonances assigned to the C‐terminal sequence including the predicted KRHRK binding region is explained by the formation of a higher molecular weight H4‐DNA complex and the resulting line broadening of the H4 peaks.

**FIGURE 2:**
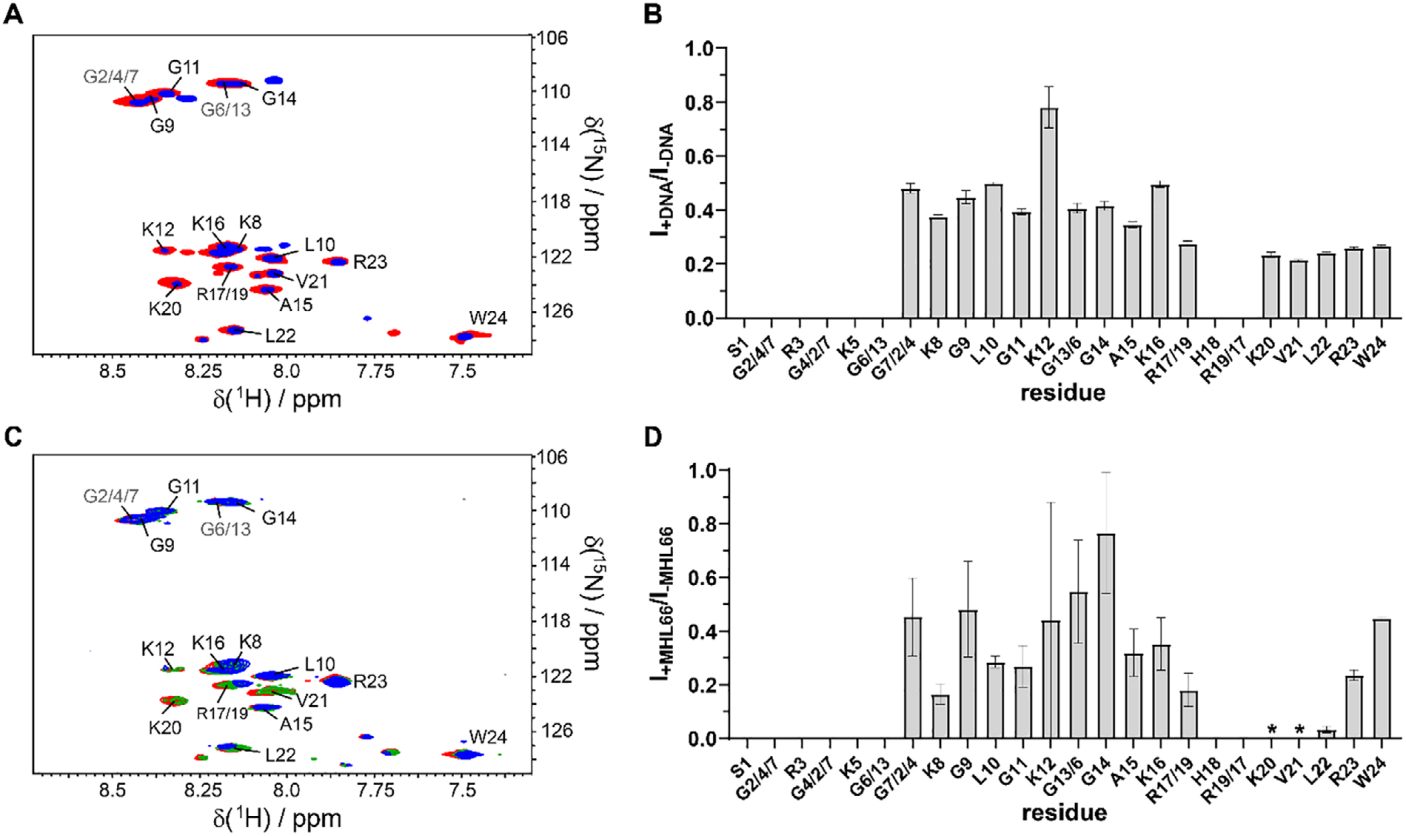
NMR Experiments: (A,C) ^15^N‐HSQC spectra of 150 µM H4_1‐23+W_ in 50 mM KPi, pH 6.5 at 25°C with assignment (black letters). Grey letters indicate an ambiguous assignment according to spectral overlap. H4 sequence: SGRGKGGKGLGKGGAKRHRKVLRW (DNA‐binding site: KRHRK). (A) Perturbation of histone resonances on DNA addition. Overlay of ^15^N‐HSQC spectra of H4 peptide (150 µM) before (red) and after titration of 20 µM DNA (blue). (B) Ratio of peak intensities of the ^15^N‐HSQC spectra after (I+DNA) and before (I‐DNA) the addition of 20 µM DNA. (C) NMR titration of the histone peptide with a tweezer peptide conjugate **2**. Overlay of ^15^N‐HSQC spectra of H4_1‐23+W_ (150 µM) before (red) and after titration of 50 µM **2** (green) and 200 µM **2** (blue). D) Ratio of peak intensities of the ^15^N‐HSQC spectra after (I+2) and before (I‐2) the addition of 200 µM **2**. The signals corresponding to K20 and V21 disappear (asterisks).

Since affinities of supramolecular phosphate tweezers with respect to arginine and lysine side chains of target proteins lie in the low micromolar range [43], they match the K_D_ values observed for our histone‐DNA complexes. Consequently, the above experiments evidence the selected peptide‐DNA system to be a valuable tool for studying and modulating histone‐DNA interaction using our tweezer molecules.

## 3 Optimization of Tweezers for Maximum Affinity and Selectivity Towards the Histone Peptide Target

Since the H4 histone peptide is almost exclusively composed of basic amino acids and glycines or leucines, the selectivity of an external binder means distinction between short fragments with high and low density of basic residues. Especially, targeting the DNA‐binding site K16‐K20 with advanced tweezers by imitating the acetylation state should weaken the histone‐DNA interaction. Hence, we decided to develop two series of novel tweezer derivatives: a) monomeric tweezer peptide conjugates with acidic residues and b) dimeric tweezer derivatives with various linkers for the short peptide stretch (H4_14‐22_) comprising four basic residues. Their affinity should later be compared to a distant peptide stretch (H4_6‐14_) of equal length with only two lysines. Initial molecular docking of the peptide stretch H4_14‐22_ complexed by a dimeric tweezer suggested that two tweezers could be placed on two lysines at position *i* and *i+4* and that their phosphate or attached anionic groups would engage in additional electrostatic interactions with the internal arginines (Figure S1). We hoped that appropriately positioned functional linkers could reinforce the whole arrangement.

A series of monomeric tweezers with attached acidic peptides (**2**, **3**, **4**) was synthesized employing the copper‐catalyzed click reaction between a butynyl tweezer **1** and an azidopeptide (Figure 3A) [44]. The aspartate and glutamate residues were intended to engage in additional ion pairs with lysines and arginines of the H4 histone peptide. As a typical example, binding of the tweezer‐peptide conjugate **2** to an H4 peptide (H4_1‐23+W_) is demonstrated by NMR chemical shift perturbation and the drop in peptide resonance intensities (^1^H,^15^N‐HSQC) in Figure 2C,D.

**FIGURE 3:**
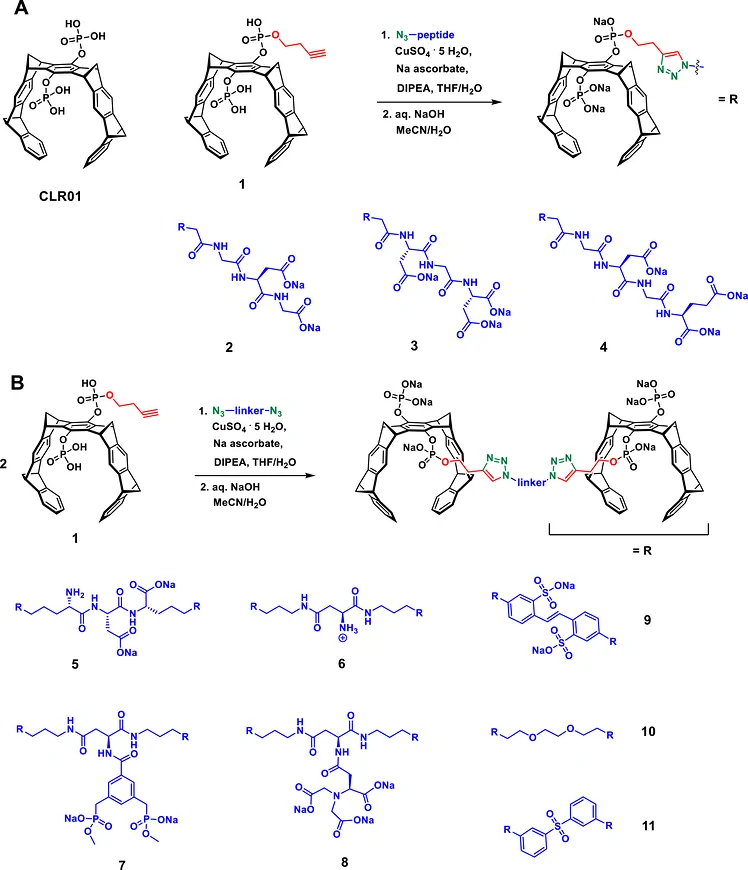
(A) Advanced monomeric tweezers **2, 3** and **4** with attached acidic peptides for the recognition of basic amino acids of the DNA‐binding site within the H4 histone peptide. All tweezer derivatives were prepared by the copper‐catalyzed click reaction between butynyl tweezer **1** and the respective azidopeptide. (B) Advanced dimeric tweezers with peptidic (**5**, **6**) and artificial (**7**, **8**, **9**, **10**, **11**) recognition sites tailored for the DNA binding site within the H4 histone peptide. All dimeric tweezer derivatives were prepared by the iterative click coupling between two eq. of butynyl tweezer **1** and the respective azidolinker (disodium‐4,4‘‐diazidostilbene‐2,2‘‐disulfonate tetrahydrate, 1,2‐bis(2‐azidoethoxy)ethane (PEG2), 3,3‐diazidodiphenylsulfone) followed by quantitative neutralization to water‐soluble sodium salts.

To improve binding, we also designed dimeric tweezers with internal peptide linkers (Figure 3B). To this end, two azidoornithines were coupled to the central aspartate, giving the Az‐Orn‐Asp‐Orn‐Az sequence. Two butynyl tweezer units were subsequently attached by double click coupling, employing an iterative coupling protocol developed earlier in our group (**5**) [45]. This design also allowed the introduction of other binding motifs in the central amino acid position: two azidopropylamine units were coupled to both carboxylic acids of Boc‐protected aspartate, and after Boc removal, the primary amine was used to introduce a bisphosphonate or an NTA moiety. *m*‐Xylylene bisphosphonates have been invented by us for arginine recognition [46]. and the NTA motif is well known for histidine recognition, forming a very stable octahedral Ni^2+^‐complex [47]. Double tweezer coupling finally gave the parent amine (**6**), and the respective bisphosphonate tweezer dimer (**7**) as well as the NTA tweezer dimer (**8**). Finally, three short linkers with electron‐rich functionalities were coupled to two tweezers, leading to very compact receptor molecules with sulfonate (**9**), glycol (**10**), and sulfone (**11**) elements. All synthesized dimeric tweezers are depicted in Figure 3B.

MD simulations indicated that all presented tweezers could in principle form stable complexes with the DNA binding site H4_14‐22_ via hydrogen bonds and π‐cation‐interactions. In each case, lysine side chains are trapped within tweezer cavities while arginine residues are complexed by the attached recognition motif (Figures S2–S4). Subsequently, binding studies were conducted to define the potency of the designed tweezers toward our target H4 peptide. Gaining information about the binding properties of each tweezer derivative would help us identify the best modulators of the histone‐DNA interaction.

The initial thought of using nickel(II) ions to induce octahedral complex formation between the NTA moiety of **8** and the histidine residue of H4_14‐22_ was soon discarded. Since the presence of nickel cations caused precipitation in solutions containing an equivalent amount of tweezer and peptide (data not shown)—most likely due to aggregation—further experiments with **8** were performed without nickel(II) ions.

The full series of new advanced tweezer derivatives was titrated with three N‐terminal fragments from the H4 histone peptide. To characterize the tweezer interaction with the DNA binding site in direct comparison with the distant fragment carrying only two lysines, we used two sequences of equal length, namely H4‐P4 (H4_6‐14_) and H4‐P1 (H4_14‐22_). Both carry two lysines at a 1,5‐distance, but the DNA binding site H4‐P1 also has two Arg and a central His. Both fragments are part of the full‐length N‐terminal 22mer histone peptide H4‐Full‐Length (H4_1‐22_), which constitutes the third titration partner (Figures 4 and S5A).

**FIGURE 4:**
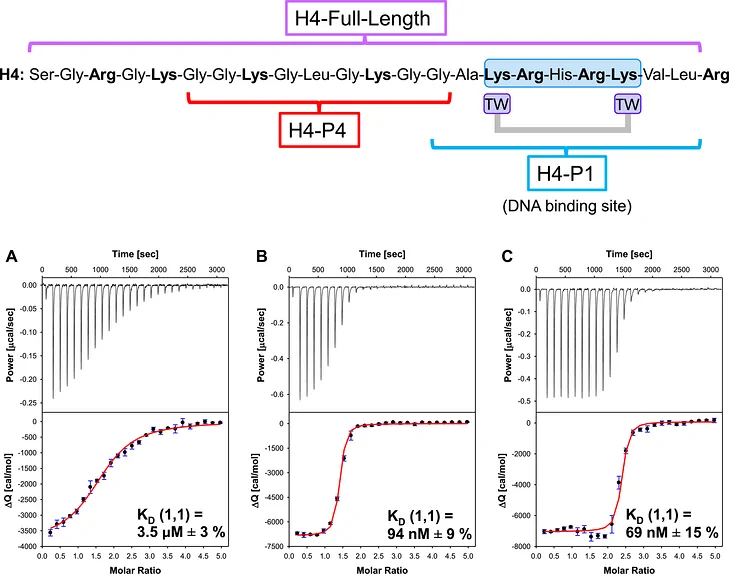
Sequence of the full‐length 22mer H4 peptide and two peptide fragments H4‐P1 and H4‐P4 investigated in this work. Both fragments carry two lysines at a 1,5‐distance. (A,B,C) ITC titrations of 20 µM to 30 µM H4 peptides (A) H4‐P4, (B) H4‐P1, and (C) H4‐Full with 500 µM **11** in potassium phosphate buffer (10 mM, pH 7.0). Note the very sharp ascent of the curve for H4‐P1 at 1.0 equiv. (1:1 complex) and for H4‐Full at 2.0 equiv. (2:1 complex), indicating very strong binding. ITC data were evaluated according to a 1SnS‐model (1 set of n sites, *AFFINImeter*). K_D_ (1,1): curve fitting for 1:1 complex. Further ITC data are available from Figures S6–S8.

ITC titrations were performed with all three histone fragments, and thermodynamic properties were recorded. For the DNA binding site and the full‐length H4 peptide, fluorescence titrations were also executed with FAM‐labeled peptides at very low concentrations in order to obtain precise K_D_ values for the best nanomolar binders. These experiments revealed exceptional affinities and selectivities for the DNA binding site and are without literature precedent (fluorescence titrations see below and full titration data in the Supporting Information). A 20–30 µM nonlabeled peptide (H4‐P1, H4‐P4 and H4‐Full‐Length) in the sample cell was titrated with aliquots of mono‐ or divalent tweezer. The resulting binding curves were analyzed using the independent sites approach of the *AFFINImeter* software (Cloud version v2.1802.5) [48]. The first series was conducted with the DNA binding site H4‐P1. **CLR01** was by far the weakest binder (K_D_ 6 µM), but already the three peptide binders were much better (K_D_ 300–500 nM), while the most powerful affinity (K_D_ 80–100 nM) was determined for compact artificial binding sites: NTA (**8**), sulfonate (**9**), and sulfone (**11**). Compared to the single tweezer **CLR01**, an additional anionic peptide attachment or a second tweezer unit strengthens the tweezer:peptide complexation. In all cases, 1:1 complexes were formed, and all were enthalpy‐driven, with small favorable entropy contributions (Table 1).

**TABLE 1:** ITC titrations of advanced tweezers with H4‐P1, i.e., the DNA binding site on H4. Stoichiometries, K_D_ values, and thermodynamic parameters were obtained by applying the independent sites approach. ITC data were evaluated according to a simple 1SnS‐model or a more flexible 2SnS‐model. K_D_ (1,1): Curve fitting for 1:1 complex.

Tweezer	Binding model	n (1,1)	K_D_ (1,1)	∆G	∆H_A_	−T*∆S
			nM	kcal mol^−1^	kcal/mol	kcal mol^−1^
**CLR01**	1SnS	1.1	6270 ± 6%	−7.1	−10.4	3.3
**2**	2SnS	1.4	518 ± 10%	−8.6	−6.8	−1.7
**3**	2SnS	1.4	317 ± 3%	−8.9	−6.9	−2.0
**4**	2SnS	1.4	284 ± 13%	−8.9	−7.3	−1.6
**5**	2SnS	1.3	404 ± 5%	−8.7	−6.2	−2.5
**6**	2SnS	1.2	564 ± 7%	−8.5	−8.6	0.1
**7**	2SnS	1.1	310 ± 21%	−8.9	−8.6	−0.3
**8**	2SnS	0.9	83 ± 8%	−9.7	−8.0	−1.7
**9**	2SnS	1.4	105% ± 12%	−9.5	−7.1	−2.4
**10**	1SnS	1.6	608 ± 10%	−8.5	−5.4	−3.0
**11**	1SnS	1.3	94 ± 9%	−9.6	−6.8	−2.7

The second series of ITC titrations was performed with the distant peptide fragment, which contains only two lysines, H4‐P4. Interestingly, K_D_ values were now in the micromolar range, not significantly lower than the parent tweezer **CLR01**. A notable exception was the compact binder **9**, which reached a 600 nM K_D_ value. Stoichiometries were between 1:1 and 2:1, indicating separate binding of both lysines. Unlike the previous peptide fragment, the absence of further basic amino acids in H4‐P4 resulted in moderate binding strength due to the lack of noncovalent interactions from additional recognition motifs. In this series, binding was much more entropy‐driven, even for the compact artificial binders, including **8** (Table 2).

**TABLE 2:** ITC titrations of advanced tweezers with H4‐P4, i.e., the distant fragment with only two lysine residues. Stoichiometries, K_D_ values, and thermodynamic parameters were obtained by applying the independent sites approach. ITC data were evaluated according to a simple 1SnS‐model. K_D_ (1,1): Curve fitting for 1:1 complex.

tweezer	Binding model	n (1,1)	K_D_ (1,1)	∆G	∆H_A_	−T*∆S
			µM	kcal mol^−1^	kcal/mol	kcal mol^−1^
**CLR01**	1SnS	1.8	18 ± 3%	−6.5	−5.2	−1.3
**2**	1SnS	1.5	24 ± 4%	−6.3	−5.5	−0.8
**3**	1SnS	1.7	10 ± 2%	−6.8	−4.7	−2.1
**4**	1SnS	1.4	12 ± 1%	−6.7	−5.0	−1.7
**5**	1SnS	1.7	4.5 ± 3%	−7.3	−3.8	−3.5
**6**	1SnS	1.8	47 ± 10%	−5.9	3.2	−9.1
**7**	1SnS	1.6	12 ± 3%	−6.7	−3.5	−3.2
**8**	1SnS	1.5	2 ± 7%	−7.8	−2.7	−5.1
**9**	1SnS	1.8	0.6 ± 2%	−8.5	−3.5	−5.0
**10**	1SnS	1.8	3.8% ± 3%	−7.4	−3.9	−3.5
**11**	1SnS	1.7	3.5 ± 3%	−7.4	−3.8	−3.6

Finally, the full‐length H4 peptide was employed in ITC titrations with the entire tweezer series. This time, K_D_ values were again mostly in the nanomolar range; only the peptide tweezers bound H4 more weakly (with low micromolar affinity). Higher stoichiometries were observed between 2:1 and 3:1, as can be expected due to the presence of several binding sites with two or more basic amino acids. Intriguingly, **11** with the sulfone unit binds even more strongly to full‐length H4 than to its DNA‐binding site (Figure 4). We tentatively explain this by back‐biting into a second binding site of the extended multivalent peptide strand (Table 3 and Figure S6). Most binding events with full‐length H4 were again enthalpy‐driven.

**TABLE 3:** ITC titrations of advanced tweezers with the full‐length H4 peptide (H4_1‐22_). Stoichiometries, K_D_ values, and thermodynamic parameters were obtained by applying the independent sites approach. ITC data were evaluated according to a simple 1SnS‐ or a more flexible 2SnS‐ and 3SnS‐model. K_D_ (1,1): Curve fitting for 1:1 complex.

tweezer	Binding model	n (1,1)	K_D_ (1,1) [nM]	∆G	∆H_A_	−T*∆S
				kcal mol^−1^	kcal/mol	kcal mol^−1^
**CLR01**	2SnS	3.3	745 ± 3%	−8.4	−6.3	−2.1
**2**	2SnS	3	2693 ± 12%	−7.6	−4.7	−2.9
**3**	2SnS	3.6	2274 ± 10%	−7.7	−4.8	−2.9
**4**	2SnS	2.8	1777 ± 5%	−7.8	−5.1	−2.7
**5**	3SnS	2.2	591 ± 12%	−8.5	829.1	−837.6
**6**	1SnS	2.4	563 ± 6%	−8.5	−8.0	−0.6
**7**	3SnS	2.1	1158 ± 8%	−8.1	−13.3	5.2
**8**	3SnS	1.7	491 ± 10%	−8.6	71.3	−79.9
**9**	2SnS	1.9	258 ± 8%	−9.0	−8.9	−0.1
**10**	1SnS	2.8	188 ± 3%	−9.2	−6.2	−2.9
**11**	1SnS	2.3	69 ± 15%	−9.8	−7.1	−2.6

Taken together, the ITC titrations reveal powerful binding, especially of the compact dimeric tweezers with artificial linkers both to H4‐P1 as well as to full H4. Binding is specific and must involve multiple attractive noncovalent interactions. The best binders reach almost two orders of magnitude lower K_D_ values than **CLR01**. A high preference for H4‐P1 (nanomolar) over H4‐P4 (micromolar) documents the success of our concept, namely, generating new tweezer derivatives, which specifically target the DNA binding site on full‐length H4.

Especially for the most powerful binders, which produced very steep binding curves, fluorescence titrations were also conducted with the full series of tweezers and H4‐P1 as well as full‐length H4. To this end, the peptides were FAM‐labeled, and complex formation was monitored by fluorescence quenching of the label emission. Binding curves were again very steep, so that all titrations were finally performed at 1 µM peptide concentration (Figure 5A,B; Supporting Information).

**FIGURE 5:**
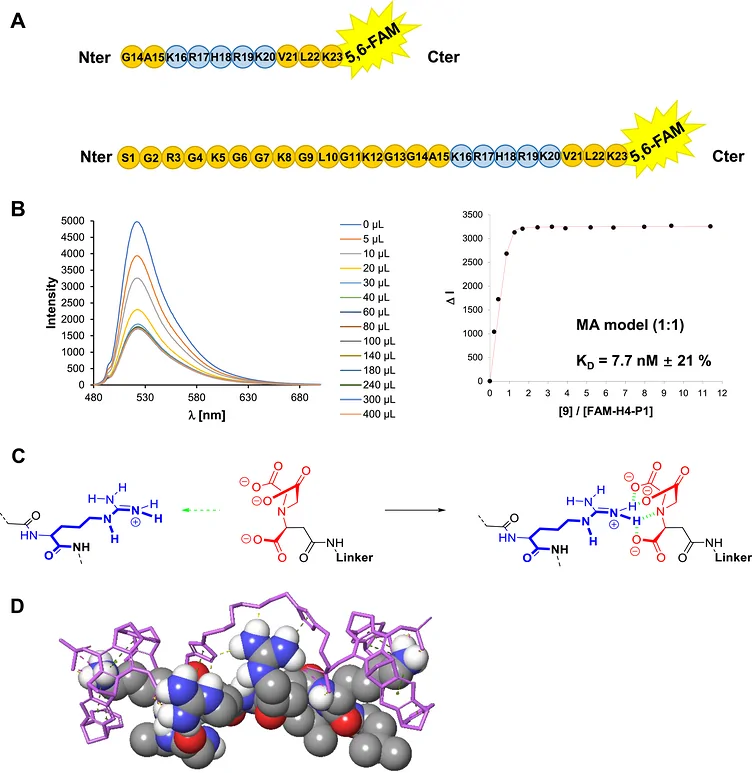
(A) FAM‐labeled DNA binding site and full H4 peptide. (B) Fluorescence titration of 1 µM FAM‐H4‐P1 with the best binding dimeric tweezer **9**. Note the strong fluorescence quenching and very sharp kink at 1.0 equivs., indicating a strict 1:1 stoichiometry of the histone peptide/tweezers complex. Further data can be found in the Supporting Information. (C) Potential recognition of the cationic arginine residue (blue) by the negatively charged NTA moiety (red). (D) Modeling picture: **10** forming a crown‐ether type complexation of a guanidinium cation of a central arginine in H4‐P1.

At 1 µM peptide, all higher aggregates seem to be suppressed in favor of a clean 1:1 complexation (Job plots) [49, 50, 51]. Consequently, very strong binding is observed (with very small errors), and some K_D_ values reach the low nanomolar range. Peptidic linkers are powerful at recognizing the DNA binding site (H4‐P1), but especially the dimeric tweezer **9** displays an exquisite affinity to both H4‐P1 and H4‐Full (8 nM K_D_ and 23 nM K_D_). These values are without literature precedent and demonstrate a superb match of tweezer and linker functionalities and a high preference for the lysine‐ and arginine‐rich DNA‐binding site on the histone peptide (Table 4). Characteristic new binding motifs between linker units and basic residues were discovered in extended MD simulations (discrete water, 300K, 100 ns).

**TABLE 4:** Fluorescence titrations of labeled FAM‐H4‐P1 and FAM‐H4‐Full‐Length with molecular tweezers and the resulting stoichiometries as well as K_D_ values [nM] (*AFFINImeter*). 30–32 µM Tweezer solutions were added in aliquots to a 1 µM peptide in potassium phosphate buffer (10 mM, pH 7.0).

Tweezer	Binding model	Stoichiometry	FAM‐H4‐P1 K_D_ [nM]	FAM‐H4‐Full K_D_ [nM]
**CLR01**	MA	1:1	638 ± 3%	553 ± 1%
**2**	MA	1:1	28 ± 18%	328 ± 8%
**3**	MA	1:1	44% ± 22%	205 ± 3%
**4**	MA	1:1	17 ± 21%	209 ± 11%
**5**	MA	1:1	19 ± 25%	136 ± 4%
**6**	MA	1:1	398 ± 2%	78 ± 2%
**7**	MA	1:1	110 ± 13%	250 ± 2%
**8**	MA	1:1	47 ± 9%	54 ± 7%
**9**	MA	1:1	7.7 ± 21%	23 ± 32%
**10**	MA	1:1	122 ± 4%	76 ± 15%
**11**	MA	1:1	286 ± 2%	23 ± 36%

Modeling studies generated interesting additional contacts between the linkers of the best binders and the H4‐P1 peptide, which led to attractive interactions and expelled ordered water molecules; these may in part explain their remarkable affinities. Thus, hydrogen‐bond‐enforced coordination of the arginine guanidinium cation by the NTA moiety of NTA tweezer **8** creates a new recognition motif (Figure 5C). Formation of an octahedral complex between the NTA unit of **8** and the histidine of H4‐P1 via Ni^2+^ is not necessary in this case to achieve strong binding. Arginine residues of H4‐P1 can both be recognized by two negative sulfonate groups of **9** via H bonds (Figure S4). A complexation of one arginine residue would be possible with the PEG linker forming a crown ether where the glycol and triazol units interact with the guanidinium cation via π‐cation and H bonds (Figure 5D).

In the case of **11**, the diphenylsulfone linker reduces the distance between two tweezer units by taking the form of a roof‐shaped structure upon lysine binding. Consequently, both arginine residues may be complexed by the tweezer phosphate groups via hydrogen bonds (Figure S4).

Unfortunately, it is not trivial to pinpoint these structures in a highly dynamic complex with averaged NMR signals, even in NOESY experiments. We and others have, however, found such motifs earlier in crystal structures of related complexes between linkers of our supramolecular binders and various proteins [52, 53].

Taken together, ITC and fluorescence titrations both indicate very strong binding of dimeric tweezers to the DNA binding site and also to full‐length H4 histone peptide. The absolute K_D_ values obtained from fluorescence titrations are superior to those determined by ITC; however, here the concentrations were much higher, and clean 1:1 complexes were formed at 1 µM concentration. Job plots provide experimental evidence for this; only fluorescence titrations with full‐length H4 histone peptide indicate a shift toward partial formation of 2:1 complexes.

## 4 Modified Tweezers Interfere With Histone‐DNA Complex Formation

For a functional test, we intended to determine the dose‐dependent inhibitory influence of optimized tweezers on the stability of histone‐DNA complexes. The ability of modified tweezers to interrupt the essential histone‐DNA interaction will provide a measure of their epigenetic modulation efficacy. To this end, complex formation of DNA and FAM‐labelled histone‐peptide (H4m) was monitored in the presence of tweezers using fluorescence anisotropy. After DNA‐binding was approaching saturation level (r_495/518_ = 0.07), the tweezers **5** were added (Figure 6A).

**FIGURE 6:**
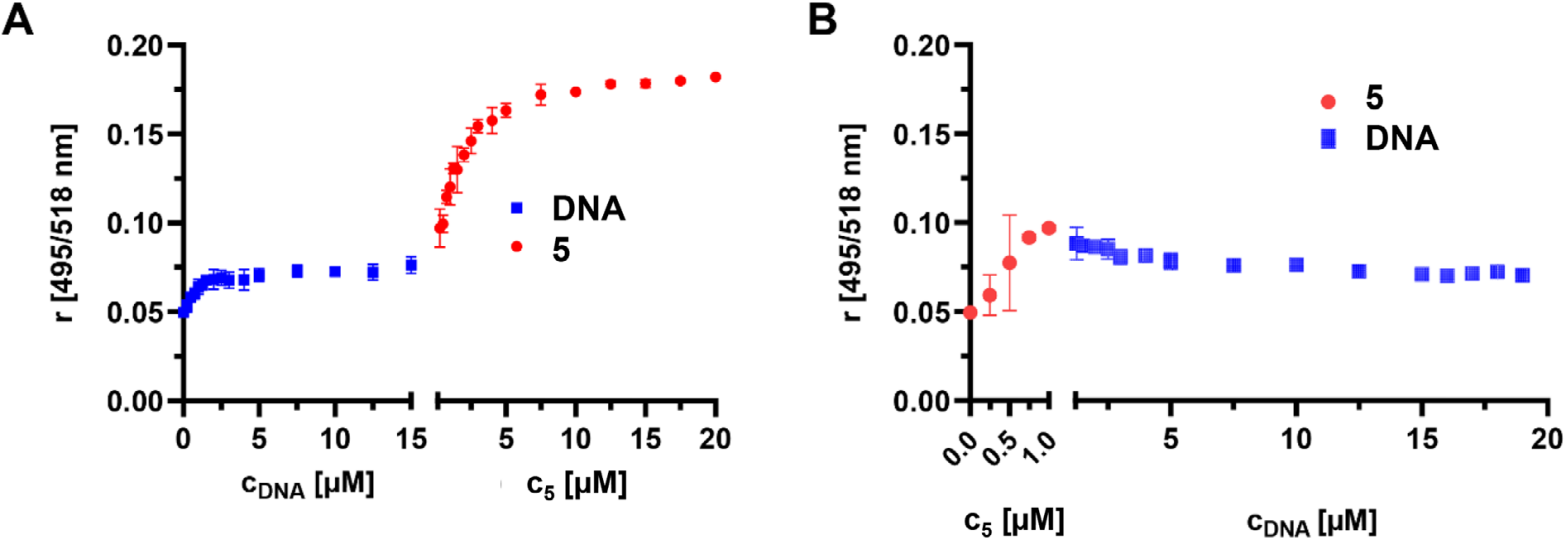
Competition experiments with histone peptide, DNA and tweezers. Fluorescence anisotropy of 0.1 µM H4m was monitored. (A) First, DNA was added up to 15 µM (blue squares), then tweezers **5** was titrated up to 20 µM (red dots). (B) Reverse competition experiment with regard to A. First, titration with up to 1 µM tweezers **5** (red dots) was performed, then DNA was added with up to 19 µM (blue squares).

The intensity increase of the anisotropy signal upon addition of tweezers (at saturation level r_495/518_ = 0.18) demonstrates tweezer‐histone complex formation. However, binding of either tweezer or DNA contributes to an enhanced anisotropy value. Therefore, the anisotropy increase upon tweezers addition cannot be attributed to the disassembly of the histone‐DNA complex unambiguously, but may also be explained by the formation of a ternary complex composed of DNA, tweezers, and histone.

Figure 6 reveals that the amplitudes of signal increase upon saturation of histone by either tweezers or DNA are different; tweezers induce a higher amplitude change than the addition of DNA. If the whole experiment is carried out in reverse order, saturation of histone with tweezer molecules should first lead to a gain in amplitude. Subsequent addition of DNA would then generate a decline of the anisotropy signal until saturating DNA concentrations are reached. This complexation behavior was actually observed (Figure 6B); it strongly points to a competitive process where the negatively charged DNA displaces tweezer molecules from their preformed complex on the histone peptide. In turn, the reverse happened in the original experiment (Figure 6A): here, tweezers displaced DNA from the histone‐DNA complex.

Modulation of epigenetic information is obtained by the presence and activity of enzymes that acetylate or deacetylate specific lysine residues on the histone tails. Since we intend to model covalent acetylation of lysines by noncovalent lysine capping, our optimized tweezers must not only compete with DNA but also with specific histone acetyl transferases (HATs) for the respective lysine binding sites on the histone surface. We therefore investigated the potential of selected tweezers with high affinity towards H4 to compete for lysine binding sites on HATs and/or to impair HAT function.

## 5 Modified Tweezers Compete With the Acetylation Machinery

To test the influence of modified tweezers on HAT activity, we used a commercially available histone acetyltransferase activity assay kit. The HAT Assay Kit contains a purified recombinant active p300 catalytic domain protein, histone H4‐N‐terminal substrate peptide, anacardic acid as a control inhibitor, and all essential buffers. The assay is used to determine the activity of HATs or potential inhibitors of HAT activity. Structurally representative dimeric tweezers (**5**, **10**, **11**) were tested for their ability to compete with HATs for H4 binding. The experiment aimed to achieve competitive inhibition of histone acetylation by addition of supramolecular peptide binders. Therefore, the HAT protein p300 was incubated with acetyl‐CoA and the histone H4 substrate peptide. During this reaction, the HAT enzyme catalyzes the transfer of acetyl groups from acetyl‐CoA to the histone peptide, resulting in the acetylated peptide and CoA‐SH. After stopping the reaction, all CoA‐SH sulfhydryl groups are quantified by adding a SH‐reactive fluorescent dye and detecting its signal.

Our experiments confirmed the competition of our tweezer derivatives with the HAT acetylase machinery, not only in a dose‐dependent manner but also at different H4 concentrations. Figure 7A,B reveal the different efficiencies of dimeric tweezers as HAT inhibitors, with **5** inhibiting only weakly, **11** moderately and **10** strongly. At equimolar concentrations of tweezer inhibitor **10** and substrate histone peptide (50 µM), the same inhibition level is reached as with anarcadic acid, an established p300 inhibitor [54].

**FIGURE 7:**
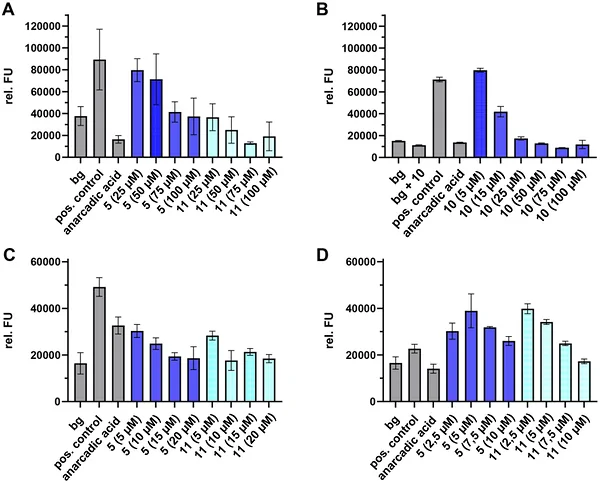
Acetylase assays performed with the molecular tweezers in the presence of 17 nM p300 and different concentrations of H4. (A) **5** (dark blue) and **11** (light blue; both 25–100 µM) and **50 µM H4**. (B) **10** (dark blue; 5–100 µM) with **50 µM H4**. (C) **5** (dark blue) and **11** (light blue; both 5–20 µM) with **10 µM H4**. **D**, **5** (dark blue) and **11** (light blue; both 2.5–10 µM) with **5 µM H4**. bg: background, anarcadic acid: inhibitor of p300 (grey bars: controls without tweezers).

If occupation of binding sites on the histone peptide determines the level of tweezer inhibition, IC_50_ values should drop when the H4 concentration is lowered. This was indeed the case: HAT inhibition occurred at much lower tweezer concentrations (Figure 7C,D). This rules out a direct complexation on p300 as the origin of HAT inhibition, since in all experiments tweezers (up to 10, 20 and 100 µM) are used in a large excess of the enzyme (17 nM). Earlier experiments with other enzymes also required a very high CLR01 excess (ADH:CLR01 = 1:865, PARP‐1:CLR01 = 1:1435) to reach half‐maximal inhibition and render direct p300 inhibition by tweezer dimers highly unlikely [55, 56].

## 6 Conclusion

In order to target the DNA‐binding site on histone peptide H4, a large number of novel tweezer derivatives were synthesized. The high density of basic amino acids inside the H4‐P1 fragment was addressed by an acidic peptide stretch attached to the parent tweezer CLR01 by a click protocol. Its charge complementarity indeed greatly enhanced its affinity towards this lysine and arginine cluster. In addition, various artificial binding motifs for lysines, arginines, and histidines were introduced as central linkers into a dimeric tweezer construct by a newly established iterative click reaction. These dimeric tweezers turned out to be highly selective for H4‐P1, the DNA‐binding site, and displayed exquisite affinities in phosphate buffer reaching the one‐digit nanomolar regime. With the best binders, a competitive displacement of DNA bound to the histone peptide was demonstrated. In addition, stoichiometric amounts of dimeric tweezers complexed the DNA‐binding site of the histone peptide and inhibited the enzymatic activity of HAT in a dose‐dependent manner. Thus, powerful new histone binders were developed that act as supramolecular PTM on a histone H4 peptide.

In a more realistic setting such as nucleosomes, H4 tails must be distinguished from H3 tails and other PTMs such as arginine or lysine methylation. In this respect, our tweezers do not show elevated affinity towards methylated arginine, and only comparable affinity to methylated lysine, as we already determined in separate NMR titrations [57]. It will be interesting to evaluate how the binding of the present compounds to H4 is affected in the presence of potentially competing peptides, such as H3‐derived peptides and methylated histone peptides. Such experiments are on our agenda for the future.

## 7 Materials and Methods

### 7.1 Design and Provision of H4 Peptides and DNA From Calf Thymus

Non‐acetylated and isotopically unlabeled *N*‐terminal peptides derived from H4 and C‐terminally labelled with a 5,6‐FAM dye (residues 7–22 + LysFAM; GKGLGKGGAKRHRKVLK(5,6‐FAM)‐NH_2_) were purchased from Caslo (Denmark). A clone for recombinant expression of an H4 peptide (residues 1–23 + W + 6His; SGRGKGGKGLGKGGAKRHRKVLRW) was purchased from Geneart/Invitrogen (Germany) in a pMA‐T shuttle vector (cloning sites EcoRI and XhoI, sequence optimized for *Escherichia coli* host and GC content). The Trp at position 24 was included to provide signal intensity for adsorption‐sensitive recording during the purification procedure.

A palindromic double‐stranded 30 bp nucleosomal DNA (sequence 5’‐ GCATGCTAATTCCGTTGTCAGCGATCTCGATTTTTCGAGATCGCTGACAACGGAATTAGCATGC ‐3’), whose sequence was derived from previous experimental work [38], was chemically synthesized by Invitrogen (Germany).

### 7.2 Optimization of Peptide‐DNA Complexes

For a detailed and unambiguous characterization of the histone:DNA complex, peptide and DNA sequences were kept as short as possible. The reduced melting temperature of a short DNA double strand was compensated by the use of an inbuilt tetra‐thymidine element linking the pairing sequences of the single‐stranded palindromic DNA stretches [58]. Melting temperatures of these complexes are in the range of 60°C–70°C. The proper K_D_ range for optimal spectroscopic monitoring was tuned by varying the salt concentrations within the buffer solutions.

### 7.3 Expression of Peptide

For recombinant expression, the sequence of the H4_1‐23+W_ was cloned into a pET‐SUMOadapt vector using the restriction sites BsaI and BamHI. The insertion was performed via blunt ends, thereby preserving the SUMO fusion protein in the final construct. For NMR spectroscopy, the ^15^N‐labeled H4_1‐23+W_ peptide (2.4 kDa) was recombinantly expressed as SUMO‐H4 fusion peptide (13.6 kDa) and, after cleavage with SUMO protease, purified by FPLC.

### 7.4 Fluorescence Anisotropy Studies of Histone‐DNA and Tweezer‐Histone Interaction

For optical spectroscopic studies, non‐acetylated peptides were labelled with Fluorescein or, preferentially 5,6‐FAM tags. To determine host:peptide affinities, the increase in fluorescence anisotropy was monitored in the presence of increasing amounts of DNA (FP technique). K_D_ values were obtained by fitting this data to an equation for a 1:1 saturation binding model process. A potassium phosphate buffer (50 mM, pH‐Wert 6.5) was used.

$$F=F_{max}\;\cdot \;\frac{\left(P+L+K_{D}\right)\;-\;\sqrt{{\left(P+L+K_{D}\right)}^{2}\;-\;(4\;\cdot \;L\;\cdot \;P)}}{2\;\cdot \;P}$$

where, F, observed signal amplitude (y–coordinate); Fmax, saturation amplitude–baseline signal at X = 0; P, peptide concentration; L, concentration of tweezers or DNA (x–coordinate).

### 7.5 Competition Experiment

Experiments were carried out on a FP8300 Spectrofluorimeter (Jasco). Complex formation/disintegration of DNA or tweezers and 0.1 µM FAM‐labelled histone‐peptide (H4m) in potassium phosphate buffer (50 mM, pH‐Wert 6.5) were monitored using fluorescence anisotropy. For the competition experiment, first DNA was added up to 15 µM (saturation level at r_495/518_ = 0.07), then tweezers were subsequently titrated up to 20 µM. For the reverse competition experiment, tweezers were first titrated up to 1 µM while DNA was subsequently added up to 19 µM.

### 7.6 Acetylase Assay

We measured HAT activity using a commercially available histone acetyltransferase activity assay kit (Active Motif, Waterloo, Belgium). The HAT Assay Kit contains a purified recombinant active p300 catalytic domain protein, histone H4‐N‐terminal substrate peptide, anacardic acid as a control inhibitor, and all essential buffers. The assay was used to determine the activity of HATs in the absence and presence of potential inhibitors. Derivatives of the molecular tweezers CLR01 (**5** & **10** & **11**) were tested for their ability to compete with HATs for histone binding. To achieve inhibition of histone acetylation by addition of tweezers, HAT protein p300 is incubated with acetyl‐CoA and histone H4 substrate peptide. During this reaction, the HAT enzyme catalyzes the transfer of acetyl groups from acetyl‐CoA to the histone peptide, resulting in the acetylated peptide and CoA‐SH. After stopping the reaction, all CoA‐SH sulfhydryl groups are quantified by adding a SH‐reactive fluorescent dye and detecting its signal (SpectraMax M5, Ex375/ Em460, at RT).

### 7.7 Synthesis of Monovalent Peptide Tweezers (Simple Click Procedure)

Under an argon atmosphere, monobutynyl tweezer **1** (1 eq.) and the respective azido‐peptide (2.2‐2.6 eq.) were dissolved in dry THF (2 mL). Then doubly‐distilled (dd) H_2_O (1 mL) and DIPEA (8.77 mg, 11.9 µL, 0.07 mmol, 5.4 eq.) were added to the reaction mixture. Following this, a catalysis solution of copper sulfate pentahydrate (8.30 mg, 0.03 mmol, 2.6 eq.) and sodium ascorbate (13.0 mg, 0.07 mmol, 5.1 eq.) in dd H_2_O (1 mL) was prepared, and the resulting yellow suspension was added immediately to the reaction solution. After stirring for 16 h at room temperature, the entire consumption of monobutynyl tweezer **1** was confirmed via HPLC analysis. The organic solvent was then removed under vacuum, and the remaining aqueous residue was lyophilized overnight. Subsequently, the purification of the crude product was achieved by preparative HPLC column chromatography (H_2_O/MeCN + 0.1% TFA; gradient). After removing the solvent under vacuum and followed by another lyophilization overnight, the desired product could be obtained as the free phosphoric acid. For deprotonation to the sodium salt, the free phosphoric acid was dissolved in MeCN/H_2_O (1:1). After the addition of aq. NaOH (1 eq. for each acid group), the transparent solution was stirred for 1 h at room temperature. After removing the solvent under vacuum, the aqueous residue was lyophilized overnight. Afterwards, the desired product could be obtained as the sodium salt.

### 7.8 Synthesis of Dimeric Tweezers With Functional Linkers (Iterative Double Click Procedure)

Under an argon atmosphere, monobutynyl tweezer **1** (1 eq.) and the respective azido‐linker (1 eq.) were dissolved in dry THF (2 mL). Then doubly‐distilled (dd) H_2_O (1 mL) and DIPEA (8.77 mg, 11.9 µL, 0.07 mmol, 5.4 eq.) were added to the reaction mixture. Following this, a catalysis solution of copper sulfate pentahydrate (8.30 mg, 0.03 mmol, 2.6 eq.) and sodium ascorbate (13.0 mg, 0.07 mmol, 5.1 eq.) in dd H_2_O (1 mL) was prepared, and the resulting yellow suspension was added immediately to the reaction solution. After stirring for 16 h, the reaction progress was controlled via HPLC analysis. After confirming the full consumption of the starting materials, another equivalent of monobutinyl tweezer **1** (1 eq.) and a second charge of the reagents, i.e., DIPEA (8.77 mg, 11.9 µL, 0.07 mmol, 5.4 eq.), dry THF (1 mL), copper sulfate pentahydrate (8.30 mg, 0.03 mmol, 2.6 eq.) and sodium ascorbate (13.0 mg, 0.07 mmol, 5.1 eq.) dissolved in ddH_2_O (1 mL), were added to the existing solution. The yellow mixture was stirred for at least another 17 h until the completive conversion of monobutinyl tweezer **1** was confirmed via HPLC analysis. The organic solvent was then removed under vacuum, and the remaining aqueous residue was lyophilized overnight. Last, the purification of the crude product was achieved by either preparative HPLC column chromatography or washing by centrifugation (SI). After removing the solvent in vacuo and subsequent lyophilization, the desired product was obtained as the free phosphoric acid. For deprotonation to the sodium salt, the free phosphoric acid was dissolved in MeCN/H_2_O (1:1). After the addition of aq. NaOH (1 eq. for each acid group), the transparent solution was stirred for 1 h at room temperature. After removing the organic solvent under vacuum, the aqueous residue was lyophilized overnight. The desired product could be obtained as the sodium salt.

### 7.9 ITC Titrations Between Tweezers and Histone Fragments

ITC titrations were carried out using a MicroCal iTC200 (GE Healthcare) operated with the software ITC200 Version 1.26. Guest and host solutions were each prepared in potassium phosphate buffer (10 mM, pH 7.0) containing 5.36 mM K_2_HPO_4_, 4.64 mM KH_2_PO_4_, and 10 mM NaCl. A 20–30 µM nonlabeled peptide (H4‐P1, H4‐P4, and H4‐Full‐Length) in sample cell was titrated with 500 µM mono‐ or divalent tweezer in titration syringe. As for CLR01, 1 mM tweezer and 50 µM peptide were used. Each experiment covered 26 successive injections with 120 s time intervals between each 1.5 µL injection (except the initial one with 0.4 µL). The temperature was maintained constant at 25°C, and the reference power was set at 5 μcal/sec. All obtained run data were analyzed with the *AFFINImeter* software (Cloud‐Version v2.1802.5) using the independent sites approach (1SnS, 2SnS, 3SnS, SI). In most cases, the simple 1SnS‐model (1 set of n sites) gave an excellent fit. For solvent control, separate measurements were done with tweezer adding to the sample cell containing only buffer solution. In case of **6** and **10**, the heat of dilution was deducted from each data point to obtain processable binding isotherms.

### 7.10 Fluorescence Titrations Between Tweezers and Histone Fragments

For quantifying the binding strength between tweezers and histone peptides, fluorescence titrations were performed on an RF‐6000 Spectro Fluorophotometer (Shimadzu) operated with the software *Shimadzu* LabSolutions RF Version 1.12. FAM‐labeled H4‐P1 and H4‐Full‐Length were each titrated with the respective tweezer derivatives. For the titration, the peptide (H4‐P1, H4‐Full‐Length) was prepared in potassium phosphate buffer (10 mM, pH‐Wert 7.0) containing 5.36 mM K_2_HPO_4_, 4.64 mM KH_2_PO_4_ and 10 mM NaCl at c = 1 µM. The tweezer was dissolved in the peptide solution (c = 30‐32 µM) to afford a constant peptide concentration over the entire titration experiment.700 µL of the peptide solution was placed in a quartz cuvette (1 cm) from *Hellma Analytics* (10 x 4 mm). The first fluorescence measurement on the spectrophotometer provided the initial fluorescence spectrum without tweezer addition (λ_Ex_ = 495 nm, λ_Em_ = 524 nm). Afterwards the tweezer solution was added in increasing amounts (0‐100 µL), leading to final host/guest ratio of ∼12:1. Followed by each addition, the resulting emission spectra were recorded and the changes in maximum emission intensity were quantified. All obtained fluorescence data were processed using the *Spectroscopy (SPEC) s*oftware of *AFFINImeter* (Cloud‐Version, Version v2.1802.5). Binding isotherms were generated by applying the stoichiometric MA binding model for 1:1 complexes. Finally, all binding parameters (K_A_/K_D_, Δδ_max)_ were derived by nonlinear regression from the resulting binding isotherms.

ΔI=ΔImaxGt·12·Ht+Gt+1K14·Ht+Gt+1K2−Ht·Gt−14·Ht+Gt+1K2−Ht·Gt

where, ∆IChanges of fluorescence intensities during titration; KBinding constant; [H]_t_, total concentration of host molecule; [G]_t_, total concentration of guest molecule.

### 7.11 Job Plot Method to Determine the Tweezer‐Peptide Complex Stoichiometry

The job plot method was used to gain information about the complex stoichiometry between tweezer and peptides. Fluorescence titrations were performed on an RF‐6000 Spectro Fluorophotometer (Shimadzu) operated with the software *Shimadzu* LabSolutions RF Version 1.12. Tweezer and peptide were dissolved separately in potassium phosphate buffer (10 mM, pH‐Wert 7.0) containing 5.36 mM K_2_HPO_4_, 4.64 mM KH_2_PO_4_, and 10 mM NaCl at c = 10 µM. A quartz cuvette (1 cm) from *Hellma Analytics* (10 x 4 mm) was first filled with 600 uL FAM‐labeled peptide. For each titration step, a certain amount of the cuvette solution was removed, and the same volume of tweezer solution was added. This way, the total volume and concentration in the cuvette remained unchanged. After each titration step, the resulting emission spectra were recorded. Then the changes in the maximum emission intensities were quantified to generate the job plot curves. Finally, complex stoichiometry was determined from the position of the curve maximum.

### 7.12 Molecular Modeling and MD Simulations of Peptides, Advanced Tweezers and Their Complexes

For the visualization of molecules as well as host‐guest complexes, molecular modeling was performed on *Maestro Schrödinger* Software 2019‐4 (Maestro Version 12.2.012). Respective guest and host structures were combined, and the resulting complexes were minimized using the OPLS_2005 force field. For the investigation of complex stability, the minimized structures were positioned in an orthorhombic box (at least 30 Å in all directions) containing discrete water molecules. Negative charges in the structures were neutralized by adding sodium ions. The prepared system was minimized with 500 ps first before carrying out the MD simulation at 298.15 K and 1013 mbar for 100 ns.

### 7.13 NMR Measurements

NMR measurements were performed on a 700 MHz Bruker Avance spectrometer equipped with a cryo‐probe. Assignments of peptides were performed by recording the following 2D and 3D spectra of ^15^N‐labelled or ^13^C‐^15^N‐labelled histone peptide H4_1‐23+W_ in 50 mM KPi, pH 6.5 at 25°C, respectively: ^15^N‐HSQC (2D), ^15^N‐Best‐TROSY (2D), HNCACB (3D), HN(CO)CACB (3D), HNCO (3D), HN(CA)CO (3D), ^15^N‐HSQC‐TOCSY). Owing to strong overlap of signals, only a partial assignment could be achieved. Peak intensities of resonances in ^15^N‐HSQC spectra of 150 mM H4_1‐23+W_ were calculated using the Bruker Topspin software. Ratios of peak intensities of the ^15^N‐HSQC‐spectra were determined before and after addition of 20 µM DNA.

## Author Contributions

P.B., T.S., and A.M.: conceptualization and supervision. M‐H.L., B.K., A.M., V.M., S.S., J.V., and M.B.: investigation, data curation, and analysis. M‐H.L. and B.K.: software and data analysis. A.M. and C.B.: visualization. P.B. and T.S.: funding acquisition. A.M., P.B., and T.S.: writing – original draft. A.M., C.B., T.S., and P.B.: writing – review and editing.

## Conflicts of Interest

The authors declare are no conflicts of interest.

## Supporting information




**Supporting File 1**: chem71588‐sup‐0001‐SuppMat.pdf.

## Data Availability

The data that support the findings of this study are available in the supplementary material of this article.
