# Supplementary material for: Modulation of Epigenetic Control by Advanced Molecular Tweezers
Source: Chemistry. 2026 Aug 19;32(42):e71588. doi: 10.1002/chem.71588 (PMC13755142; doi:10.1002/chem.71588)
Supplement: Supplementary file 1 [file CHEM-32-e71588-s001.pdf]

SUPPORTING INFORMATION

Modulation of Epigenetic Control by Advanced Molecular Tweezers

My-Hue-Le[1], Bianca Kamba[2], Anja Matena[2], Christine Beuck[2], Viktoria Momand[2], Spiros Salvanos[2], Jessica Vossen[2], Mike Blueggel[2], Thomas Schrader[1],* Peter Bayer[2]*

[1] Institute of Organic Chemistry I, Faculty of Chemistry, University of Duisburg-Essen, Universitätsstr. 7, 45117 Essen. thomas.schrader@uni-due.de
[2] Structural and Medicinal Biochemistry, Faculty of Biology, University of Duisburg-Essen, Universitätsstr. 2, 45141 Essen. peter.bayer@uni-due.de

Table of Contents

Molecular Modeling and MD Simulations…………………………………………………… 2
ITC Titrations……………………………………………………………………………... 8
Fluorescence Titrations……………………………………………………….…………… 14
Job Plots………………………………………………………………….………………. 36
Materials and Methods…………………………………………………….……………… 43
Synthesis of monovalent molecular tweezers………………………………………….... 44
Synthesis of divalent molecular tweezers……………………………………………….. 47
NMR spectra……………………………………………………………………….…….. 52
References……………………………………………………………………………….. 85

Molecular Modeling and MD Simulations

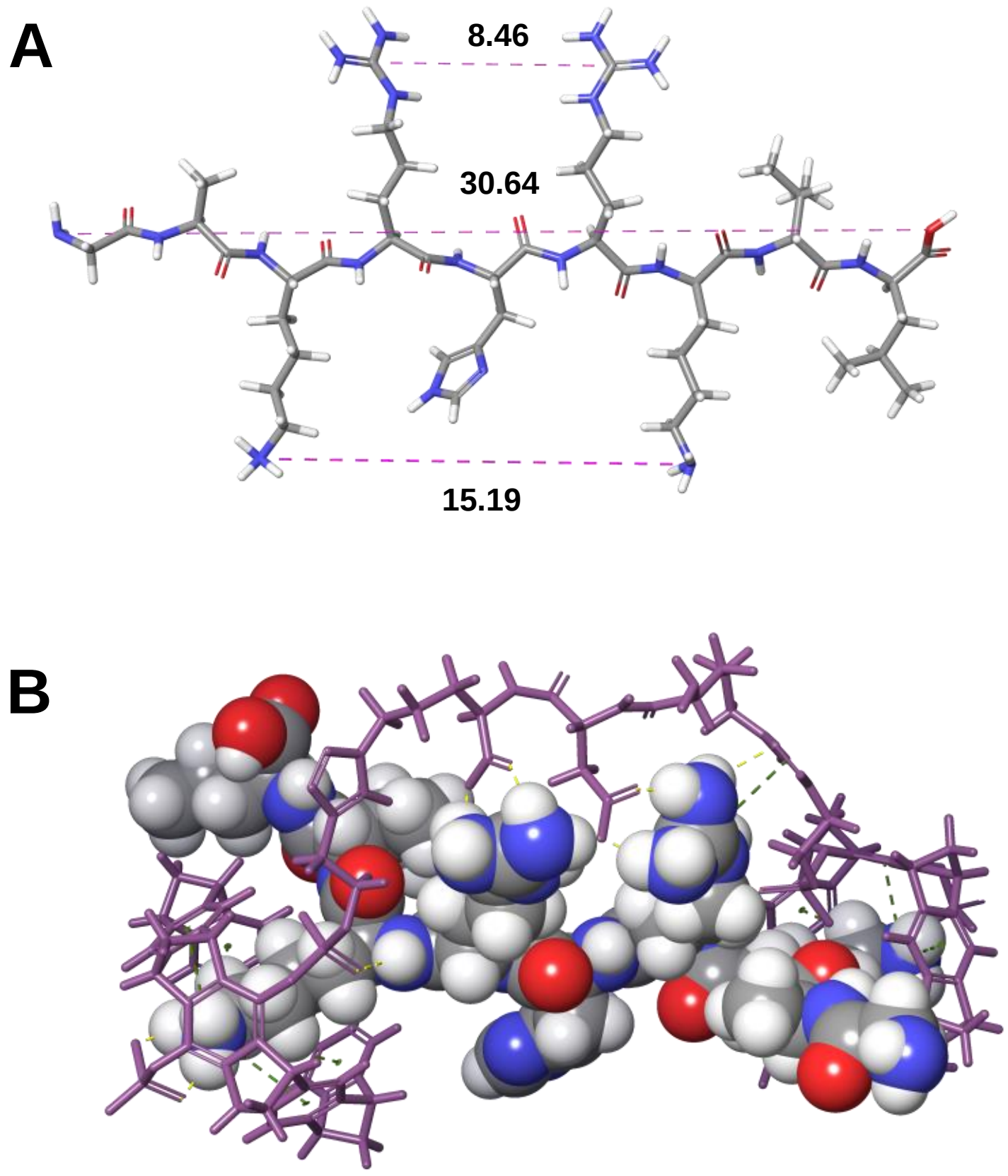

Figure S1: A) Structure of the short peptide stretch H4$_{14\text{-}22}$ as a stick model. The average distance of both lysines (i, i+5) is ~ 1.5 nm (magenta). B) MD simulation of H4$_{14\text{-}22}$ with a dimeric tweezer containing a central aspartate in explicit water at 1.0 bar und 298 K for 100 ns. Both lysine side chains (K16, K20) are threaded through the tweezer cavities while the arginine residues (R17, R19) are complexed by the anionic linker via intramolecular hydrogen bonds (yellow).

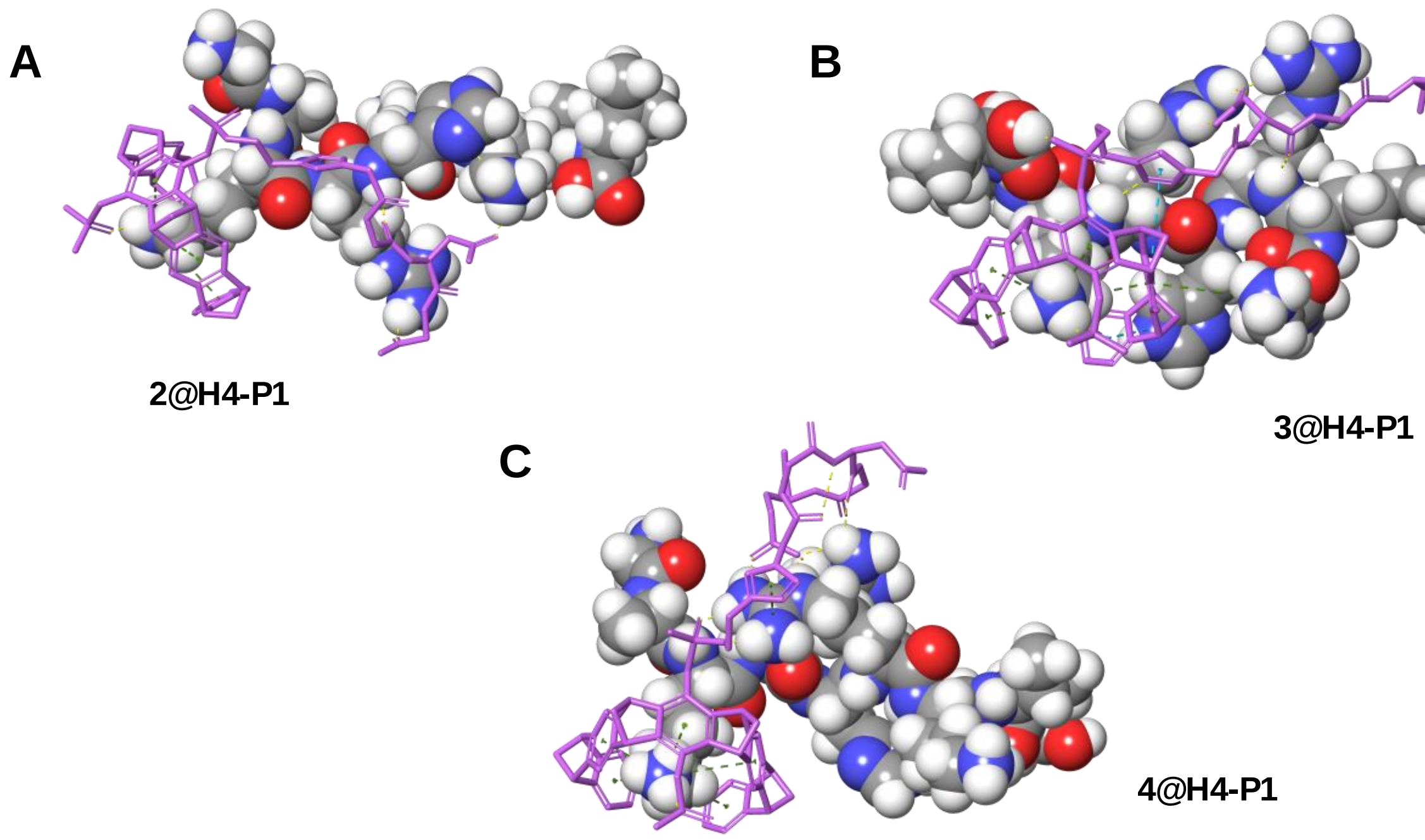

Figure S2. MD simulation of the DNA binding site H4-P1 complexed in a 1:1 stoichiometry by monomeric peptide tweezers **2** (A), **3** (B) and **4** (C). Shown as tweezer/histone peptide (CPK model) complexes. Lysines are included inside the tweezer cavity via π-cation interactions (green). Both arginines of the DNA binding site are recognized by Asp or Glu carboxylates via ion pair-reinforced hydrogen bonds (yellow). The simulations were run at 1.031 bar and 298.25 K for 100 ns.

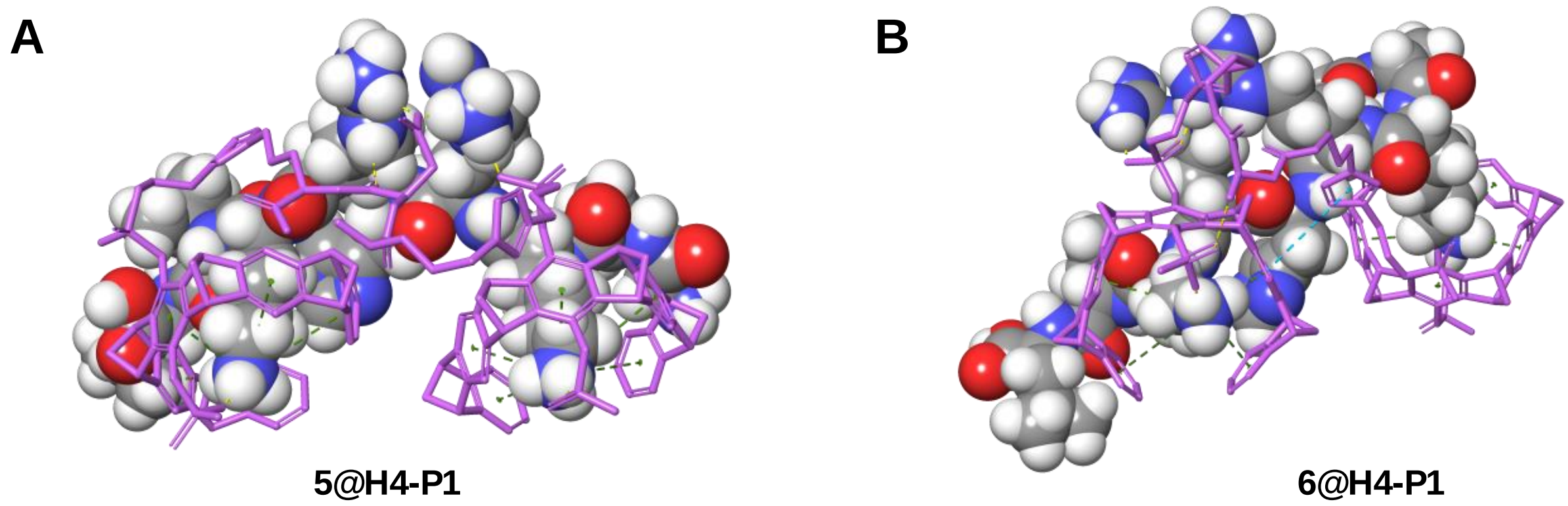

Figure S3. MD simulation of the DNA binding site H4-P1 complexed by the dimeric tweezer **5** (A) and **6** (B) Lysines are included inside the tweezer cavity via π-cation interactions (green). Both arginines of the DNA binding site are recognized by the Asp carboxylate via ion pair-reinforced hydrogen bonds (yellow). The simulations were run at 1.031 bar and 298.25 K for 100 ns.

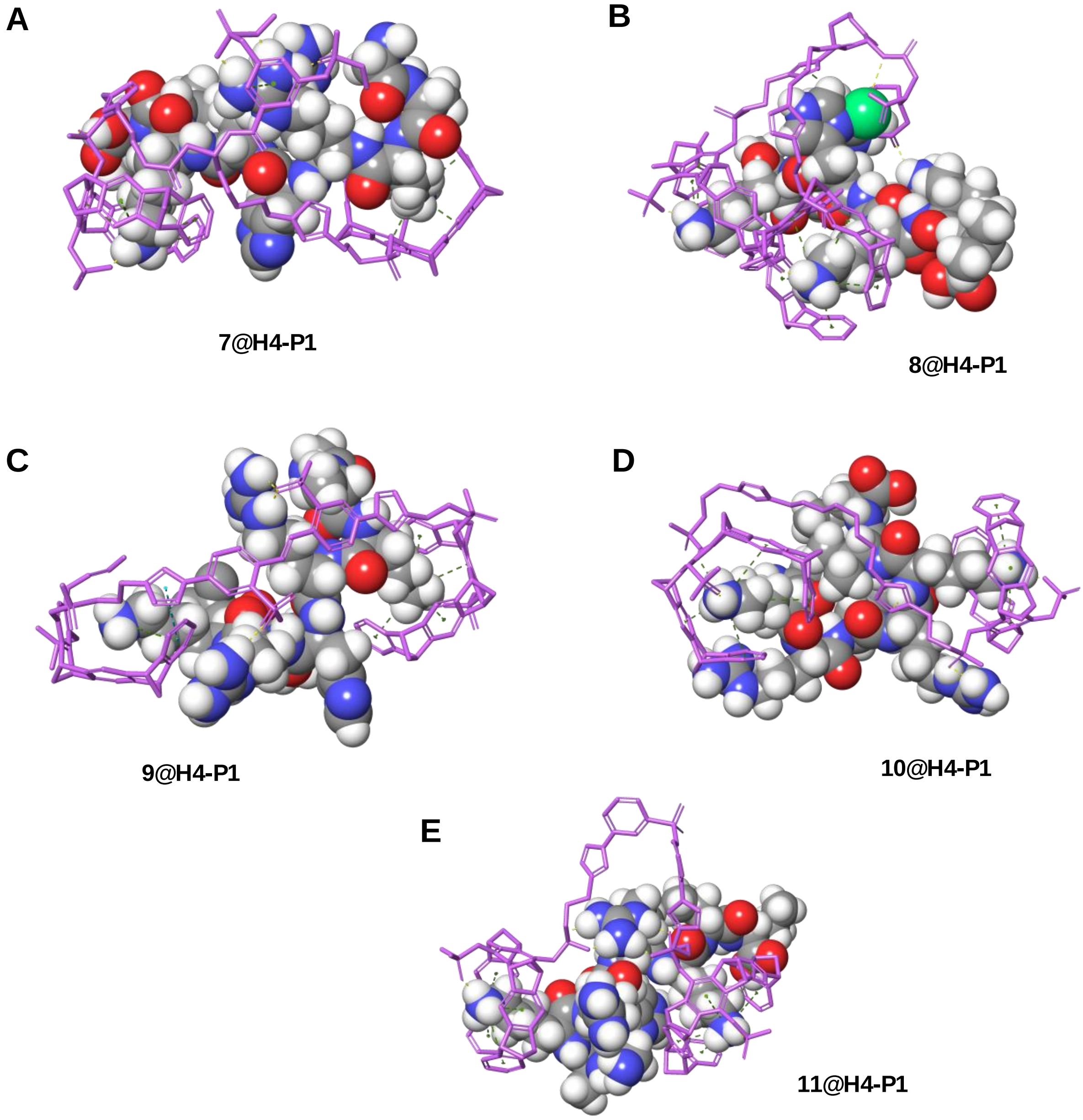

Figure S4. MD simulation of the DNA binding site H4-P1 complexed by the dimeric bisphosphonate tweezer **7** (A), NTA tweezer **8** (B), disulfonate tweezer **9** (C), PEG tweezer **10** (D) and sulfone tweezer **11** (E). Lysines are included inside the tweezer cavity via π-cation interactions (green). Both arginines of the DNA binding site are recognized by the bisphosphonate (**7**) and NTA (**8**) moiety as well as by the disulfonate (**9**) and phosphate groups (**10**/**11**) of the tweezers. A Ni^{2+} cation (light green) forms an octaedric complex with Histidine and the NTA moiety. The simulations were run at 1.031 bar and 298.25 K for 100 ns.

H4-P1 ($H4_{14\text{-}22}$) - DNA binding site

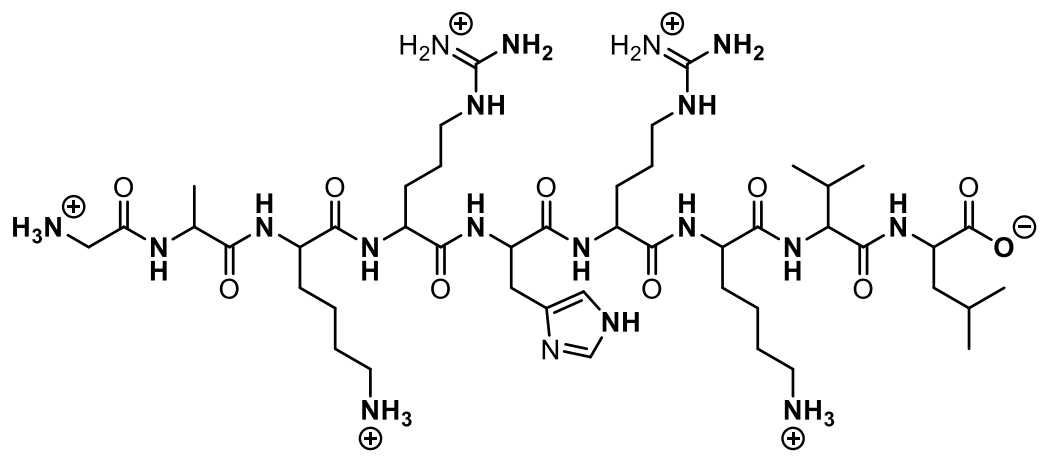

Gly-Ala-**Lys**-**Arg**-His-**Arg**-**Lys**-Val-Leu

H4-P4 ($H4_{6\text{-}14}$)

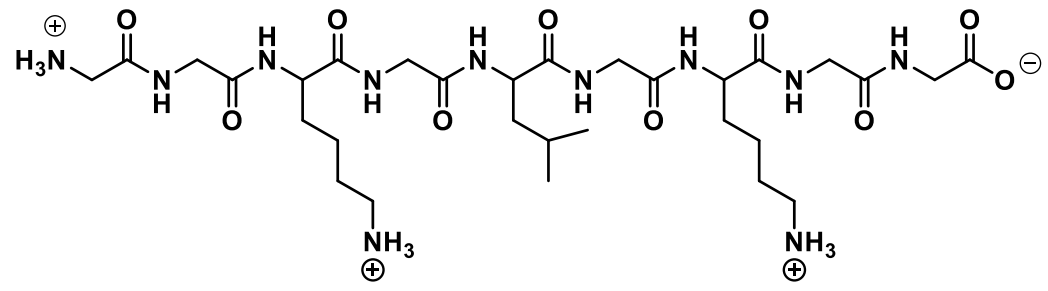

Gly-Gly-**Lys**-Gly-Leu-Gly-**Lys**-Gly-Gly

H4-Full-Length ($H4_{1\text{-}22}$)

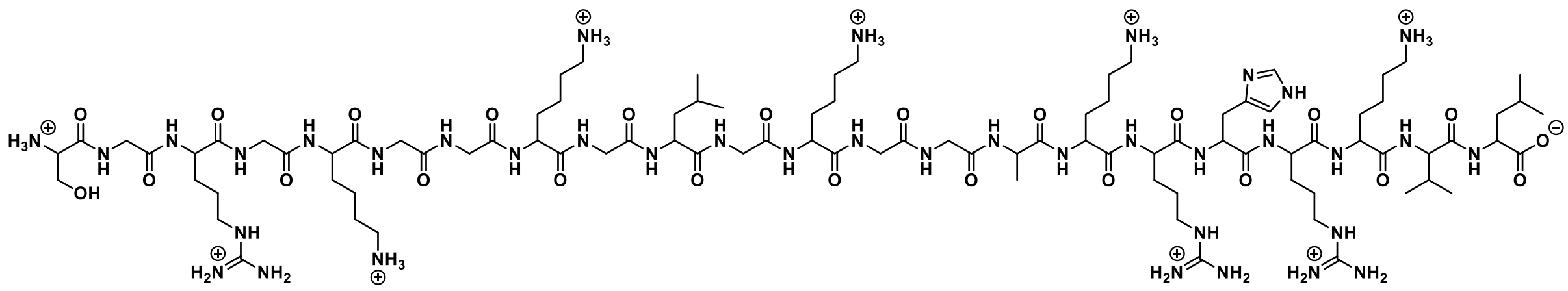

Ser-Gly-**Arg**-Gly-**Lys**-Gly-Gly-**Lys**-Gly-Leu-Gly-**Lys**-Gly-Gly-Ala-**Lys**-**Arg**-His-**Arg**-**Lys**-Val-Leu

Figure S5A: Structures of the H4 peptide fragments H4-P1 ($H4_{14\text{-}22}$), H4-P4 ($H4_{6\text{-}14}$) and H4-Full-Length ($H4_{1\text{-}22}$) used in titration experiments.

Fluorescence Anisotropy

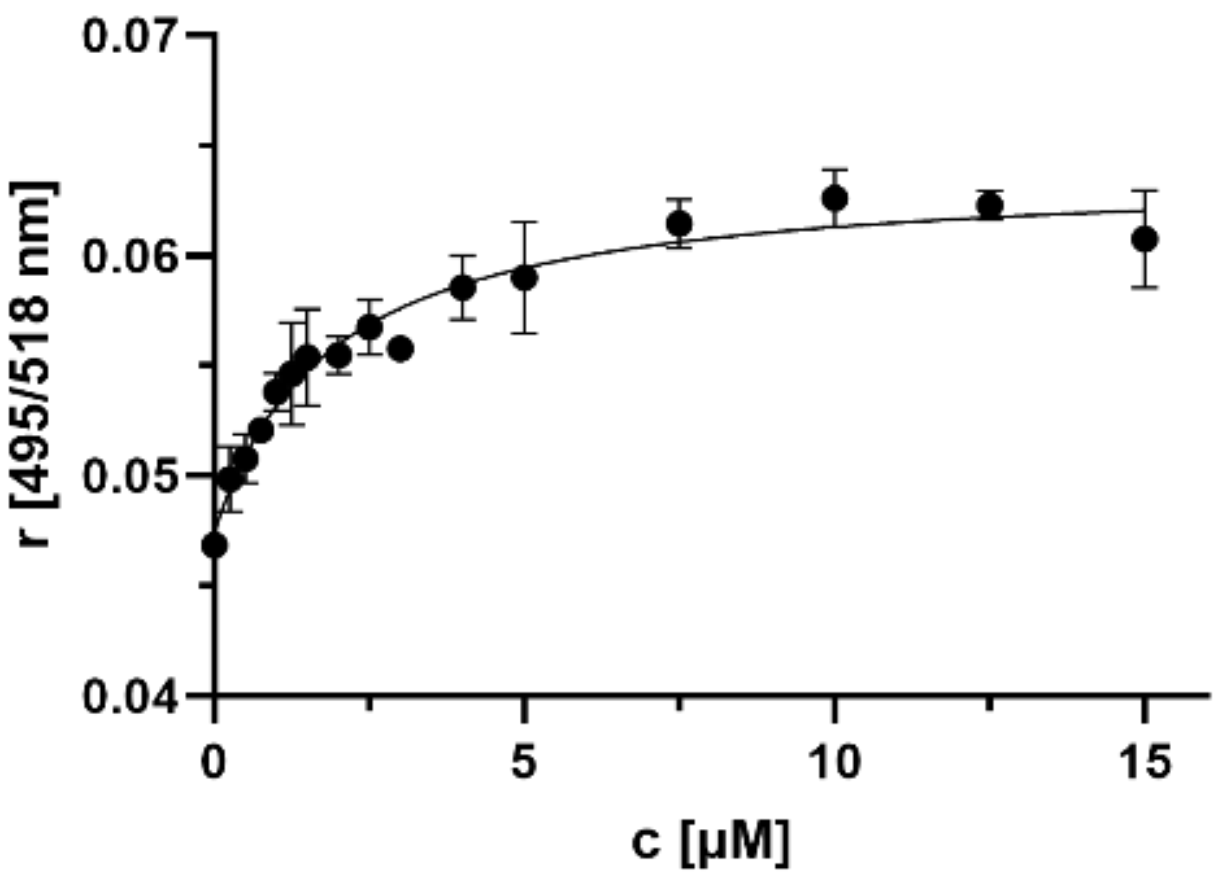

Figure S5B: *Anisotropy measurements of histone H4 and DNA.* DNA was added stepwise up to 15 µM to 0.1 µM $H4_{7\text{-}22}$-5,6-FAM. A K_D value of 1.8 µM was obtained from nonlinear curve-fitting (R^2 = 0.91; one-site binding model; saturation binding).

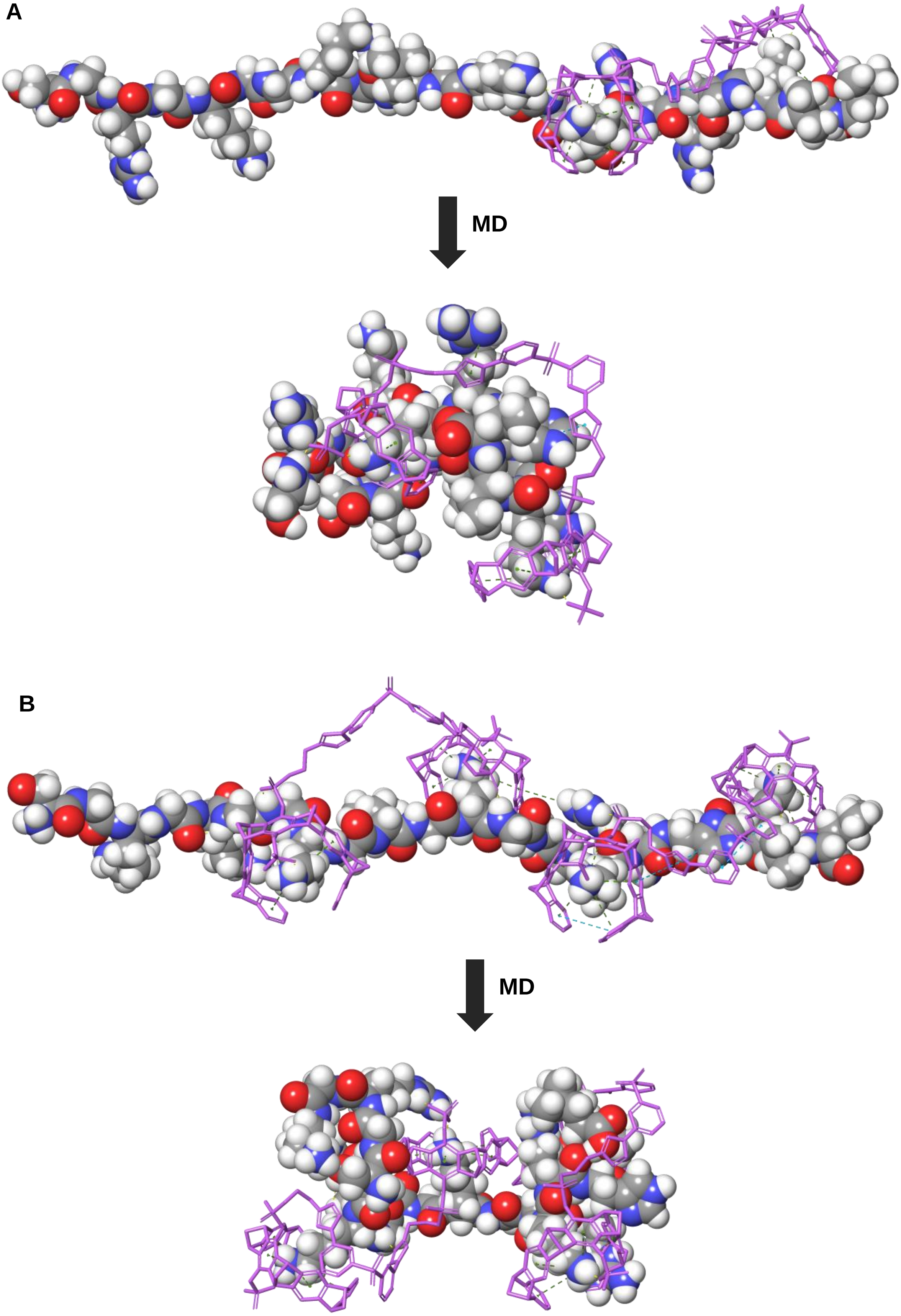

Figure S6: MD simulation of a 1:1 (A) and a 1:2 (B) complex between **11** and H4-Full-Length in discrete water at 1.0 bar and 300 K for 100 ns. Top: side view of the complex before running the simulations; below: front view after running the simulations.

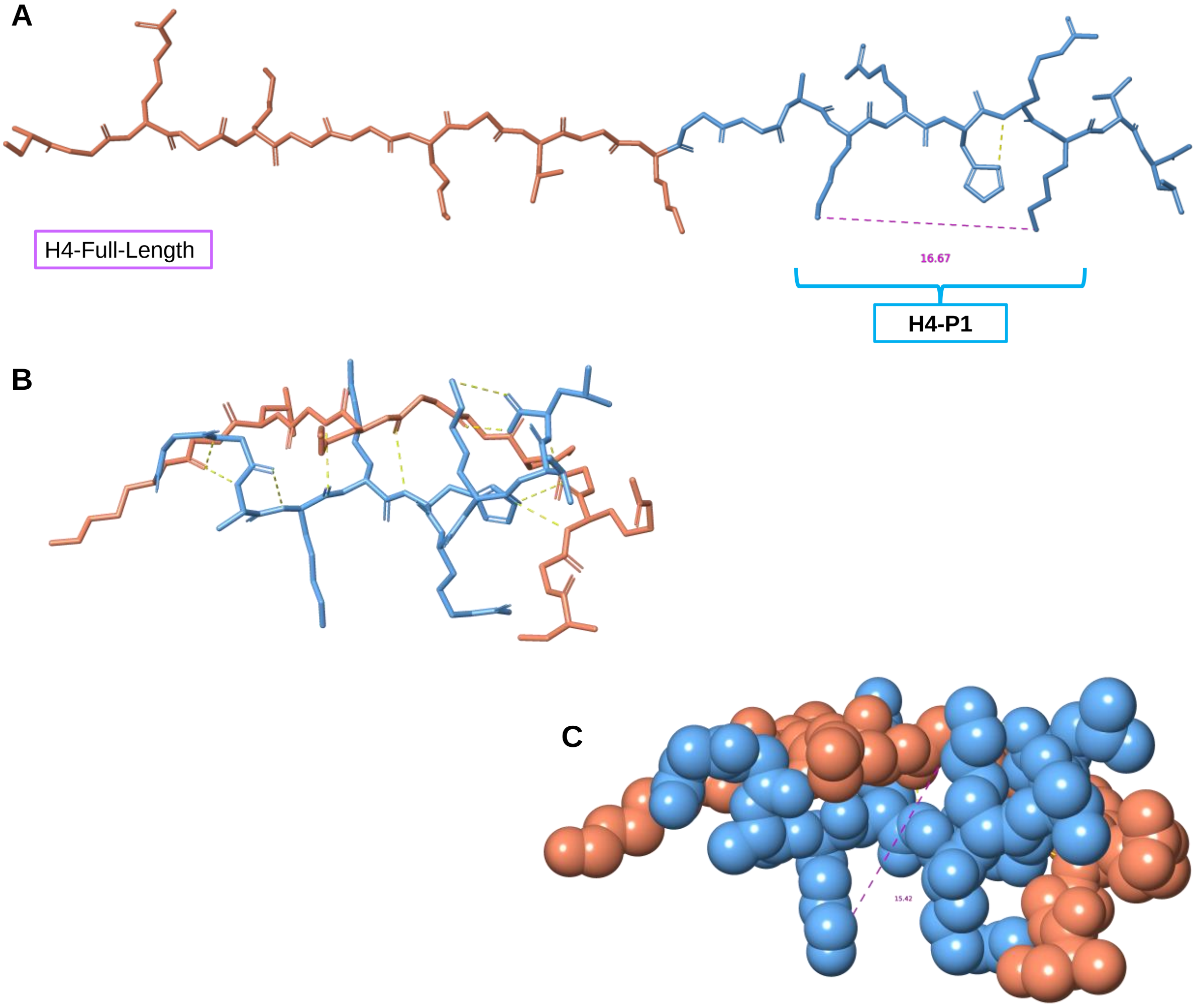

Figure S7: A) Molecular Modeling of H4-Full-Length in its extended conformation. In the DNA binding site (blue) the distance between both lysines (i, i+5) was determined at 1.7 nM. B) MD simulation of the H4-Full-Length Peptide in a solvent box of discrete water molecules at 1.0 bar and 300 K for 100 ns. (B) Stick model; (C) CPK model. The lysine (i, i+5) distance is determined at 1.5 nm.

ITC titrations of new tweezer derivatives with H4 peptides:

ITC titrations of advanced tweezers with H4-P1

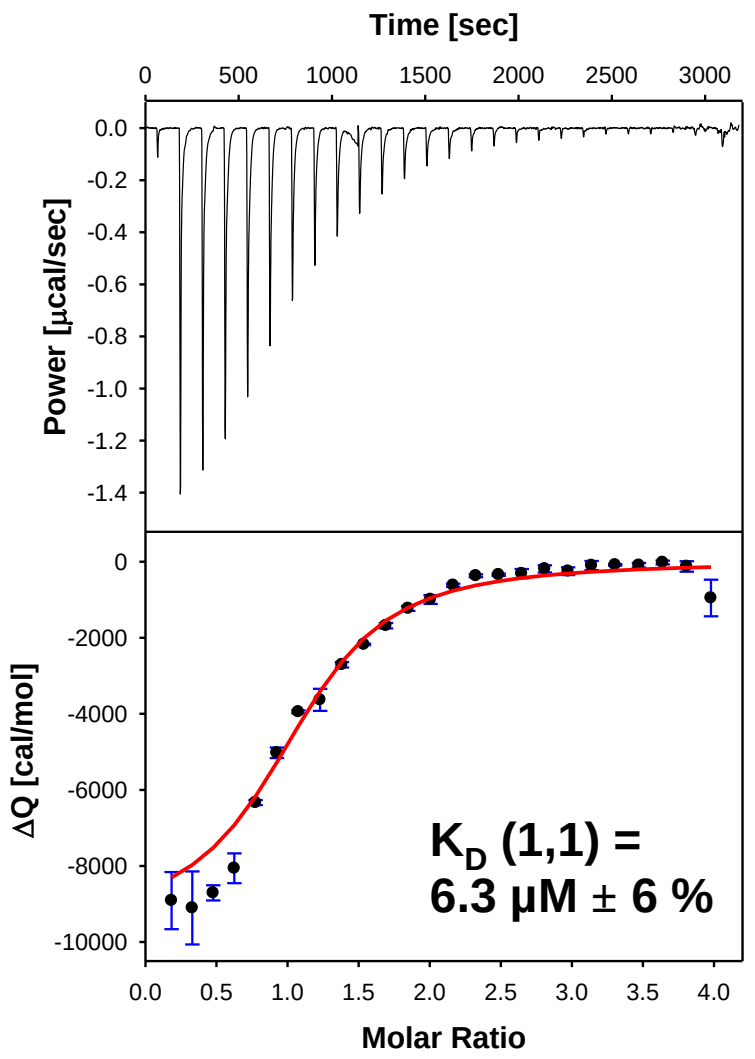

1 mM **CLR01** To 50 µM **H4-P1**

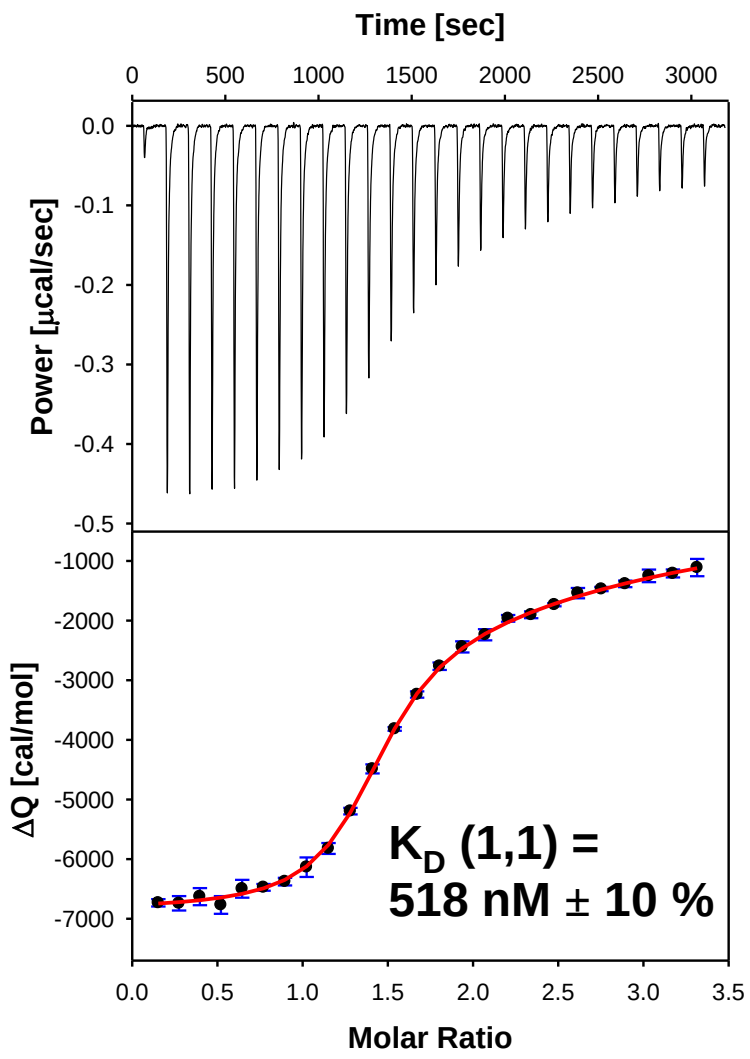

500 µM **2** To 30 µM **H4-P1**

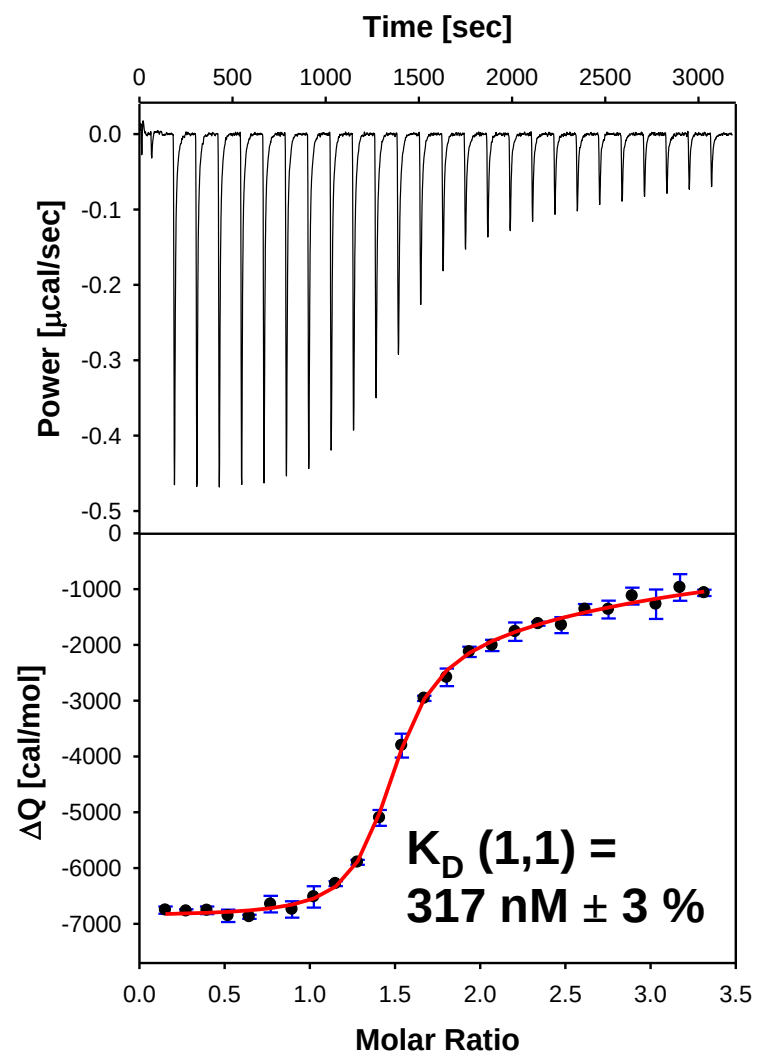

500 µM **3** To 30 µM **H4-P1**

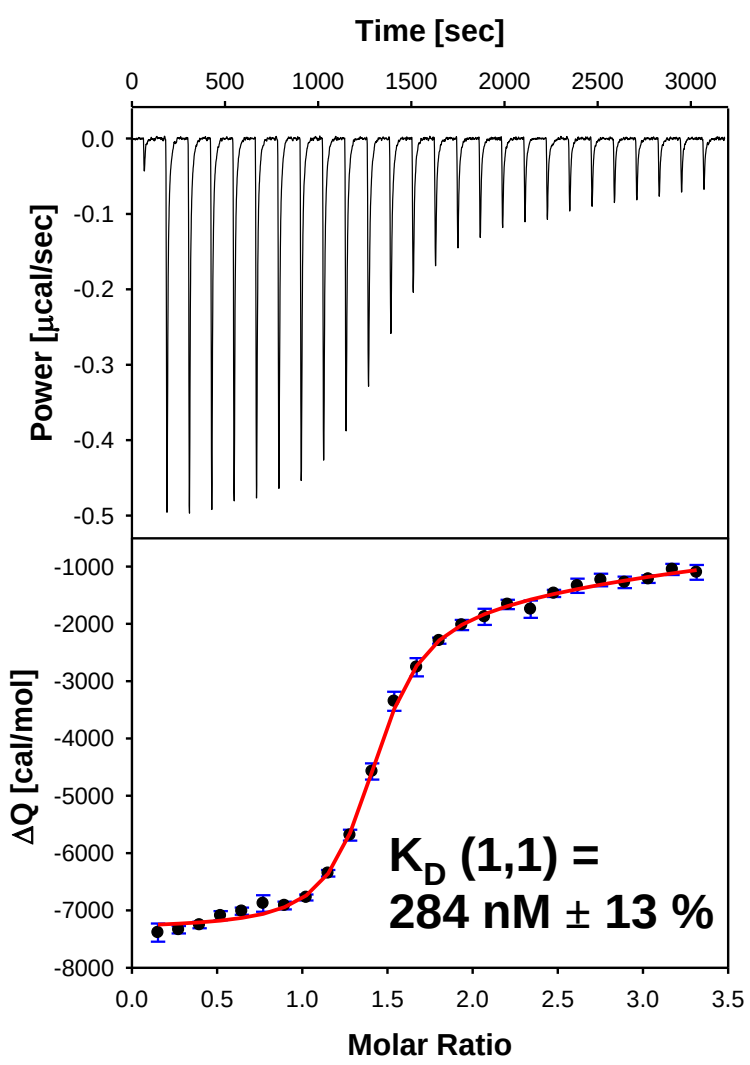

500 µM **4** To 30 µM **H4-P1**

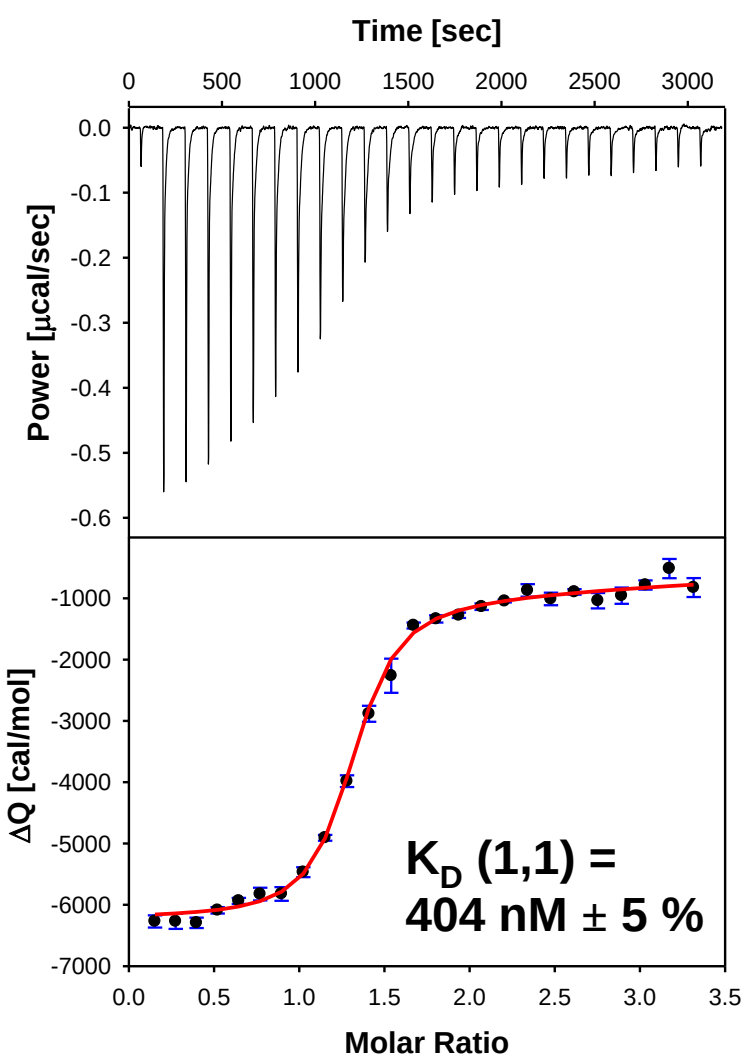

500 µM **5** To 30 µM **H4-P1**

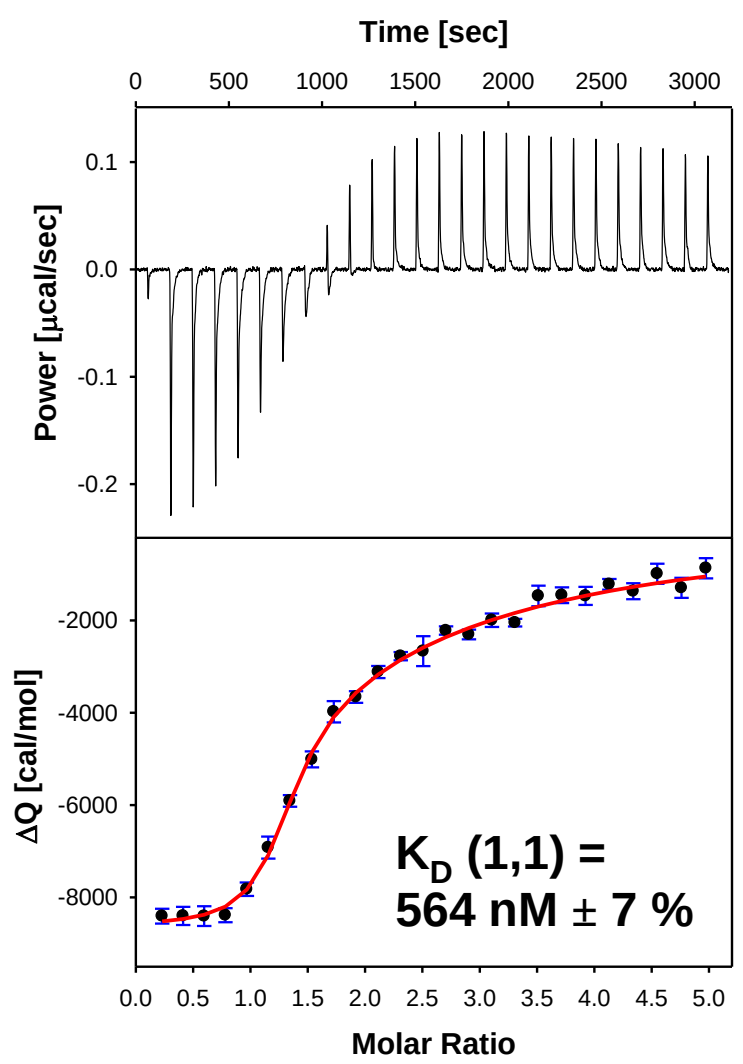

500 µM **6** To 20 µM **H4-P1**

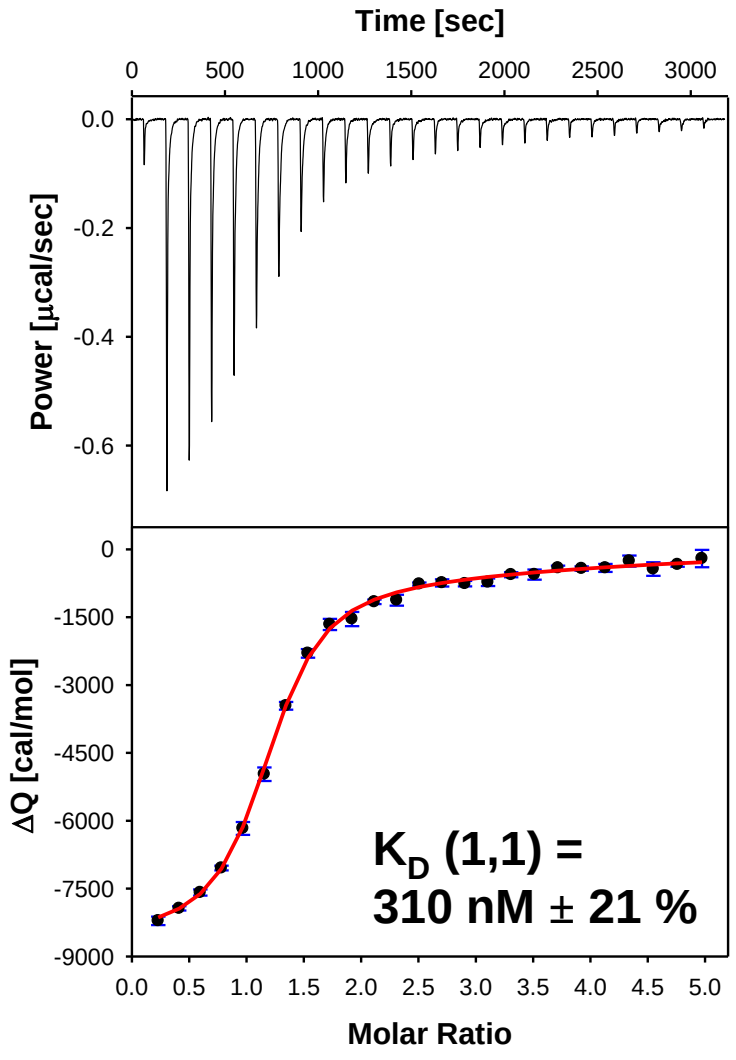

500 µM **7** To 20 µM **H4-P1**

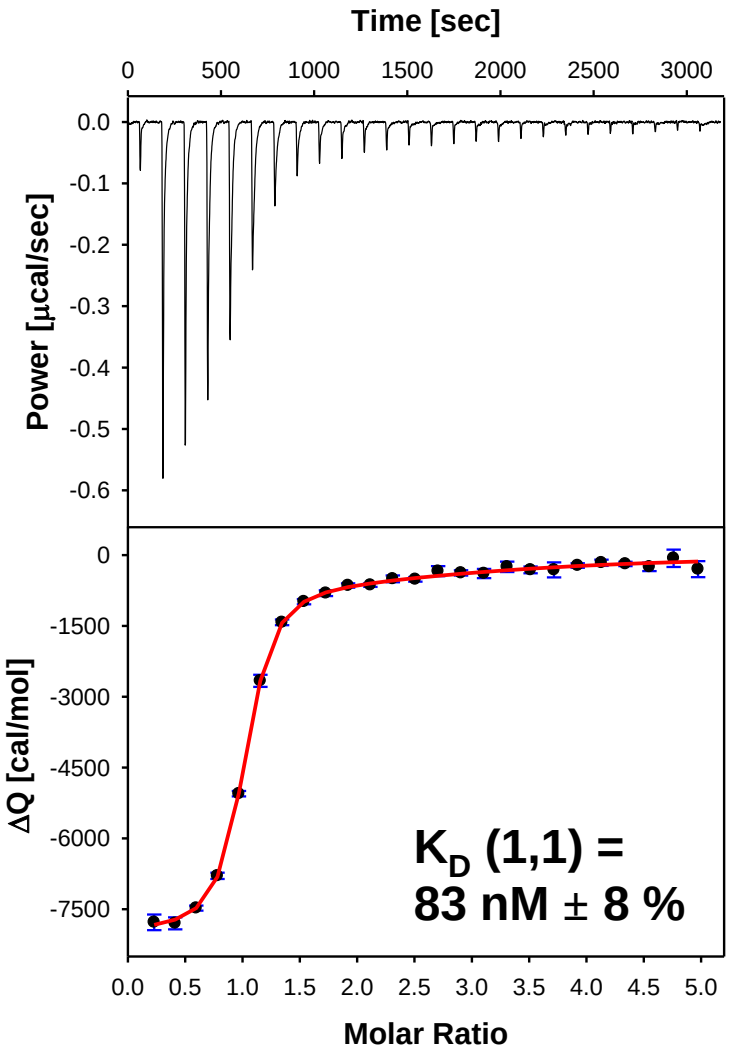

500 µM **8** To 20 µM **H4-P1**

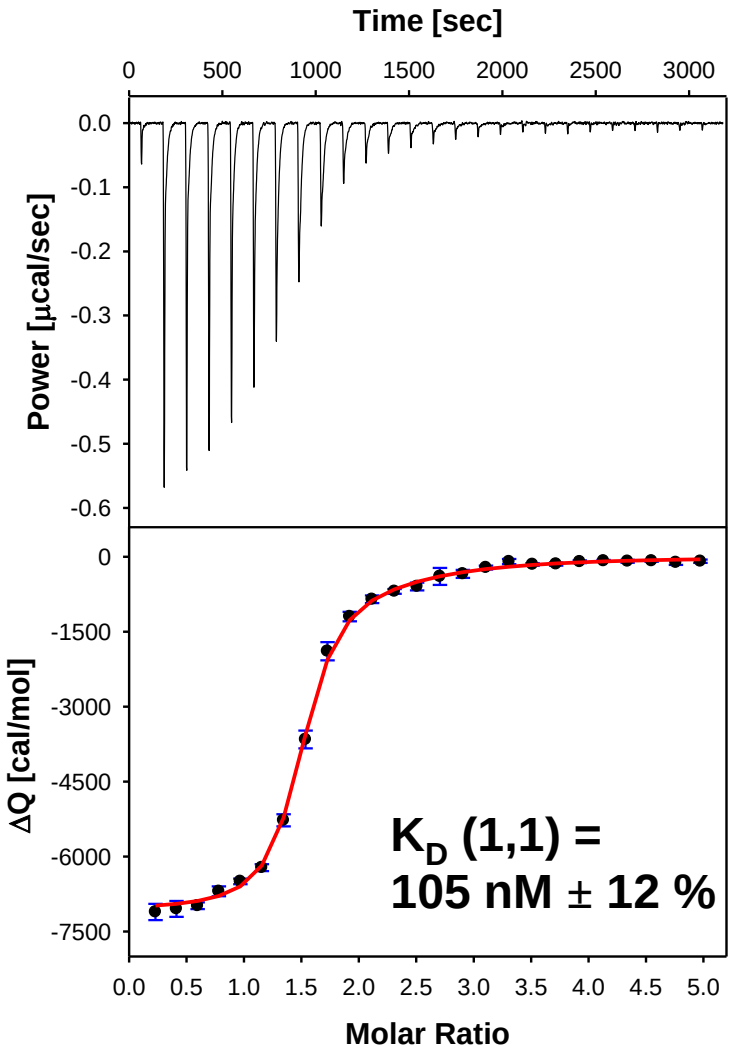

500 µM **9** To 20 µM **H4-P1**

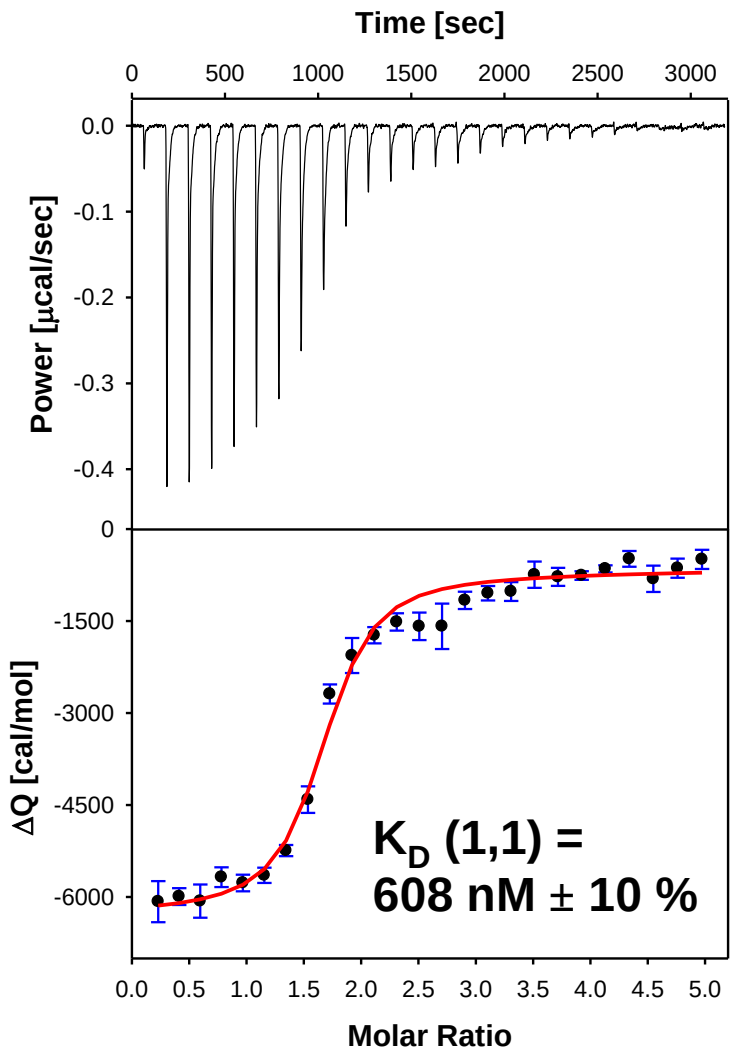

500 µM **10** To 20 µM **H4-P1**

Figure S8: ITC titrations of 1 mM **CLR01** or 500 µM advanced tweezers to 50 µM or 30 µM **H4-P1** in 10 mM KPi buffer (pH 7.0) evaluated according to a simple 1SnS-model. K_D (1,1): curve fitting for 1:1 complex.

ITC titrations of advanced tweezers with H4-P4

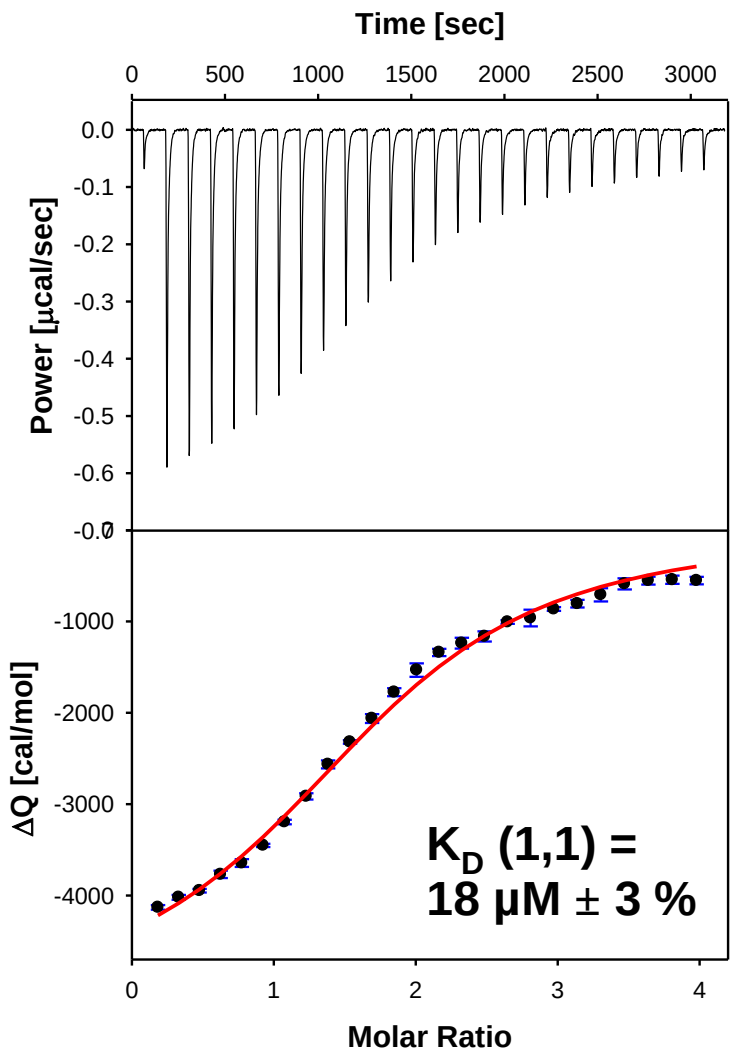

1 mM **CLR01** To 50 µM **H4-P4**

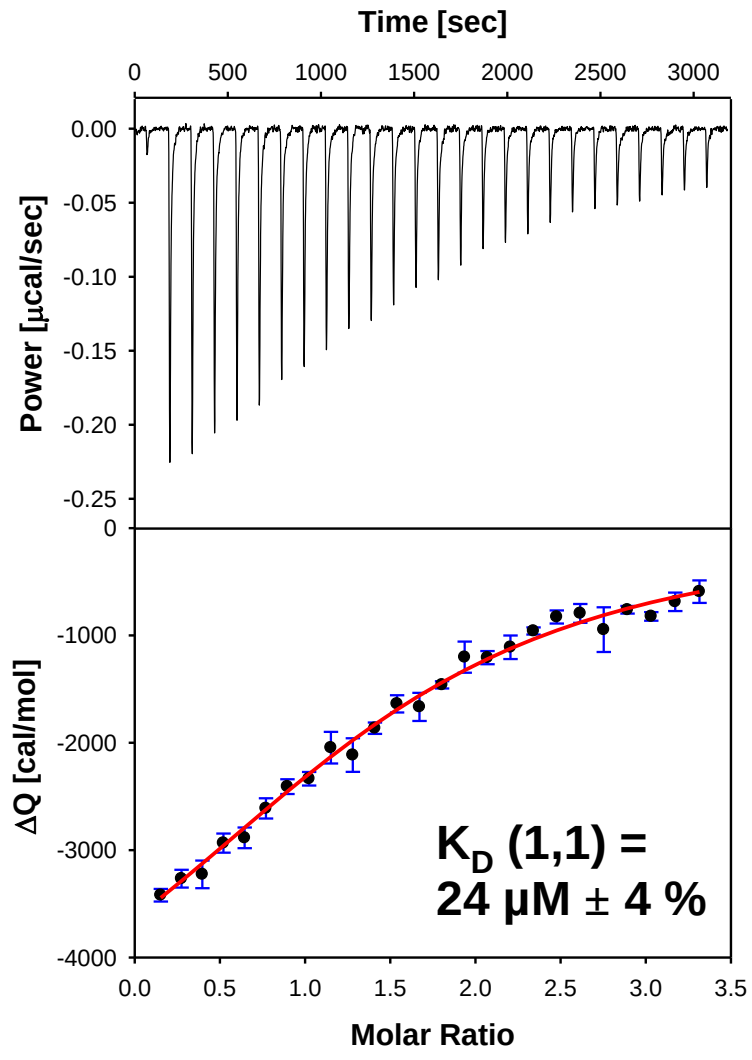

500 µM **2** To 30 µM **H4-P4**

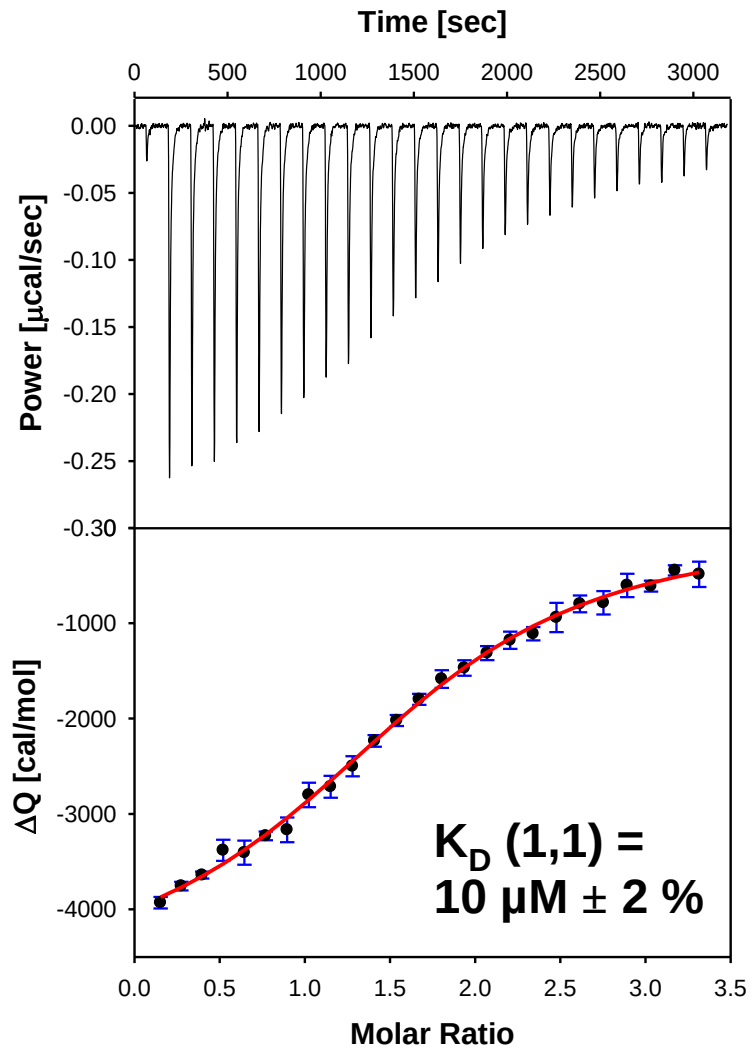

500 µM **3** To 30 µM **H4-P4**

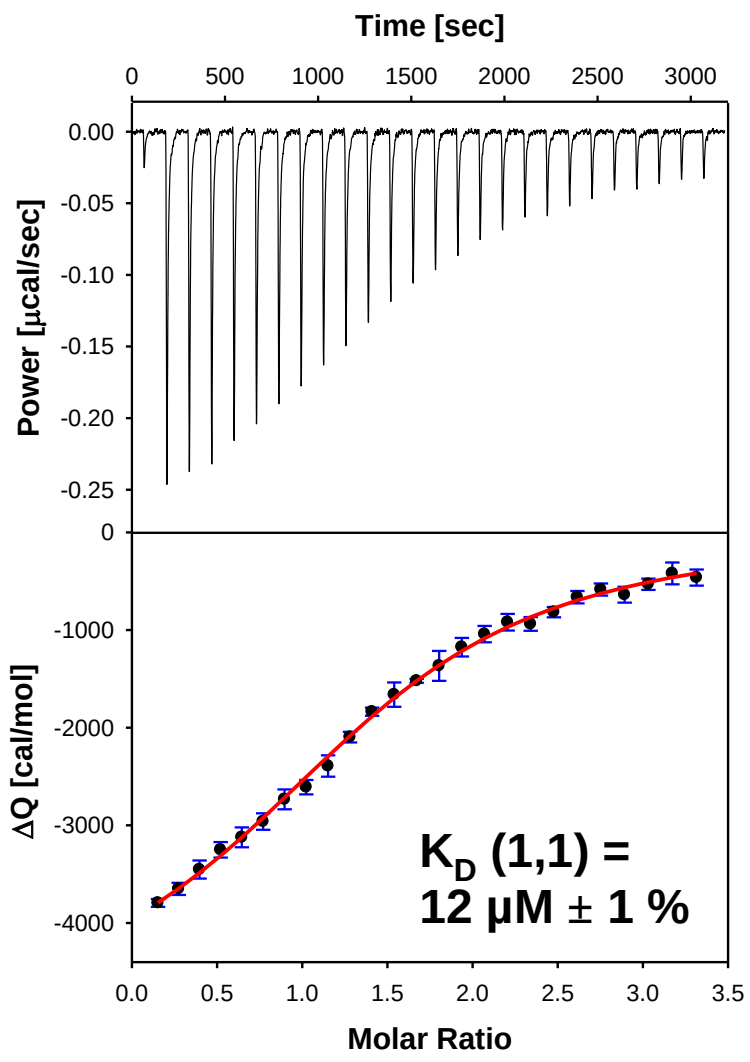

500 µM **4** To 30 µM **H4-P4**

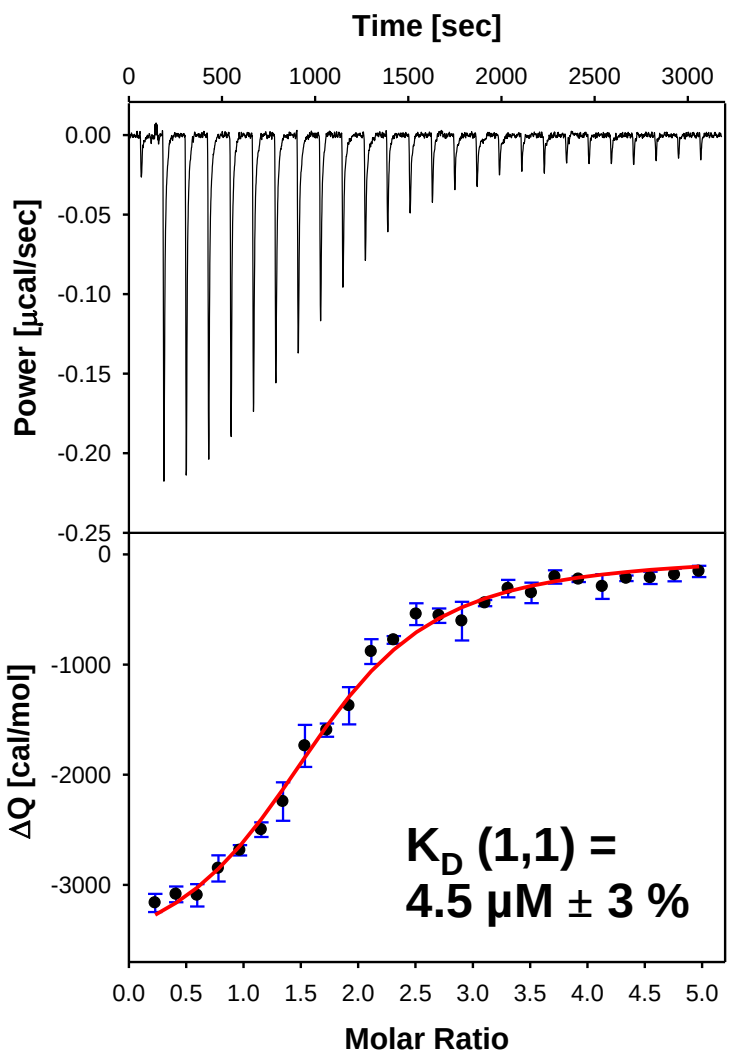

500 µM **5** To 20 µM **H4-P4**

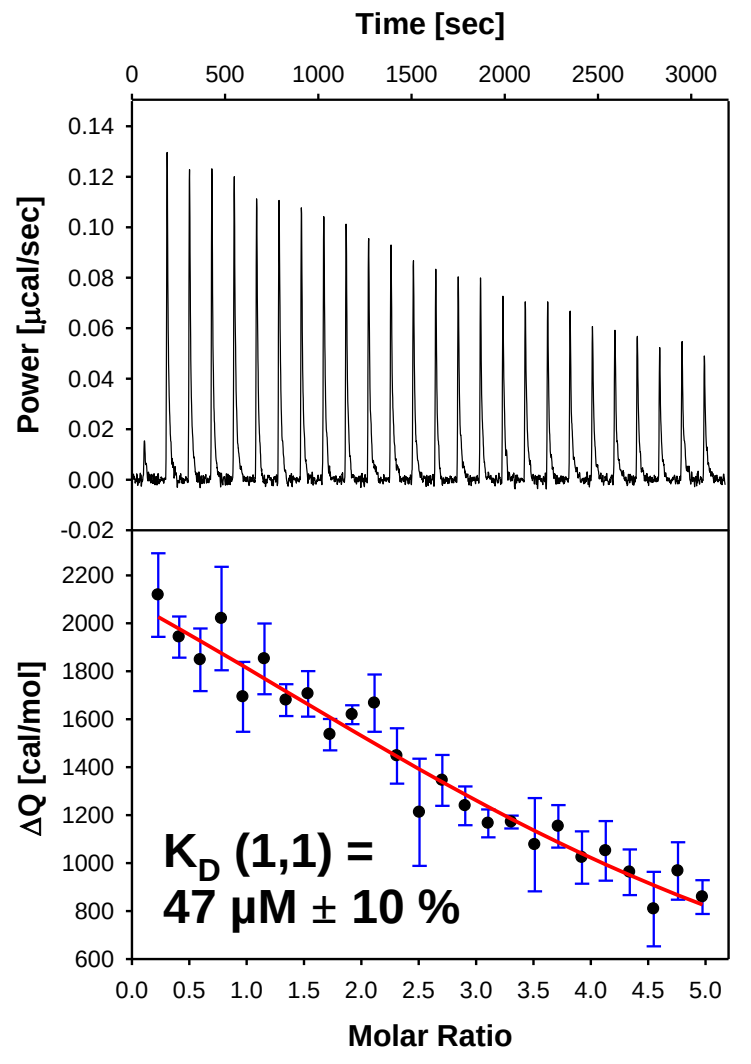

500 µM **6** To 20 µM **H4-P4**

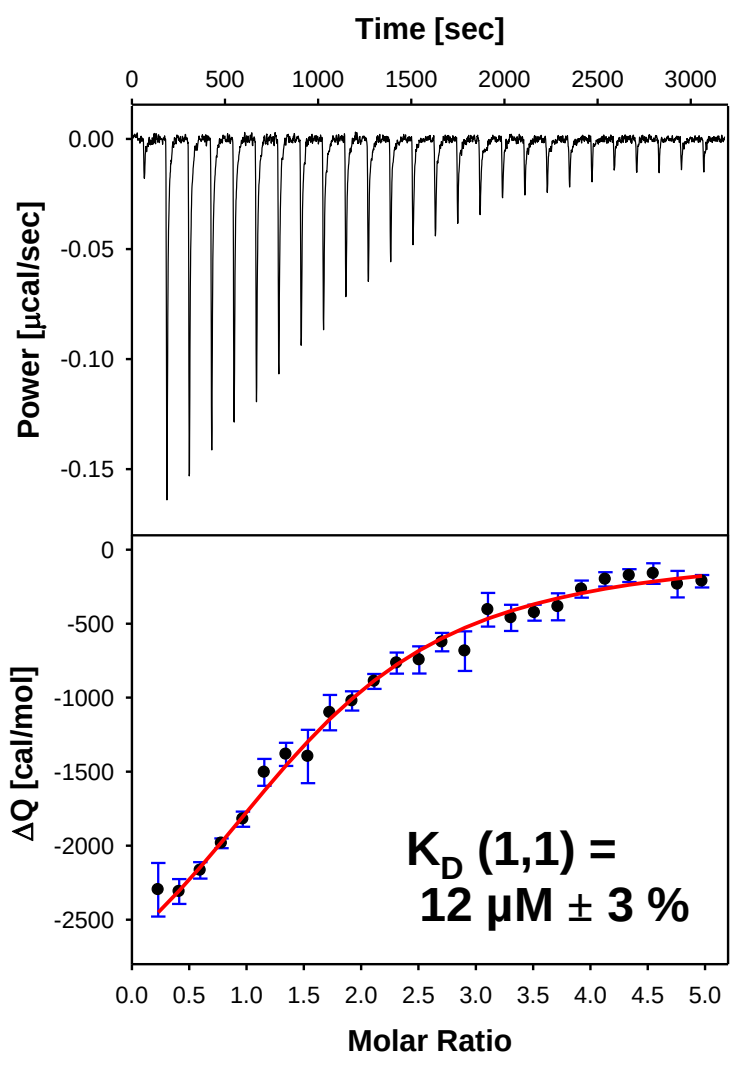

500 µM **7** To 20 µM **H4-P4**

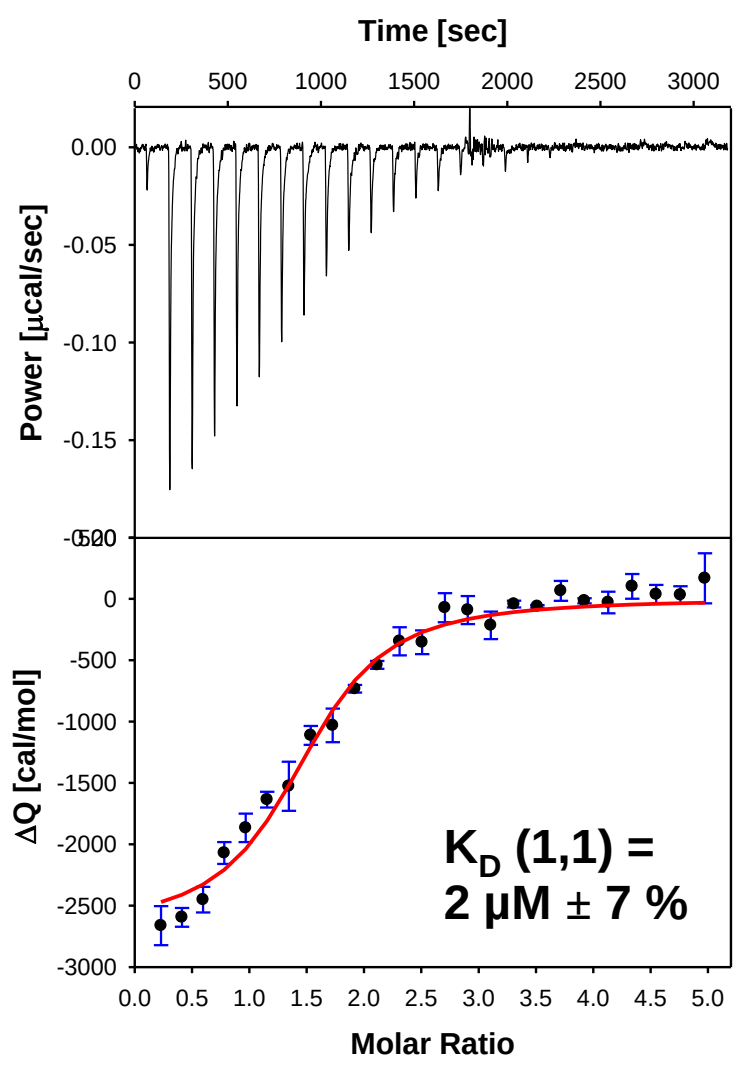

500 µM **8** To 20 µM **H4-P4**

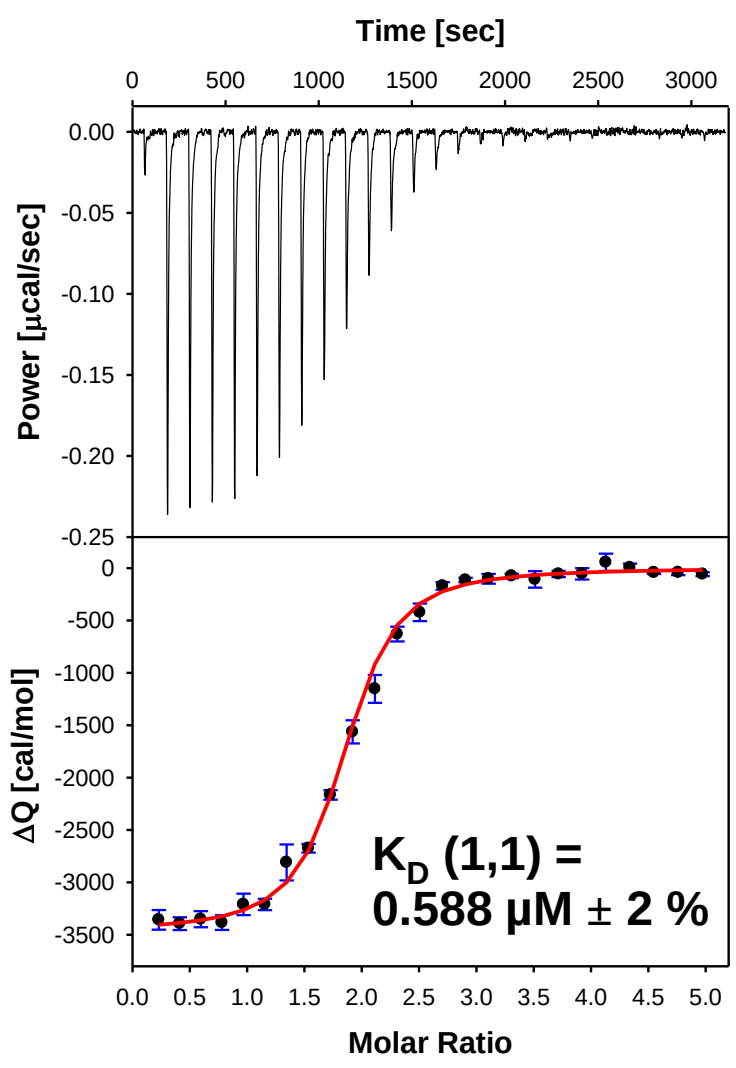

500 µM **9** To 20 µM **H4-P4**

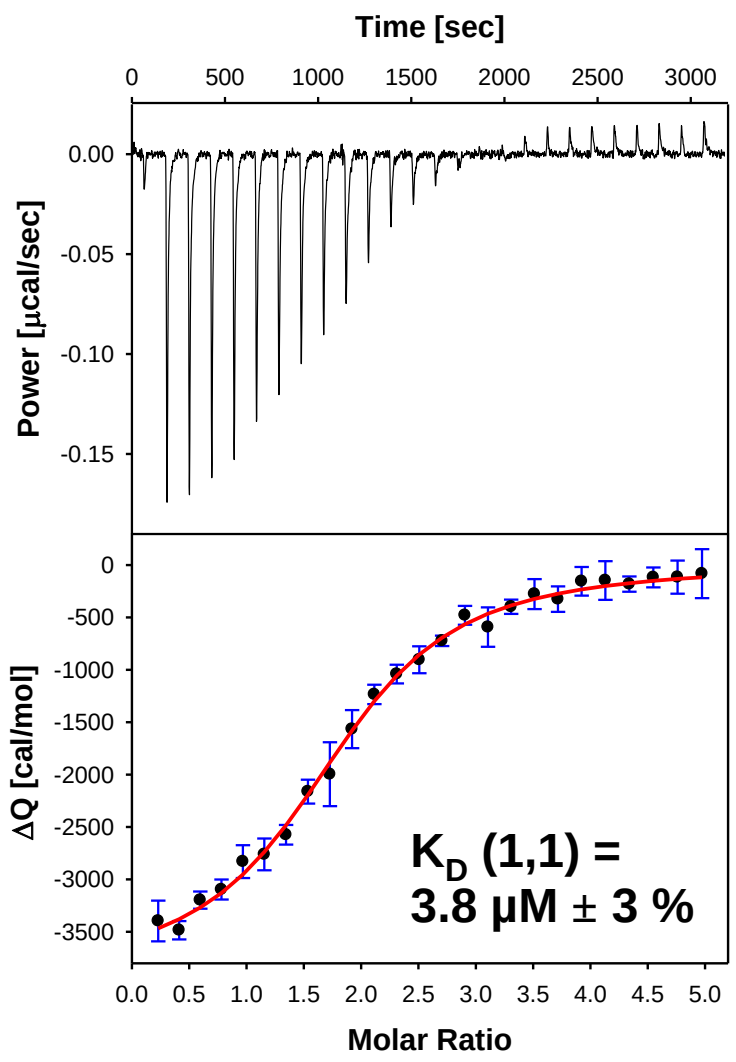

500 µM **10** To 20 µM **H4-P4**

Figure S9: ITC titrations of 1 mM **CLR01** or 500 µM advanced tweezers to 50 uM or 30 µM **H4-P4** in 10 mM KPi buffer (pH 7.0) evaluated according to a simple 1SnS-model. K_D (1,1): curve fitting for 1:1 complex.

ITC titrations of advanced tweezers with H4-Full-Length

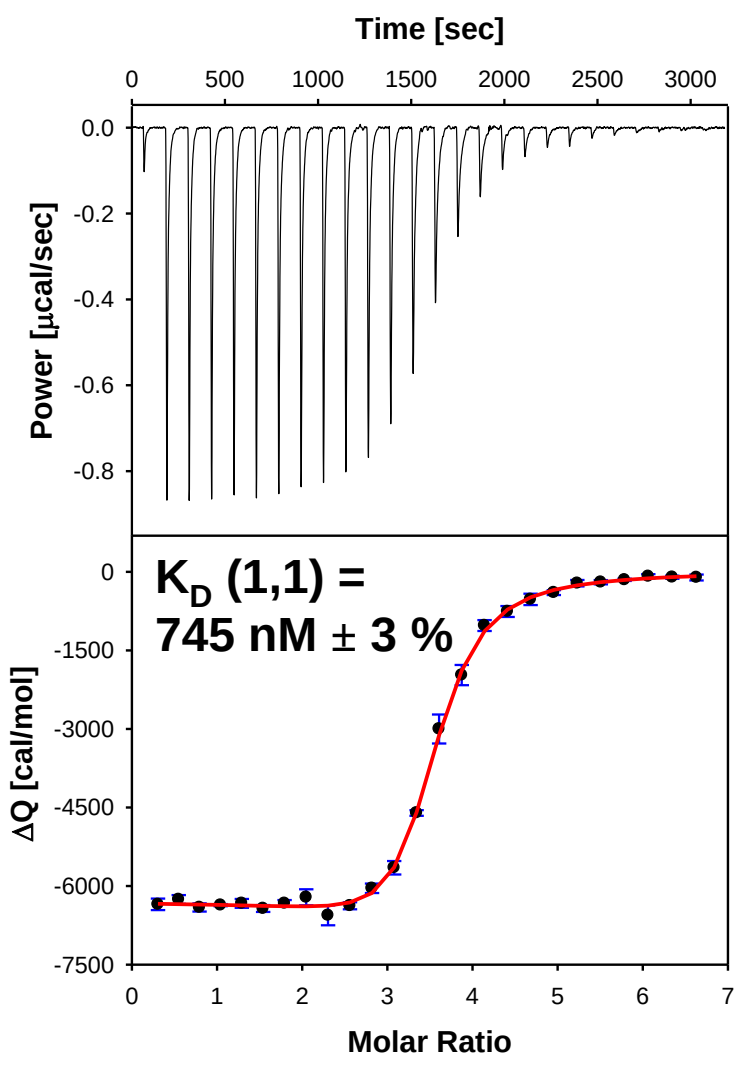

1 mM **CLR01** To 30 µM **H4-Full**

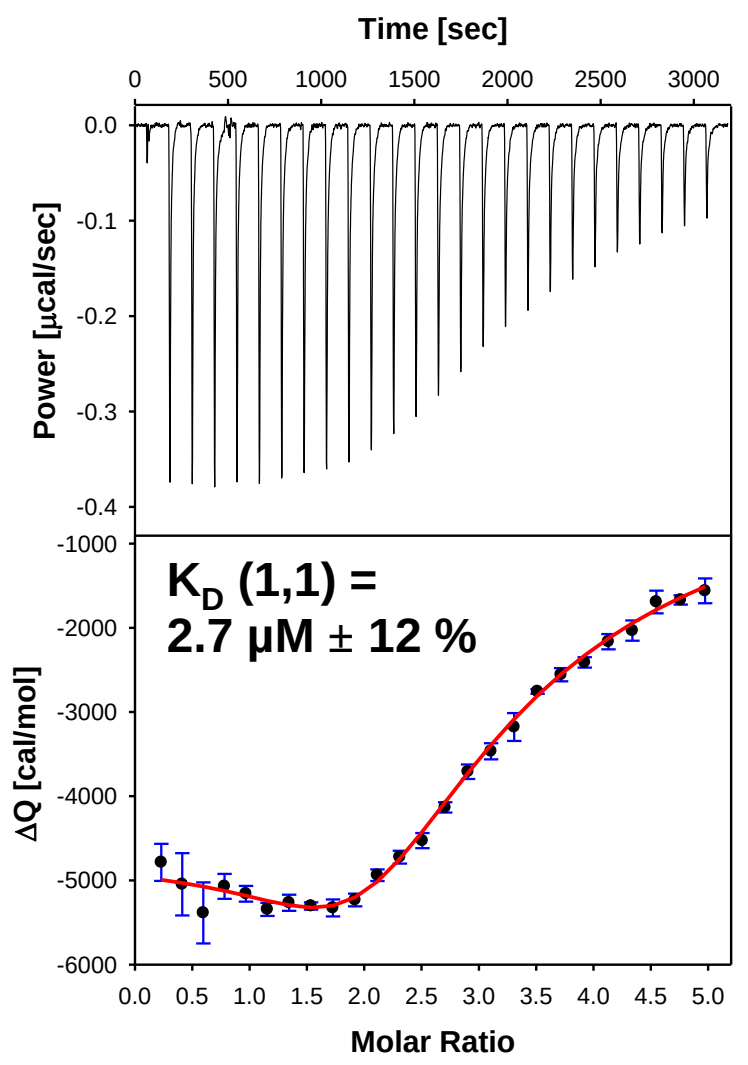

500 µM **2** To 20 µM **H4-Full**

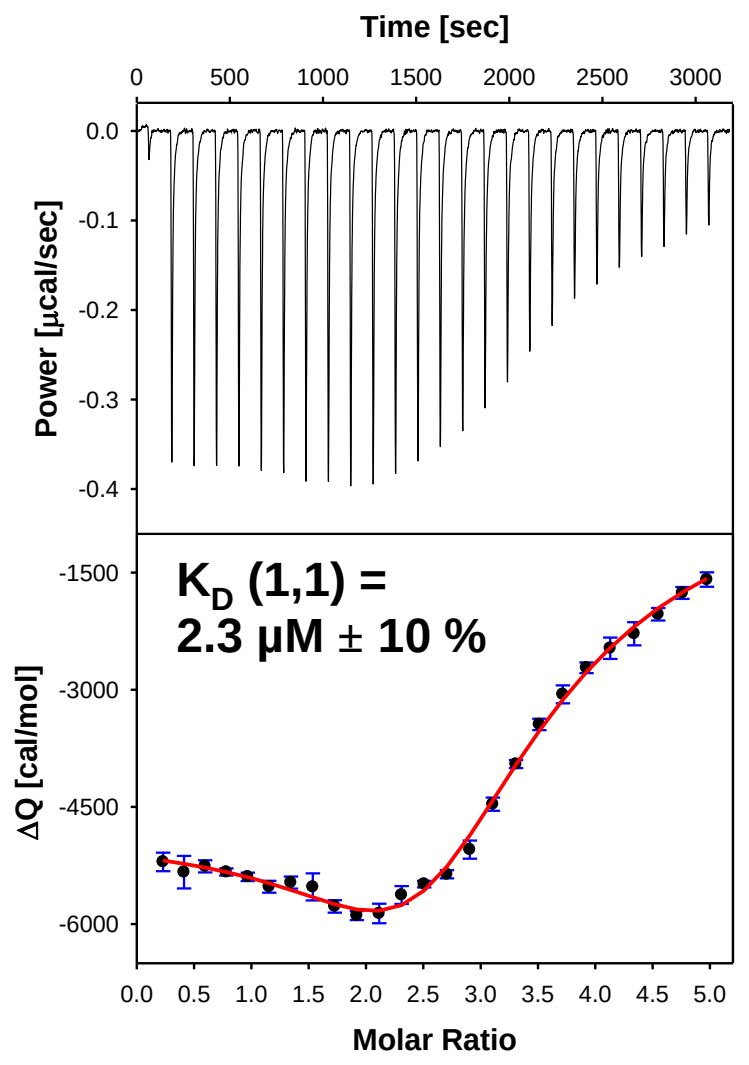

500 µM **3** To 20 µM **H4-Full**

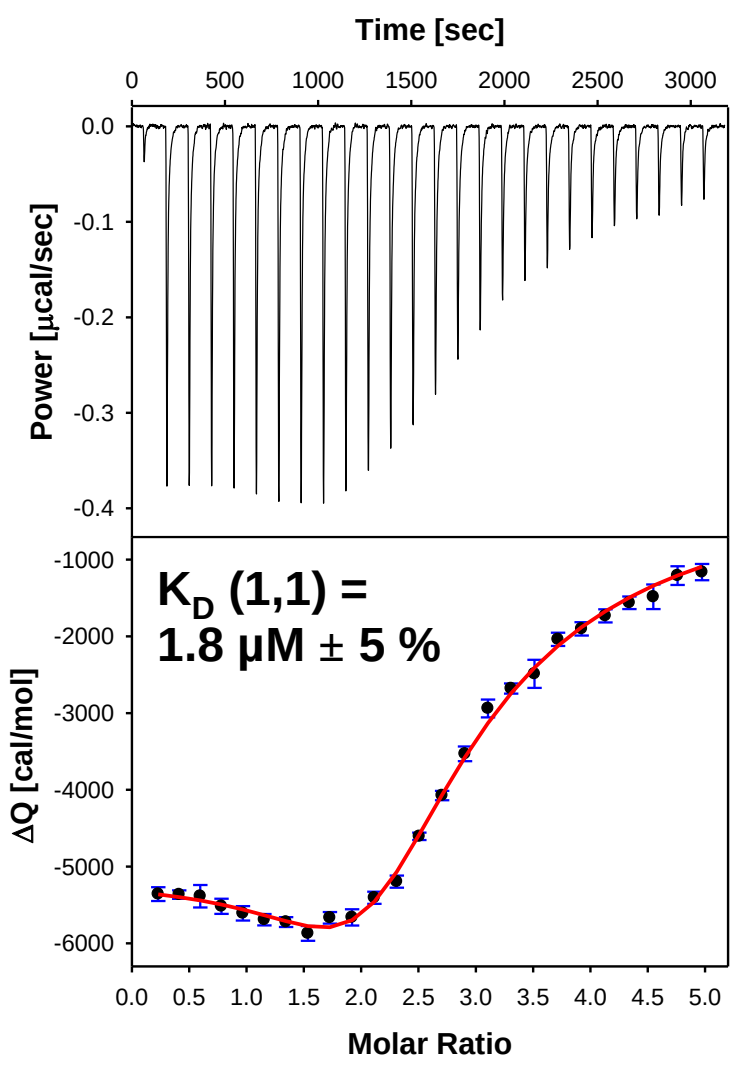

500 µM **4** To 20 µM **H4-Full**

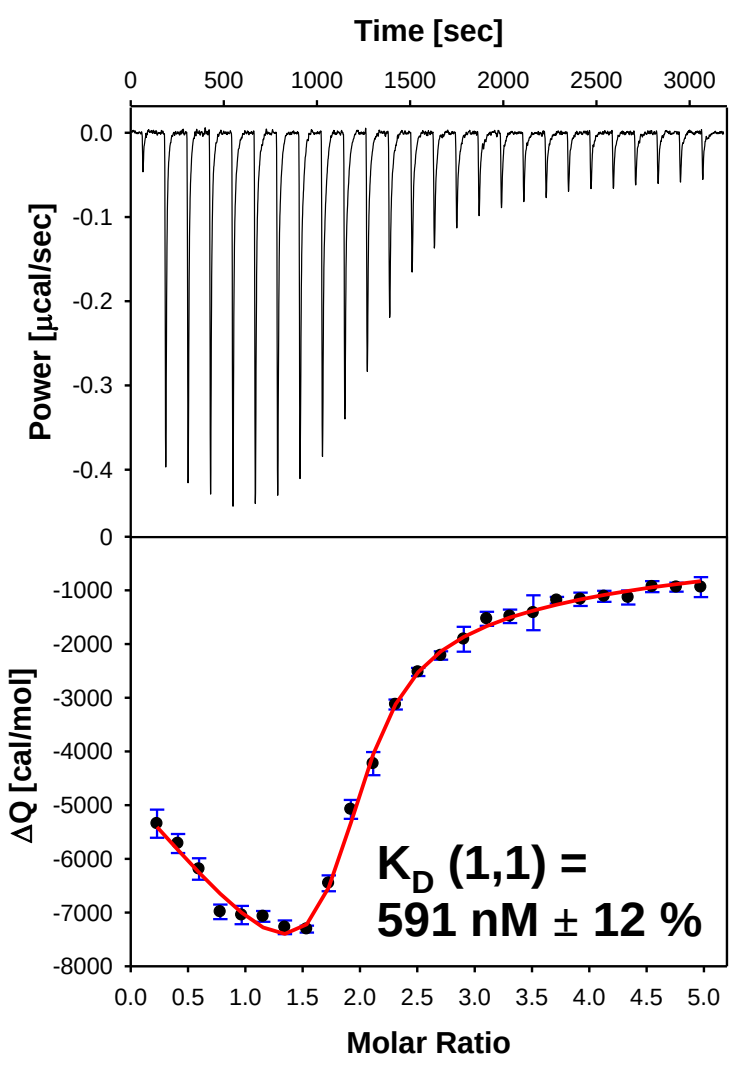

500 µM **5** To 20 µM **H4-Full**

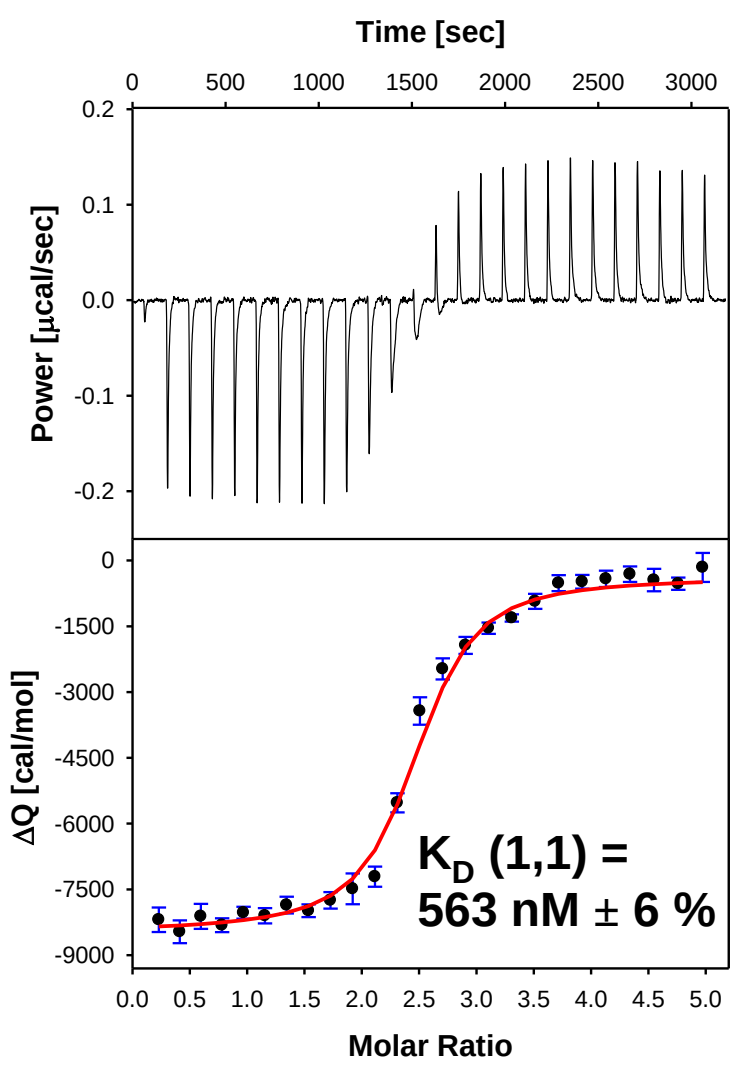

500 µM **6** To 20 µM **H4-Full**

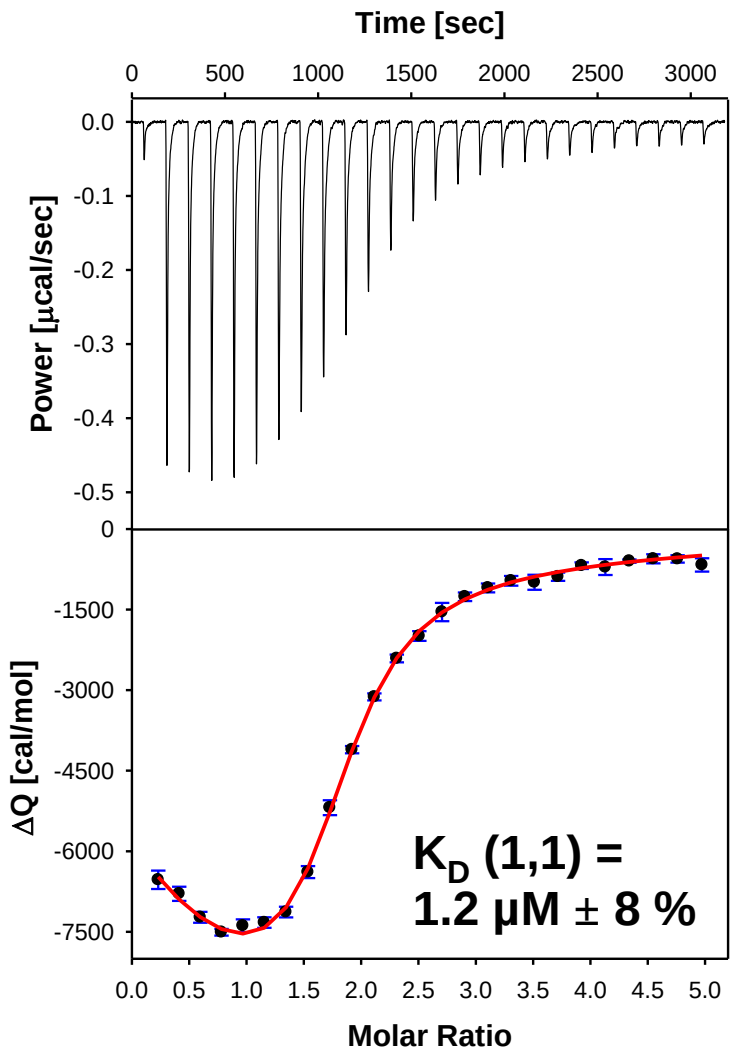

500 µM **7** To 20 µM H4-Full

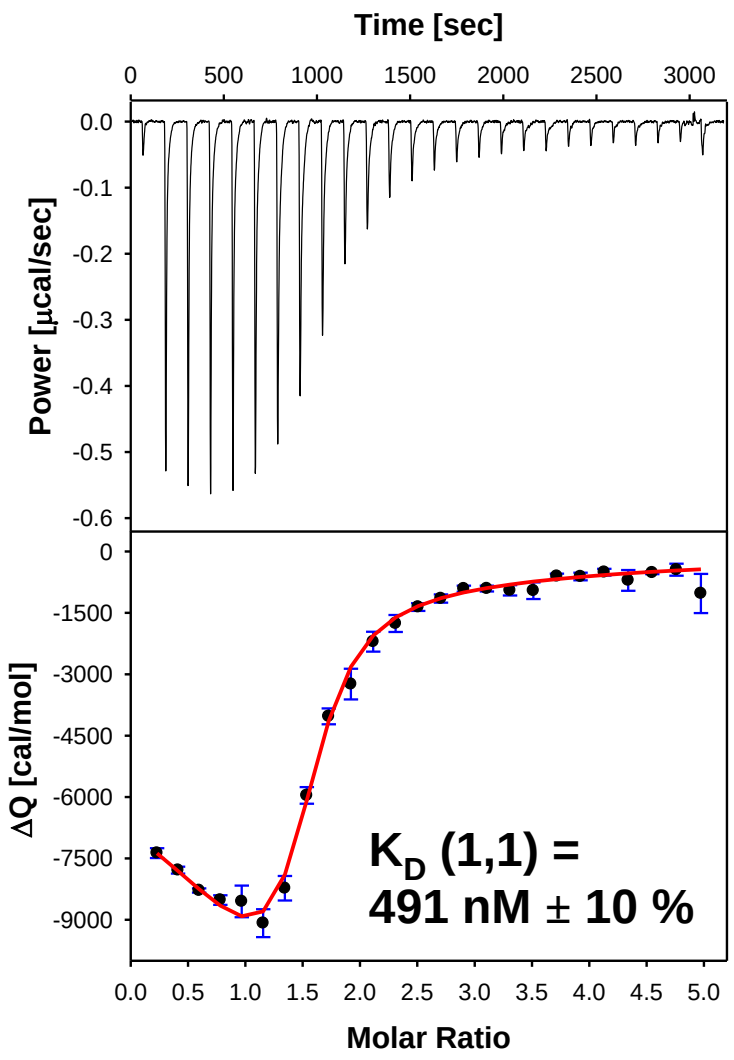

500 µM **8** To 20 µM **H4-Full**

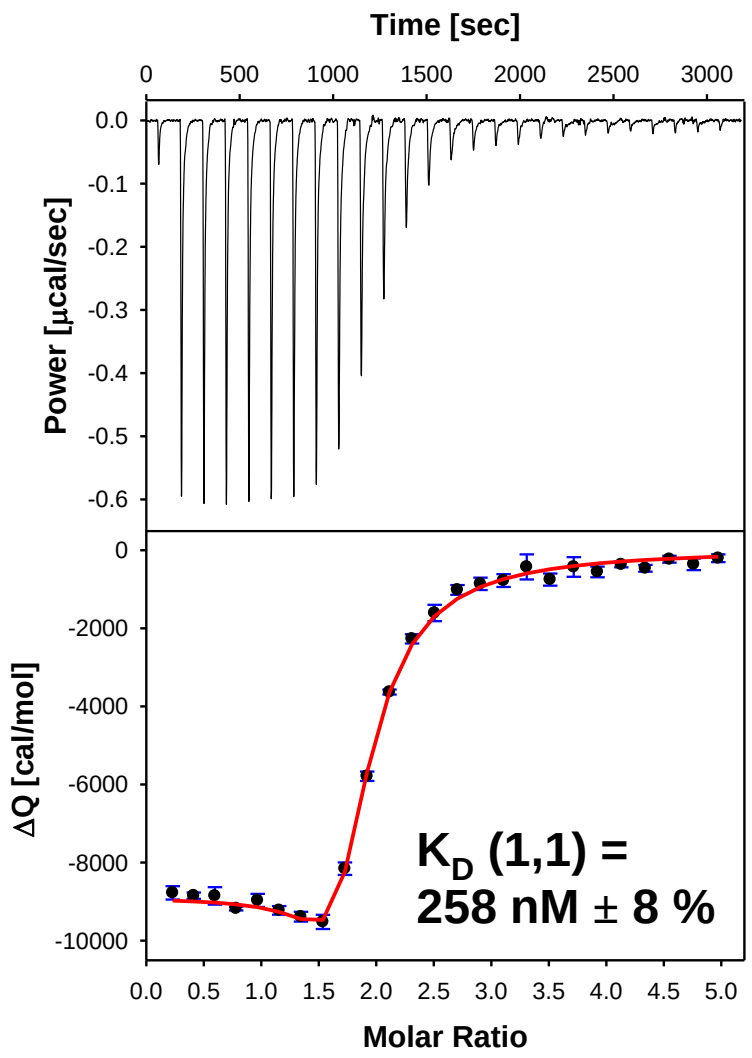

500 µM **9** To 20 µM **H4-Full**

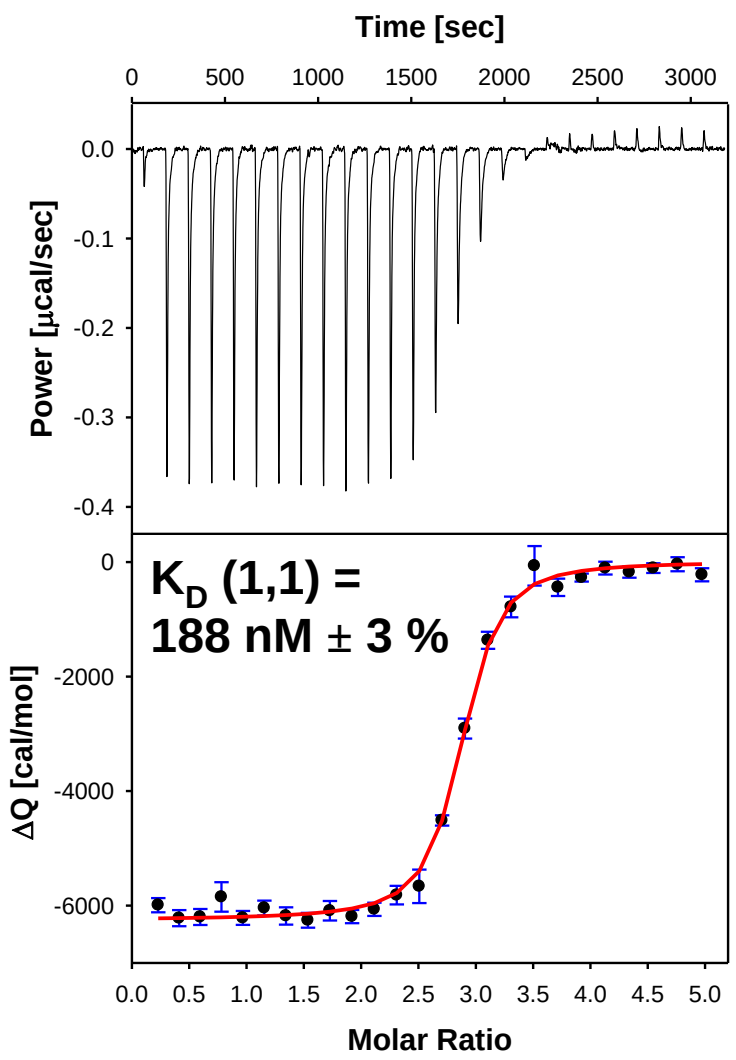

500 µM **10** To 20 µM **H4-Full**

Figure S10: ITC titrations of 1 mM **CLR01** or 500 µM advanced tweezers to 30 uM or 20 µM **H4-Full-Length** in 10 mM KPi buffer (pH 7.0) evaluated according to a simple 1SnS-model. K$_D$ (1,1): curve fitting for 1:1 complex.

Fluorescence titrations of new advanced tweezers with FAM-labeled H4 fragments:

Fluorescence titration of **FAM-H4-P1** with diphosphate tweezer **CLR01** in KPi buffer (10 mM, pH 7.0)

λ_{ex} = 495 nm	Tweezer	Peptide
λ_{em} = 522 nm	**CLR01**	**FAM-H4-P1**
Amount [mg]:	0.039	0.055
Volume [mL]:	1.5	31.74
Concentration [mol/L]:	3.19E-05	1.00E-06

Tweezer V (µL)	Peptide V (µL)	[Peptide] [mol/L]	[Tweezer] [mol/L]	[Tweezer]/ [Peptide]	F.I. (I_{522} nm)	ΔI_{obs}	ΔI_{calc}
0	700	1.00E-06	0.00E+00	0.00	5313	0	0
5	705	1.00E-06	2.26E-07	0.23	4583	730	554
10	710	1.00E-06	4.50E-07	0.45	4076	1236	1037
20	720	1.00E-06	8.87E-07	0.89	3412	1900	1791
30	730	1.00E-06	1.31E-06	1.31	3018	2294	2316
40	740	1.00E-06	1.73E-06	1.73	2646	2666	2686
60	760	1.00E-06	2.52E-06	2.52	2307	3006	3128
80	780	1.00E-06	3.27E-06	3.27	2040	3273	3377
100	800	1.00E-06	3.99E-06	3.99	1879	3434	3534
140	840	1.00E-06	5.32E-06	5.32	1593	3719	3715
180	880	1.00E-06	6.53E-06	6.53	1457	3856	3815
240	940	1.00E-06	8.15E-06	8.15	1354	3959	3903
300	1000	1.00E-06	9.58E-06	9.58	1317	3995	3956
400	1100	1.00E-06	1.16E-05	11.61	1267	4045	4008

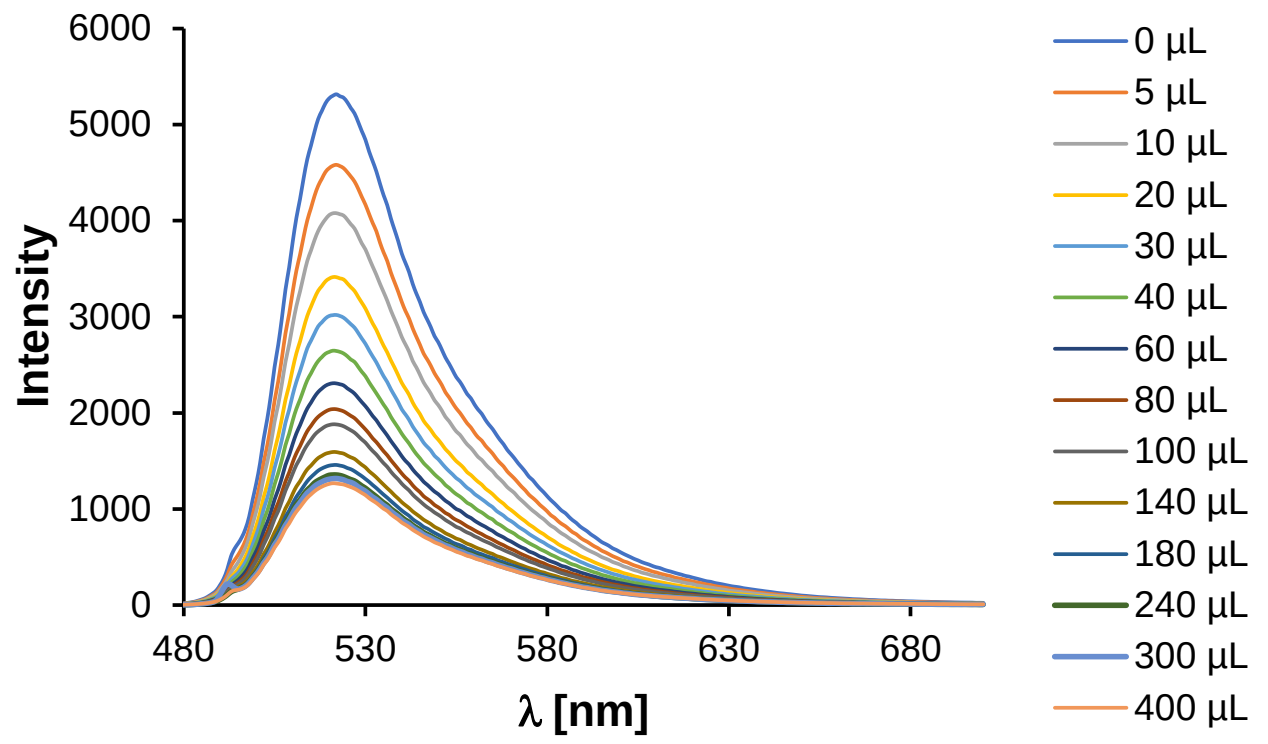

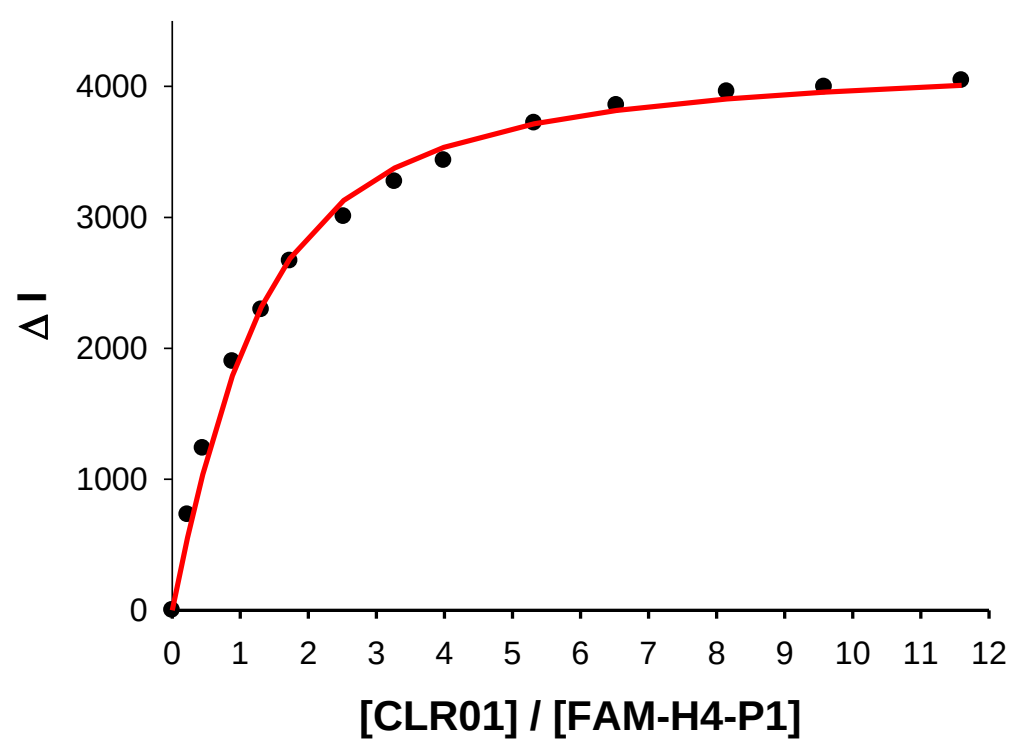

MA model	K_A = 1567100 M^{-1} ± 410931 M^{-1}
	K_D = 638 nM ± 3 %
	ΔI_{max} = 4247.8 ± 14.801

Fluorescence titration of **FAM-H4-P1** with peptide tweezer **2** in KPi buffer (10 mM, pH 7.0)

λ_{ex} = 495 nm	Tweezer	Peptide
λ_{em} = 522 nm	**2**	**FAM-H4-P1**
Amount [mg]:	0.033	0.055
Volume [mL]:	0.87	31.735
Concentration [mol/L]:	3.11E-05	1.00E-06

Tweezer V (µL)	Peptide V (µL)	[Peptide] [mol/L]	[Tweezer] [mol/L]	[Tweezer]/ [Peptide]	F·I· (I_{522} nm)	ΔI_{obs}	ΔI_{calc}
0	700	1.00E-06	0.00E+00	0.00	4364	0	0
5	705	1.00E-06	2.21E-07	0.22	4037	326	295
10	710	1.00E-06	4.38E-07	0.44	3687	677	578
20	720	1.00E-06	8.64E-07	0.86	3272	1092	1066
30	730	1.00E-06	1.28E-06	1.28	3120	1243	1282
40	740	1.00E-06	1.68E-06	1.68	3056	1308	1331
60	760	1.00E-06	2.46E-06	2.46	3004	1359	1357
80	780	1.00E-06	3.19E-06	3.19	3003	1360	1365
100	800	1.00E-06	3.89E-06	3.89	3051	1312	1369
140	840	1.00E-06	5.19E-06	5.19	3029	1335	1373
180	880	1.00E-06	6.37E-06	6.37	3045	1318	1375
240	940	1.00E-06	7.95E-06	7.95	2986	1378	1377
300	1000	1.00E-06	9.34E-06	9.34	2931	1432	1377
400	1100	1.00E-06	1.13E-05	11.32	2897	1466	1378

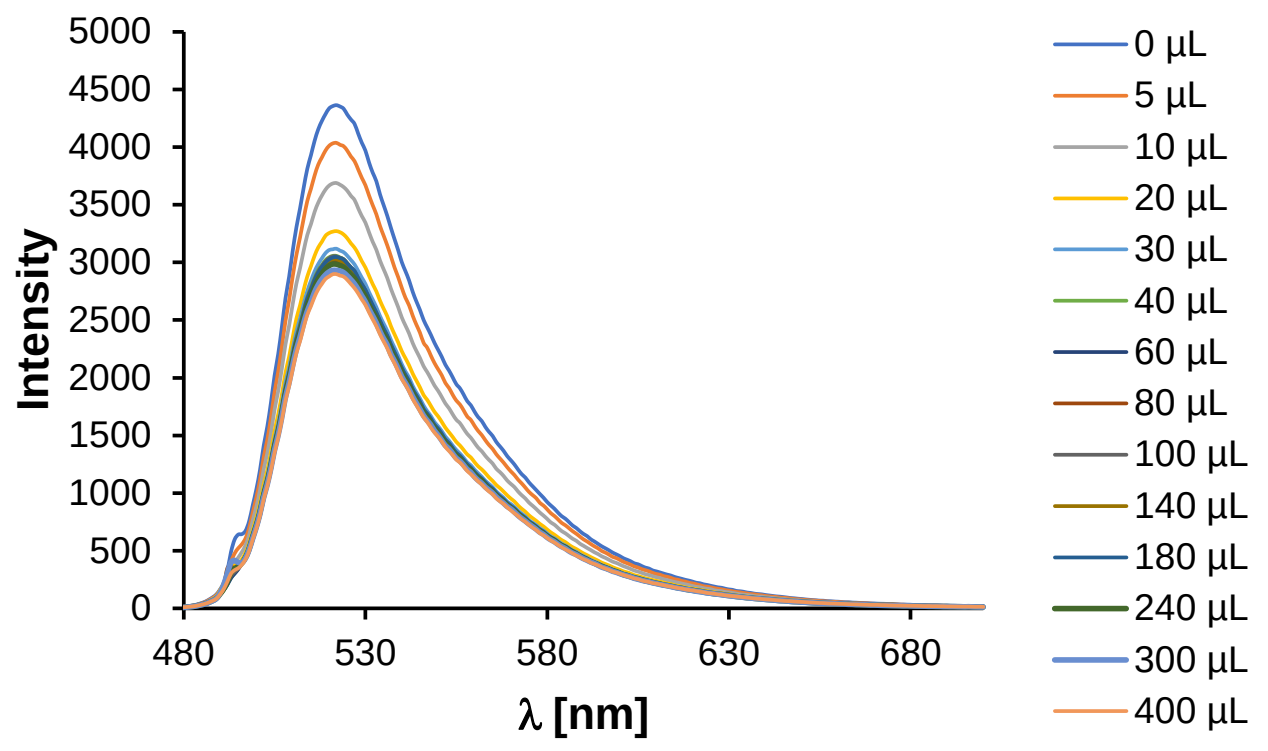

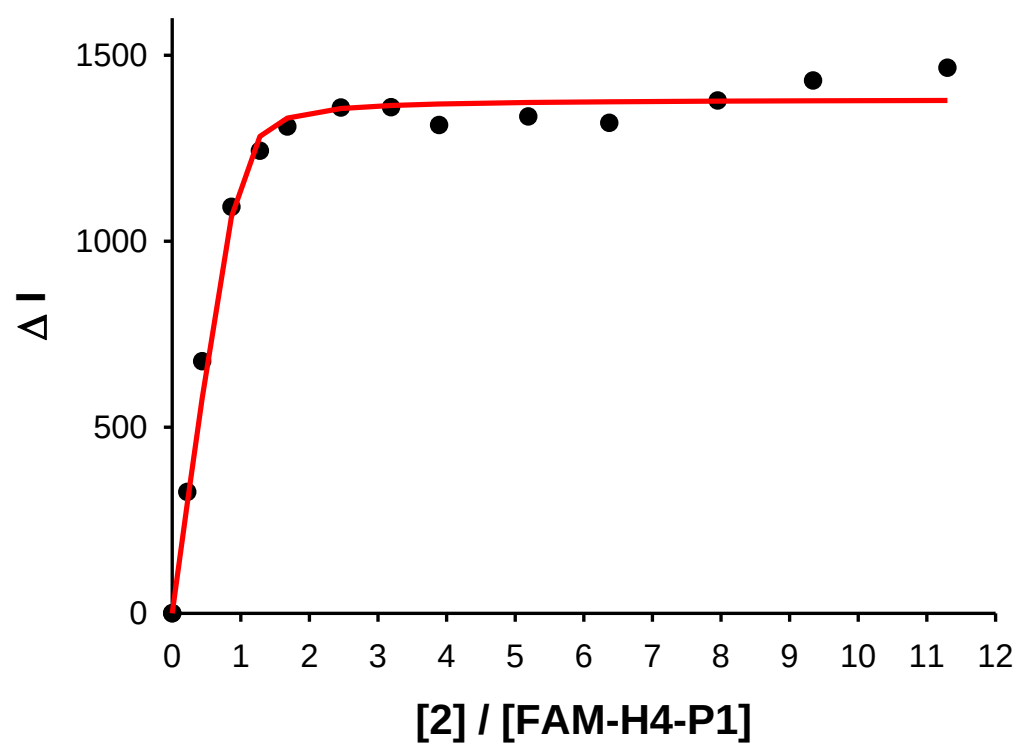

MA model	K_A = 36289000 M^{-1} ± 6429800 M^{-1}
	K_D = 28 nM ± 18 %
	ΔI_{max} = 1382 ± 6.674

Fluorescence titration of **FAM-H4-P1** with peptide tweezer **3** in KPi buffer (10 mM, pH 7.0)

λ_{ex} = 495 nm	Tweezer	Peptide
λ_{em} = 522 nm	**3**	**FAM-H4-P1**
Amount [mg]:	0.063	0.055
Volume [mL]:	1.5	31.735
Concentration [mol/L]:	3.23E-05	1.00E-06

Tweezer V (µL)	Peptide V (µL)	[Peptide] [mol/L]	[Tweezer] [mol/L]	[Tweezer]/ [Peptide]	F·I· (I_{522} nm)	ΔI_{obs}	ΔI_{calc}
0	700	1.00E-06	0.00E+00	0.00	4749	0	0
5	705	1.00E-06	2.29E-07	0.23	4031	718	440
10	710	1.00E-06	4.55E-07	0.46	3571	1178	858
20	720	1.00E-06	8.98E-07	0.90	3138	1611	1542
30	730	1.00E-06	1.33E-06	1.33	3004	1746	1839
40	740	1.00E-06	1.75E-06	1.75	2978	1772	1924
60	760	1.00E-06	2.55E-06	2.55	2910	1839	1975
80	780	1.00E-06	3.32E-06	3.32	2801	1948	1992
100	800	1.00E-06	4.04E-06	4.04	2807	1942	2001
140	840	1.00E-06	5.39E-06	5.39	2727	2022	2009
180	880	1.00E-06	6.61E-06	6.61	2724	2025	2014
240	940	1.00E-06	8.26E-06	8.26	2698	2051	2017
300	1000	1.00E-06	9.70E-06	9.70	2684	2065	2019
400	1100	1.00E-06	1.18E-05	11.76	2617	2132	2021

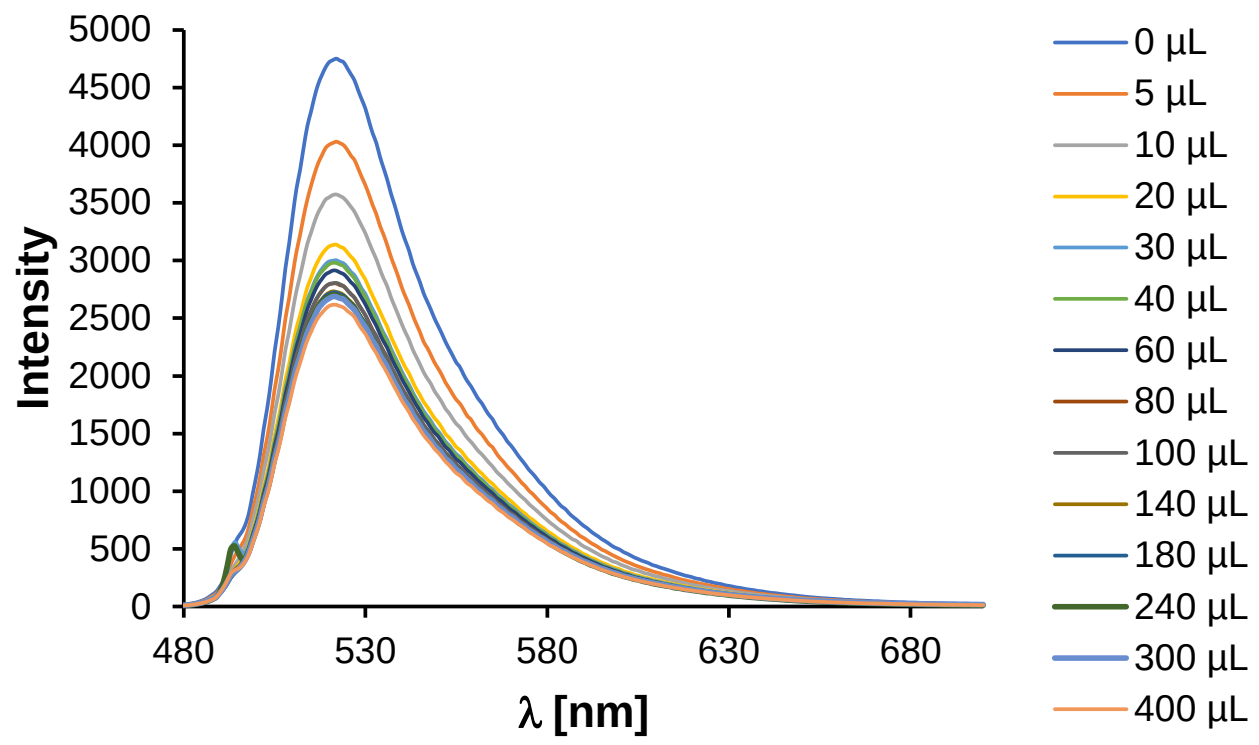

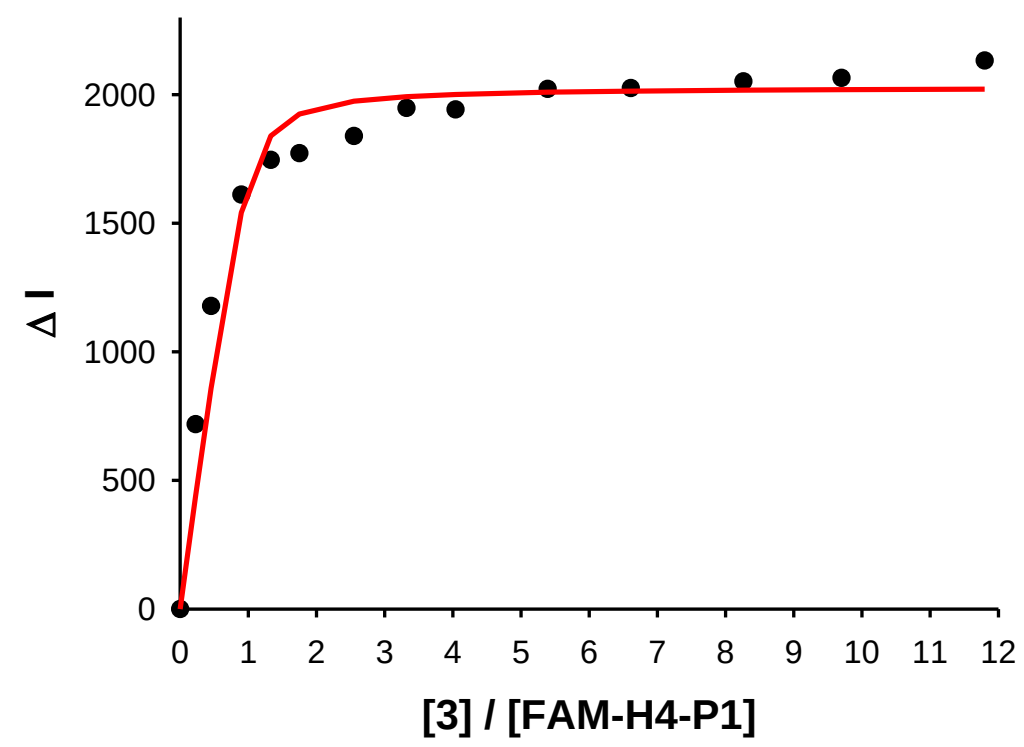

MA model	K_A = 22872000 M^{-1} ± 5052100 M^{-1}
	K_D = 44 nM ± 22 %
	ΔI_{max} = 2029.2 ± 9.8102

Fluorescence titration of **FAM-H4-P1** with peptide tweezer **4** in KPi buffer (10 mM, pH 7.0)

λ_{ex} = 495 nm	Tweezer	Peptide
λ_{em} = 522 nm	**4**	**FAM-H4-P1**
Amount [mg]:	0.047	0.055
Volume [mL]:	1.1	31.735
Concentration [mol/L]:	3.12E-05	1.00E-06

Tweezer V (µL)	Peptide V (µL)	[Peptide] [mol/L]	[Tweezer] [mol/L]	[Tweezer]/ [Peptide]	F·I· (I_{522} nm)	ΔI_{obs}	ΔI_{calc}
0	700	1.00E-06	0.00E+00	0.00	4586	0	0
5	705	1.00E-06	2.21E-07	0.22	3770	816	468
10	710	1.00E-06	4.39E-07	0.44	3287	1299	923
20	720	1.00E-06	8.66E-07	0.87	2990	1597	1729
30	730	1.00E-06	1.28E-06	1.28	2543	2043	2059
40	740	1.00E-06	1.69E-06	1.69	2409	2178	2115
60	760	1.00E-06	2.46E-06	2.46	2430	2157	2141
80	780	1.00E-06	3.20E-06	3.20	2440	2147	2149
100	800	1.00E-06	3.90E-06	3.90	2511	2075	2153
140	840	1.00E-06	5.20E-06	5.20	2459	2128	2157
180	880	1.00E-06	6.38E-06	6.38	2454	2132	2159
240	940	1.00E-06	7.96E-06	7.96	2451	2136	2160
300	1000	1.00E-06	9.36E-06	9.36	2437	2149	2161
400	1100	1.00E-06	1.13E-05	11.34	2446	2140	2162

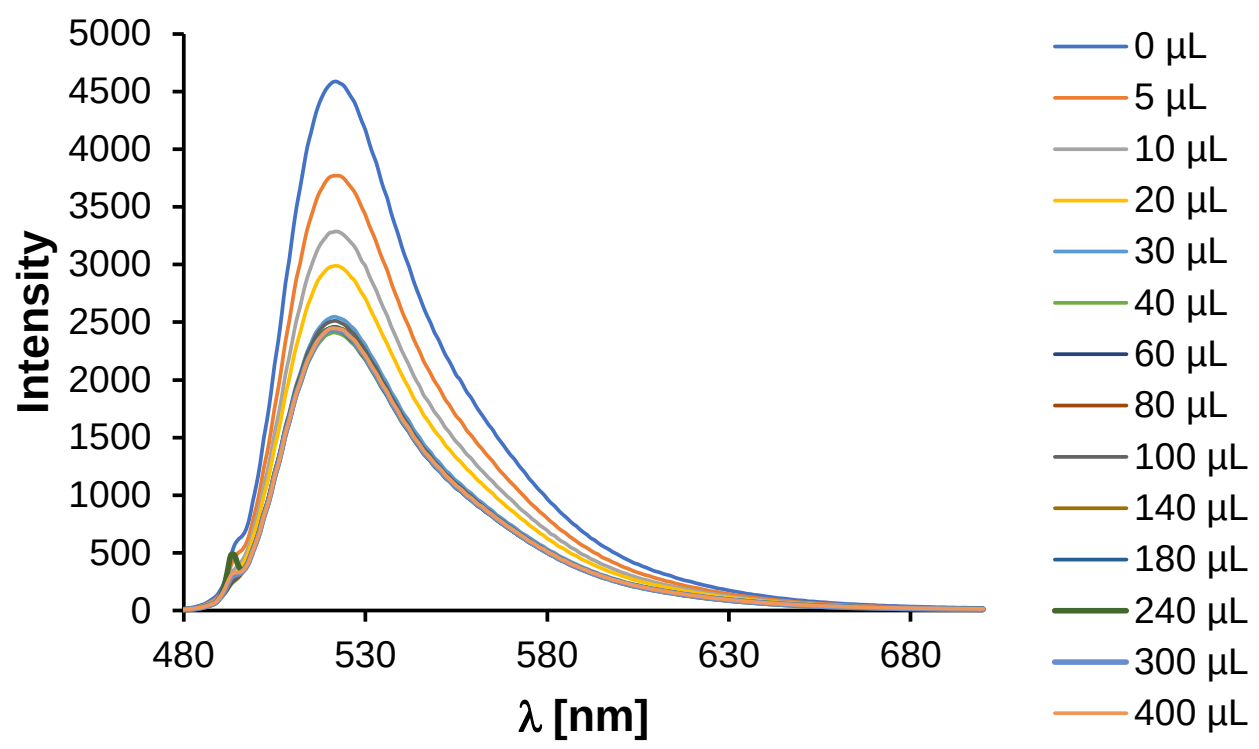

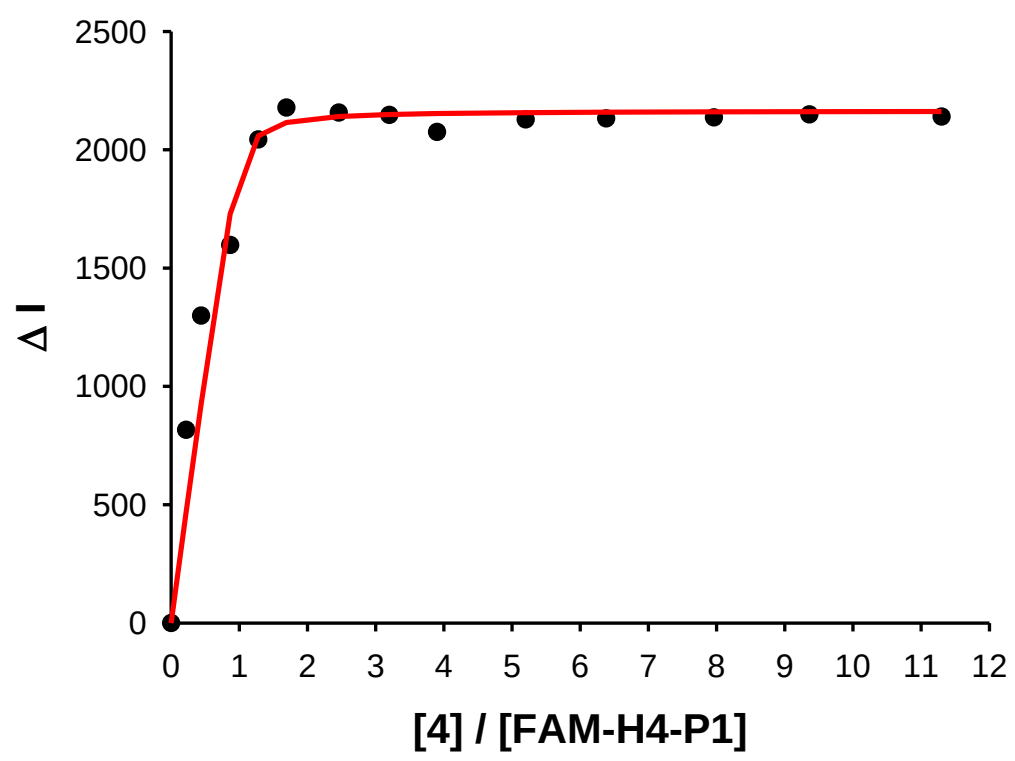

MA model	K_A = 58504000 M^{-1} ± 12471000 M^{-1}
	K_D = 17 nM ± 21 %
	ΔI_{max} = 2165.6 ± 4.3014

Fluorescence titration of **FAM-H4-P1** with Asp tweezer **5** in KPi buffer (10 mM, pH 7.0)

λ_{ex} = 495 nm<br>λ_{em} = 522 nm	Tweezer<br>**5**	Peptide<br>**FAM-H4-P1**
Amount [mg]:	0.044	0.055
Volume [mL]:	0.63	31.735
Concentration [mol/L]:	3.09E-05	1.00E-06

Tweezer V (µL)	Peptide V (µL)	[Peptide] [mol/L]	[Tweezer] [mol/L]	[Tweezer]/ [Peptide]	F·I· (I_{522} nm)	ΔI_{obs}	ΔI_{calc}
0	700	1.00E-06	0.00E+00	0.00	4615	0	0
5	705	1.00E-06	2.19E-07	0.22	3763	853	623
10	710	1.00E-06	4.35E-07	0.44	3007	1608	1227
20	720	1.00E-06	8.58E-07	0.86	2283	2333	2294
30	730	1.00E-06	1.27E-06	1.27	1967	2648	2753
40	740	1.00E-06	1.67E-06	1.67	1834	2781	2836
60	760	1.00E-06	2.44E-06	2.44	1779	2836	2876
80	780	1.00E-06	3.17E-06	3.17	1846	2770	2889
100	800	1.00E-06	3.86E-06	3.86	1846	2769	2895
140	840	1.00E-06	5.15E-06	5.15	1735	2881	2901
180	880	1.00E-06	6.32E-06	6.32	1700	2916	2904
240	940	1.00E-06	7.89E-06	7.89	1677	2938	2906
300	1000	1.00E-06	9.27E-06	9.27	1660	2956	2907
400	1100	1.00E-06	1.12E-05	11.23	1585	3030	2908

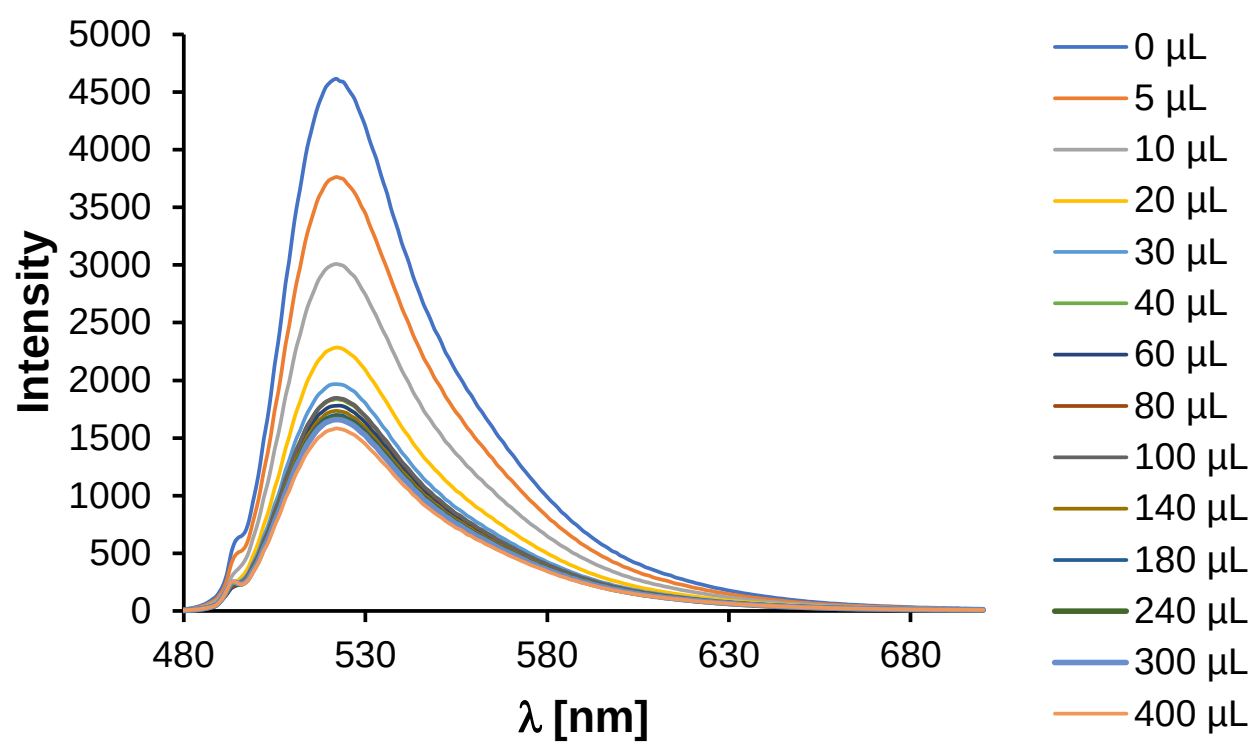

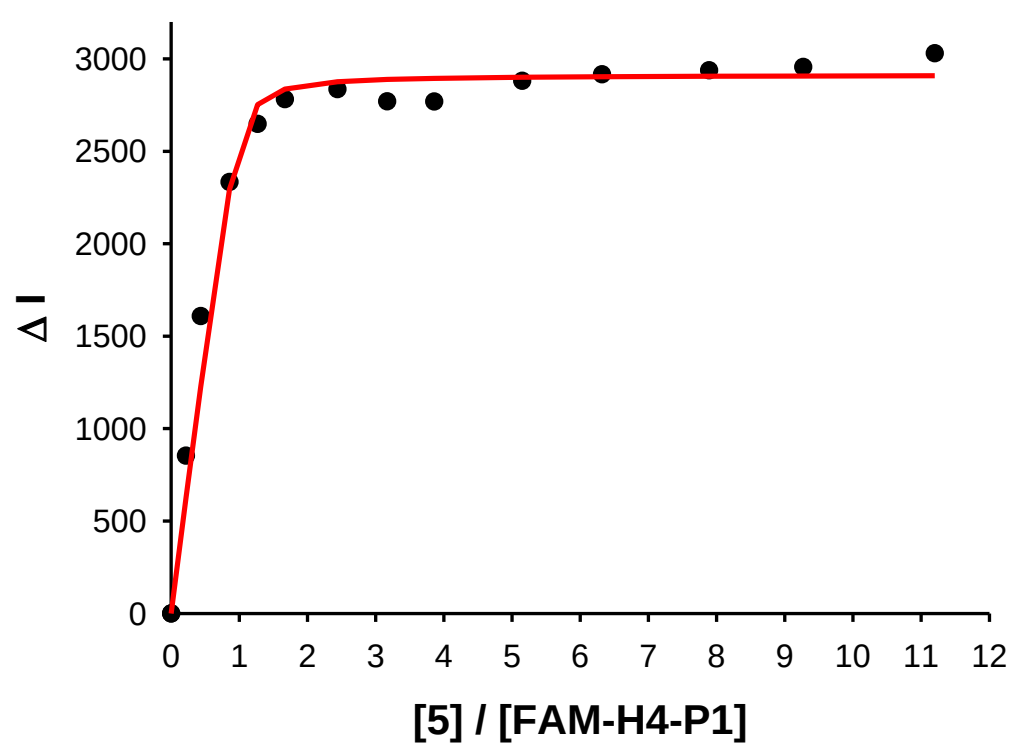

MA model	K_A = 52495000 M^{-1} ± 12889000 M^{-1}<br>K_D = 19 nM ± 25 %<br>ΔI_{max} = 2913.9 ± 9.659

Fluorescence titration of **FAM-H4-P1** with Asp tweezer **6** in KPi buffer (10 mM, pH 7.0)

λ_{ex} = 495 nm<br>λ_{em} = 522 nm	Tweezer<br>**6**	Peptide<br>**FAM-H4-P1**
Amount [mg]:	0.061	0.055
Volume [mL]:	1	31.735
Concentration [mol/L]:	3.07E-05	1.00E-06

Tweezer V (µL)	Peptide V (µL)	[Peptide] [mol/L]	[Tweezer] [mol/L]	[Tweezer]/ [Peptide]	F·I· (I_{522} nm)	ΔI_{obs}	ΔI_{calc}
0	700	1.00E-06	0.00E+00	0.00	4711	0	0
5	705	1.00E-06	2.18E-07	0.22	3888	823	602
10	710	1.00E-06	4.32E-07	0.43	3472	1239	1129
20	720	1.00E-06	8.53E-07	0.85	2727	1984	1955
30	730	1.00E-06	1.26E-06	1.26	2263	2448	2504
40	740	1.00E-06	1.66E-06	1.66	1834	2877	2862
60	760	1.00E-06	2.42E-06	2.42	1452	3259	3254
80	780	1.00E-06	3.15E-06	3.15	1348	3363	3457
100	800	1.00E-06	3.84E-06	3.84	1253	3458	3575
140	840	1.00E-06	5.12E-06	5.12	1023	3688	3705
180	880	1.00E-06	6.28E-06	6.28	914	3797	3775
240	940	1.00E-06	7.84E-06	7.84	847	3864	3834
300	1000	1.00E-06	9.21E-06	9.21	777	3934	3869
400	1100	1.00E-06	1.12E-05	11.17	775	3936	3904

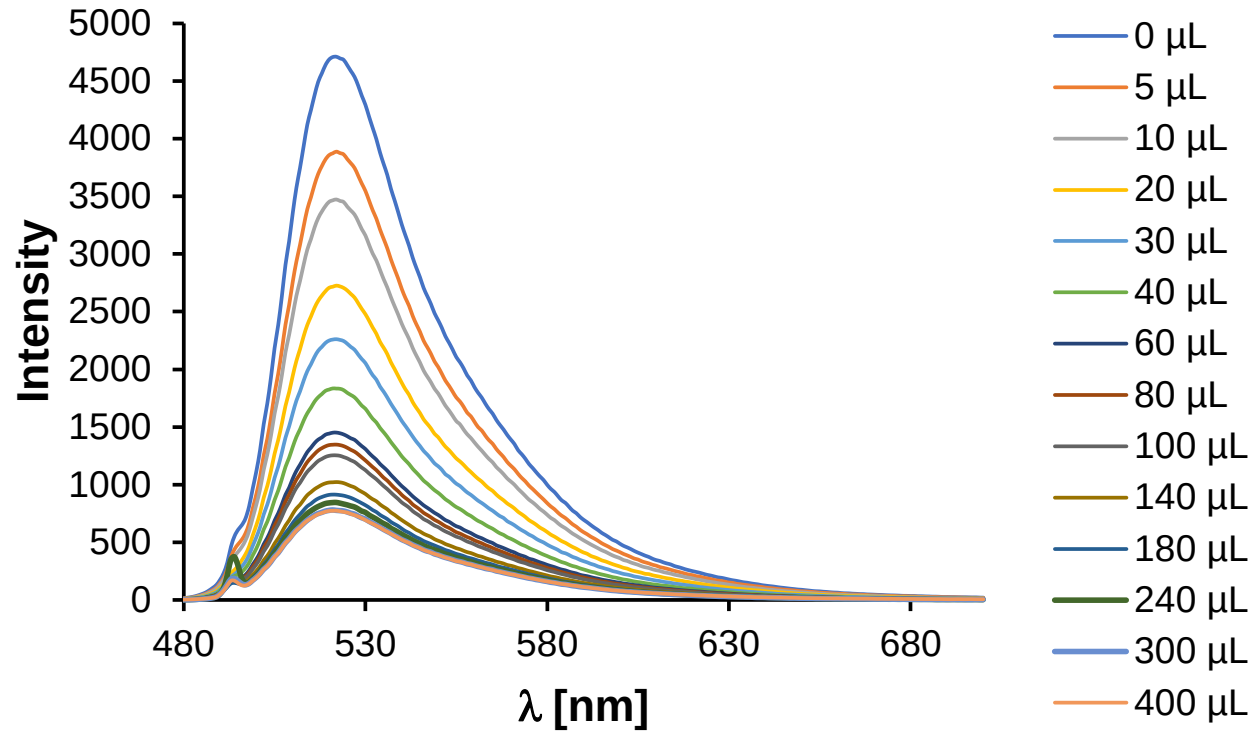

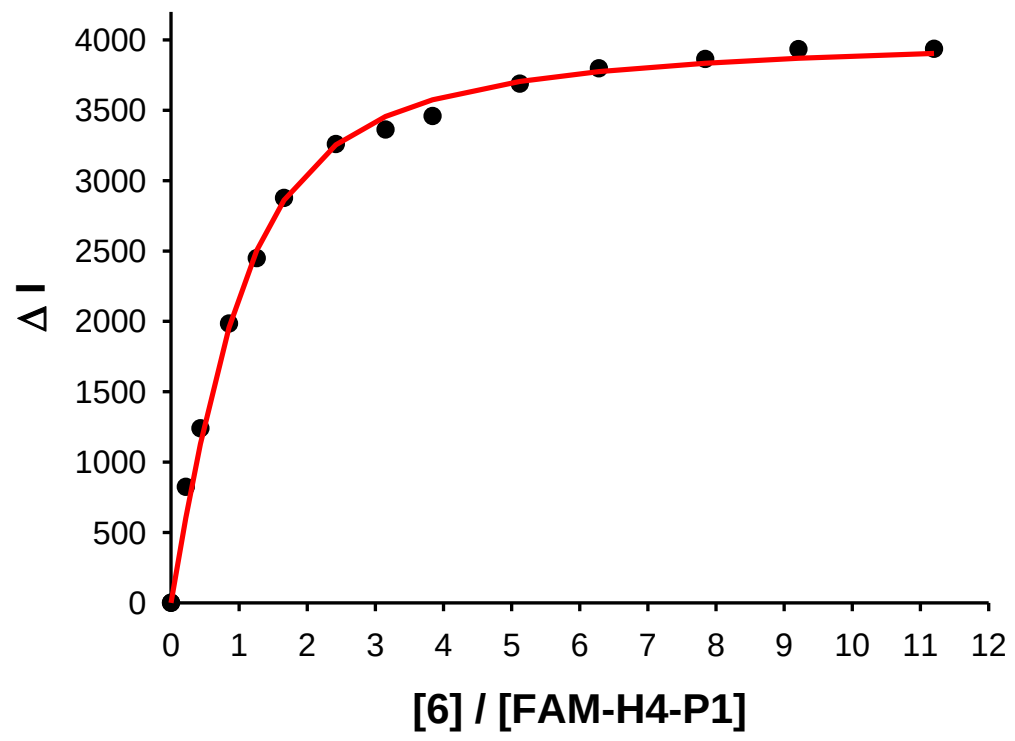

MA model	K_A = 2509800 M^{-1} ± 50547 M^{-1}<br>K_D = 398 nM ± 2 %<br>ΔI_{max} = 4056 ± 10.56

Fluorescence titration of **FAM-H4-P1** with bisphosphonate tweezer **7** in KPi buffer (10 mM, pH 7.0)

λ_{ex} = 495 nm	Tweezer	Peptide
λ_{em} = 522 nm	**7**	**FAM-H4-P1**
Amount [mg]:	0.050	0.055
Volume [mL]:	0.69	31.735
Concentration [mol/L]:	3.08E-05	1.00E-06

Tweezer V (µL)	Peptide V (µL)	[Peptide] [mol/L]	[Tweezer] [mol/L]	[Tweezer]/ [Peptide]	F·I· (I_{522} nm)	ΔI_{obs}	ΔI_{calc}
0	700	1.00E-06	0.00E+00	0.00	4587	0	0
5	705	1.00E-06	2.19E-07	0.22	3518	1069	605
10	710	1.00E-06	4.34E-07	0.43	2992	1594	1160
20	720	1.00E-06	8.56E-07	0.86	2414	2173	2044
30	730	1.00E-06	1.27E-06	1.27	2165	2422	2536
40	740	1.00E-06	1.67E-06	1.67	2060	2527	2757
60	760	1.00E-06	2.43E-06	2.43	1815	2772	2925
80	780	1.00E-06	3.16E-06	3.16	1682	2904	2990
100	800	1.00E-06	3.85E-06	3.85	1609	2977	3024
140	840	1.00E-06	5.14E-06	5.14	1505	3082	3058
180	880	1.00E-06	6.31E-06	6.31	1466	3121	3075
240	940	1.00E-06	7.87E-06	7.87	1468	3119	3090
300	1000	1.00E-06	9.25E-06	9.25	1425	3162	3098
400	1100	1.00E-06	1.12E-05	11.21	1411	3176	3105

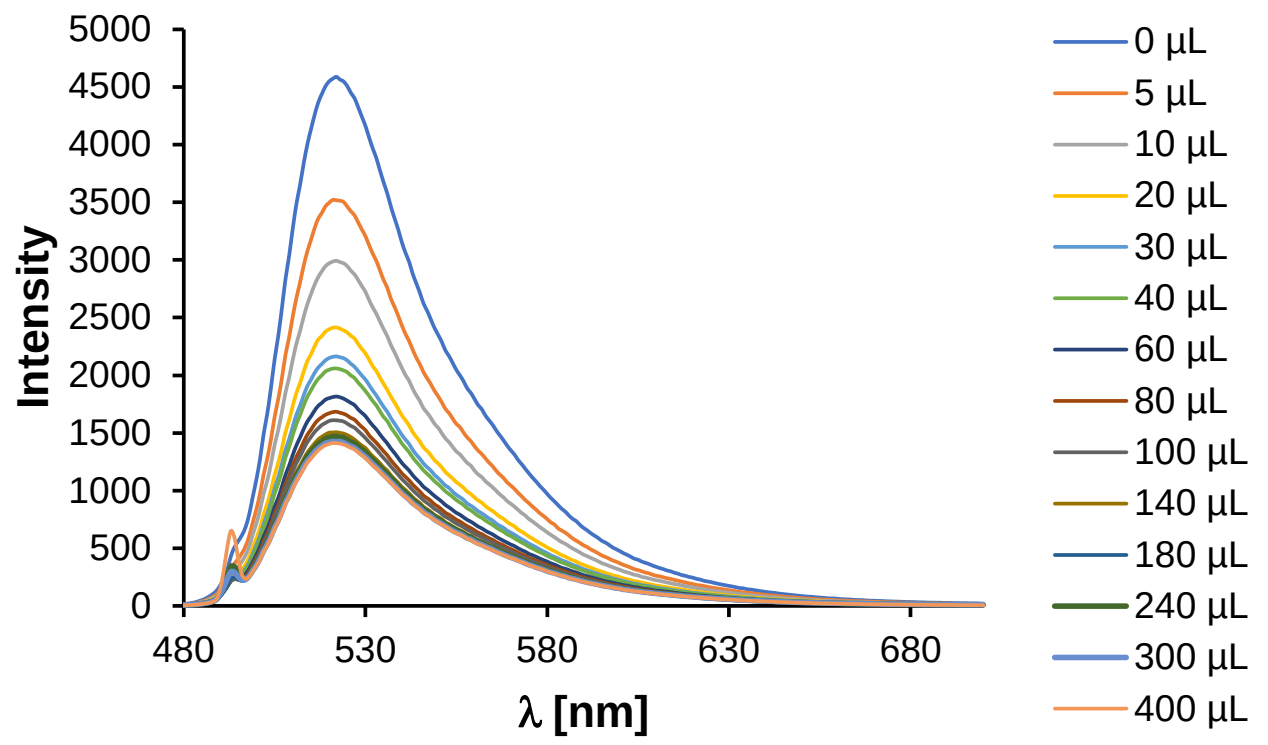

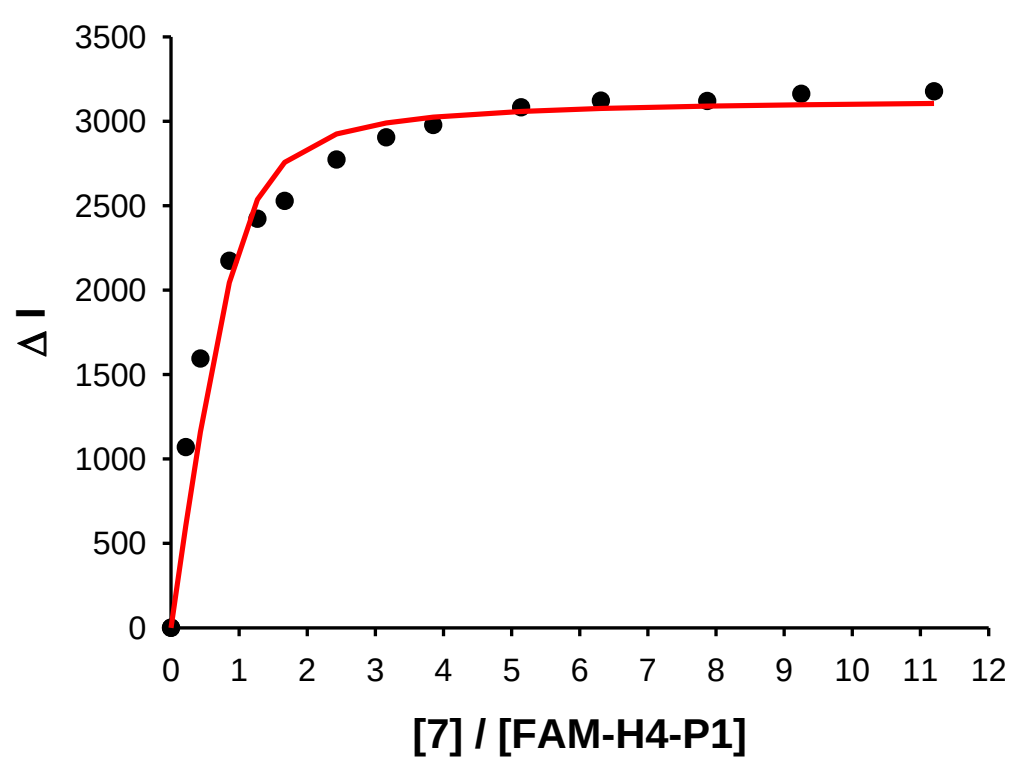

**_MA model_**	K_A = 9115500 M^{-1} ± 1170000 M^{-1}
	K_D = 110 nM ± 13 %
	ΔI_{max} = 3138.8 ± 13.298

Fluorescence titration of **FAM-H4-P1** with nitriloacetate tweezer **8** in KPi buffer (10 mM, pH 7.0)

λ_{ex} = 495 nm	Tweezer	Peptide
λ_{em} = 522 nm	**8**	**FAM-H4-P1**
Amount [mg]:	0.054	0.055
Volume [mL]:	0.77	31.735
Concentration [mol/L]:	3.07E-05	1.00E-06

Tweezer V (µL)	Peptide V (µL)	[Peptide] [mol/L]	[Tweezer] [mol/L]	[Tweezer]/ [Peptide]	F·I· (I_{522} nm)	ΔI_{obs}	ΔI_{calc}
0	700	1.00E-06	0.00E+00	0.00	4561	0	0
5	705	1.00E-06	2.18E-07	0.22	3815	746	569
10	710	1.00E-06	4.33E-07	0.43	3083	1478	1128
20	720	1.00E-06	8.53E-07	0.85	2337	2224	2183
30	730	1.00E-06	1.26E-06	1.26	2087	2474	2584
40	740	1.00E-06	1.66E-06	1.66	2015	2546	2608
60	760	1.00E-06	2.42E-06	2.42	1967	2594	2617
80	780	1.00E-06	3.15E-06	3.15	1947	2613	2619
100	800	1.00E-06	3.84E-06	3.84	1927	2633	2621
140	840	1.00E-06	5.12E-06	5.12	1977	2584	2622
180	880	1.00E-06	6.28E-06	6.28	1943	2618	2623
240	940	1.00E-06	7.84E-06	7.84	1954	2607	2623
300	1000	1.00E-06	9.21E-06	9.21	1950	2611	2623
400	1100	1.00E-06	1.12E-05	11.17	1902	2659	2624

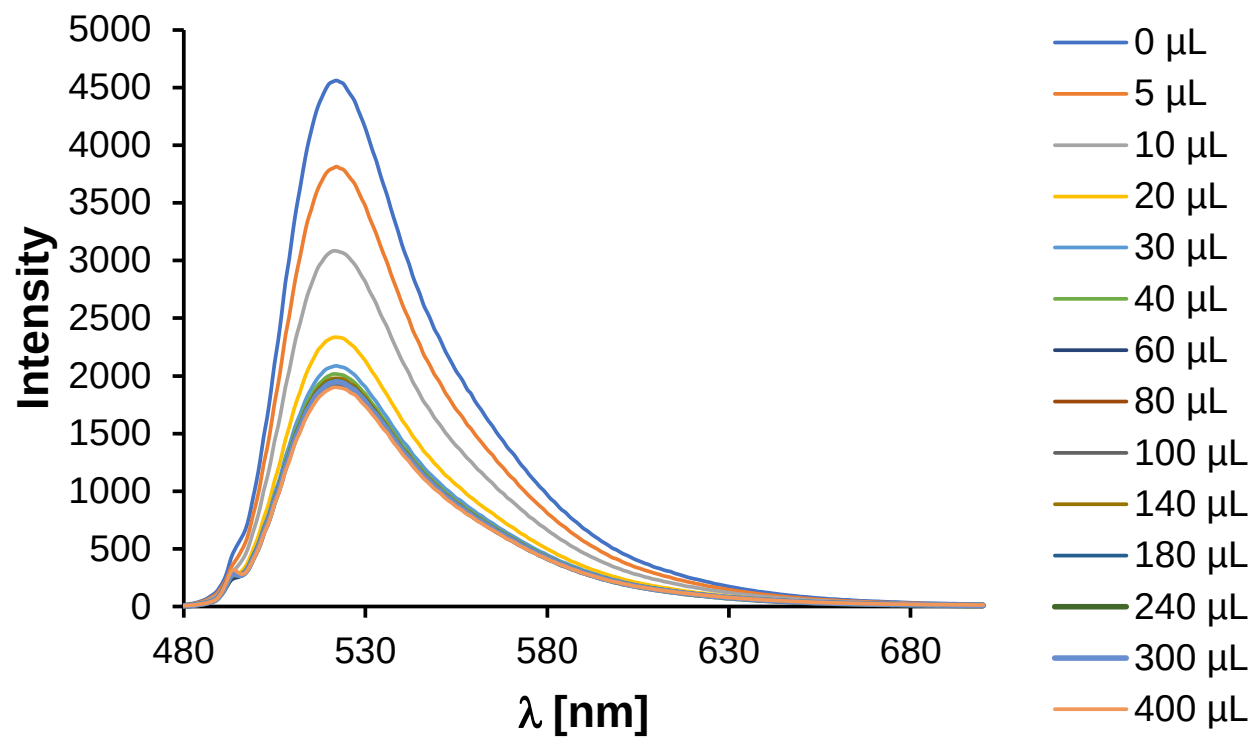

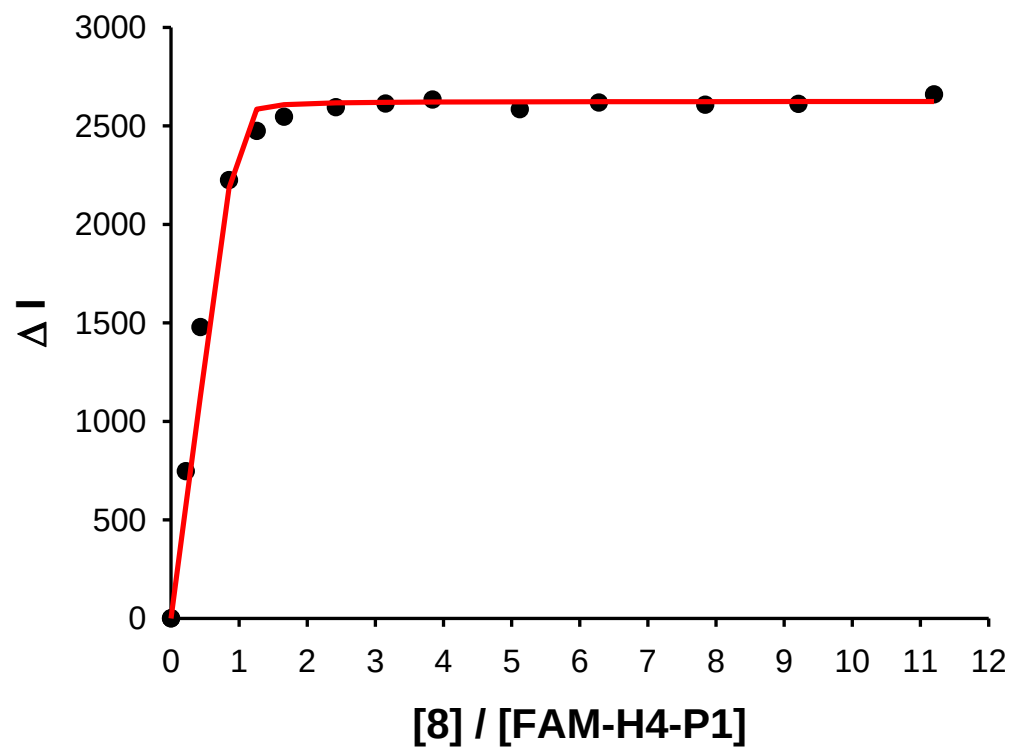

MA model	$K_A = 21323000\ M^{-1} \pm 1883800\ M^{-1}$
	K_D = 47 nM ± 9 %
	ΔI_{max} = 2647.2 ± 3.9434

Fluorescence titration of **FAM-H4-P1** with distilbene disulfonate tweezer **9** in KPi buffer (10 mM, pH 7.0)

λ_{ex} = 495 nm	Tweezer	Peptide
λ_{em} = 522 nm	**9**	**FAM-H4-P1**
Amount [mg]:	0.064	0.055
Volume [mL]:	0.95	31.735
Concentration [mol/L]:	3.13E-05	1.00E-06

Tweezer V (µL)	Peptide V (µL)	[Peptide] [mol/L]	[Tweezer] [mol/L]	[Tweezer]/ [Peptide]	F·I· (I_{522} nm)	ΔI_{obs}	ΔI_{calc}
0	700	1.00E-06	0.00E+00	0.00	4979	0	0
5	705	1.00E-06	2.22E-07	0.22	3942	1037	716
10	710	1.00E-06	4.40E-07	0.44	3258	1720	1415
20	720	1.00E-06	8.68E-07	0.87	2299	2679	2706
30	730	1.00E-06	1.28E-06	1.28	1853	3125	3178
40	740	1.00E-06	1.69E-06	1.69	1777	3202	3223
60	760	1.00E-06	2.47E-06	2.47	1747	3231	3242
80	780	1.00E-06	3.21E-06	3.21	1734	3244	3247
100	800	1.00E-06	3.91E-06	3.91	1766	3212	3250
140	840	1.00E-06	5.21E-06	5.21	1747	3231	3253
180	880	1.00E-06	6.39E-06	6.39	1753	3226	3254
240	940	1.00E-06	7.98E-06	7.98	1735	3243	3255
300	1000	1.00E-06	9.38E-06	9.38	1715	3264	3256
400	1100	1.00E-06	1.14E-05	11.36	1725	3253	3256

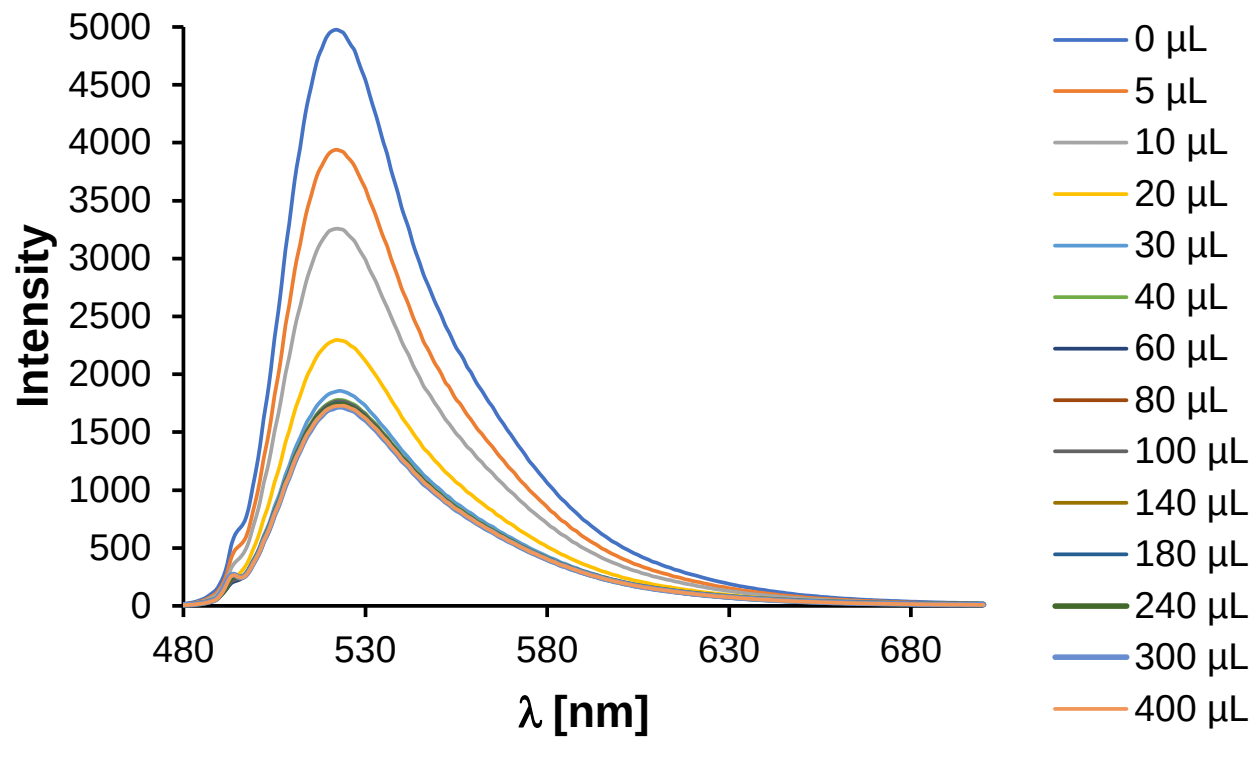

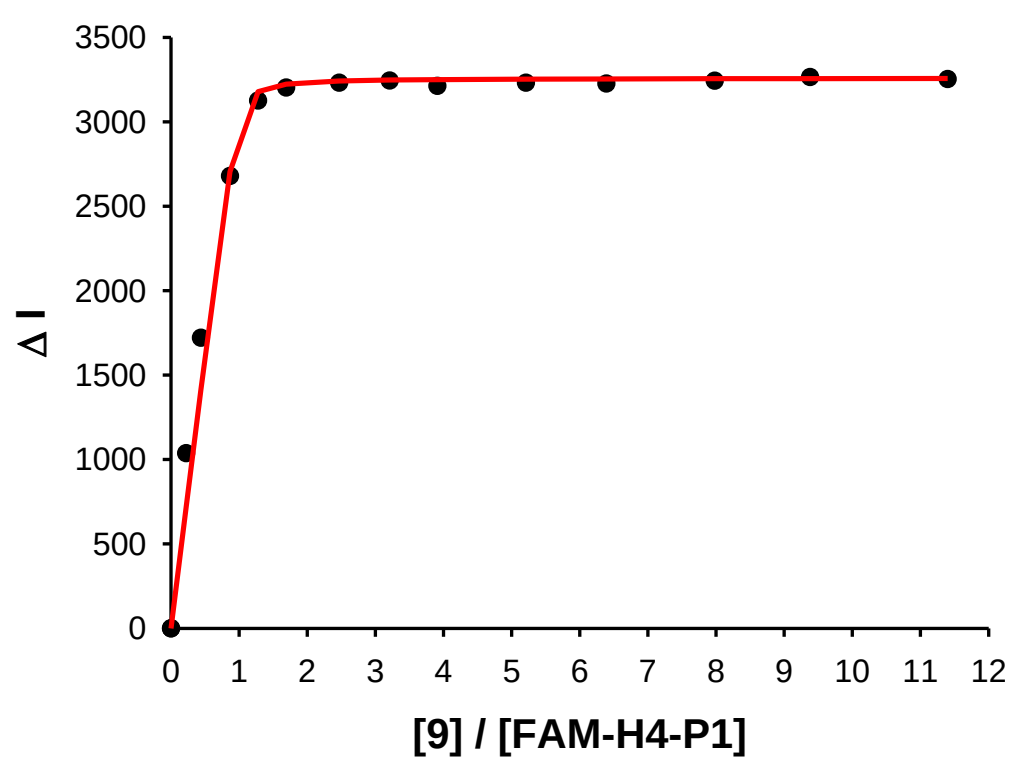

MA model	K_A = 129590000 M^{-1} ± 27528000 M^{-1}
	K_D = 7.7 nM ± 21 %
	ΔI_{max} = 3258.6 ± 3.4815

Fluorescence titration of **FAM-H4-P1** with PEG2 tweezer **10** in KPi buffer (10 mM, pH 7.0)

λ_{ex} = 495 nm	Tweezer	Peptide
λ_{em} = 522 nm	**10**	**FAM-H4-P1**
Amount [mg]:	0.046	0.055
Volume [mL]:	0.78	31.735
Concentration [mol/L]:	3.12E-05	1.00E-06

Tweezer V (µL)	Peptide V (µL)	[Peptide] [mol/L]	[Tweezer] [mol/L]	[Tweezer]/ [Peptide]	F·I· (I_{522} nm)	ΔI_{obs}	ΔI_{calc}
0	700	1.00E-06	0.00E+00	0.00	4583	0	0
5	705	1.00E-06	2.21E-07	0.22	3758	825	652
10	710	1.00E-06	4.40E-07	0.44	3107	1476	1253
20	720	1.00E-06	8.67E-07	0.87	2366	2218	2192
30	730	1.00E-06	1.28E-06	1.28	1879	2705	2712
40	740	1.00E-06	1.69E-06	1.69	1683	2900	2958
60	760	1.00E-06	2.46E-06	2.46	1509	3075	3148
80	780	1.00E-06	3.20E-06	3.20	1529	3054	3224
100	800	1.00E-06	3.90E-06	3.90	1413	3171	3263
140	840	1.00E-06	5.20E-06	5.20	1297	3286	3304
180	880	1.00E-06	6.38E-06	6.38	1196	3388	3324
240	940	1.00E-06	7.97E-06	7.97	1202	3381	3341
300	1000	1.00E-06	9.36E-06	9.36	1150	3434	3350
400	1100	1.00E-06	1.13E-05	11.35	1149	3434	3359

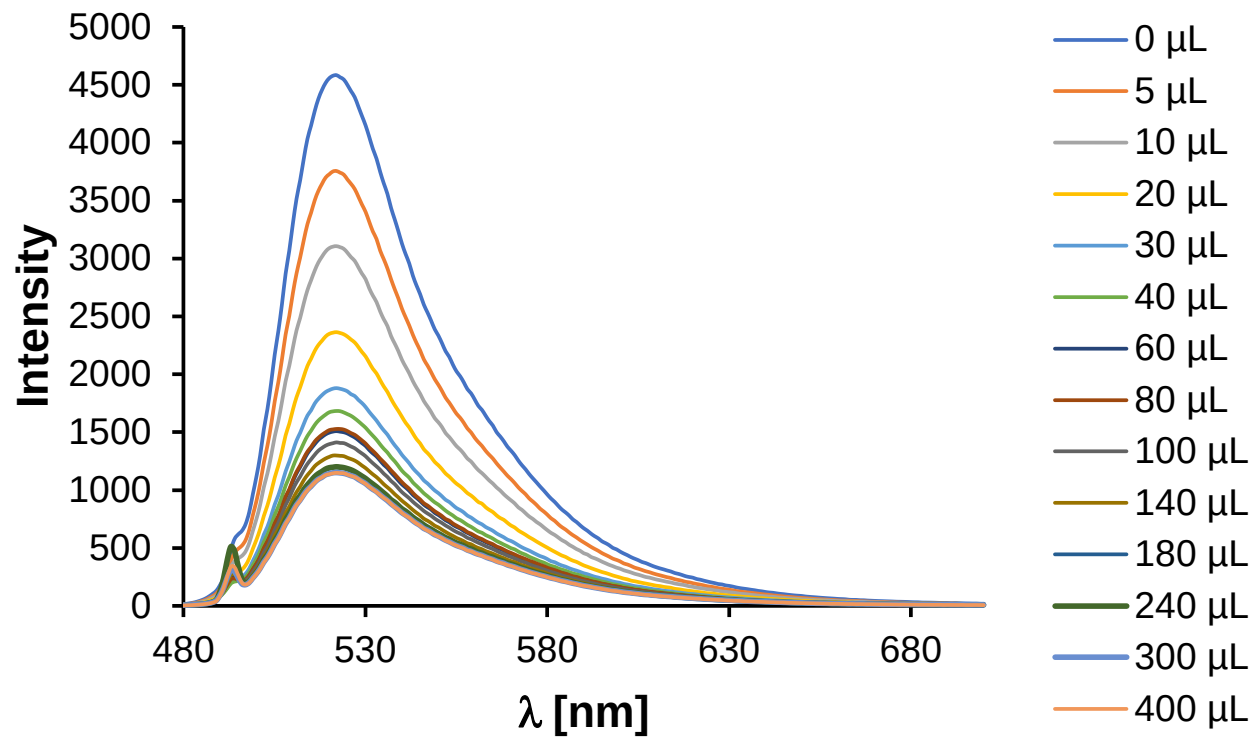

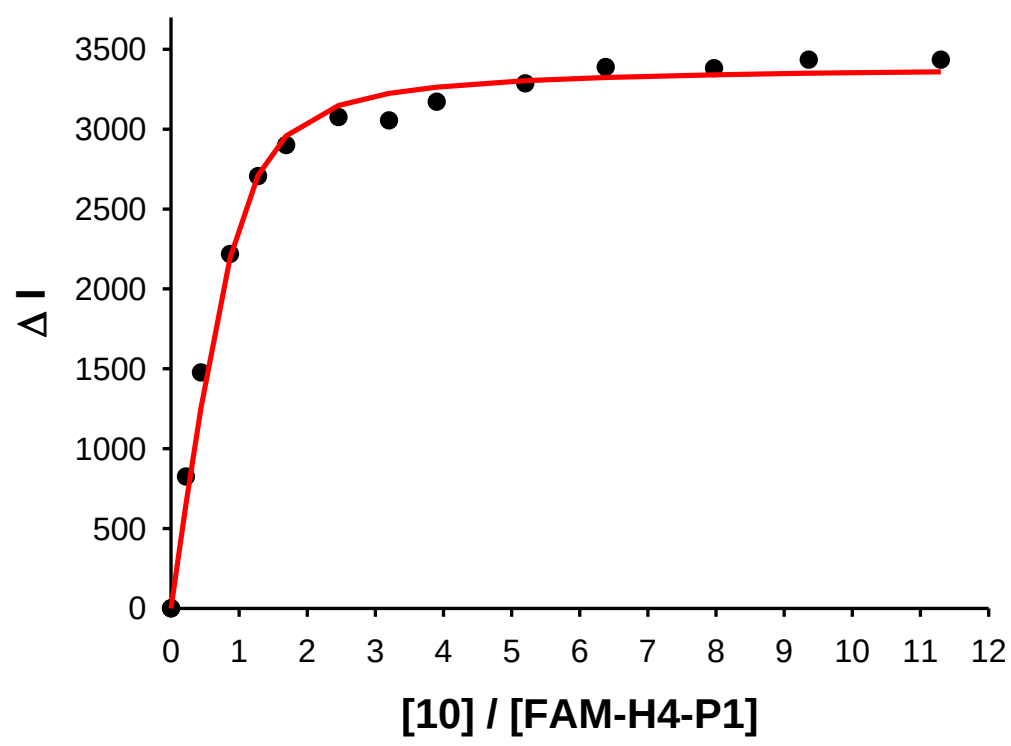

MA model	K_A = 8175500 M^{-1} ± 354400 M^{-1}
	K_D = 122 nM ± 4 %
	ΔI_{max} = 3399.2 ± 11.909

Fluorescence titration of **FAM-H4-P1** with diphenylsulfone tweezer **11** in KPi buffer (10 mM, pH 7.0)

λ_{ex} = 495 nm	Tweezer	Peptide
λ_{em} = 522 nm	**11**	**FAM-H4-P1**
Amount [mg]:	0.058	0.055
Volume [mL]:	0.93	31.735
Concentration [mol/L]:	3.13E-05	1.00E-06

Tweezer V (µL)	Peptide V (µL)	[Peptide] [mol/L]	[Tweezer] [mol/L]	[Tweezer]/ [Peptide]	F·I· (I_{522} nm)	ΔI_{obs}	ΔI_{calc}
0	700	1.00E-06	0.00E+00	0.00	4754	0	0
5	705	1.00E-06	2.22E-07	0.22	3963	791	676
10	710	1.00E-06	4.41E-07	0.44	3446	1308	1275
20	720	1.00E-06	8.71E-07	0.87	2586	2167	2201
30	730	1.00E-06	1.29E-06	1.29	2006	2747	2784
40	740	1.00E-06	1.69E-06	1.69	1562	3191	3126
60	760	1.00E-06	2.47E-06	2.47	1292	3461	3478
80	780	1.00E-06	3.21E-06	3.21	1138	3616	3643
100	800	1.00E-06	3.92E-06	3.92	1035	3718	3736
140	840	1.00E-06	5.22E-06	5.22	929	3825	3836
180	880	1.00E-06	6.41E-06	6.41	886	3868	3888
240	940	1.00E-06	8.00E-06	8.00	834	3919	3932
300	1000	1.00E-06	9.40E-06	9.40	798	3956	3958
400	1100	1.00E-06	1.14E-05	11.40	706	4048	3983

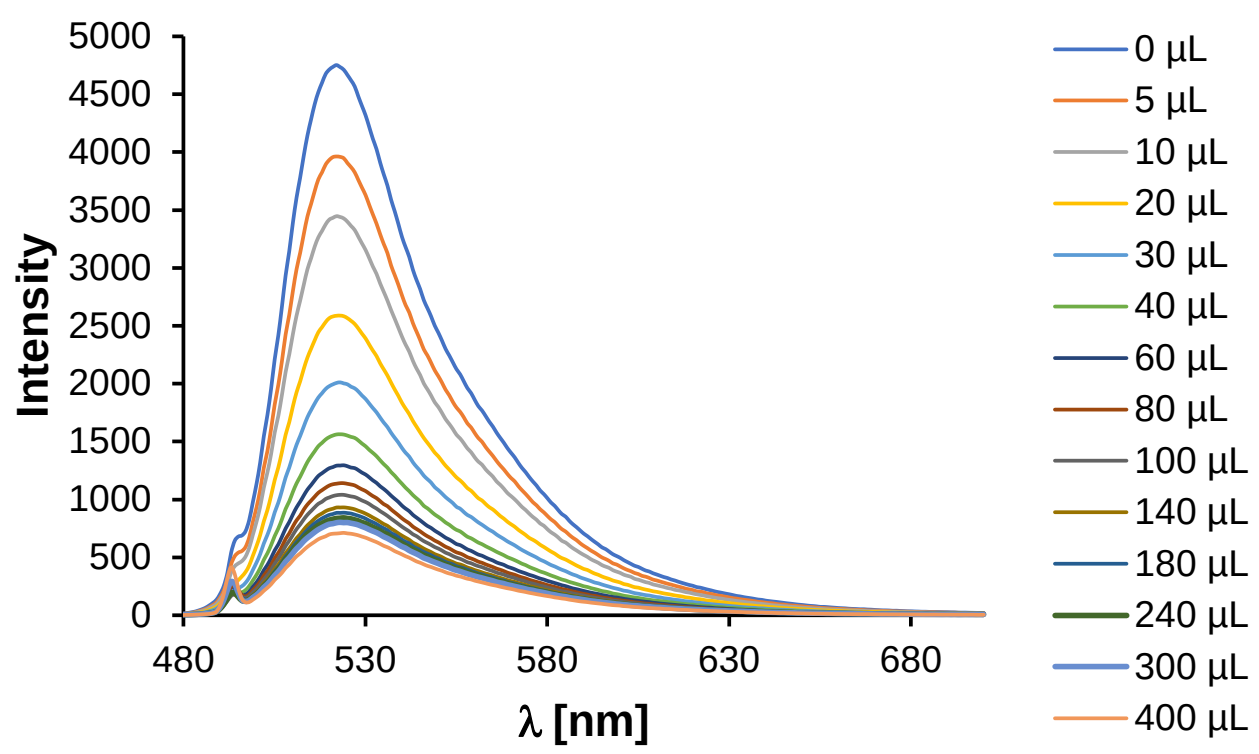

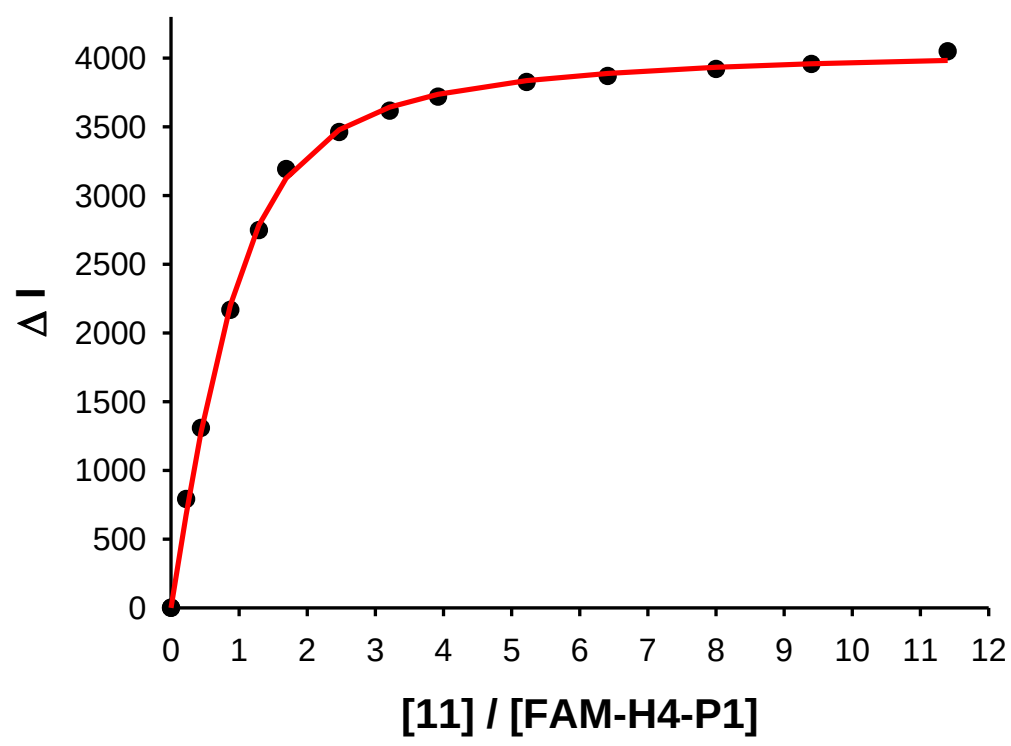

MA model	K_A = 3491800 M^{-1} ± 64068 M^{-1}
	K_D = 286 nM ± 2 %
	ΔI_{max} = 4092.3 ± 7.1221

Fluorescence titration of **FAM-H4-Full-Length** with diphosphate tweezer **CLR01** in KPi buffer (10 mM, pH 7.0)

λ_{ex} = 495 nm	Tweezer	Peptide
λ_{em} = 522 nm	**CLR01**	**FAM-H4-Full-Length**
Amount [mg]:	0.042	0.14
Volume [mL]:	1.7	46.369
Concentration [mol/L]:	3.03E-05	1.00E-06

Tweezer V (µL)	Peptide V (µL)	[Peptide] [mol/L]	[Tweezer] [mol/L]	[Tweezer]/ [Peptide]	F·I· (I_{522} nm)	ΔI_{obs}	ΔI_{calc}
0	700	1.00E-06	0.00E+00	0.00	4816	0	0
5	705	1.00E-06	2.15E-07	0.22	4365	452	588
10	710	1.00E-06	4.27E-07	0.43	3780	1036	1102
20	720	1.00E-06	8.42E-07	0.84	2935	1881	1916
30	730	1.00E-06	1.25E-06	1.25	2300	2517	2490
40	740	1.00E-06	1.64E-06	1.64	1879	2937	2878
60	760	1.00E-06	2.39E-06	2.39	1455	3361	3348
80	780	1.00E-06	3.11E-06	3.11	1200	3616	3609
100	800	1.00E-06	3.79E-06	3.79	1036	3780	3769
140	840	1.00E-06	5.05E-06	5.05	871	3945	3951
180	880	1.00E-06	6.20E-06	6.20	774	4042	4052
240	940	1.00E-06	7.74E-06	7.74	693	4123	4140
300	1000	1.00E-06	9.10E-06	9.10	627	4189	4192
400	1100	1.00E-06	1.10E-05	11.03	570	4246	4242

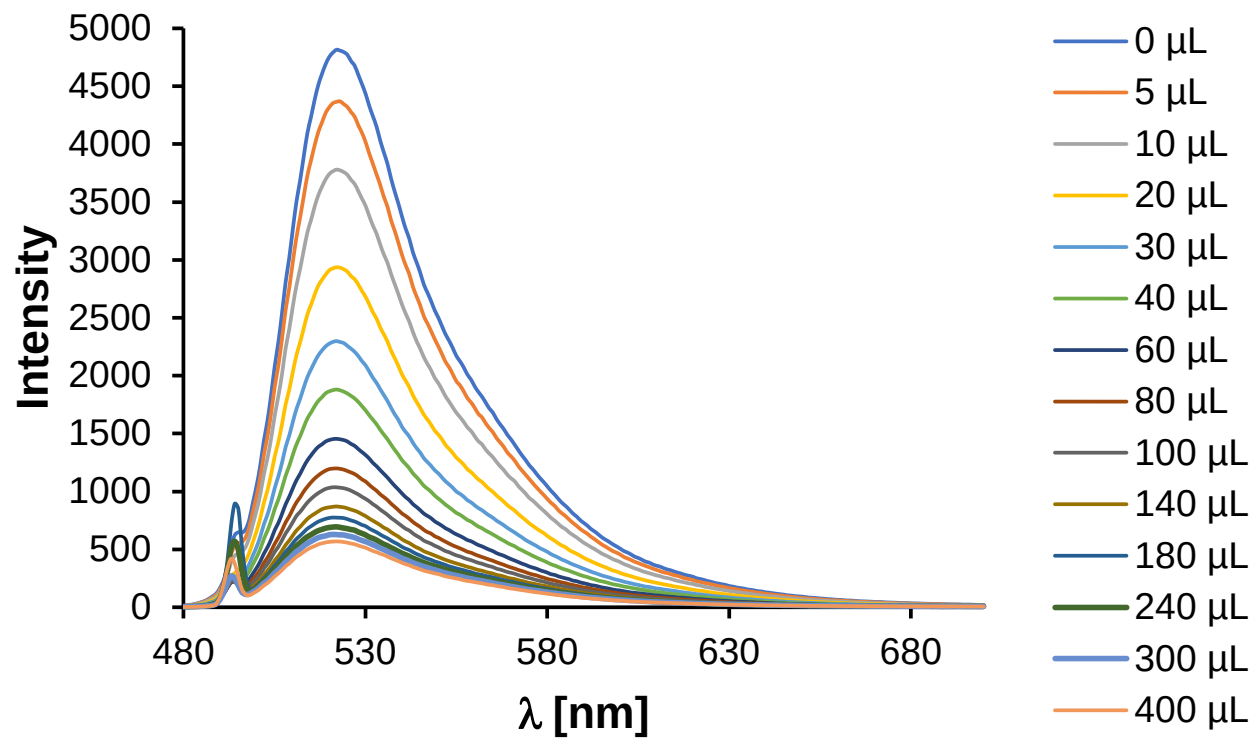

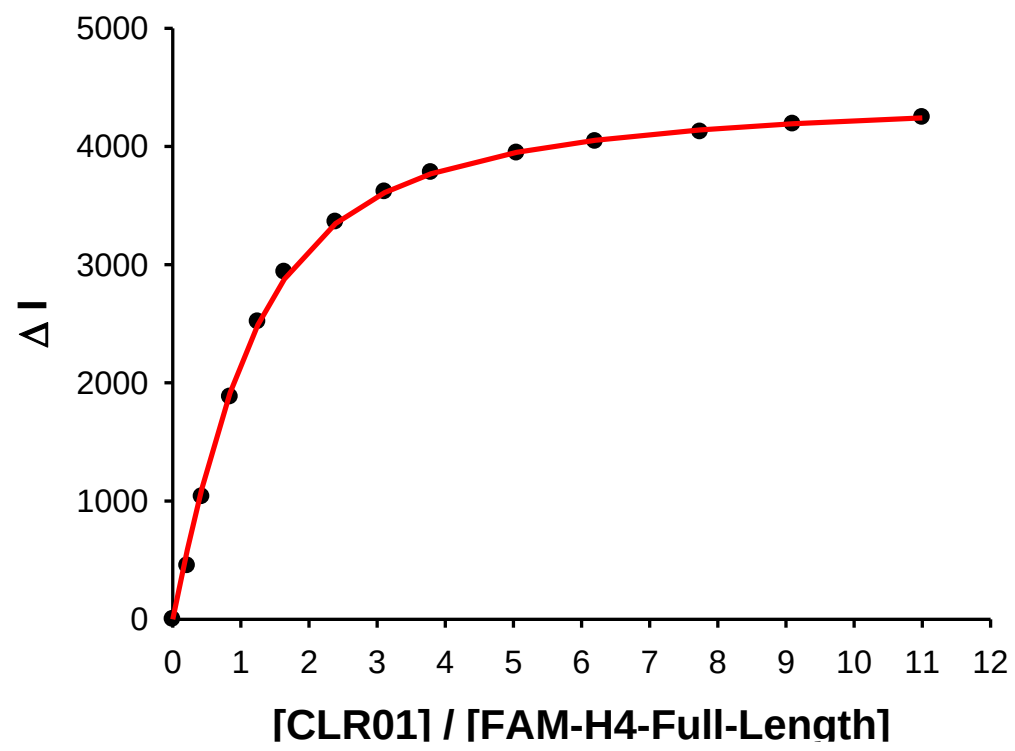

MA model	K_A = 1807900 M^{-1} ± 21360 M^{-1}
	K_D = 553 nM ± 1.2 %
	ΔI_{max} = 4475.6 ± 5.3966

Fluorescence titration of **FAM-H4-Full-Length** with peptide tweezer **2** in KPi buffer (10 mM, pH 7.0)

λ_{ex} = 495 nm<br>λ_{em} = 522 nm	Tweezer<br>**2**	Peptide<br>**FAM-H4-Full-Length**
Amount [mg]:	0.042	0.14
Volume [mL]:	1.1	46.369
Concentration [mol/L]:	3.13E-05	1.00E-06

Tweezer V (µL)	Peptide V (µL)	[Peptide] [mol/L]	[Tweezer] [mol/L]	[Tweezer]/ [Pepitd]	F·I· (I_{522} nm)	ΔI_{obs}	ΔI_{calc}
0	700	1.00E-06	0.00E+00	0.00	4890	0	0
5	705	1.00E-06	2.22E-07	0.22	4080	810	467
10	710	1.00E-06	4.41E-07	0.44	3857	1033	879
20	720	1.00E-06	8.70E-07	0.87	3240	1650	1516
30	730	1.00E-06	1.29E-06	1.29	2906	1984	1927
40	740	1.00E-06	1.69E-06	1.69	2718	2172	2174
60	760	1.00E-06	2.47E-06	2.47	2656	2234	2438
80	780	1.00E-06	3.21E-06	3.21	2559	2332	2566
100	800	1.00E-06	3.92E-06	3.92	2469	2421	2639
140	840	1.00E-06	5.22E-06	5.22	2196	2694	2718
180	880	1.00E-06	6.41E-06	6.41	2089	2801	2760
240	940	1.00E-06	8.00E-06	8.00	2030	2860	2796
300	1000	1.00E-06	9.40E-06	9.40	1999	2891	2816
400	1100	1.00E-06	1.14E-05	11.39	1831	3059	2837

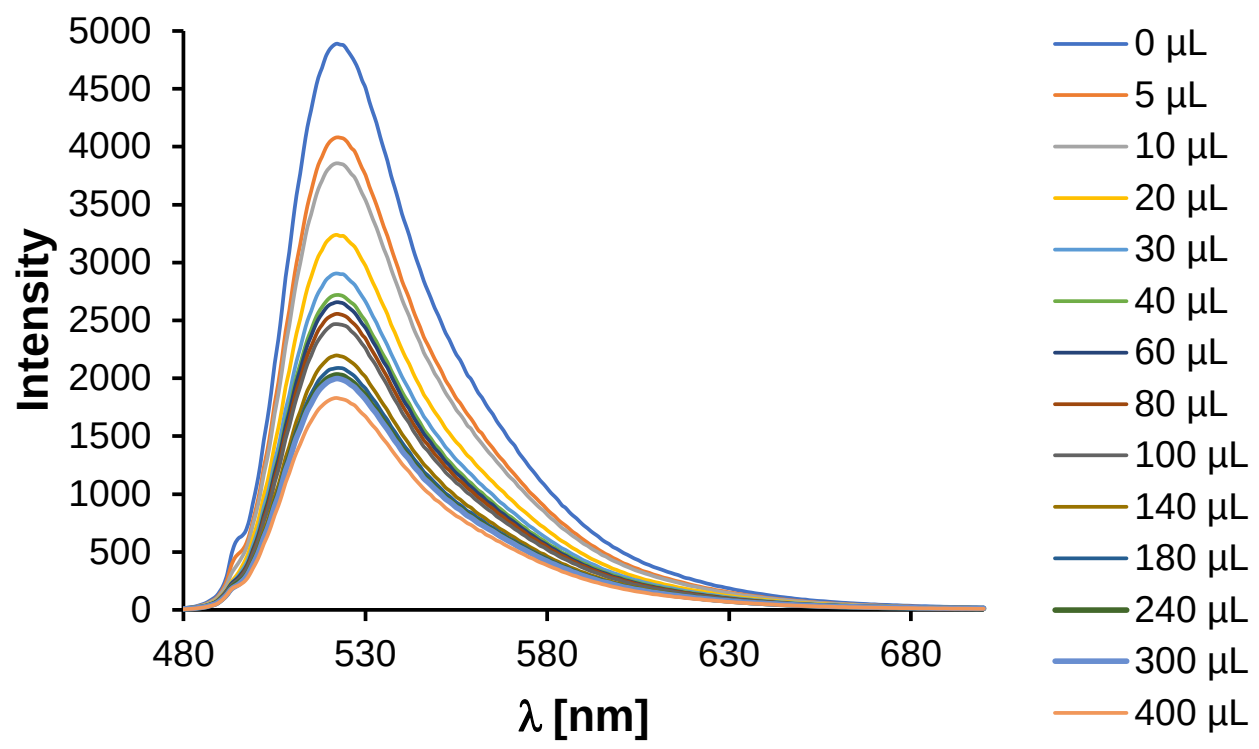

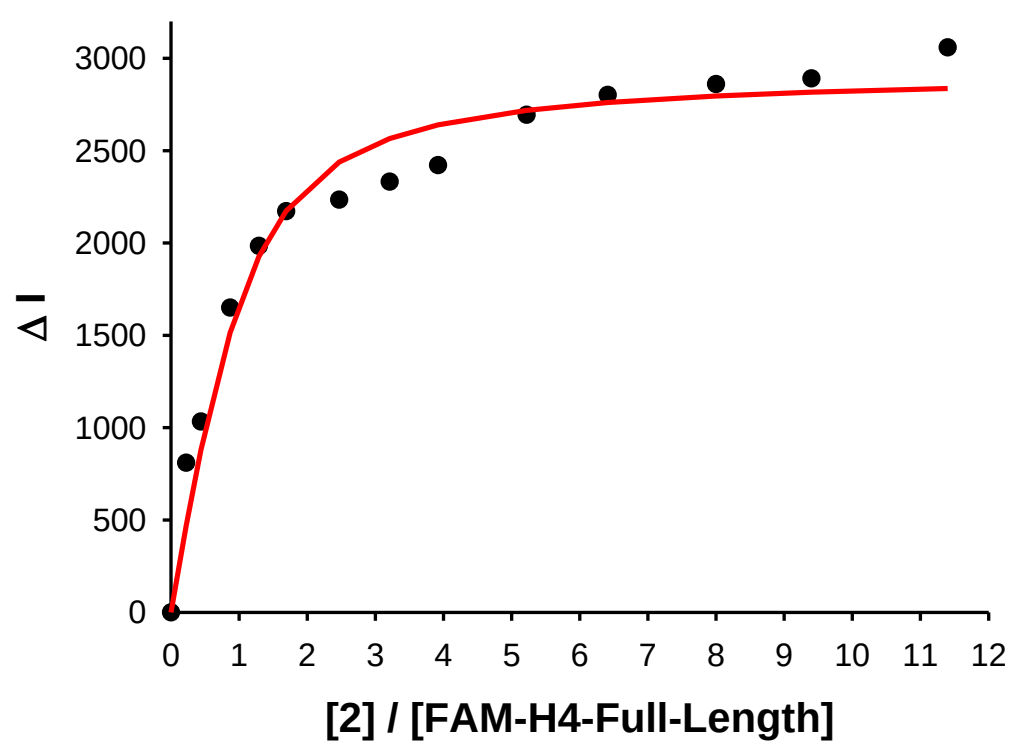

MA model	K_A = 3052700 M^{-1} ± 243680 M^{-1}<br>K_D = 328 nM ± 8 %<br>ΔI_{max} = 2925.8 ± 28.906

Fluorescence titration of **FAM-H4-Full-Length** with peptide tweezer **3** in KPi buffer (10 mM, pH 7.0)

λ_{ex} = 495 nm	Tweezer	Peptide
λ_{em} = 522 nm	**3**	**FAM-H4-Full-Length**
Amount [mg]:	0.053	0.14
Volume [mL]:	1.3	46.369
Concentration [mol/L]:	3.14E-05	1.00E-06

Tweezer V (µL)	Peptide V (µL)	[Peptide] [mol/L]	[Tweezer] [mol/L]	[Tweezer]/ [Pepitd]	F·I· (I_{522} nm)	ΔI_{obs}	ΔI_{calc}
0	700	1.00E-06	0.00E+00	0.00	5020	0	0
5	705	1.00E-06	2.23E-07	0.22	4548	472	464
10	710	1.00E-06	4.42E-07	0.44	4173	848	878
20	720	1.00E-06	8.72E-07	0.87	3557	1464	1520
30	730	1.00E-06	1.29E-06	1.29	3107	1913	1904
40	740	1.00E-06	1.70E-06	1.70	2874	2146	2114
60	760	1.00E-06	2.48E-06	2.48	2695	2325	2305
80	780	1.00E-06	3.22E-06	3.22	2586	2434	2389
100	800	1.00E-06	3.92E-06	3.92	2560	2461	2435
140	840	1.00E-06	5.23E-06	5.23	2534	2486	2483
180	880	1.00E-06	6.42E-06	6.42	2598	2422	2507
240	940	1.00E-06	8.01E-06	8.01	2505	2515	2528
300	1000	1.00E-06	9.42E-06	9.42	2492	2528	2540
400	1100	1.00E-06	1.14E-05	11.41	2439	2581	2551

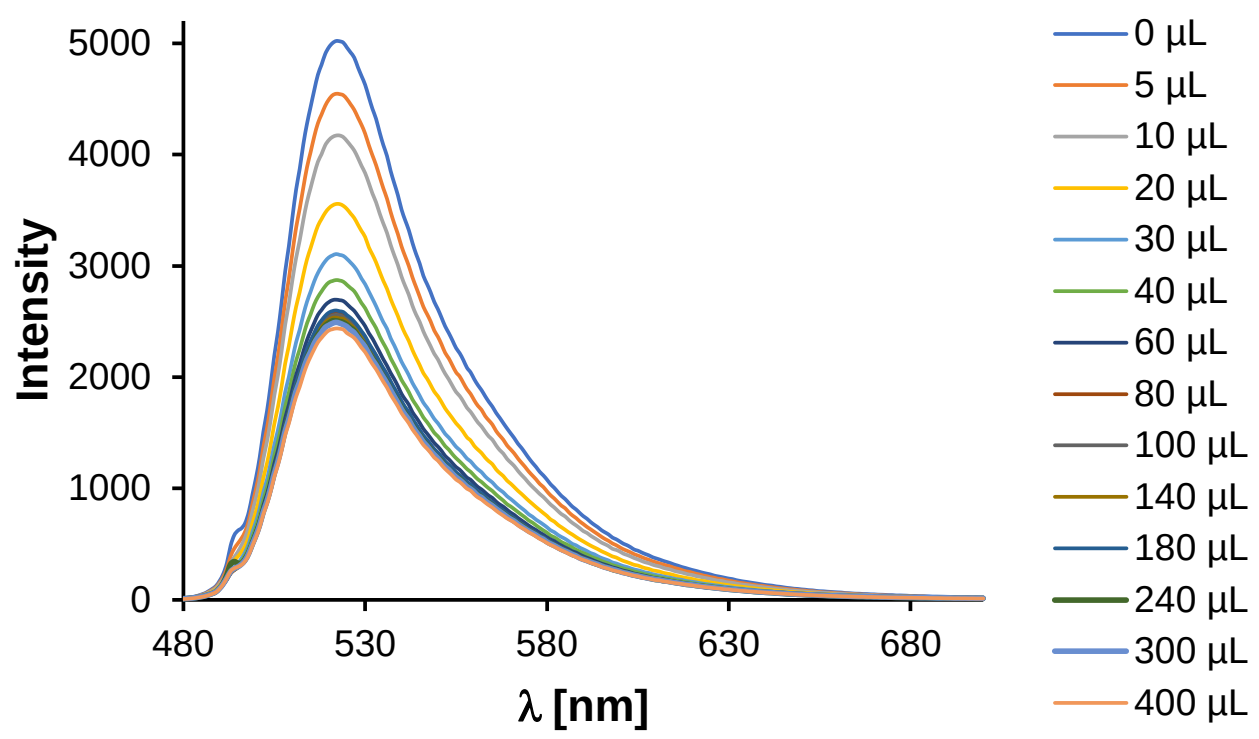

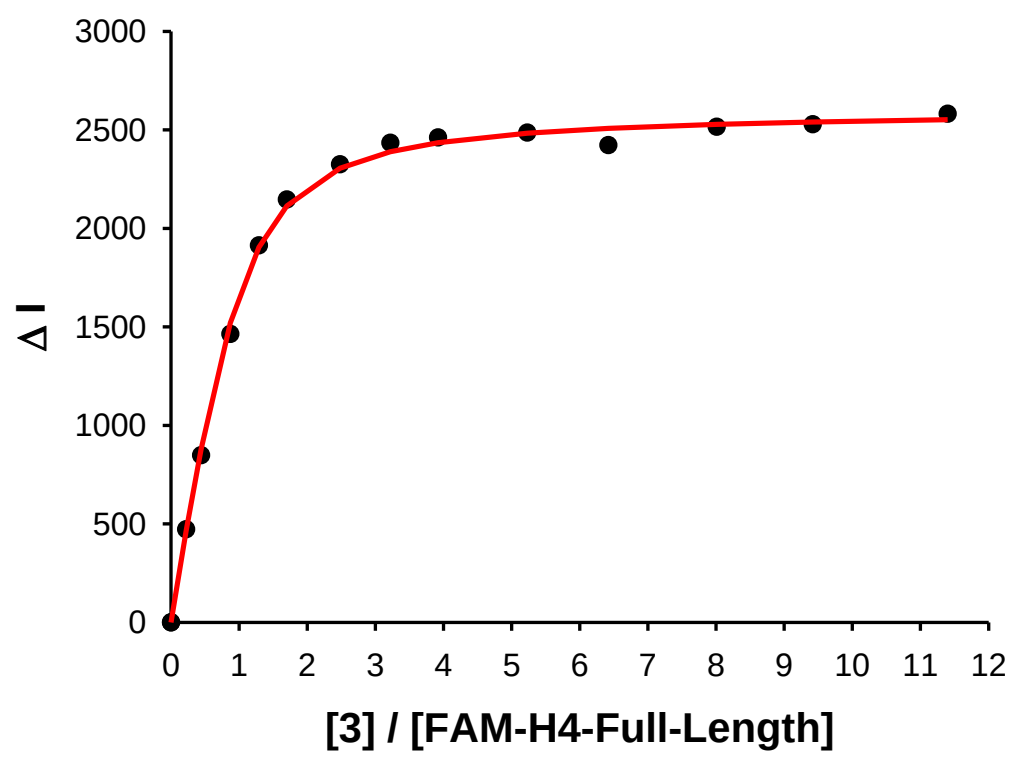

MA model	K_A = 4886700 M^{-1} ± 135380 M^{-1}
	K_D = 205 nM ± 3 %
	ΔI_{max} = 2601.5 ± 6.756

Fluorescence titration of **FAM-H4-Full-Length** with peptide tweezer **4** in KPi buffer (10 mM, pH 7.0)

λ_{ex} = 495 nm	Tweezer	Peptide
λ_{em} = 522 nm	**4**	**FAM-H4-Full-Length**
Amount [mg]:	0.049	0.14
Volume [mL]:	1.15	46.369
Concentration [mol/L]:	3.11E-05	1.00E-06

Tweezer V (µL)	Peptide V (µL)	[Peptide] [mol/L]	[Tweezer] [mol/L]	[Tweezer]/ [Pepitd]	F·I· (I_{522} nm)	ΔI_{obs}	ΔI_{calc}
0	700	1.00E-06	0.00E+00	0.00	4682	0	0
5	705	1.00E-06	2.21E-07	0.22	3790	892	503
10	710	1.00E-06	4.38E-07	0.44	3383	1300	982
20	720	1.00E-06	8.64E-07	0.86	2892	1790	1788
30	730	1.00E-06	1.28E-06	1.28	2626	2056	2165
40	740	1.00E-06	1.68E-06	1.68	2475	2207	2269
60	760	1.00E-06	2.46E-06	2.46	2339	2343	2330
80	780	1.00E-06	3.19E-06	3.19	2340	2343	2350
100	800	1.00E-06	3.89E-06	3.89	2370	2312	2360
140	840	1.00E-06	5.18E-06	5.18	2407	2276	2369
180	880	1.00E-06	6.36E-06	6.36	2404	2278	2374
240	940	1.00E-06	7.94E-06	7.94	2391	2291	2378
300	1000	1.00E-06	9.33E-06	9.33	2218	2465	2380
400	1100	1.00E-06	1.13E-05	11.31	2125	2557	2382

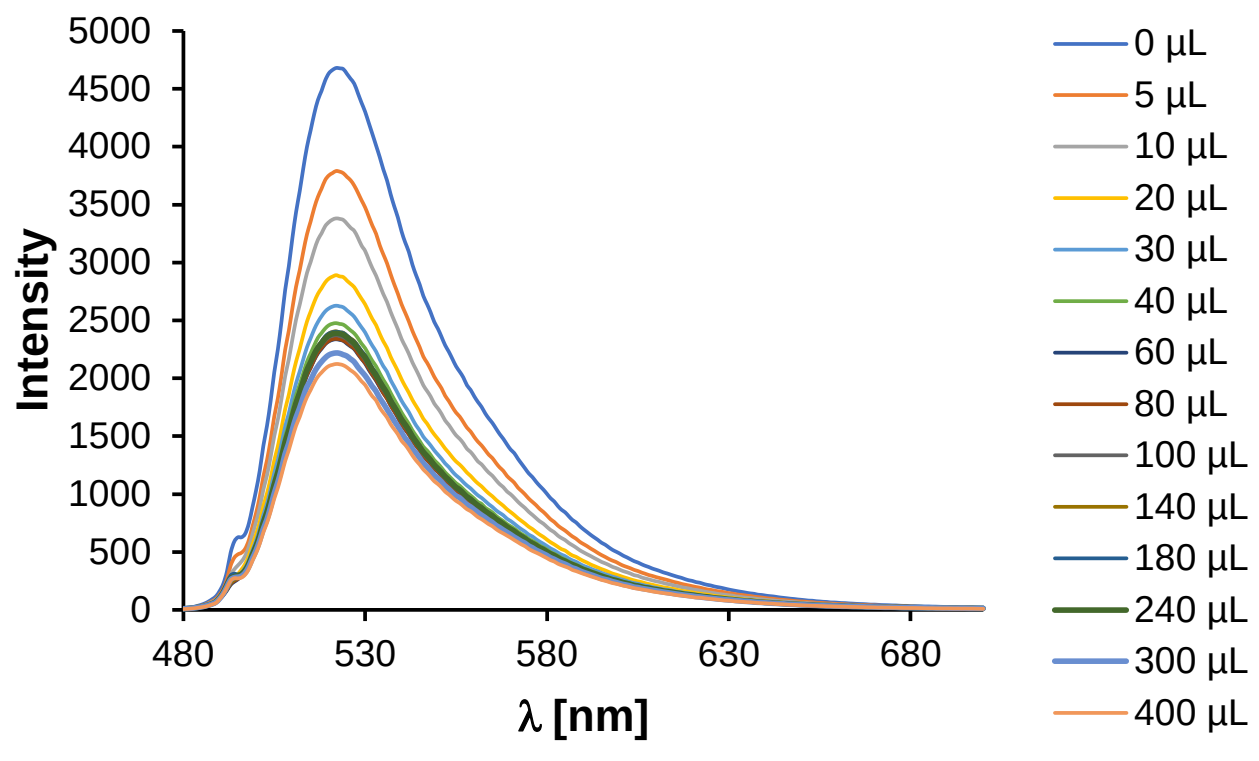

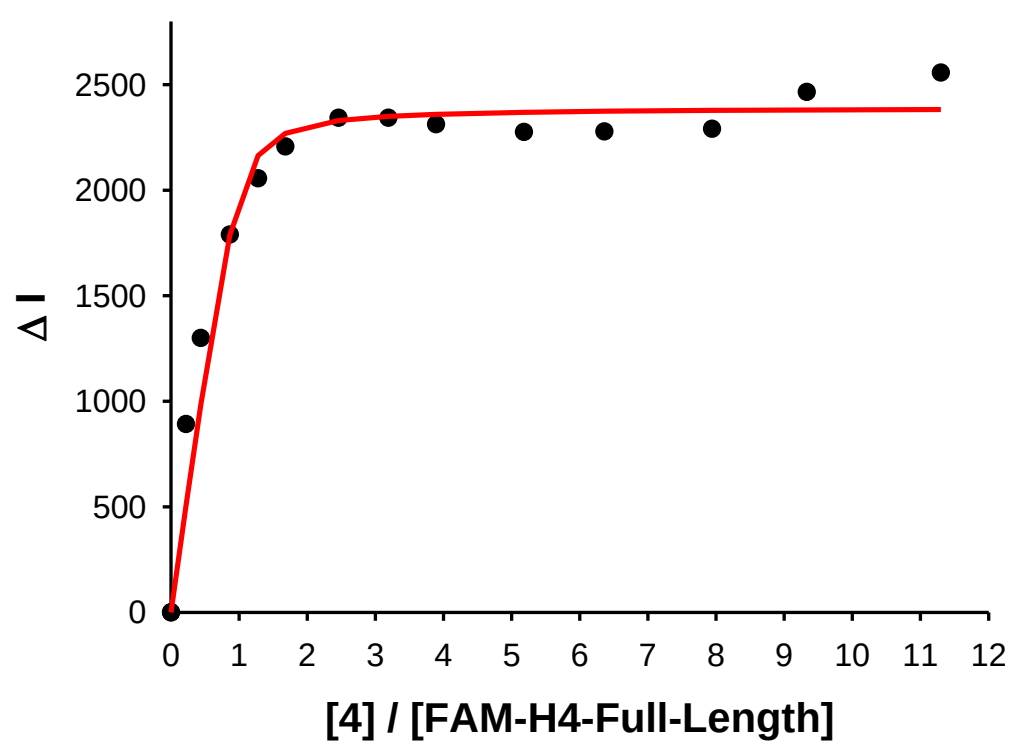

MA model	K_A = 4785400 M^{-1} ± 537880 M^{-1}
	K_D = 209 nM ± 11 %
	ΔI_{max} = 2469.2 ± 18.083

Fluorescence titration of **FAM-H4-Full-Length** with Asp tweezer **5** in KPi buffer (10 mM, pH 7.0)

λ_{ex} = 495 nm	Tweezer	Peptide
λ_{em} = 522 nm	**5**	**FAM-H4-Full-Length**
Amount [mg]:	0.044	0.14
Volume [mL]:	0.625	46.369
Concentration [mol/L]:	3.11E-05	1.00E-06

Tweezer V (µL)	Peptide V (µL)	[Peptide] [mol/L]	[Tweezer] [mol/L]	[Tweezer]/ [Peptide]	F·I· (I_{522} nm)	ΔI_{obs}	ΔI_{calc}
0	700	1.00E-06	0.00E+00	0.00	4790	0	0
5	705	1.00E-06	2.21E-07	0.22	4055	735	705
10	710	1.00E-06	4.39E-07	0.44	3267	1523	1348
20	720	1.00E-06	8.65E-07	0.87	2528	2262	2353
30	730	1.00E-06	1.28E-06	1.28	1925	2865	2922
40	740	1.00E-06	1.68E-06	1.68	1572	3218	3196
60	760	1.00E-06	2.46E-06	2.46	1302	3488	3423
80	780	1.00E-06	3.19E-06	3.19	1263	3527	3513
100	800	1.00E-06	3.89E-06	3.89	1233	3557	3560
140	840	1.00E-06	5.19E-06	5.19	1177	3613	3609
180	880	1.00E-06	6.37E-06	6.37	1160	3630	3634
240	940	1.00E-06	7.95E-06	7.95	1149	3641	3654
300	1000	1.00E-06	9.34E-06	9.34	1138	3652	3666
400	1100	1.00E-06	1.13E-05	11.32	1144	3646	3677

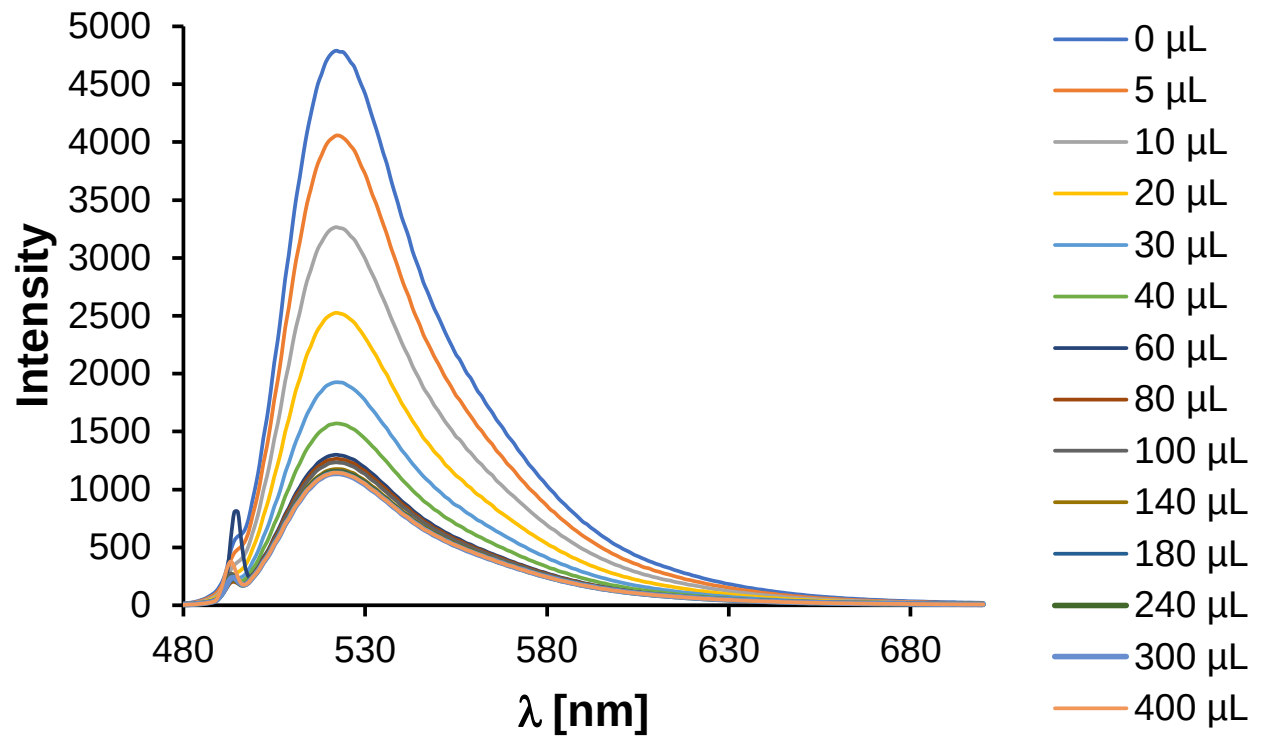

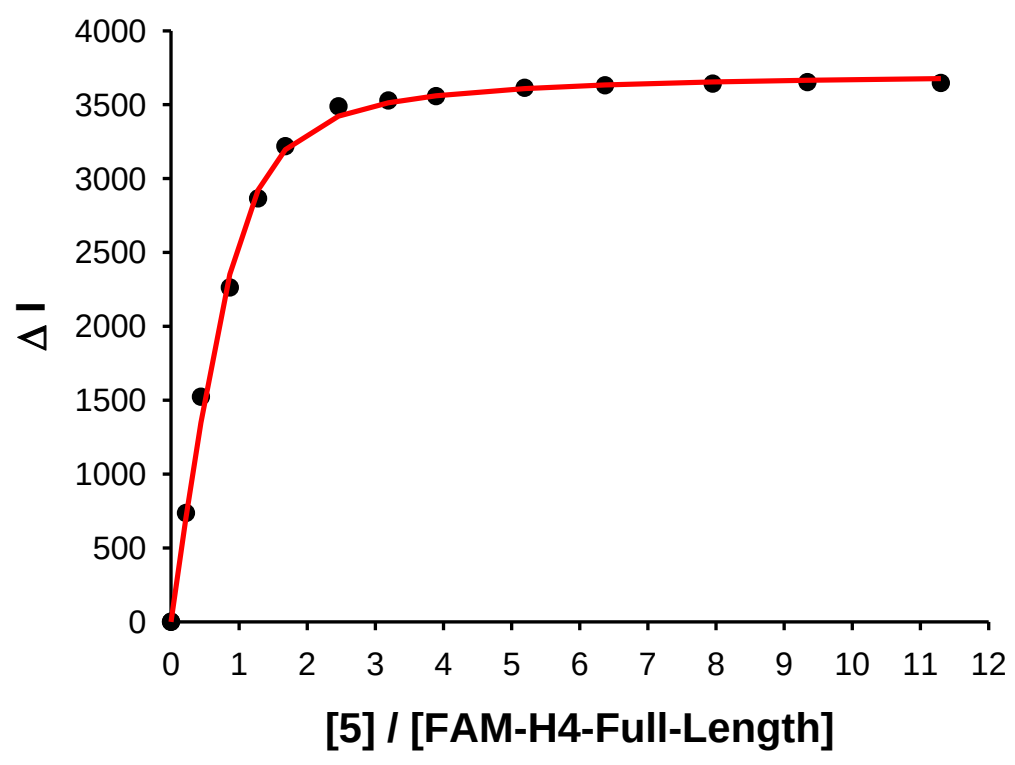

MA model	K_A = 7336000 M^{-1} ± 312690 M^{-1}
	K_D = 136 nM ± 4 %
	ΔI_{max} = 3725.6 ± 5.7336

Fluorescence titration of **FAM-H4-Full-Length** with Asp tweezer **6** in KPi buffer (10 mM, pH 7.0)

λ_{ex} = 495 nm	Tweezer	Peptide
λ_{em} = 522 nm	**6**	**FAM-H4-Full-Length**
Amount [mg]:	0.075	0.087
Volume [mL]:	1.2	28.815
Concentration [mol/L]:	3.15E-05	1.00E-06

Tweezer V (µL)	Peptide V (µL)	[Peptide] [mol/L]	[Tweezer] [mol/L]	[Tweezer]/ [Peptide]	F.I. (I_{522} nm)	ΔI_{obs}	ΔI_{calc}
0	700	1.00E-06	0.00E+00	0.00	4468	0	0
5	705	1.00E-06	2.23E-07	0.22	3660	808	867
10	710	1.00E-06	4.43E-07	0.44	2787	1682	1676
20	720	1.00E-06	8.74E-07	0.87	1526	2942	2970
30	730	1.00E-06	1.29E-06	1.29	861	3608	3627
40	740	1.00E-06	1.70E-06	1.70	552	3916	3885
60	760	1.00E-06	2.48E-06	2.48	369	4100	4061
80	780	1.00E-06	3.23E-06	3.23	311	4157	4126
100	800	1.00E-06	3.93E-06	3.93	271	4197	4158
140	840	1.00E-06	5.24E-06	5.24	264	4204	4191
180	880	1.00E-06	6.43E-06	6.43	261	4207	4208
240	940	1.00E-06	8.03E-06	8.03	264	4204	4221
300	1000	1.00E-06	9.44E-06	9.44	269	4199	4229
400	1100	1.00E-06	1.14E-05	11.44	286	4182	4236

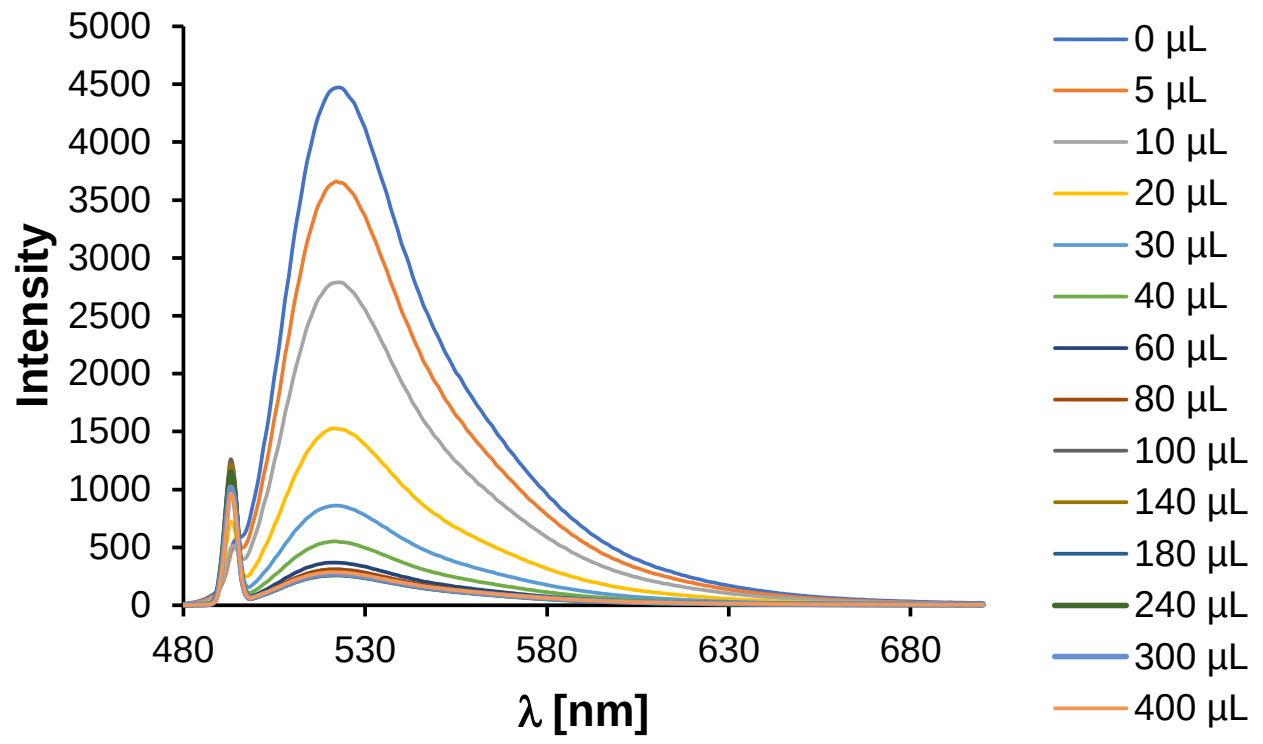

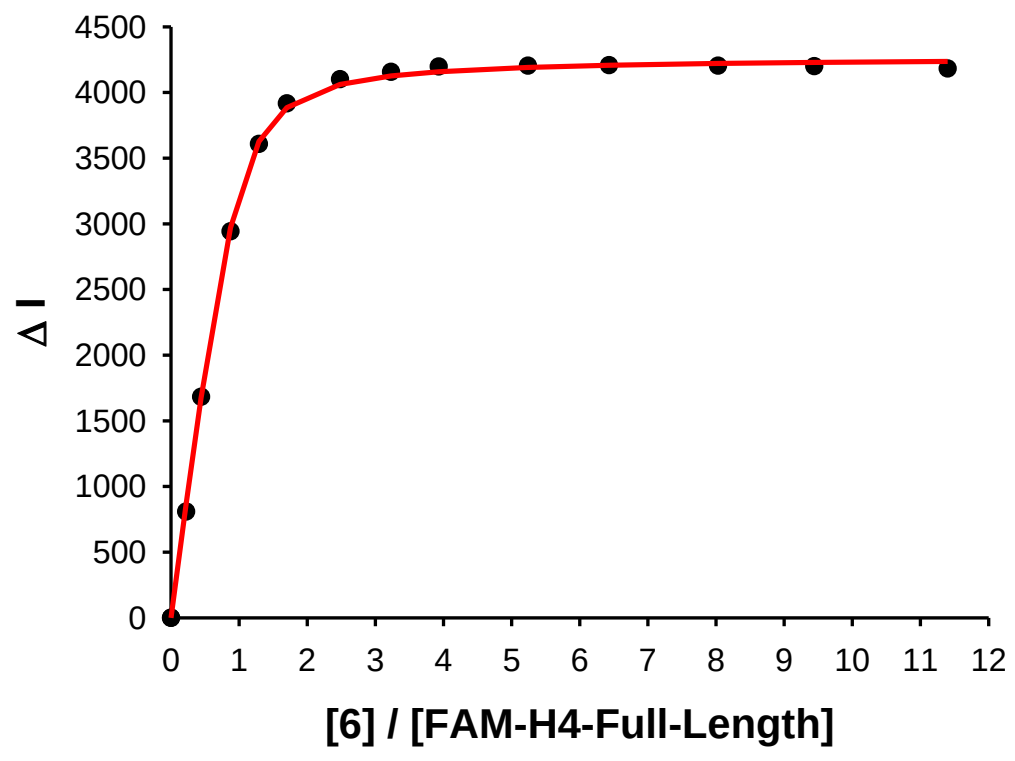

MA model	K_A = 12852000 M^{-1} ± 247750 M^{-1}
	K_D = 78 nM ± 2 %
	ΔI_{max} = 4267.8 ± 4.6581

Fluorescence titration of **FAM-H4-Full-Length** with bisphosphonate tweezer **7** in KPi buffer (10 mM, pH 7.0)

λ_{ex} = 285 nm	Tweezer	Peptide
λ_{em} = 323 nm	**7**	**FAM-H4-Full-Length**
Amount [mg]:	0.075	0.14
Volume [mL]:	1.03	46.369
Concentration [mol/L]:	3.10E-05	1.00E-06

Tweezer V (µL)	Peptide V (µL)	[Peptide] [mol/L]	[Tweezer] [mol/L]	[Tweezer]/ [Peptide]	F·I· (I_{522} nm)	ΔI_{obs}	ΔI_{calc}
0	700	1.00E-06	0.00E+00	0.00	5176	0	0
5	705	1.00E-06	2.20E-07	0.22	4024	1152	744
10	710	1.00E-06	4.36E-07	0.44	3657	1520	1402
20	720	1.00E-06	8.60E-07	0.86	2719	2458	2427
30	730	1.00E-06	1.27E-06	1.27	2128	3048	3062
40	740	1.00E-06	1.67E-06	1.67	1794	3383	3432
60	760	1.00E-06	2.45E-06	2.45	1488	3688	3798
80	780	1.00E-06	3.18E-06	3.18	1305	3872	3962
100	800	1.00E-06	3.87E-06	3.87	1183	3994	4053
140	840	1.00E-06	5.16E-06	5.16	1026	4151	4150
180	880	1.00E-06	6.34E-06	6.34	960	4216	4201
240	940	1.00E-06	7.91E-06	7.91	902	4274	4243
300	1000	1.00E-06	9.29E-06	9.29	860	4317	4268
400	1100	1.00E-06	1.13E-05	11.26	822	4355	4292

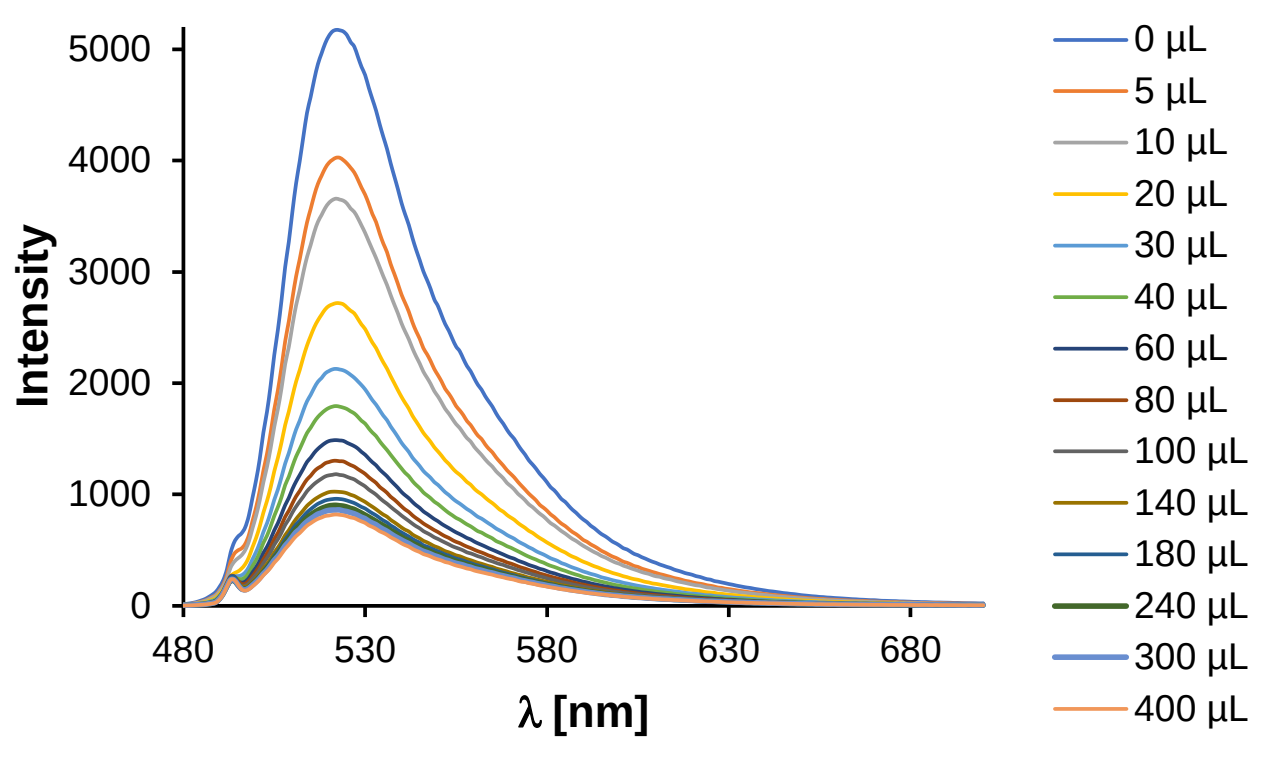

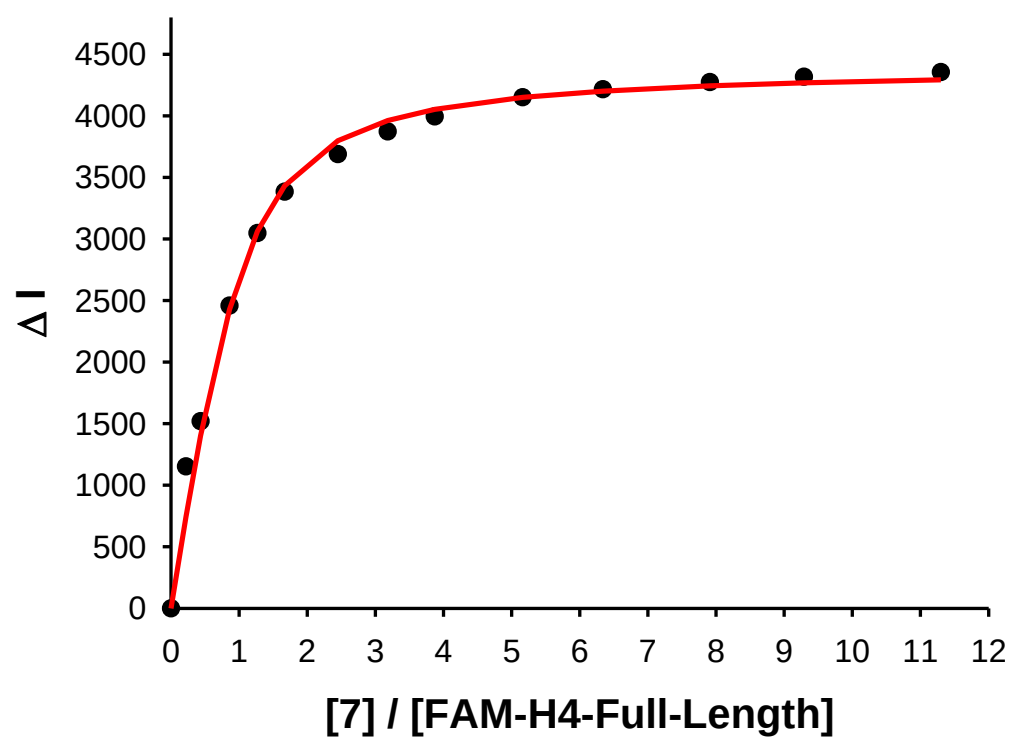

MA model

K_A = 4002600 M^{-1} ± 99544 M^{-1}

K_D = 250 nM ± 2 %

ΔI_{max} = 4396 ± 9.4908

Fluorescence titration of **FAM-H4-Full-Length** with nitriloacetate tweezer **8** in KPi buffer (10 mM, pH 7.0)

Volume [mL]: λ_{em} = 524 nm	Tweezer **8**	Peptide **FAM-H4-Full-Length**
Amount [mg]:	0.076	0.14
Volume [mL]:	1.07	46.369
Concentration [mol/L]:	3.11E-05	1.00E-06

Tweezer V (µL)	Peptide V (µL)	[Peptide] [mol/L]	[Tweezer] [mol/L]	[Tweezer]/ [Peptide]	F·I· (I_{524} nm)	ΔI_{obs}	ΔI_{calc}
0	700	1.00E-06	0.00E+00	0.00	4743	0	0
5	705	1.00E-06	2.21E-07	0.22	3918	826	739
10	710	1.00E-06	4.38E-07	0.44	3177	1566	1435
20	720	1.00E-06	8.64E-07	0.86	2106	2637	2583
30	730	1.00E-06	1.28E-06	1.28	1677	3066	3147
40	740	1.00E-06	1.68E-06	1.68	1463	3280	3332
60	760	1.00E-06	2.46E-06	2.46	1291	3453	3448
80	780	1.00E-06	3.19E-06	3.19	1254	3489	3488
100	800	1.00E-06	3.89E-06	3.89	1235	3508	3508
140	840	1.00E-06	5.18E-06	5.18	1239	3505	3527
180	880	1.00E-06	6.36E-06	6.36	1215	3529	3537
240	940	1.00E-06	7.94E-06	7.94	1204	3539	3545
300	1000	1.00E-06	9.33E-06	9.33	1182	3561	3550
400	1100	1.00E-06	1.13E-05	11.31	1160	3584	3554

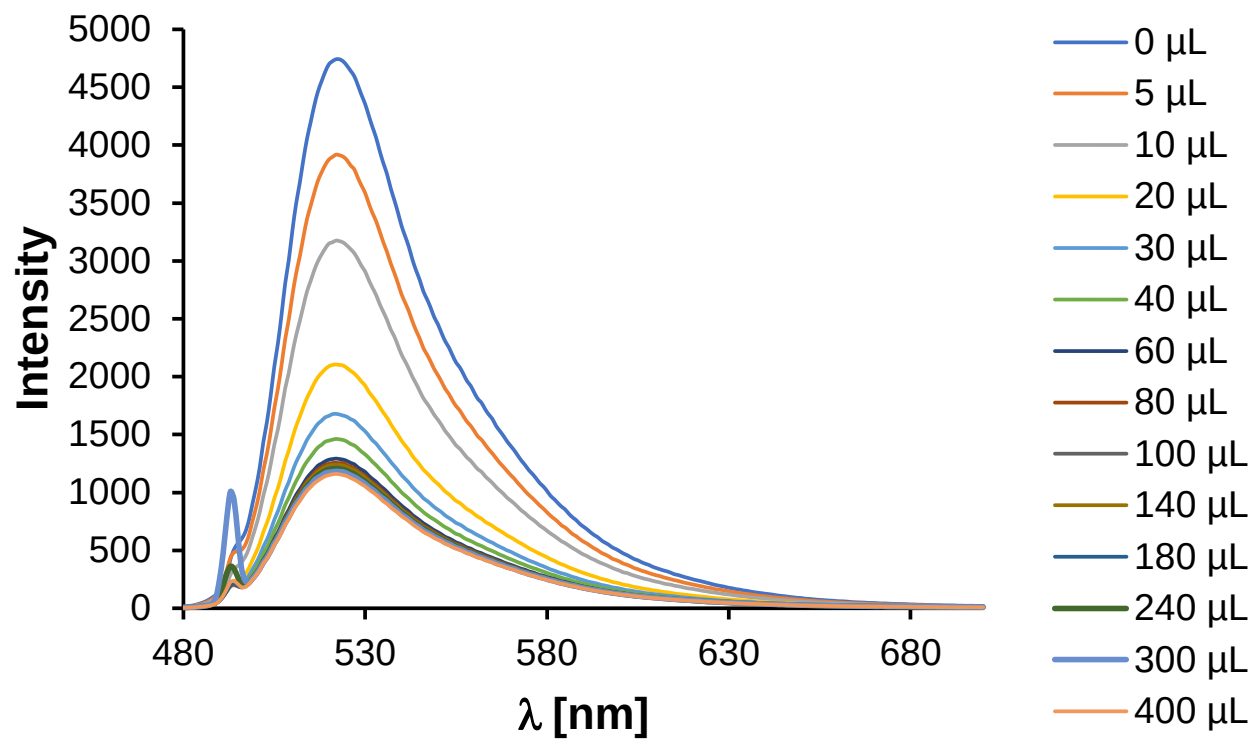

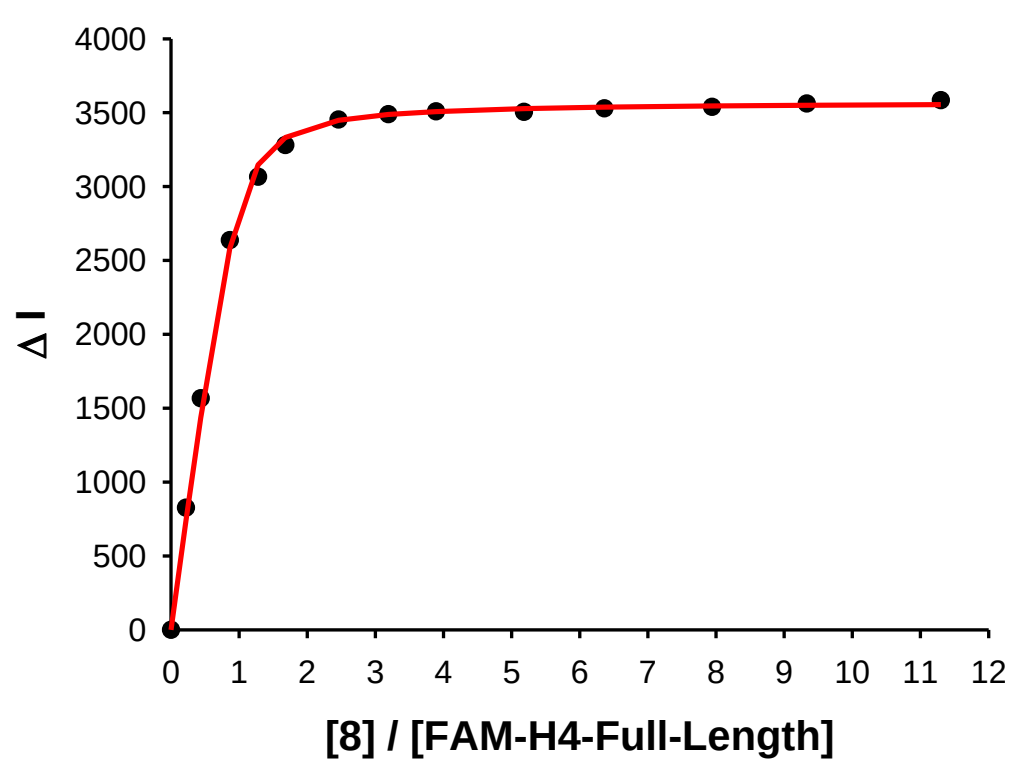

MA model	K_A = 18494000 M^{-1} ± 1332700 M^{-1}
	K_D = 54 nM ± 7 %
	ΔI_{max} = 3572.9 ± 4.1524

Fluorescence titration of **FAM-H4-Full-Length** with distilbene disulfonate tweezer **9** in KPi buffer (10 mM, pH 7.0)

λ_{ex} = 495 nm	Tweezer	Peptide
λ_{em} = 524 nm	**9**	**FAM-H4-Full-Length**
Amount [mg]:	0.046	0.084
Volume [mL]:	0.68	27.822
Concentration [mol/L]:	3.14E-05	1.00E-06

Tweezer V (µL)	Peptide V (µL)	[Peptide] [mol/L]	[Tweezer] [mol/L]	[Tweezer]/ [Peptide]	F·I· (I_{524} nm)	ΔI_{obs}	ΔI_{calc}
0	700	1.00E-06	0.00E+00	0.00	4685	0	0
5	705	1.00E-06	2.23E-07	0.22	3855	830	807
10	710	1.00E-06	4.42E-07	0.44	3022	1663	1584
20	720	1.00E-06	8.72E-07	0.87	1883	2802	2932
30	730	1.00E-06	1.29E-06	1.29	1217	3468	3497
40	740	1.00E-06	1.70E-06	1.70	925	3760	3613
60	760	1.00E-06	2.48E-06	2.48	823	3862	3671
80	780	1.00E-06	3.22E-06	3.22	870	3815	3689
100	800	1.00E-06	3.92E-06	3.92	925	3759	3698
140	840	1.00E-06	5.23E-06	5.23	994	3691	3707
180	880	1.00E-06	6.42E-06	6.42	1054	3631	3711
240	940	1.00E-06	8.01E-06	8.01	1110	3575	3715
300	1000	1.00E-06	9.41E-06	9.41	1064	3621	3717
400	1100	1.00E-06	1.14E-05	11.41	1059	3626	3719

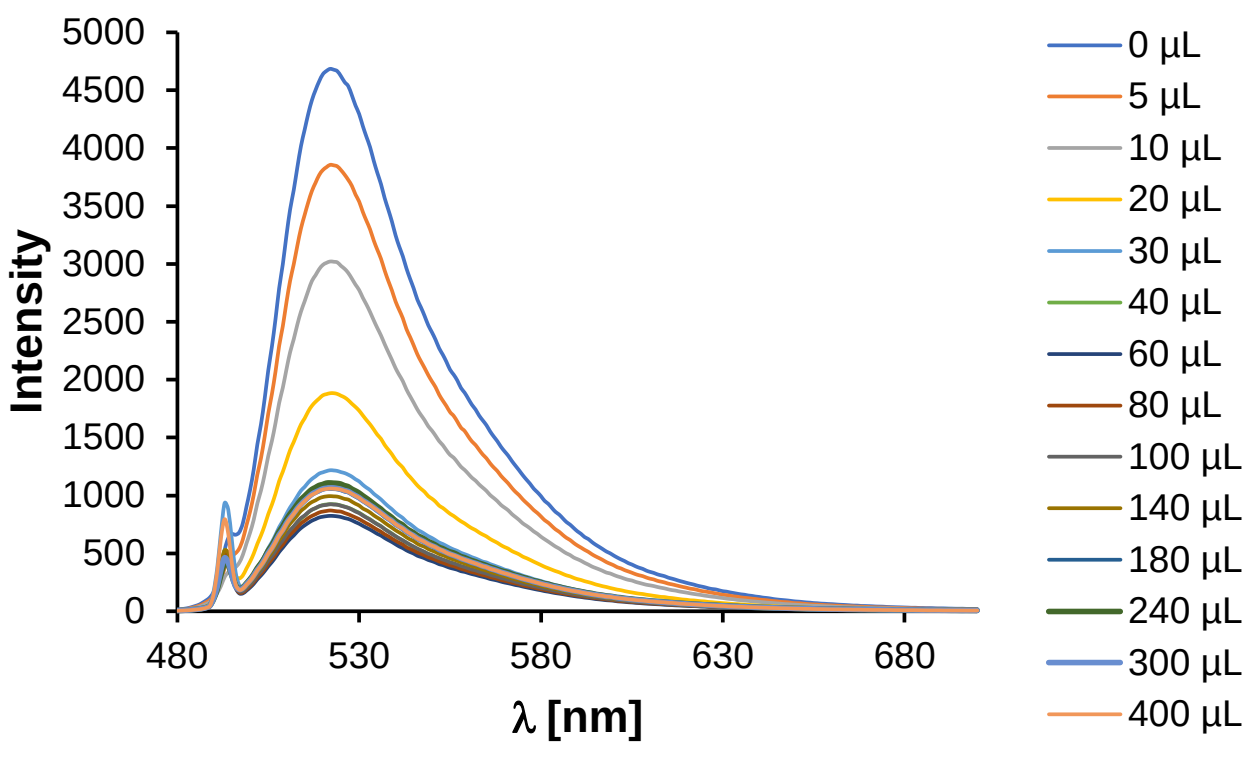

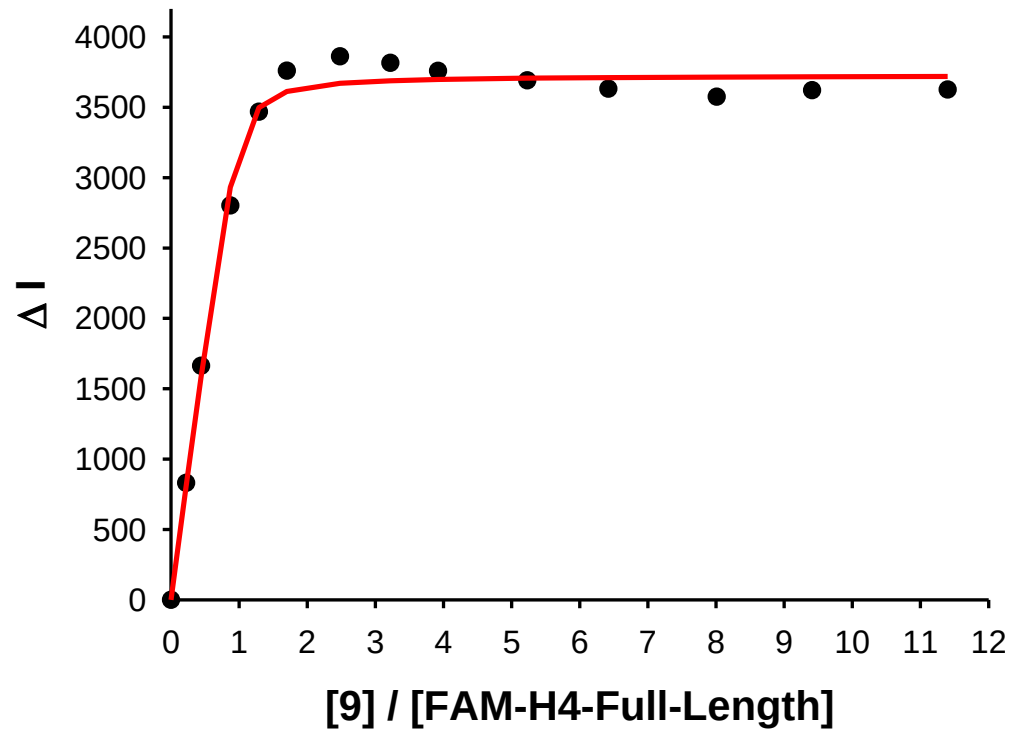

**MA model**	K_A = 43264000 M^{-1} ± 13971000 M^{-1}
	K_D = 23 nM ± 32 %
	ΔI_{max} = 3727.3 ± 13.259

Fluorescence titration of **FAM-H4-Full-Length** with PEG2 tweezer **10** in KPi buffer (10 mM, pH 7.0)

λ_{ex} = 495 nm	Tweezer	Peptide
λ_{em} = 524 nm	**10**	**FAM-H4-Full-Length**
Amount [mg]:	0.061	0.14
Volume [mL]:	1.04	46.369
Concentration [mol/L]:	3.10E-05	1.00E-06

Tweezer V (µL)	Peptide V (µL)	[Peptide] [mol/L]	[Tweezer] [mol/L]	[Tweezer]/ [Peptide]	F·I· (I_{524} nm)	ΔI_{obs}	ΔI_{calc}
0	700	1.00E-06	0.00E+00	0.00	4764	0	0
5	705	1.00E-06	2.20E-07	0.22	4098	666	906
10	710	1.00E-06	4.37E-07	0.44	3078	1686	1754
20	720	1.00E-06	8.62E-07	0.86	1896	2868	3121
30	730	1.00E-06	1.28E-06	1.28	879	3885	3835
40	740	1.00E-06	1.68E-06	1.68	412	4352	4106
60	760	1.00E-06	2.45E-06	2.45	339	4426	4292
80	780	1.00E-06	3.18E-06	3.18	316	4448	4360
100	800	1.00E-06	3.88E-06	3.88	307	4457	4395
140	840	1.00E-06	5.17E-06	5.17	331	4433	4429
180	880	1.00E-06	6.35E-06	6.35	345	4419	4446
240	940	1.00E-06	7.93E-06	7.93	367	4398	4460
300	1000	1.00E-06	9.31E-06	9.31	385	4379	4468
400	1100	1.00E-06	1.13E-05	11.29	407	4357	4476

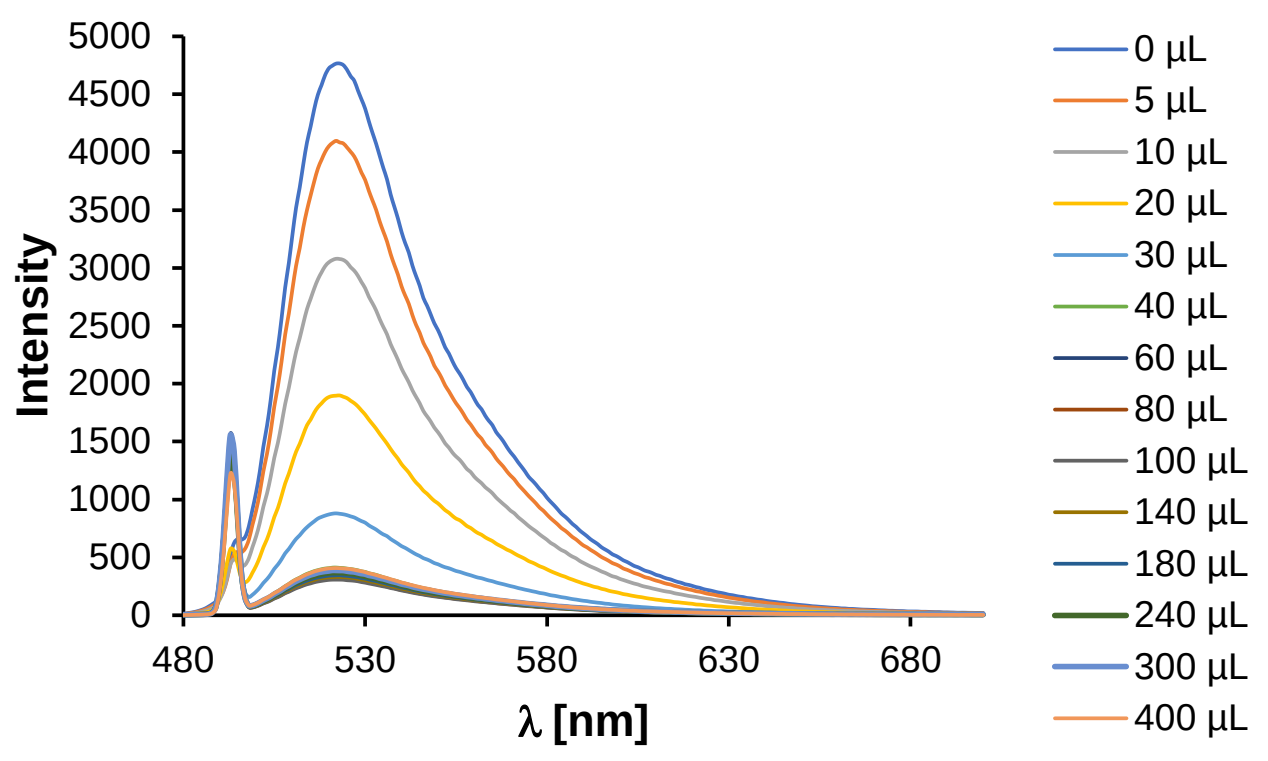

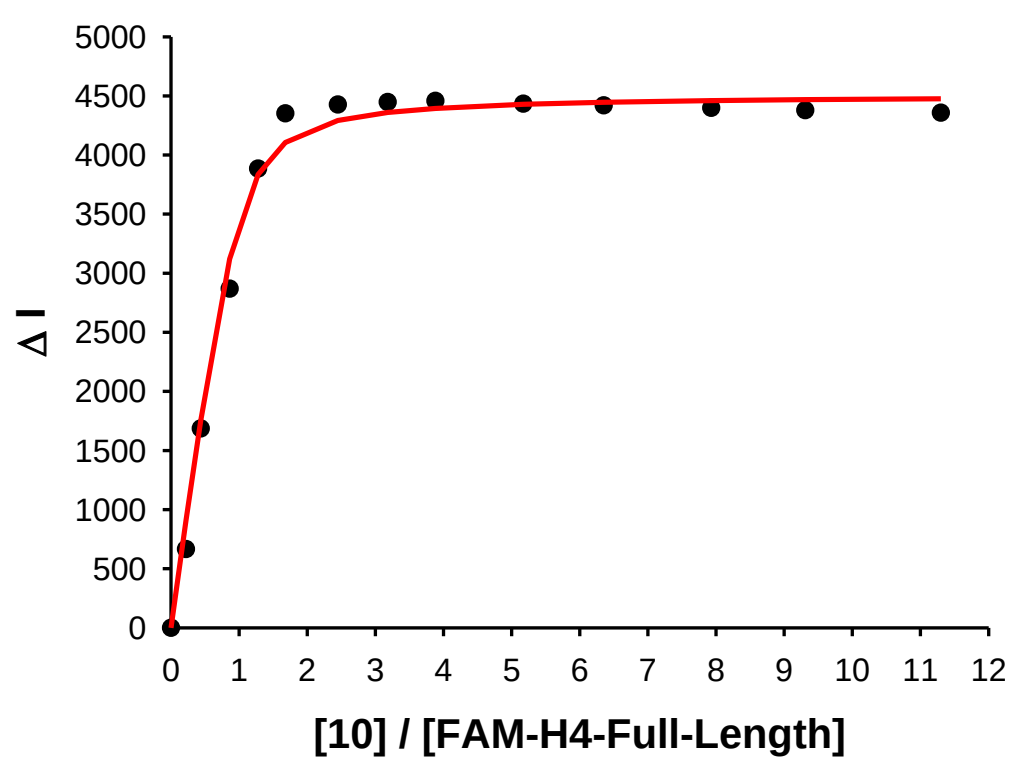

MA model	K_A = 13242000 M^{-1} ± 1988600 M^{-1}
	K_D = 76 nM ± 15 %
	ΔI_{max} = 4508.7 ± 14.722

Fluorescence titration of **FAM-H4-Full-Length** with diphenylsulfone tweezer **11** in KPi buffer (10 mM, pH 7.0)

λ_{ex} = 495 nm<br>λ_{em} = 524 nm	Tweezer<br>**11**	Peptide<br>**FAM-H4-Full-Length**
Amount [mg]:	0.058	0.084
Volume [mL]:	0.93	27.822
Concentration [mol/L]:	3.13E-05	1.00E-06

Tweezer V (μL)	Peptide V (μL)	[Peptide] [mol/L]	[Tweezer] [mol/L]	[Tweezer]/ [Peptide]	F·I· (I_{524} nm)	ΔI_{obs}	ΔI_{calc}
0	700	1.00E-06	0.00E+00	0.00	4819	0	0
5	705	1.00E-06	2.22E-07	0.22	3465	1354	1008
10	710	1.00E-06	4.41E-07	0.44	2693	2126	1981
20	720	1.00E-06	8.71E-07	0.87	1306	3512	3673
30	730	1.00E-06	1.29E-06	1.29	362	4456	4385
40	740	1.00E-06	1.69E-06	1.69	247	4572	4528
60	760	1.00E-06	2.47E-06	2.47	199	4619	4601
80	780	1.00E-06	3.21E-06	3.21	190	4628	4625
100	800	1.00E-06	3.92E-06	3.92	180	4638	4636
140	840	1.00E-06	5.22E-06	5.22	181	4637	4648
180	880	1.00E-06	6.41E-06	6.41	184	4635	4653
240	940	1.00E-06	8.00E-06	8.00	189	4630	4658
300	1000	1.00E-06	9.40E-06	9.40	194	4625	4660
400	1100	1.00E-06	1.14E-05	11.40	205	4613	4663

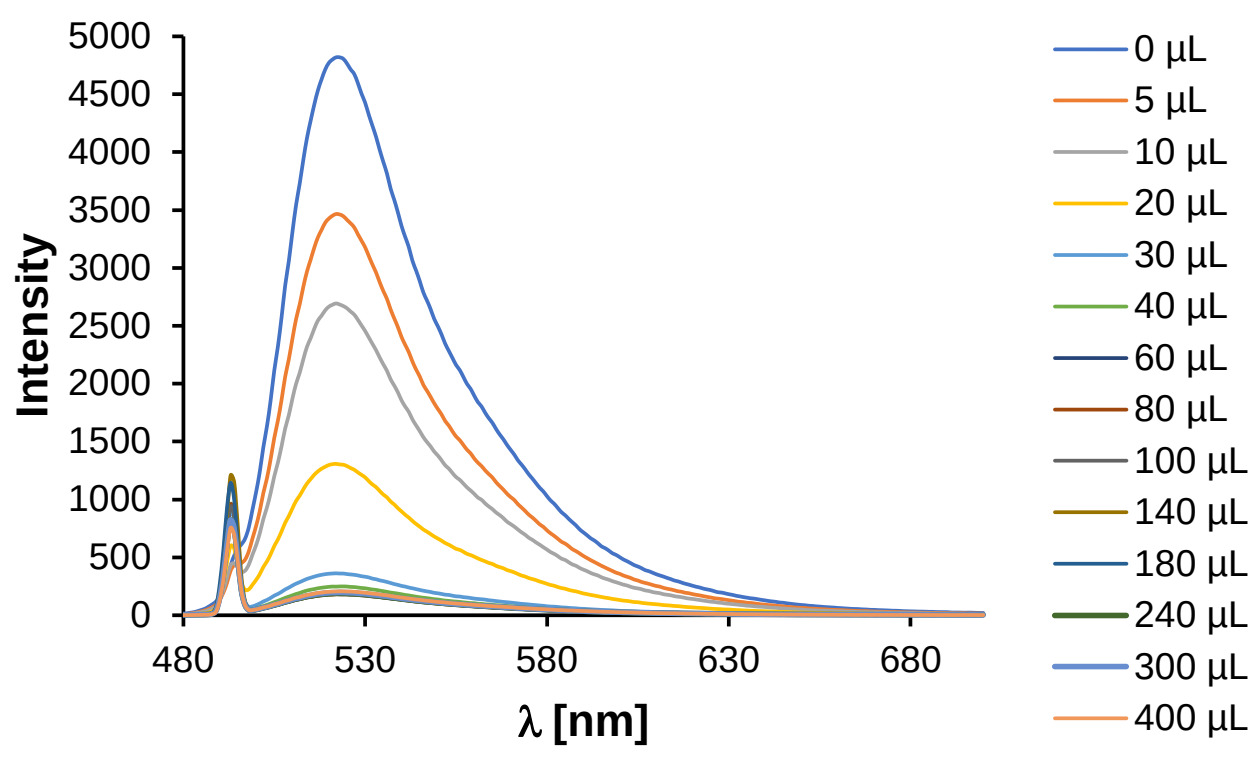

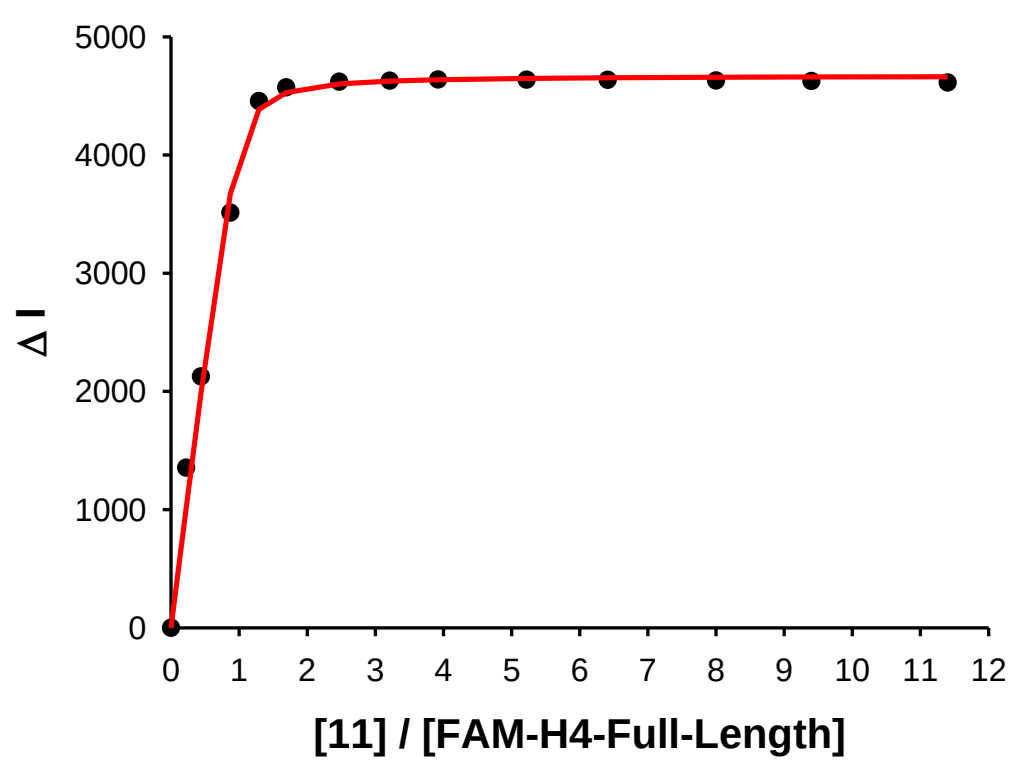

MA model	K_A = 43279000 M^{-1} ± 15563000 M^{-1}<br>K_D = 23 nM ± 36 %<br>ΔI_{max} = 4673 ± 7.6498

Job plot method

Table S1: Pipetting scheme for a job plot experiment. The total volume and concentration in the cuvette were kept constant by removing a certain amount of the cuvette solution, followed by the addition of the tweezer solution**.**

No	Removal from cuvette [µL]	Addition to cuvette [µL]	FAM-peptide [µM]	Tweezer [µM]	Total concentration [µM]	x (FAM-peptide)
1	0	0	10	0	10	1
2	60	60	9	1	10	0.9
3	66.7	66.7	8	2	10	0.8
4	75	75	7	3	10	0.7
5	85.7	85.7	6	4	10	0.6
6	100	100	5	5	10	0.5
7	120	120	4	6	10	0.4
8	150	150	3	7	10	0.3
9	200	200	2	8	10	0.2
10	300	300	1	9	10	0.1
11	600	600	0	10	10	0

Table S2: Mole fractions from Job plots for complex formation between advanced tweezers and FAM peptides. Complex stoichiometry was determined from the position of the curve maximum.

Tweezer	x (FAM-H4-P1)	x (FAM-H4-Full)
**CLR01**	0.45	0.39
**2**	0.45	0.43
**3**	0.45	0.42
**4**	0.47	0.42
**5**	0.46	0.42
**6**	0.42	0.42
**7**	0.5	0.5
**8**	0.5	0.5
**9**	0.5	0.42
**10**	0.48	0.4
**11**	0.49	0.42

Job Plot method with FAM-H4-P1

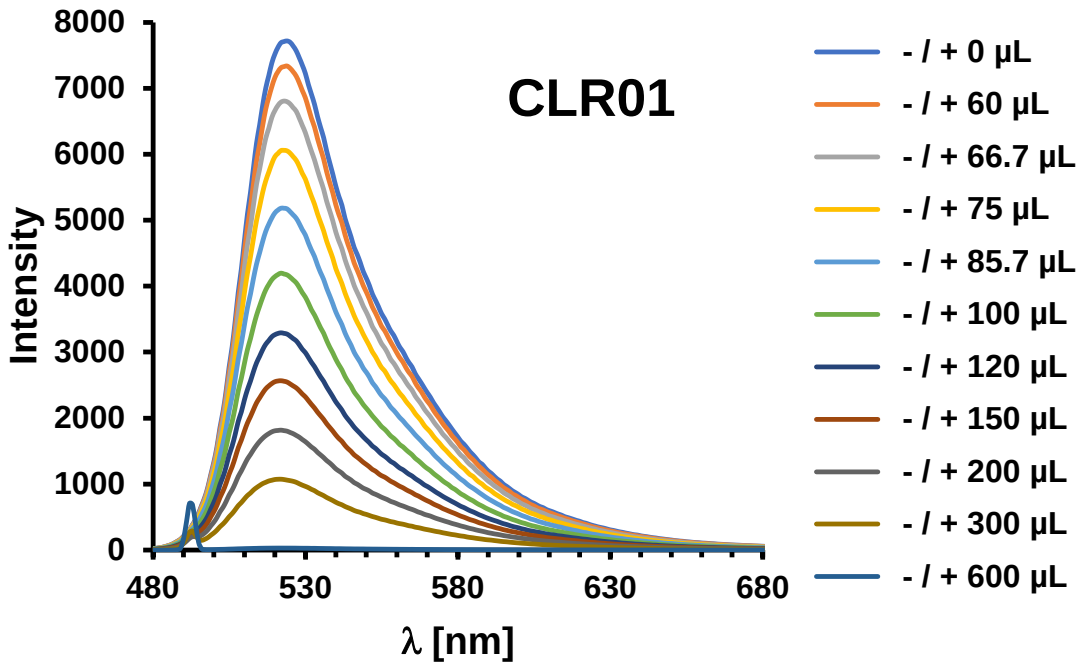

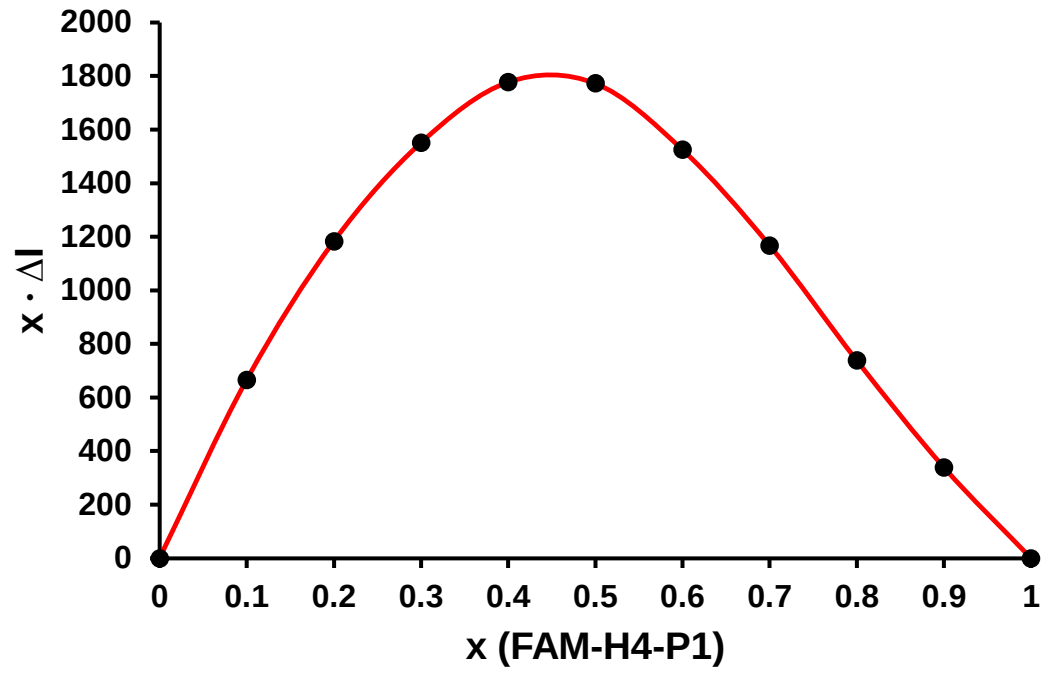

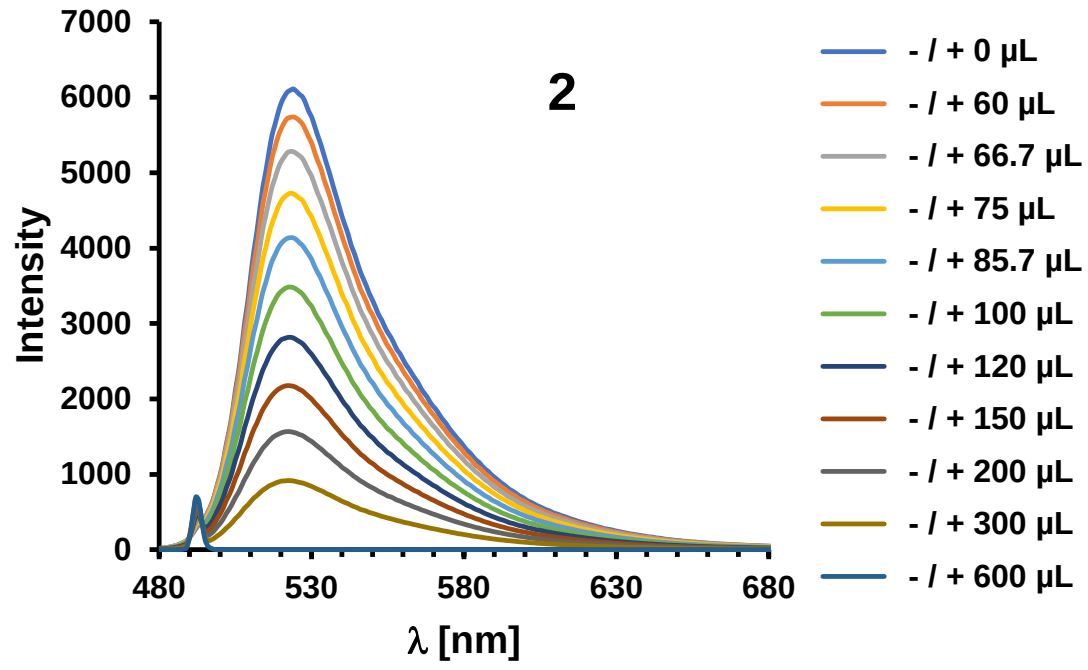

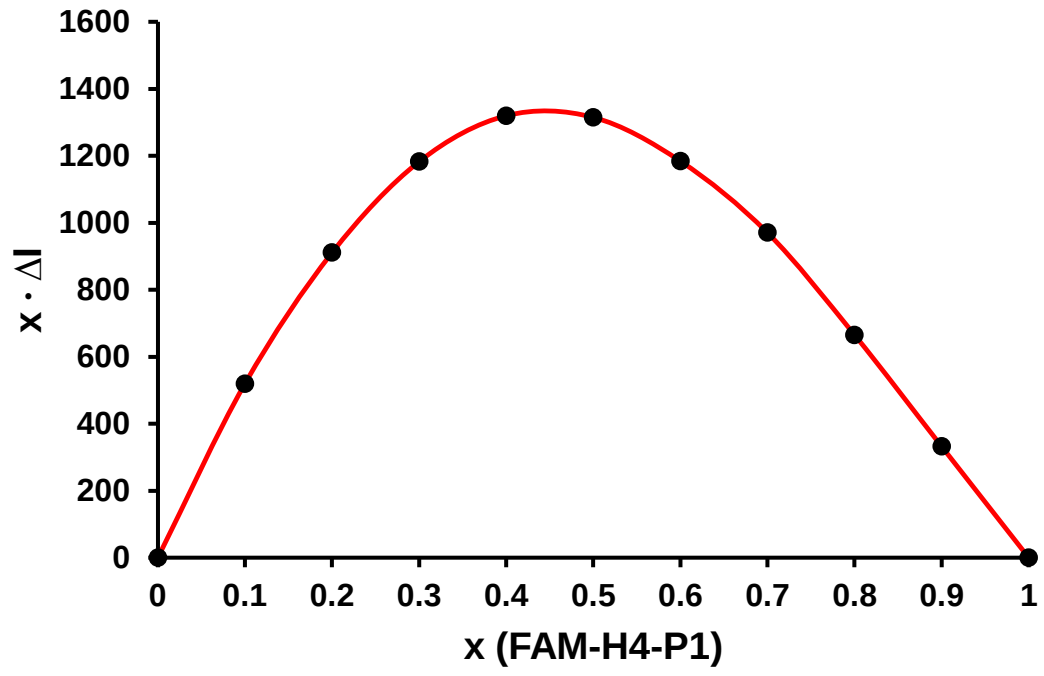

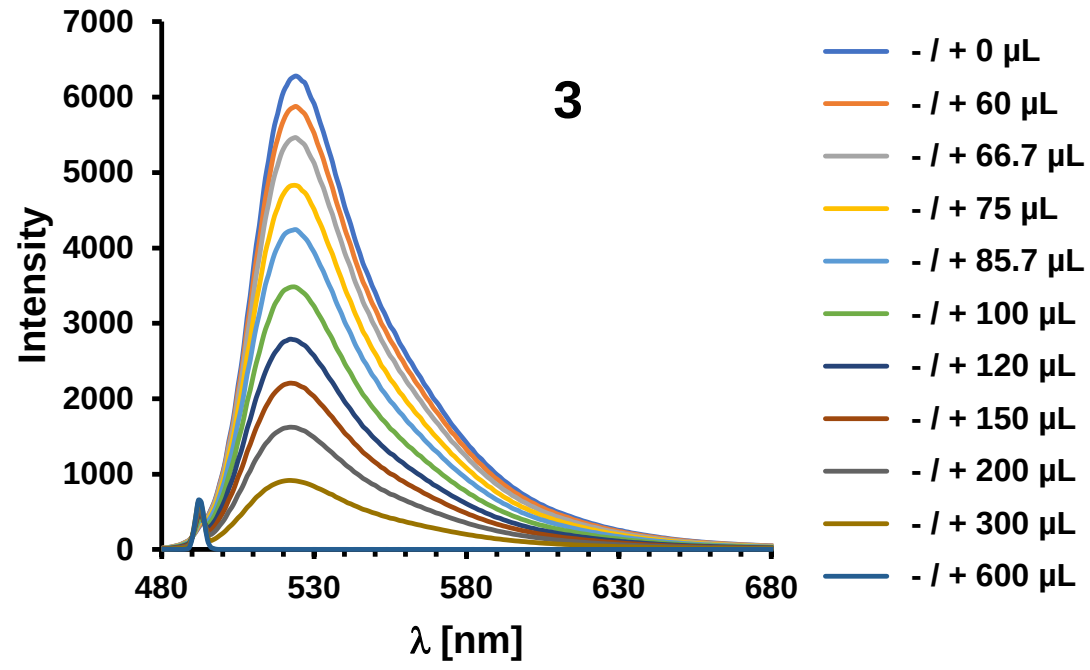

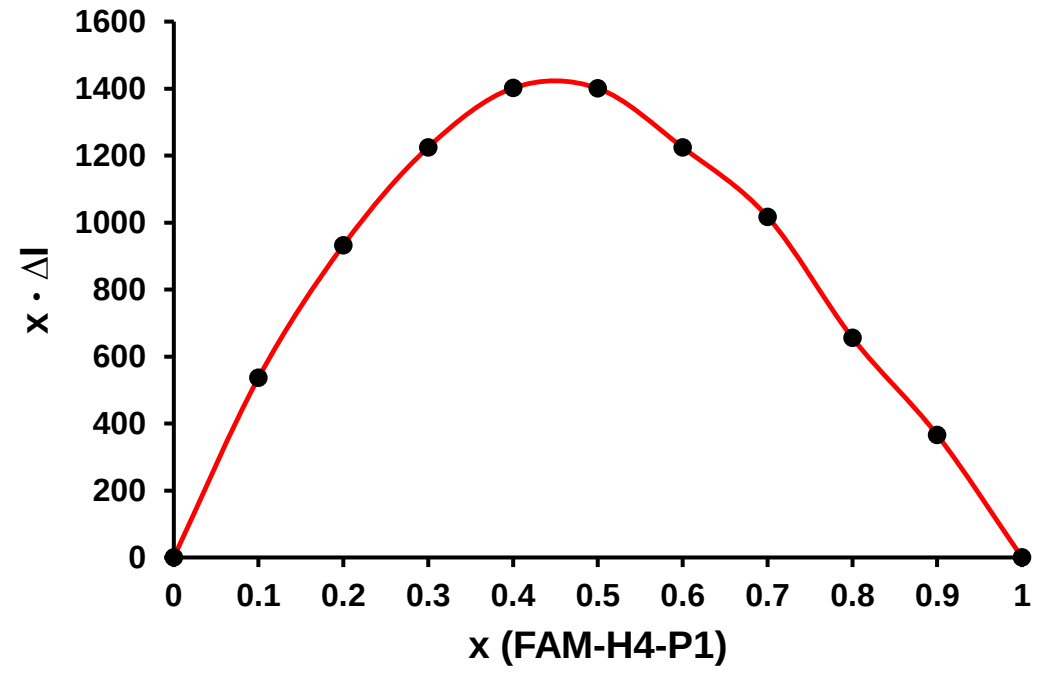

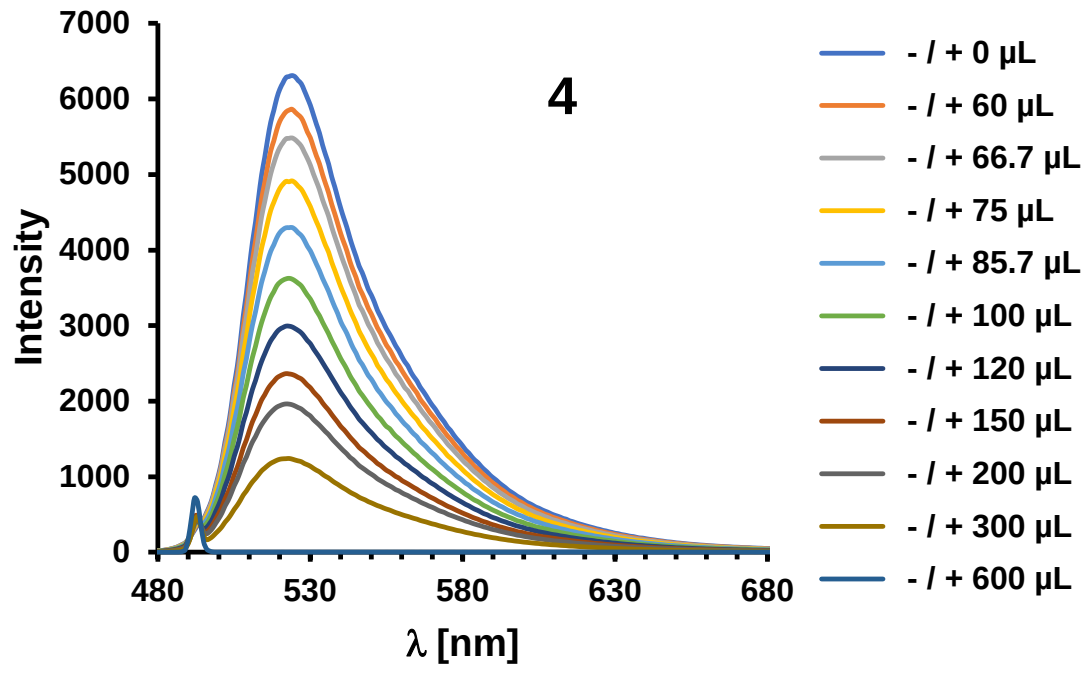

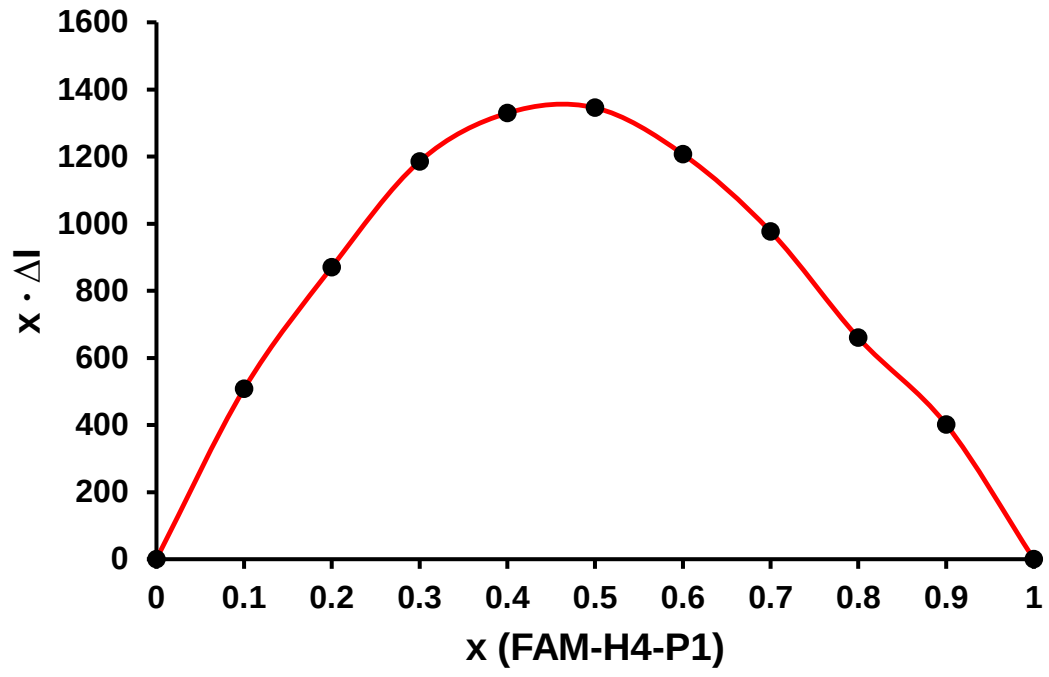

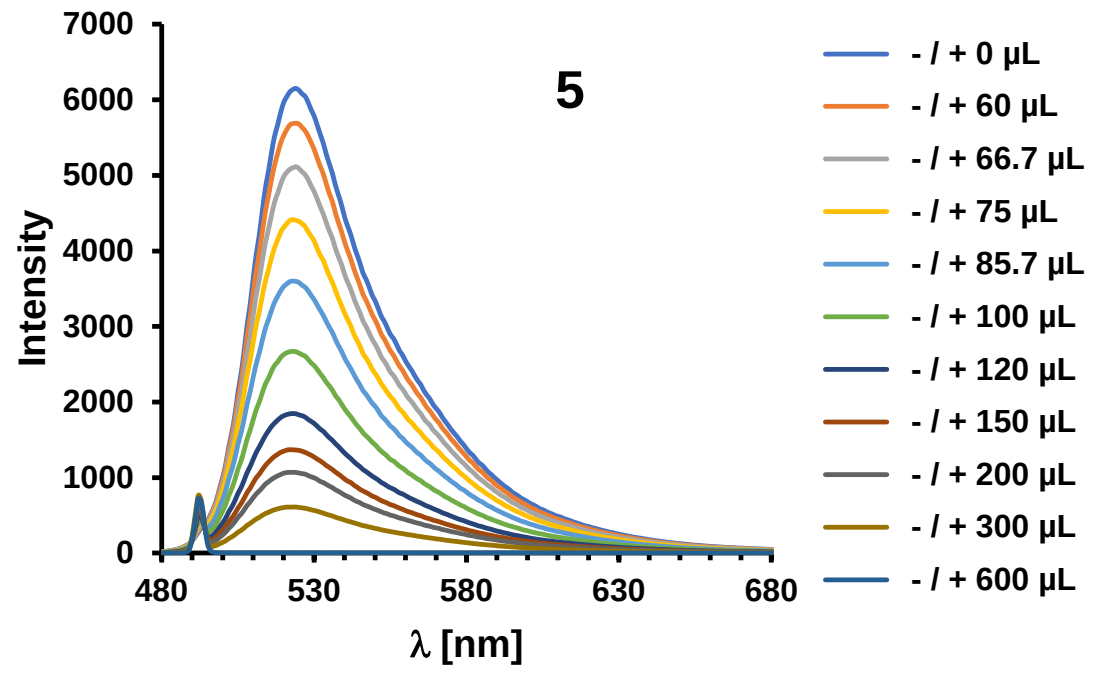
5
Intensity
λ [nm]
- / + 0 µL
- / + 60 µL
- / + 66.7 µL
- / + 75 µL
- / + 85.7 µL
- / + 100 µL
- / + 120 µL
- / + 150 µL
- / + 200 µL
- / + 300 µL
- / + 600 µL

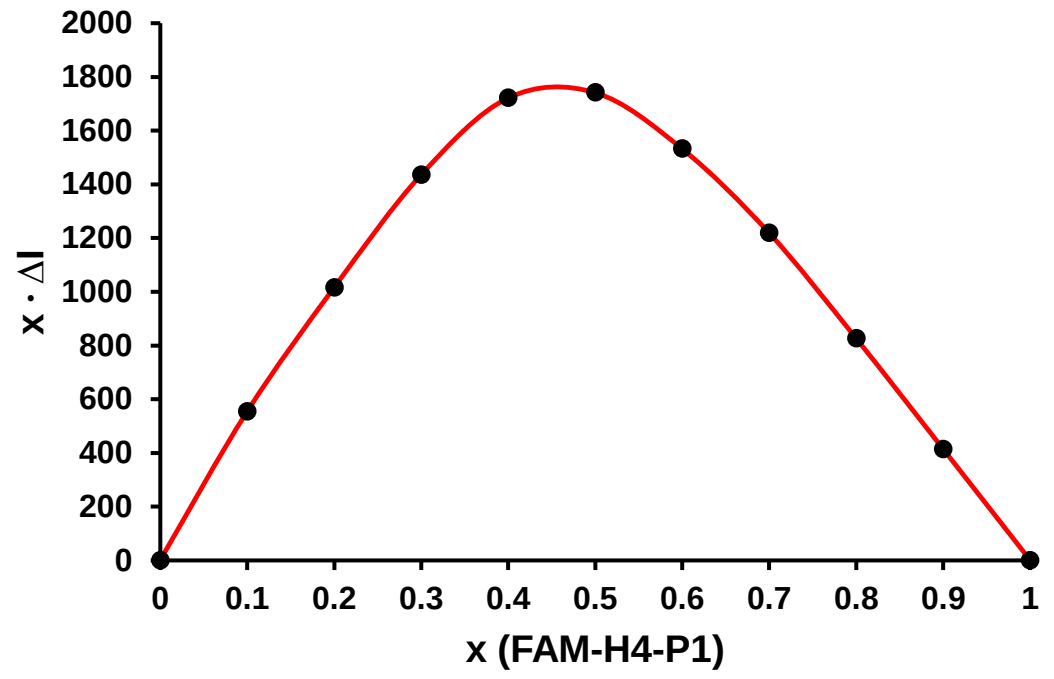
x · ΔI
x (FAM-H4-P1)

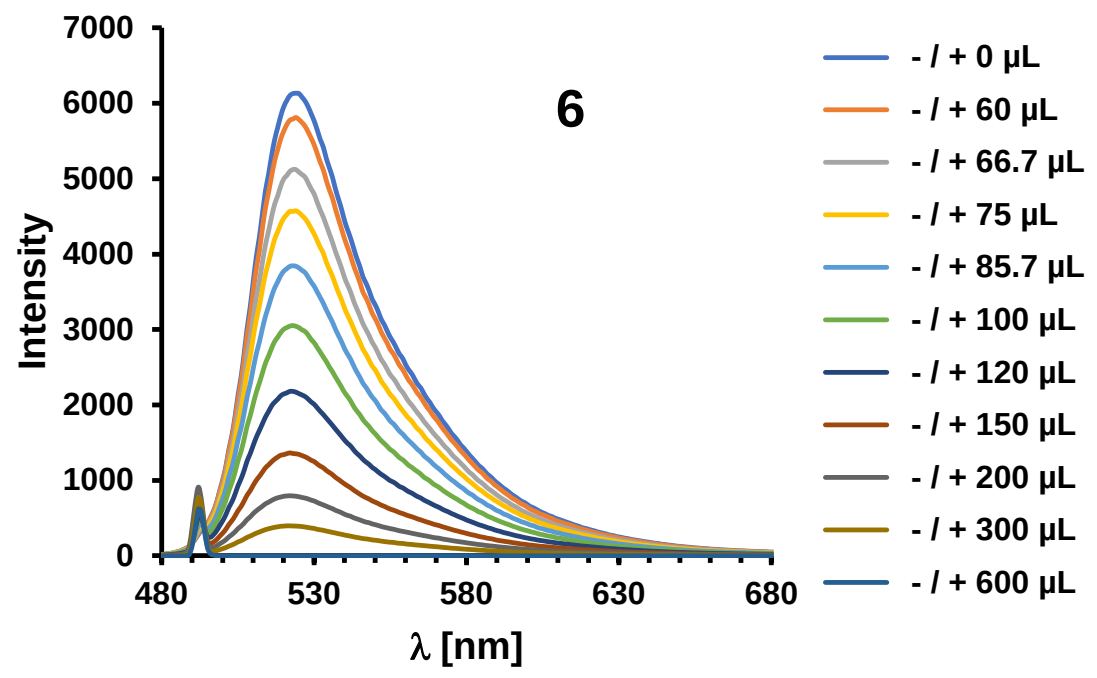
6
Intensity
λ [nm]
- / + 0 µL
- / + 60 µL
- / + 66.7 µL
- / + 75 µL
- / + 85.7 µL
- / + 100 µL
- / + 120 µL
- / + 150 µL
- / + 200 µL
- / + 300 µL
- / + 600 µL

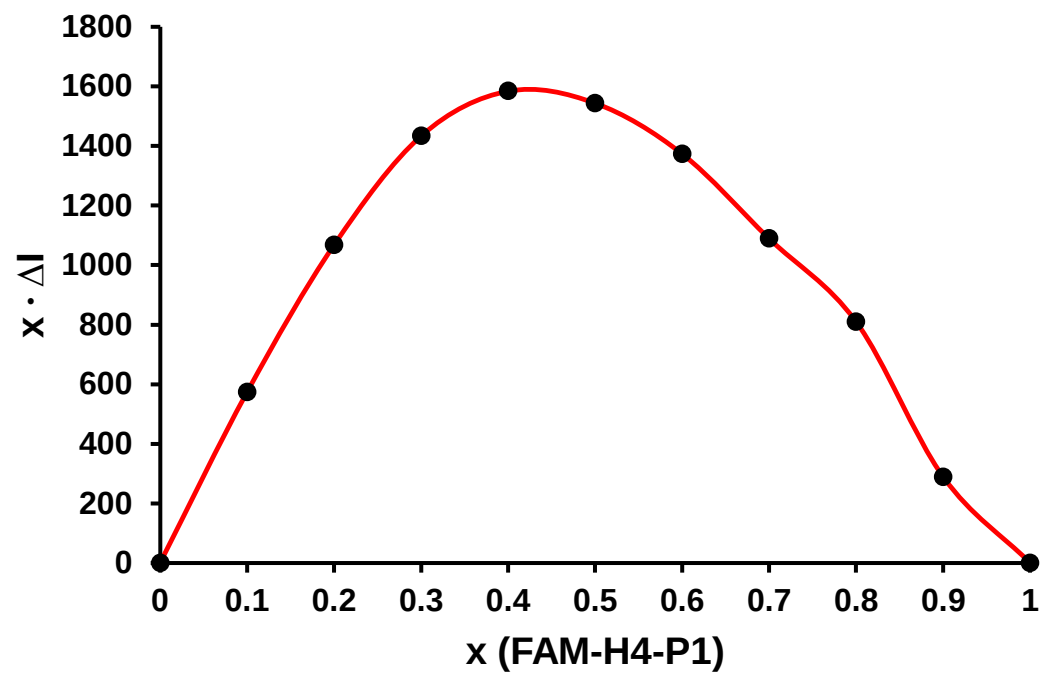
x · ΔI
x (FAM-H4-P1)

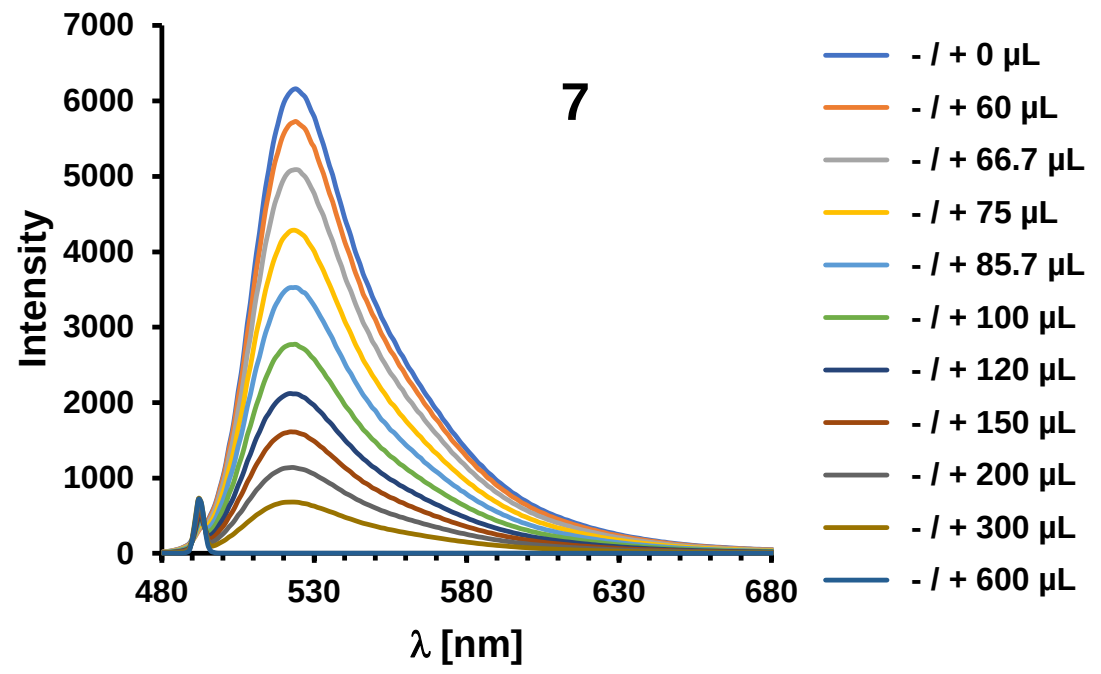
7
Intensity
λ [nm]
- / + 0 µL
- / + 60 µL
- / + 66.7 µL
- / + 75 µL
- / + 85.7 µL
- / + 100 µL
- / + 120 µL
- / + 150 µL
- / + 200 µL
- / + 300 µL
- / + 600 µL

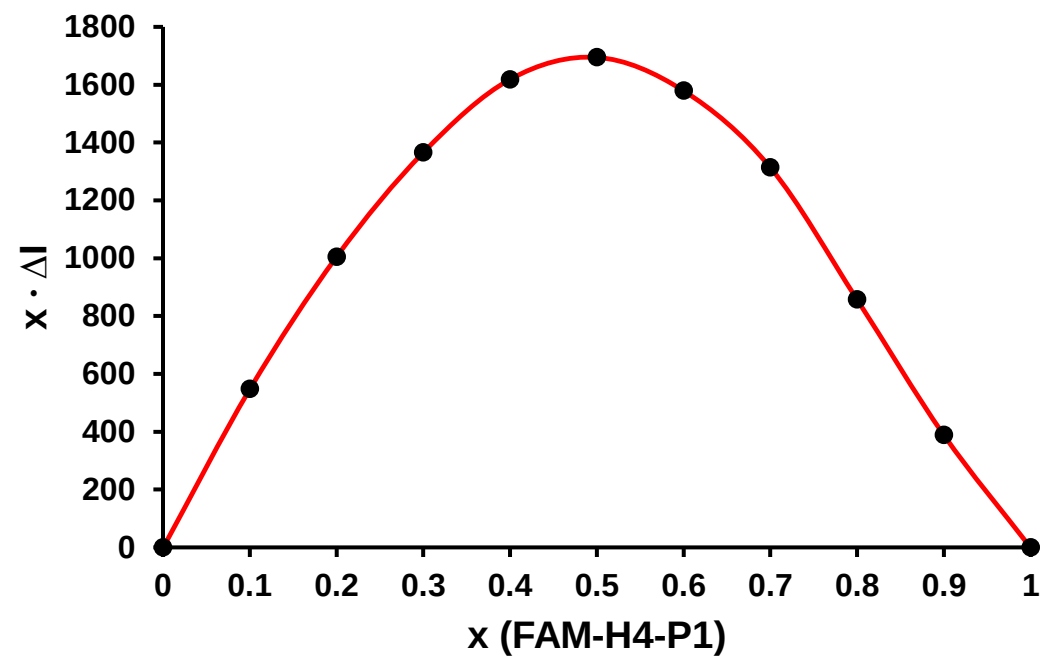
x · ΔI
x (FAM-H4-P1)

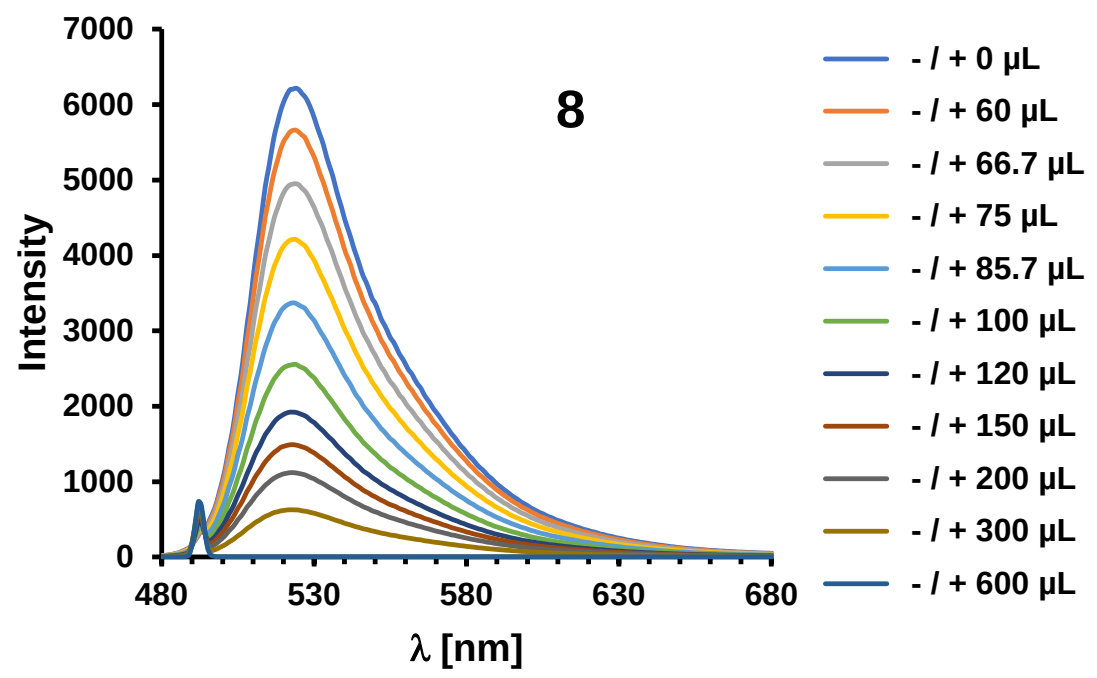
8
Intensity
λ [nm]
- / + 0 µL
- / + 60 µL
- / + 66.7 µL
- / + 75 µL
- / + 85.7 µL
- / + 100 µL
- / + 120 µL
- / + 150 µL
- / + 200 µL
- / + 300 µL
- / + 600 µL

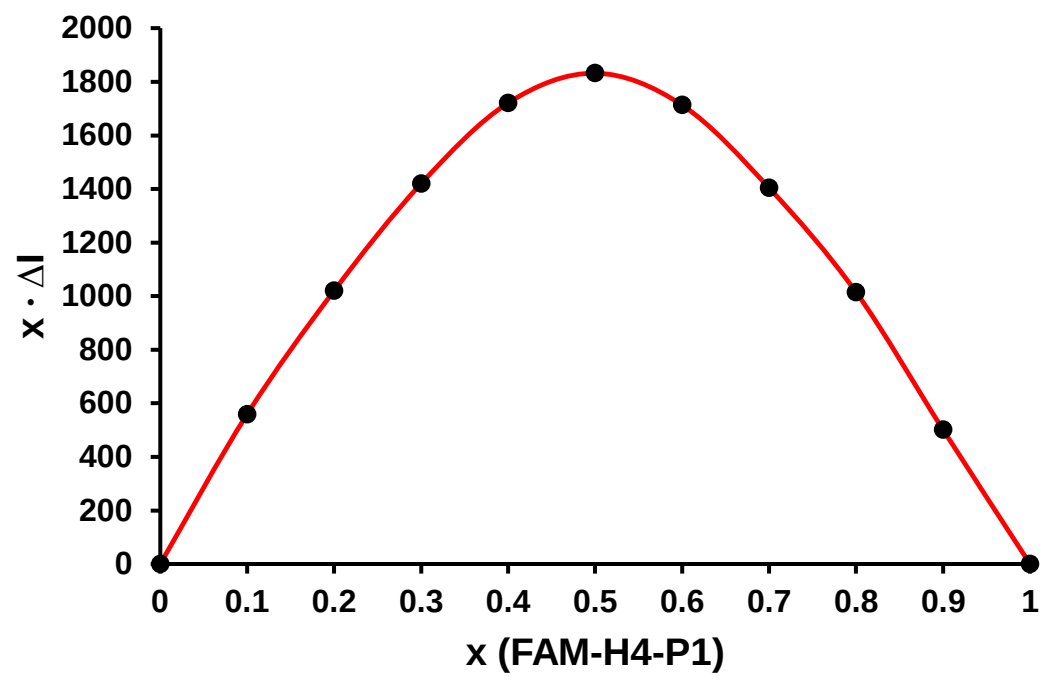
x · ΔI
x (FAM-H4-P1)

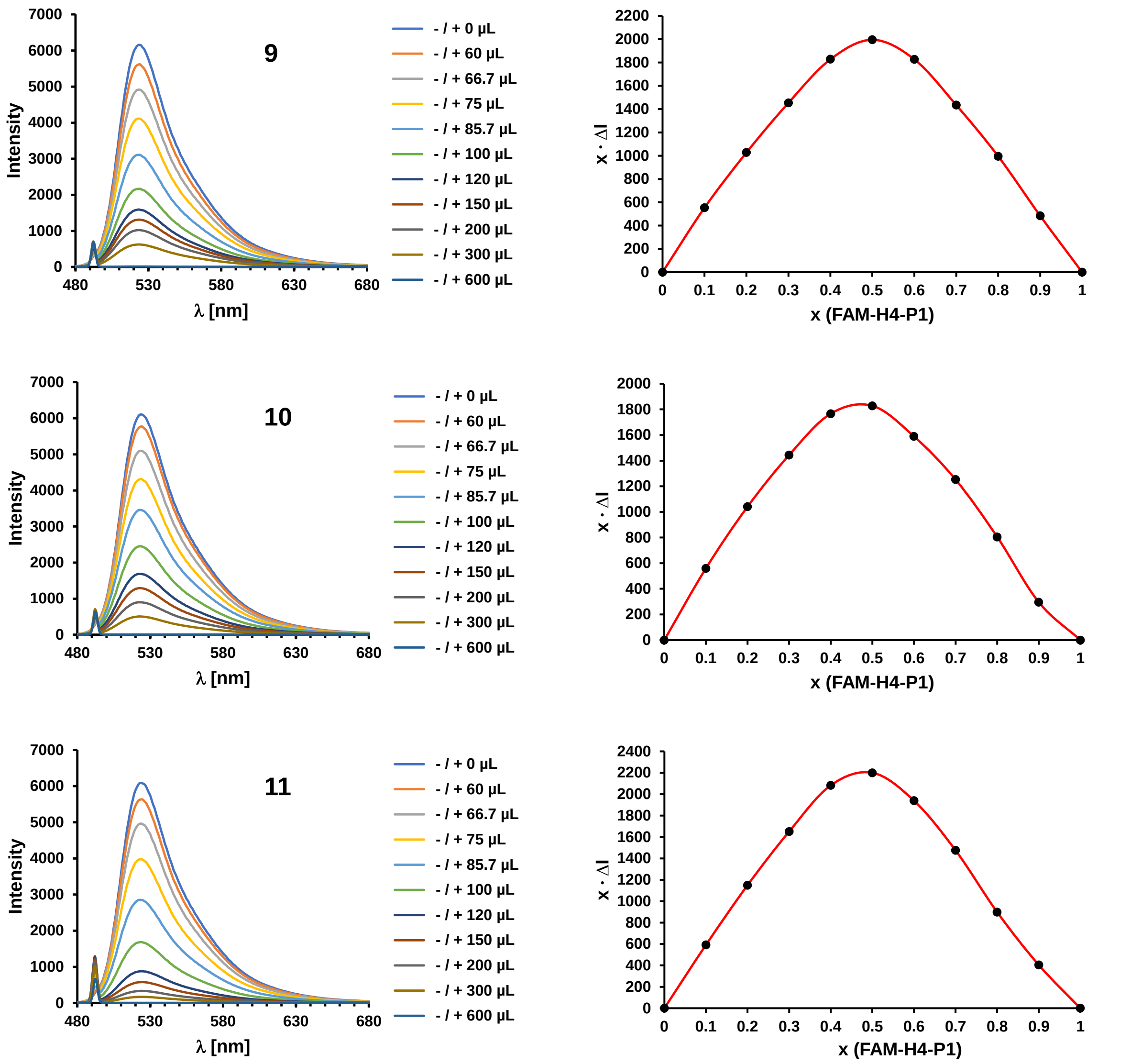

Figure S11: Determination of complex stoichiometry via the Job Plot method executed for the complexation of H4-P1 with advanced tweezers. Left: Fluorescence spectra (exc. at lEx=495 nm and emi. at lEm= 524 nm. Right: Job-Plots for the determination of the respective complex stoichiometry. In most cases a 1:1 complex was formed.

Job Plot method with FAM-H4-Full-Length

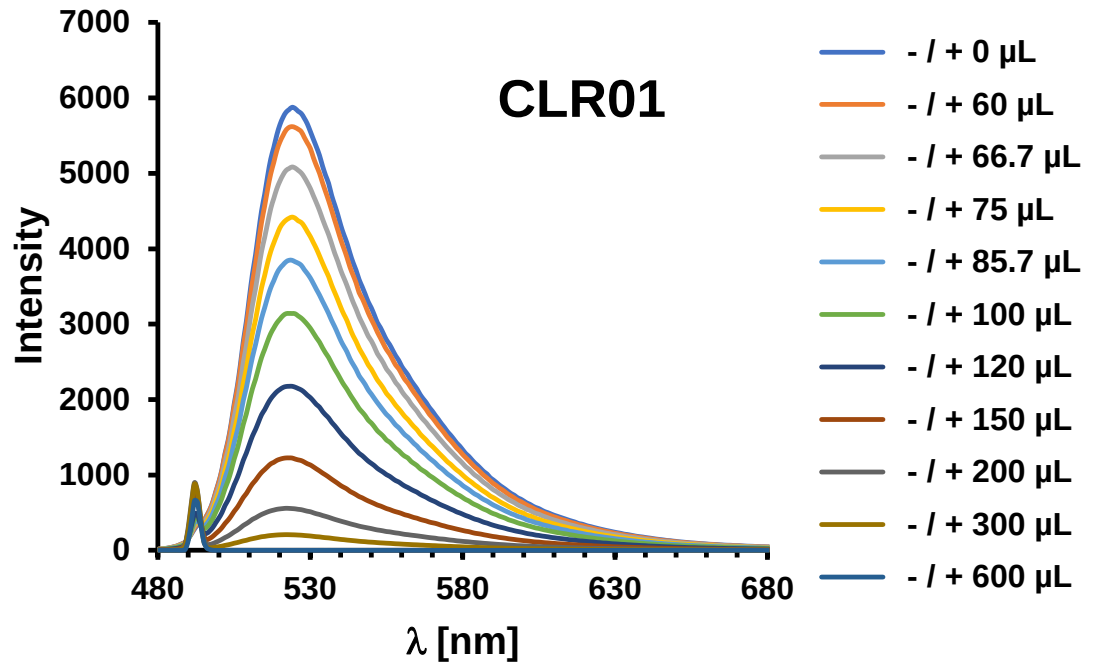

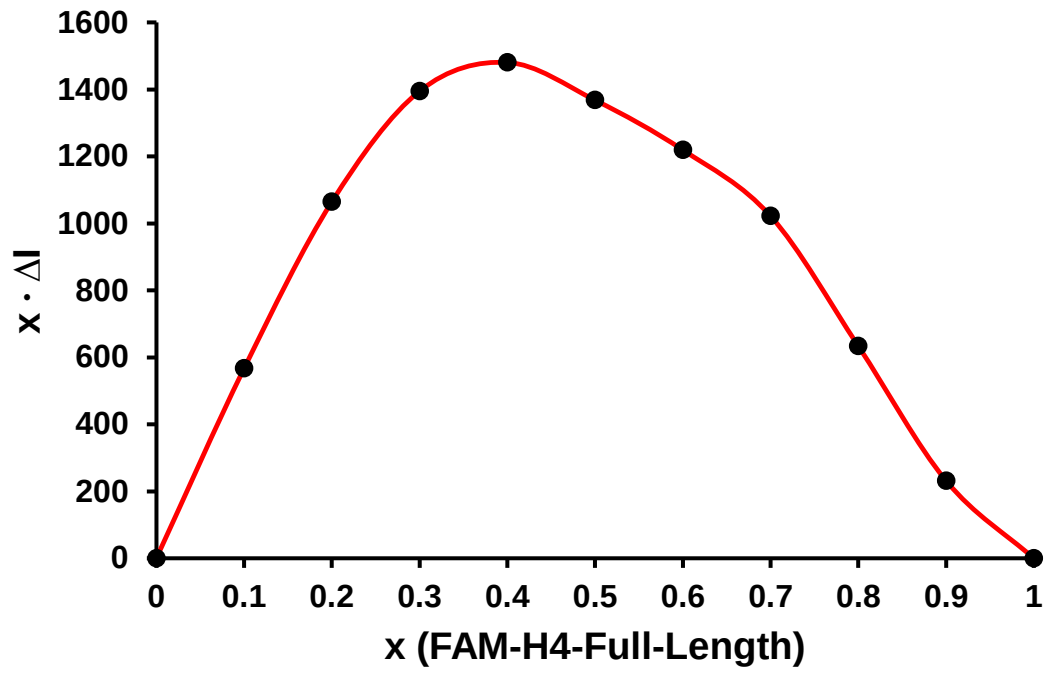

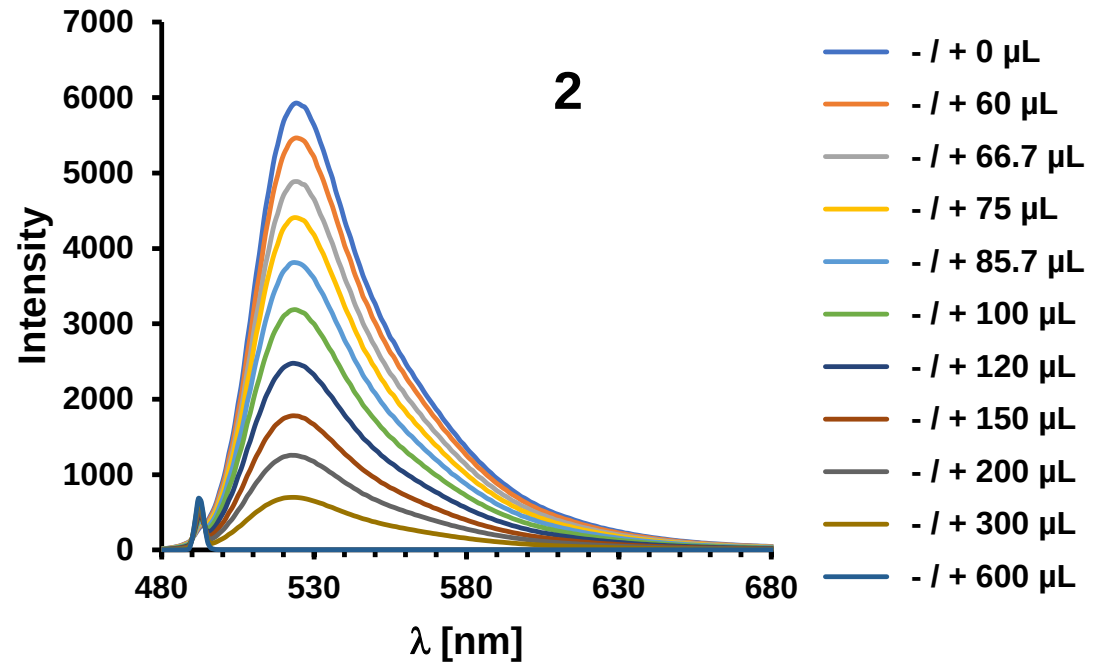

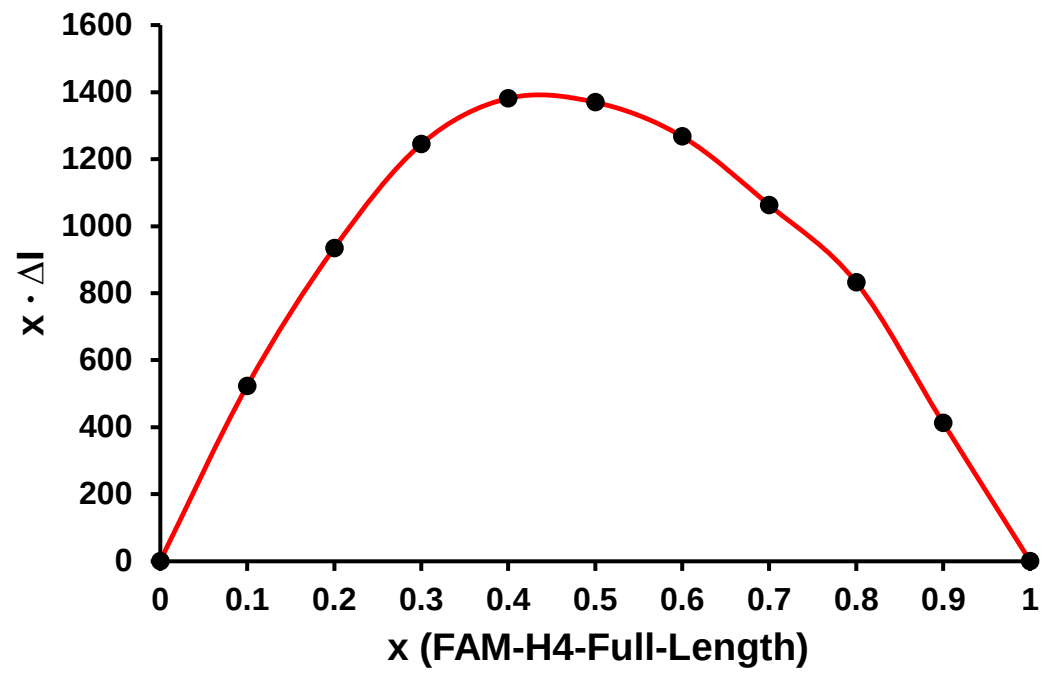

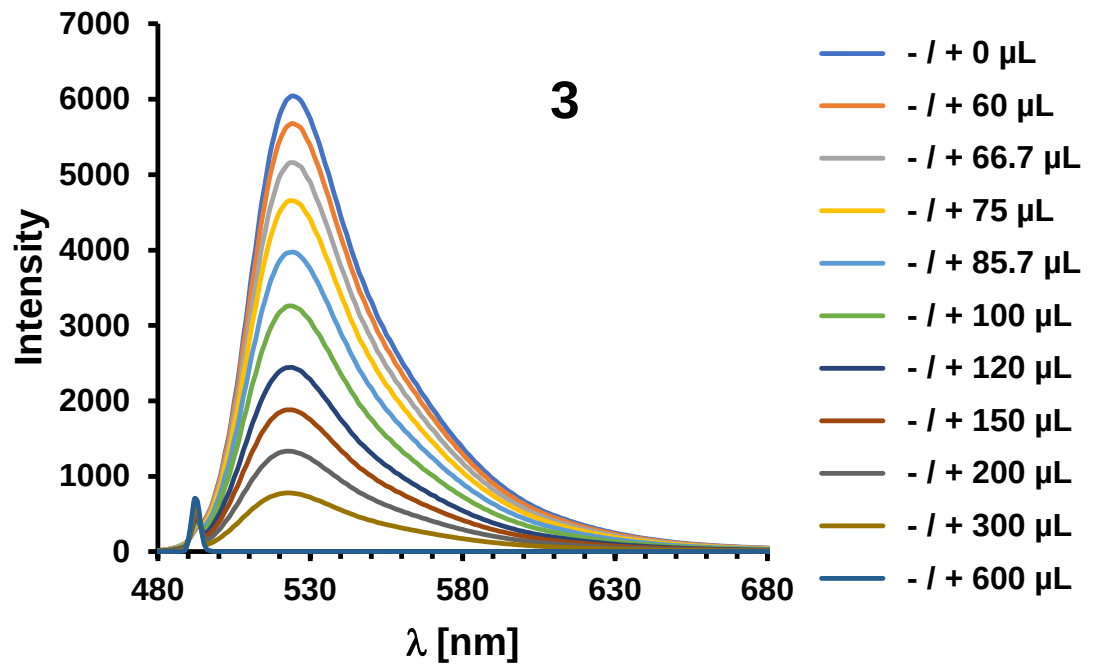

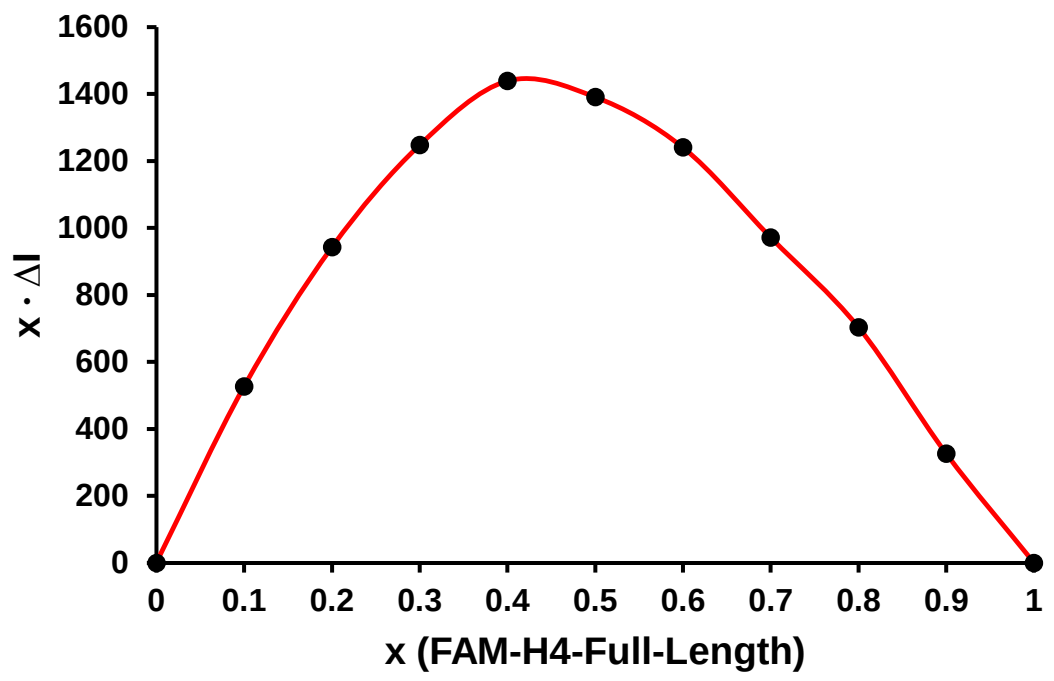

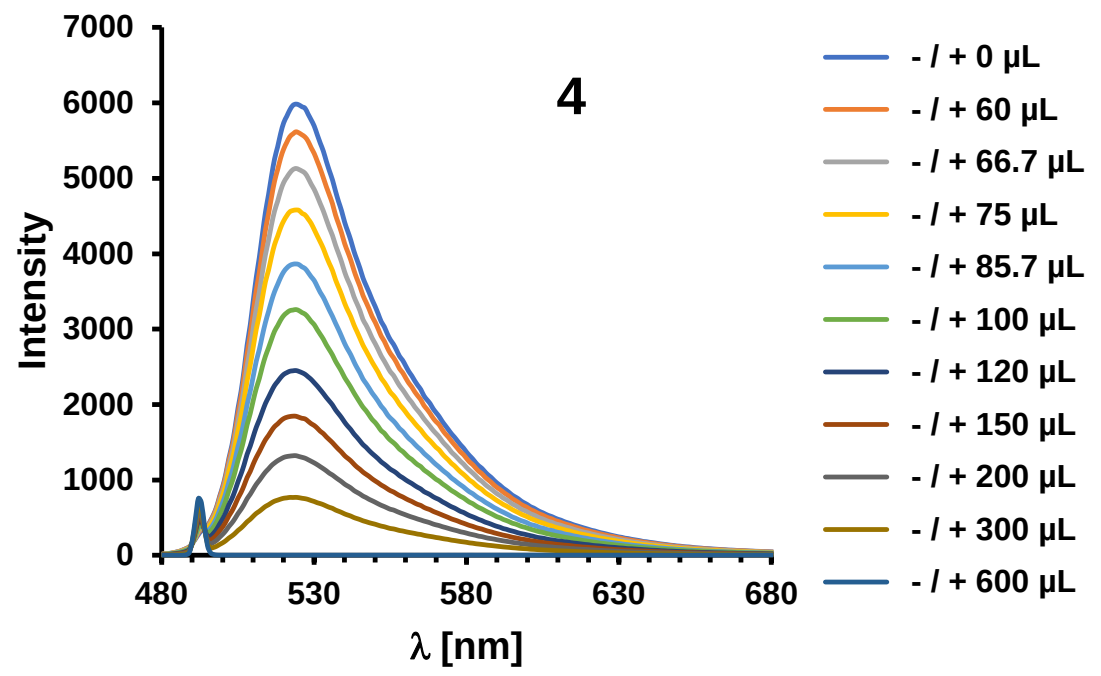

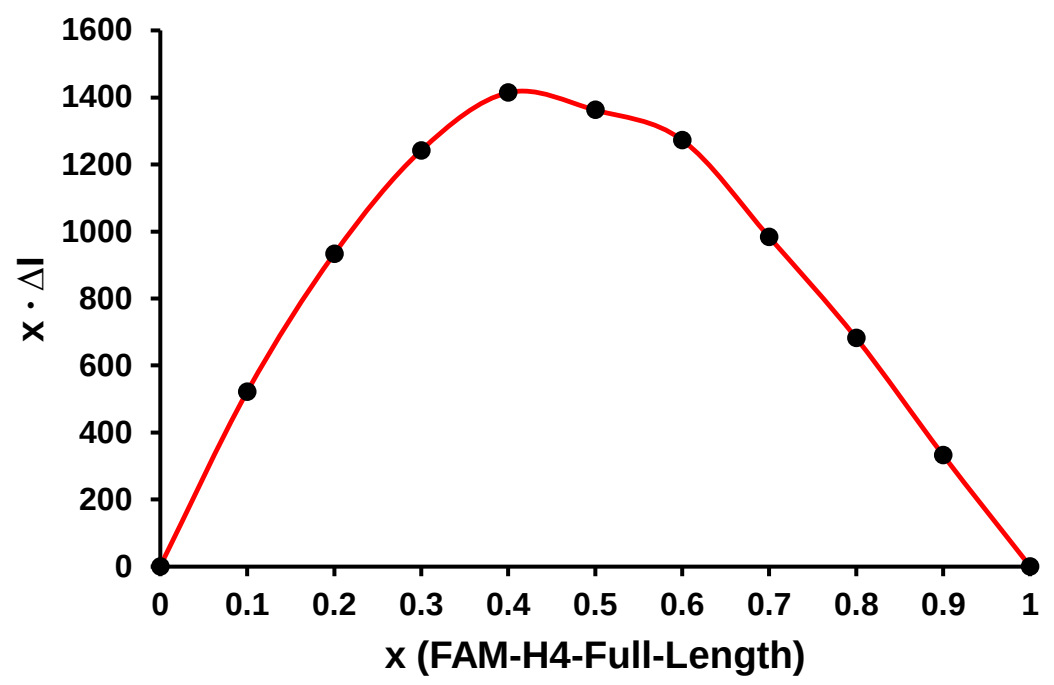

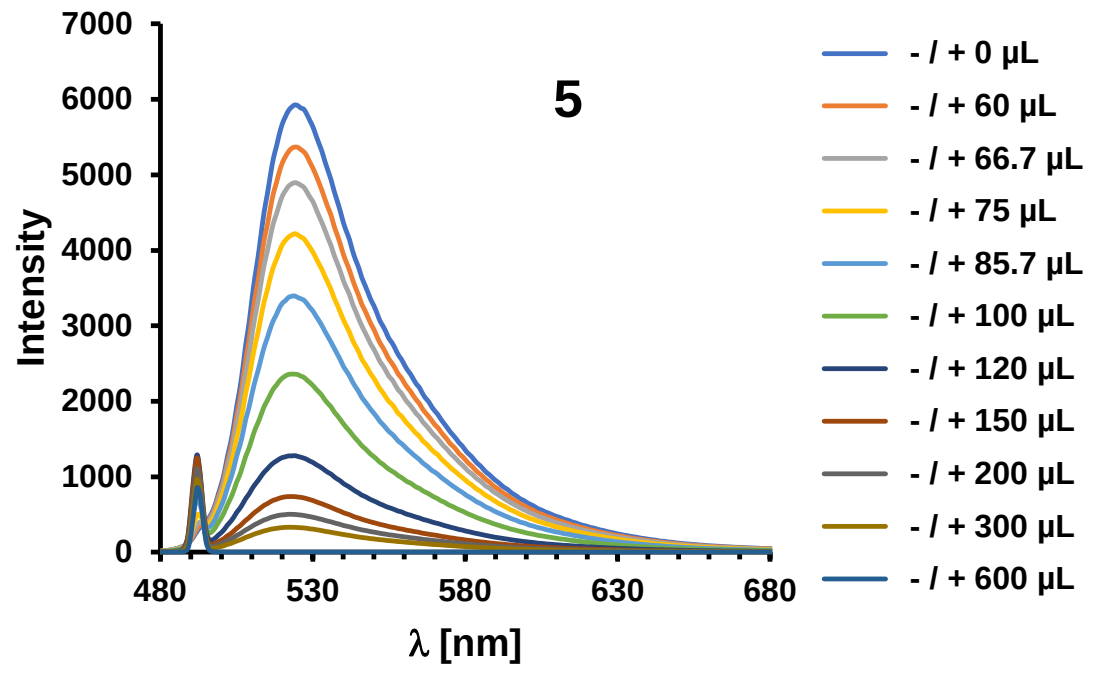
5
Intensity
λ [nm]
0 1000 2000 3000 4000 5000 6000 7000
480 530 580 630 680
- / + 0 µL
- / + 60 µL
- / + 66.7 µL
- / + 75 µL
- / + 85.7 µL
- / + 100 µL
- / + 120 µL
- / + 150 µL
- / + 200 µL
- / + 300 µL
- / + 600 µL

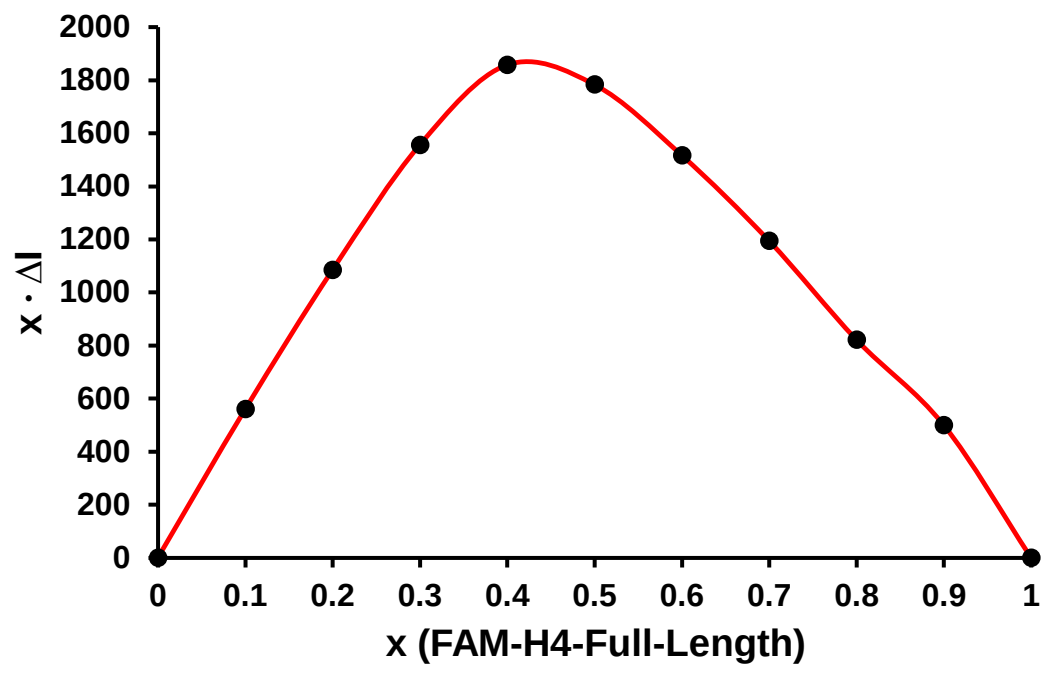
x · ΔI
x (FAM-H4-Full-Length)
0 200 400 600 800 1000 1200 1400 1600 1800 2000
0 0.1 0.2 0.3 0.4 0.5 0.6 0.7 0.8 0.9 1

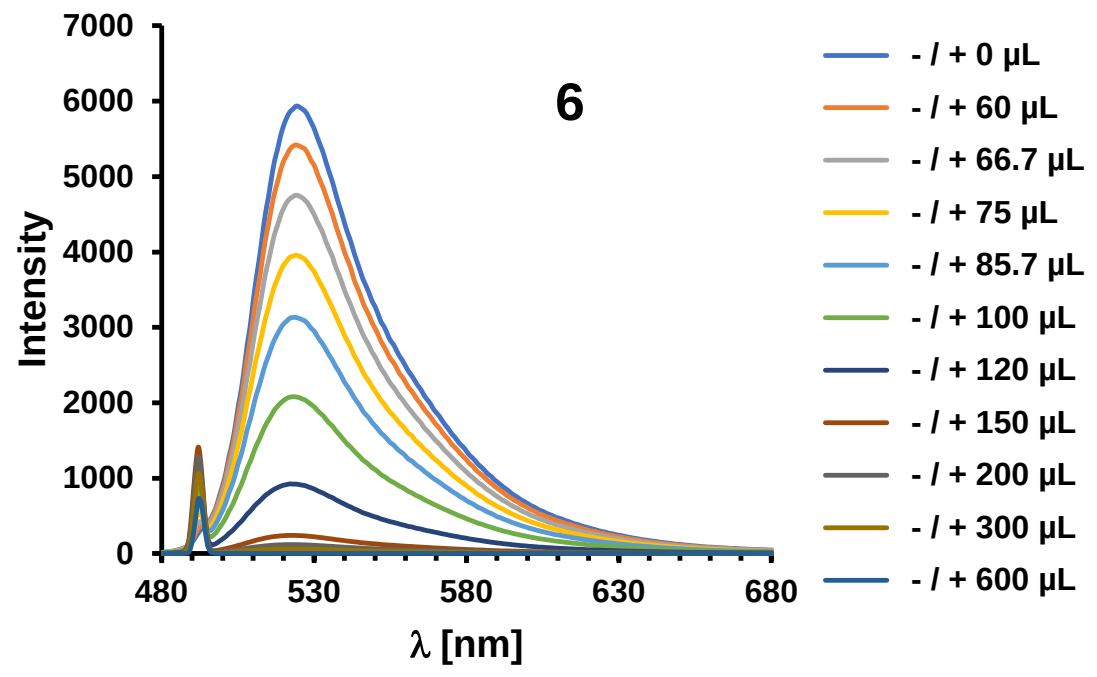
6
Intensity
λ [nm]
0 1000 2000 3000 4000 5000 6000 7000
480 530 580 630 680
- / + 0 µL
- / + 60 µL
- / + 66.7 µL
- / + 75 µL
- / + 85.7 µL
- / + 100 µL
- / + 120 µL
- / + 150 µL
- / + 200 µL
- / + 300 µL
- / + 600 µL

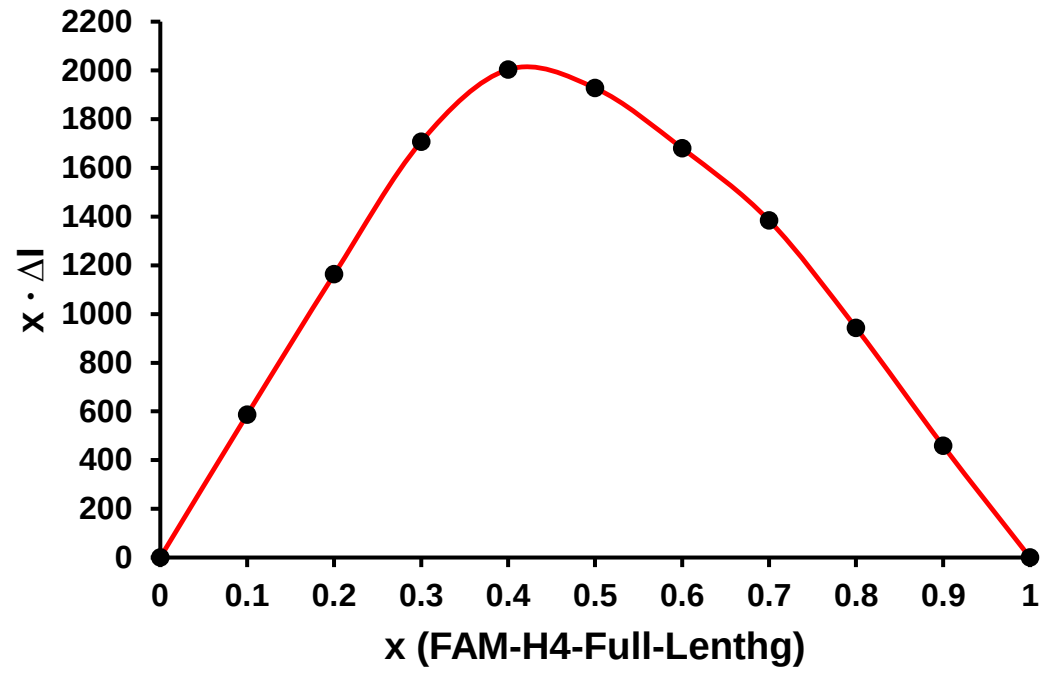
x · ΔI
x (FAM-H4-Full-Lenthg)
0 200 400 600 800 1000 1200 1400 1600 1800 2000 2200
0 0.1 0.2 0.3 0.4 0.5 0.6 0.7 0.8 0.9 1

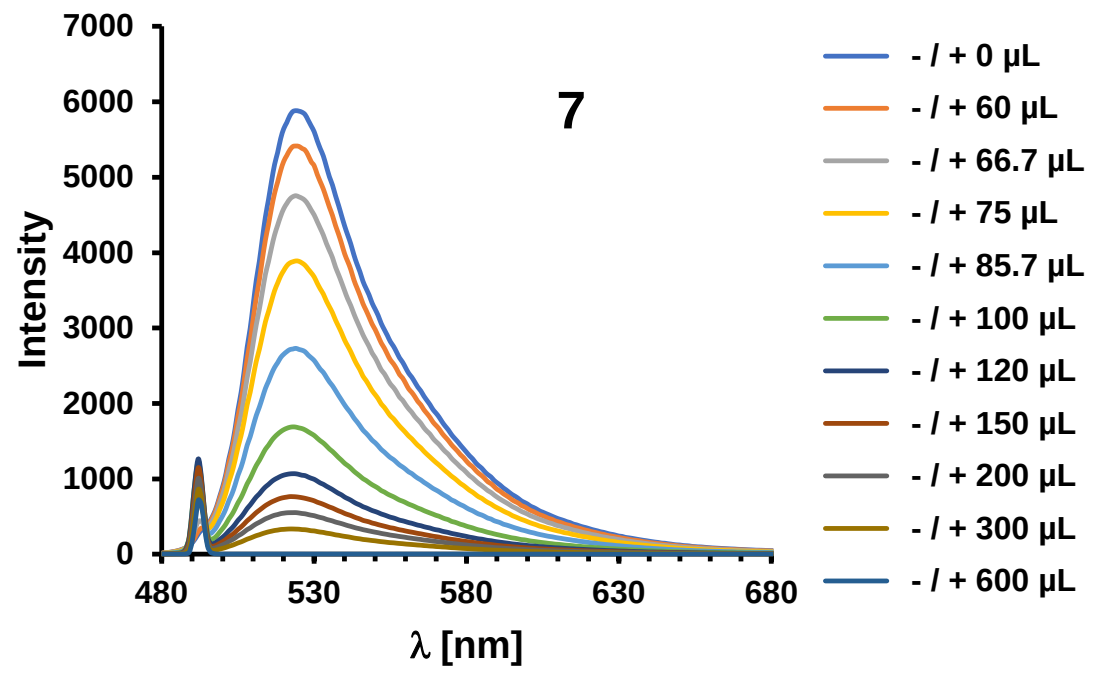
7
Intensity
λ [nm]
0 1000 2000 3000 4000 5000 6000 7000
480 530 580 630 680
- / + 0 µL
- / + 60 µL
- / + 66.7 µL
- / + 75 µL
- / + 85.7 µL
- / + 100 µL
- / + 120 µL
- / + 150 µL
- / + 200 µL
- / + 300 µL
- / + 600 µL

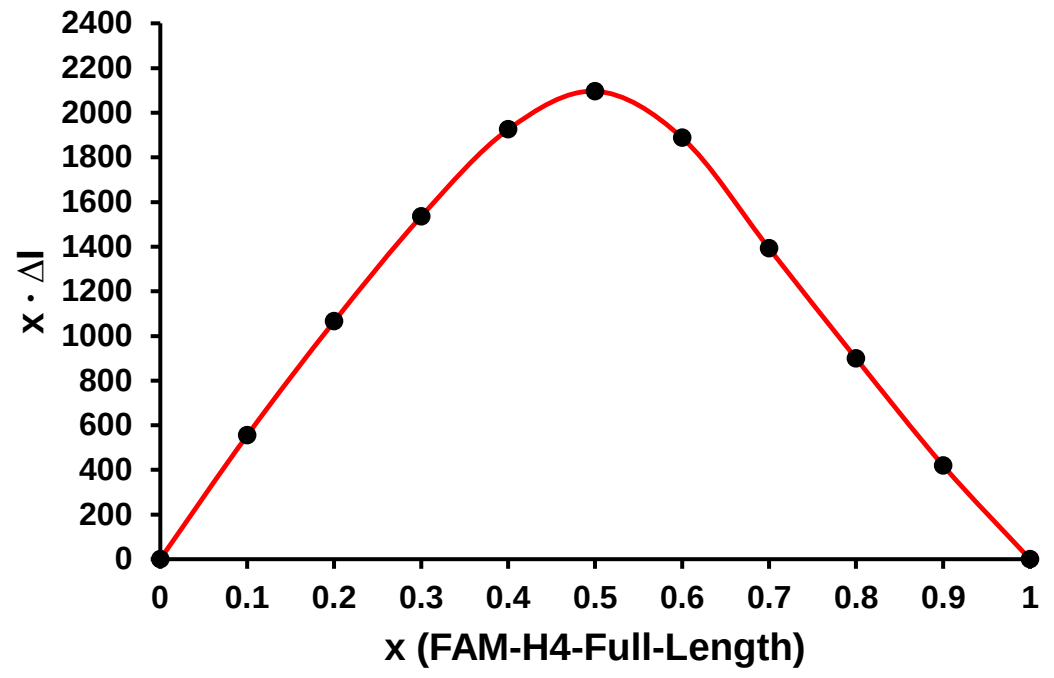
x · ΔI
x (FAM-H4-Full-Length)
0 200 400 600 800 1000 1200 1400 1600 1800 2000 2200 2400
0 0.1 0.2 0.3 0.4 0.5 0.6 0.7 0.8 0.9 1

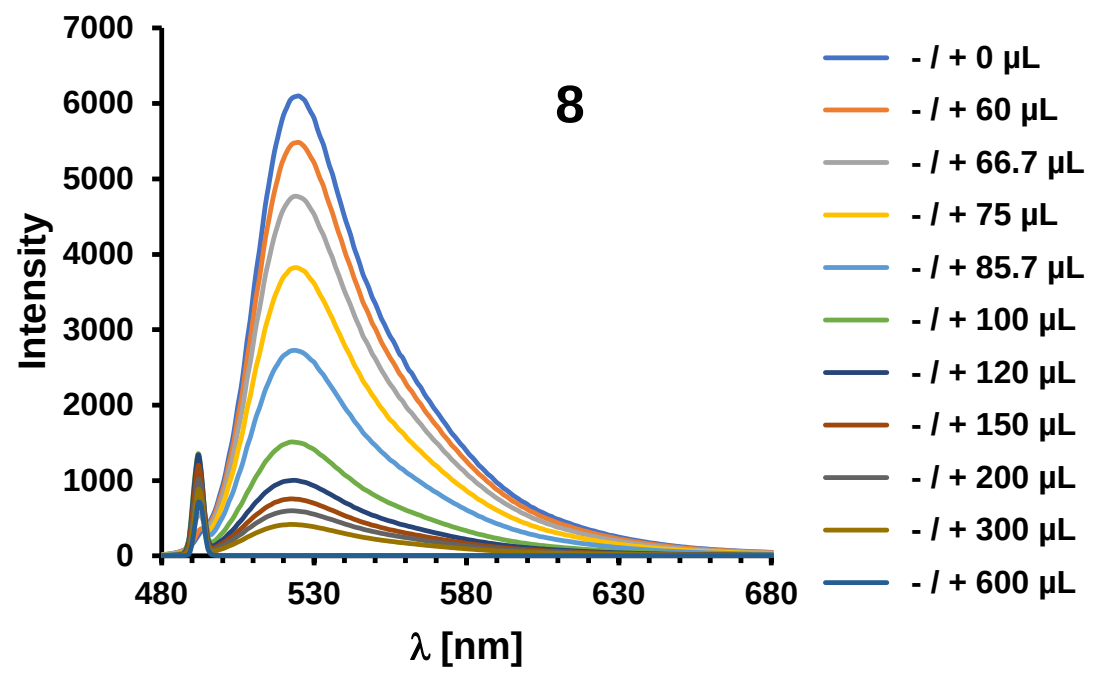
8
Intensity
λ [nm]
0 1000 2000 3000 4000 5000 6000 7000
480 530 580 630 680
- / + 0 µL
- / + 60 µL
- / + 66.7 µL
- / + 75 µL
- / + 85.7 µL
- / + 100 µL
- / + 120 µL
- / + 150 µL
- / + 200 µL
- / + 300 µL
- / + 600 µL

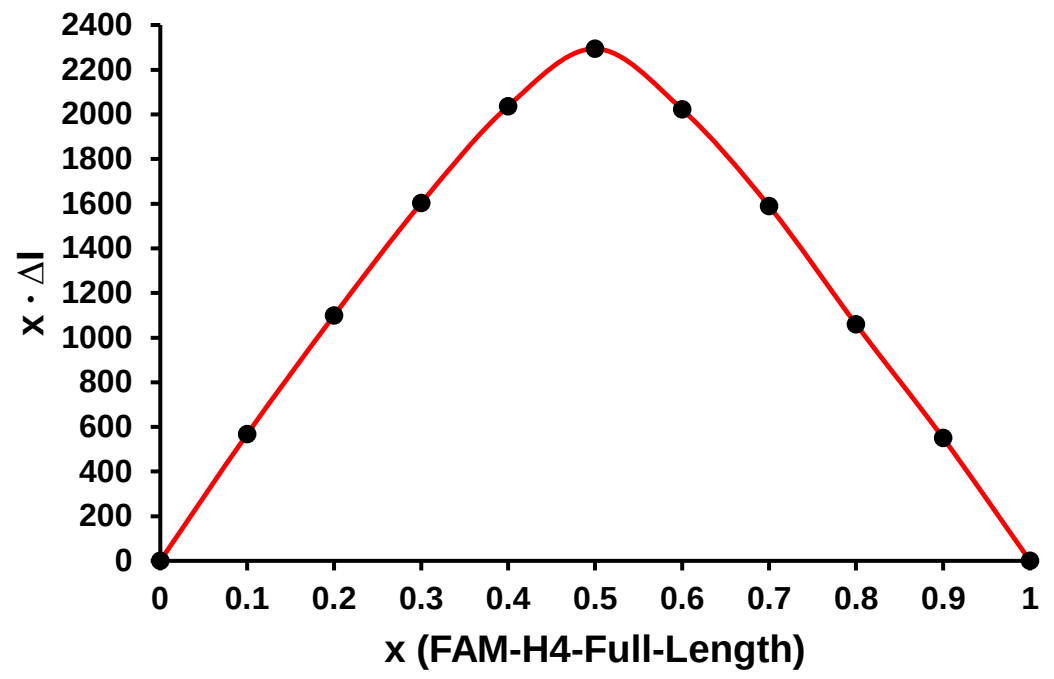
x · ΔI
x (FAM-H4-Full-Length)
0 200 400 600 800 1000 1200 1400 1600 1800 2000 2200 2400
0 0.1 0.2 0.3 0.4 0.5 0.6 0.7 0.8 0.9 1

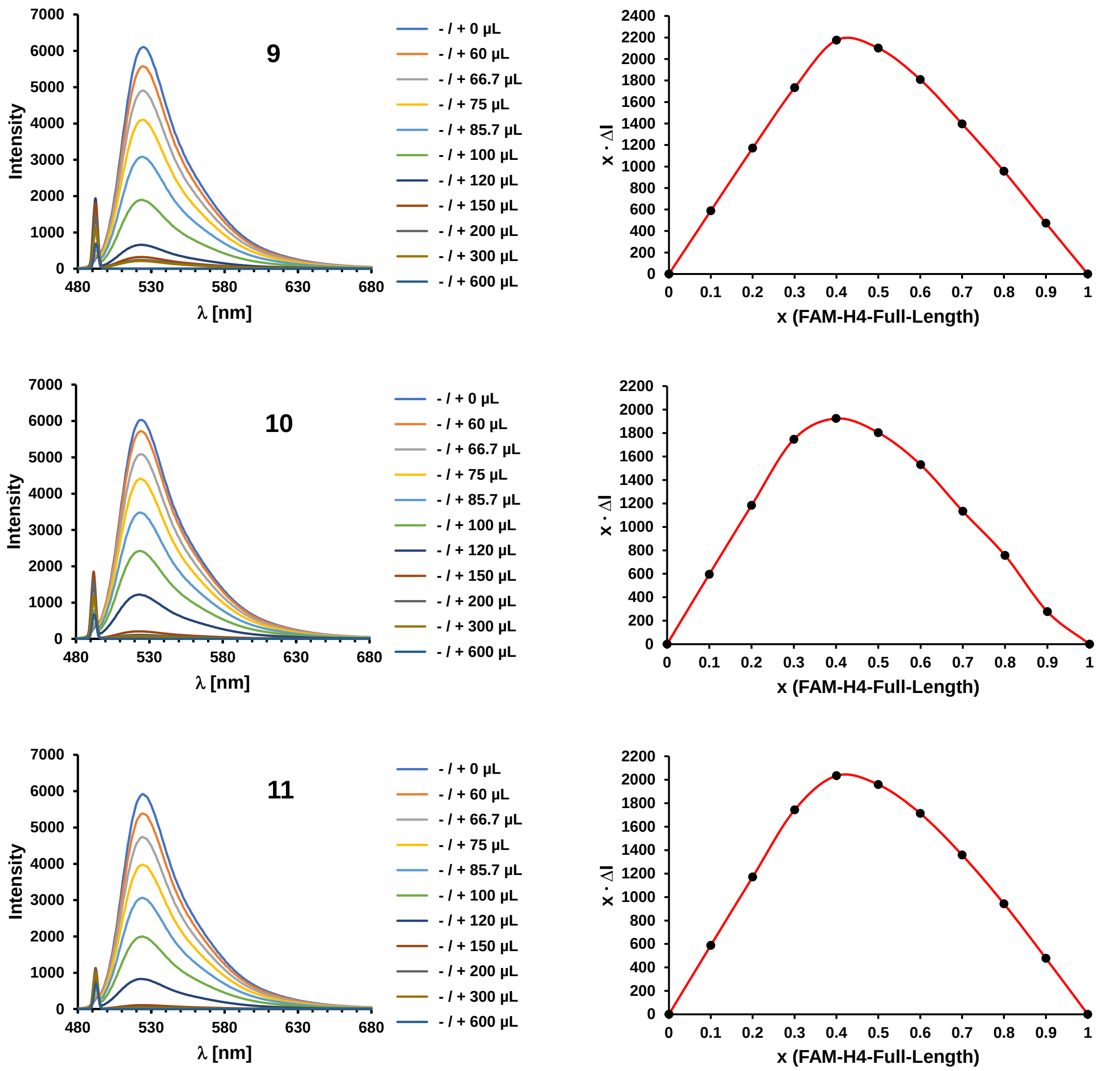

Figure S12: Determination of complex stoichiometry via the Job Plot method executed for the complexation of H4-Full-Length with advanced tweezers. Left: Fluorescence spectra (exc. at IEx=495 nm and emi. at IEm= 524 nm. Right: Job-Plots for the determination of the respective complex stoichiometry. Formation of 1:1 as well as 1:2 complexes were both possible.

General Methods and Materials

Required chemicals for the tweezer synthesis were used as purchased from Sigma-Aldrich, Merck, VWR, TCI, AlfaAesar, Acros Organics, Bachem, abcr, Fluka and Fisher Chemicals in commercial grade. Tetrahydrofuran was dried over sodium and benzephenone prior using. The parent tweezer **CLR01** was prepared by the already known synthesis route.[1],[2] [X] All new monomeric and dimeric tweezer were prepared according to one of the two procedures written below. Acidic azidopeptides for monomeric tweezers were synthesized using the CEM Liberty Microwave Peptide Synthesizer. H4 peptide fragments as well as the labeled ones were ordered from INTAVIS with at least 95-98 % purity.
Purification of certain tweezer compounds was achieved via preparative reversed-phase high performance liquid chromatography (HPLC) using the Shimadzu Prominence UFLC system. The reverse phase column from Phenomenex (Luna® 5 µm C18 (2), 100 Å, 150/ 21.2 mm) was served as stationary phase. A gradient run via acetonitrile/water + 0.1% TFA (flow rate of 25 mL min^{-1}) facilitated the isolation of the desired product.
For the characterization of all synthesized compounds, NMR spectra (^{1}H, ^{13}C and ^{31}P NMR) were recorded with the Bruker AVNeo400 (^{1}H = 400 MHz; ^{13}C = 101 MHz, ^{31}P = 162 MHz) and *Bruker* DRX 600 (^{1}H = 600 MHz; ^{13}C = 151 MHz, ^{31}P = 243 MHz) at 25 °C. High-resolution mass spectra were acquired with the Q-TOF mass spectrometer Bruker maXis 4G (ESI).

Synthesis of clickable azidopeptides for monomeric tweezers

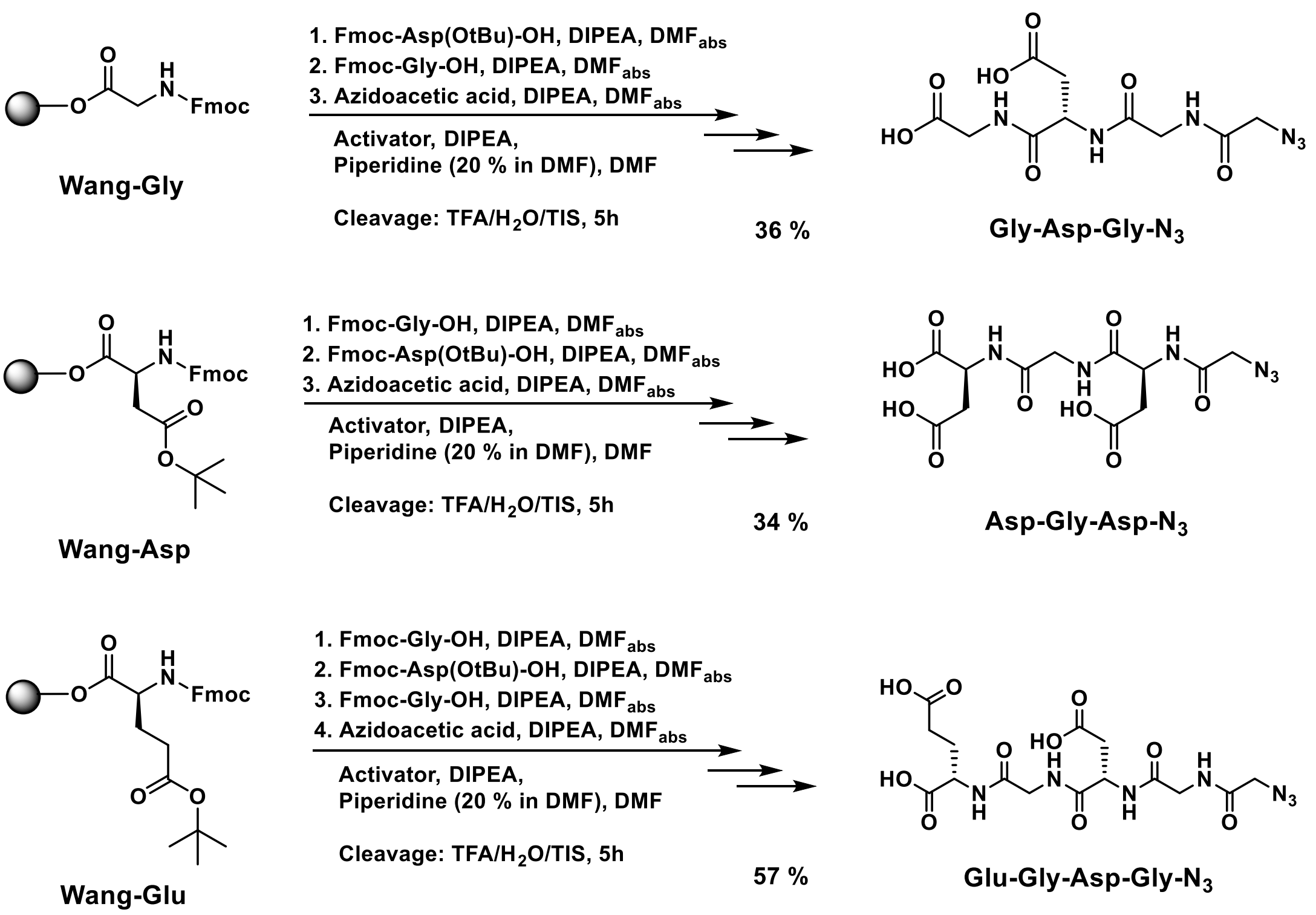

Figure S13: Automated solid phase peptide synthesis of selected acidic peptide attachments with N-terminal azidoacetate unit. Coupling was performed with an Fmoc protocol on Wang resin preloaded with the first amino acid. Cleavage was effected with TFA/H_2O/TIS, purification was achieved by precipitation with cold ether and subsequent preparative HPLC, if necessary.

Synthesis of mono- and divalent molecular tweezers

Monovalent and divalent molecular tweezers were synthesized from the monobutinyl tweezer **1,** equipped with one terminal alkyne group. This key compound was prepared according to the already published synthesis route starting from the precursor diacetoxy tweezer.[3] For this work, several peptides and linkers carrying one or two azides were introduced into monobutinyl tweezer **1**. All monovalent peptide tweezers were synthesized according to the one-pot click protocol, while the dimeric tweezers were prepared via an iterative coupling method.[4] Both procedures are described in the manuscript. The required amount of starting compounds, purification method and the characterisation of new molecular tweezers are detailed below.

The synthesis of the dimeric PEG tweezer **10** was already published.[4]

Purification method and characterization of new molecular tweezers

1) Synthesis of Molecular Tweezers with Peptidic Attachments

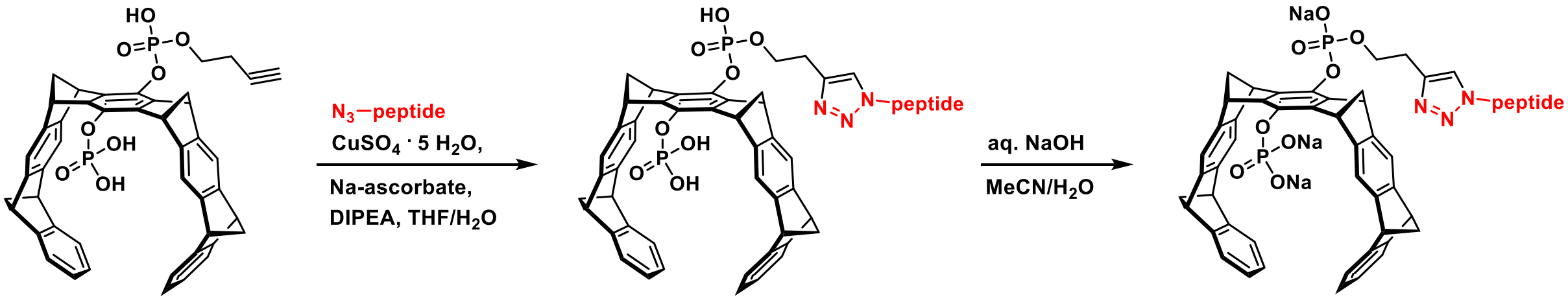

1

Figure S14. Simple click protocol of butynyl MT with an azidopeptide, followed by quantitative neutralization to water-soluble peptide tweezer.

Peptide tweezer 2:

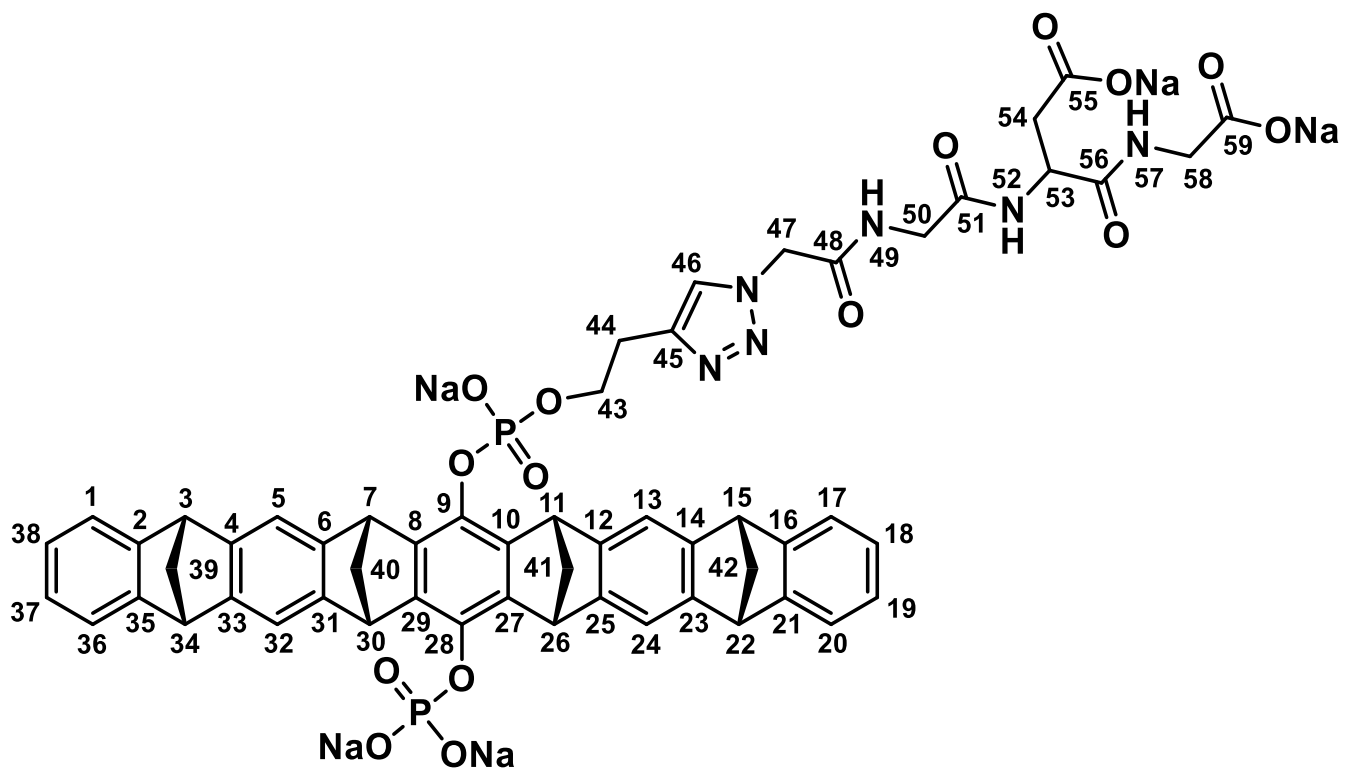

The phosphoric acid **2**was prepared from monobutinyl tweezer **1** (12 mg, 15.4 µmol, 1 eq.) and azido-peptide **N$_3$-Gly-Asp-Gly** (13 mg, 39.4 µmol, 2.6 eq.) using the one-pot click procedure. The crude product was purified by preparative HPLC column chromatography (H_2O/MeCN + 0.1 % TFA; gradient). After lyophilization, the free phosphoric acid was obtained as a colourless solid (9.9 mg, 8.93 µmol, 58 %). ^{1}H NMR (400 MHz, DMSO-*d$_6$*): δ = 2.21 (d, *J* = 7.0 Hz, 2H), 2.29 (d, *J* = 8.4 Hz, 6H), 2.47 (d, *J* = 8.4 Hz, 1H), 2.71 (dd, *J* = 16.5, 5.2 Hz, 1H), 2.94 (t, *J* = 6.7 Hz, 2H), 3.66 – 3.77 (m, 2H), 3.80 – 3.92 (m, 2H), 4.06 – 4.16 (m, 6H), 4.31 (d, *J* = 11.1 Hz, 4H), 4.66 (td, *J* = 8.2, 5.4 Hz, 1H), 5.20 (s, 2H), 6.71 – 6.83 (m, 4H), 7.00 – 7.09 (m, 4H), 7.11 (d, *J* = 5.1 Hz, 4H), 7.98 (s, 1H), 8.14 (t, *J* = 5.8 Hz, 1H), 8.37 (d, *J* = 8.2 Hz, 1H), 8.62 (t, *J* = 5.4 Hz, 1H) ppm; ^{13}C NMR (101 MHz, DMSO-*d$_6$*): δ = 26.7, 36.3, 40.8, 42.1, 48.0, 49.3, 50.3, 51.5, 65.7, 67.8, 69.0, 118.9, 121.5, 124.3, 124.5, 141.0, 141.6, 142.8, 146.7, 147.0, 150.3, 165.9, 168.3, 170.9, 170.9, 171.6 ppm; ^{31}P NMR (162 MHz, DMSO-*d$_6$*): δ = -5.43, -5.88 ppm; HRMS (pos. ESI, MeOH): m/z calc. for $C_{56}H_{50}N_6O_{15}P_2$ [M+H]$^+$: 1109.2882, found: 1109.2888.
Deprotonation of the phosphoric acid **2**(9.9 mg, 8.93 µmol, 1 eq.) with aq. NaOH (44.6 µL, 45 µmol, 5 eq.) delivered the pentasodium salt **2**as a colourless solid in quantitative yield (11.1 mg, 9.1 µmol, 98 % quant.).^{1}H NMR (400 MHz, D_2O): δ = 2.26 (d, *J* = 7.6 Hz, 2H), 2.32 (s, 1H), 2.36 (s, 4H), 2.46 (d, *J* = 7.6

Hz, 2H), 2.58 – 2.65 (m, 1H), 2.75 (dd, *J* = 16.0, 5.0 Hz, 1H), 3.34 (s, 2H), 3.73 (q, *J* = 17.3 Hz, 2H), 4.08 (s, 2H), 4.17 (d, *J* = 8.6 Hz, 4H), 4.28 (s, 2H), 4.56 (s, 2H), 4.72 (dd, *J* = 8.5, 5.0 Hz, 1H), 5.11 (s, 2H), 6.34 (s, 4H), 6.97 (dd, *J* = 39.8, 3.2 Hz, 4H), 7.30 (d, *J* = 22.6 Hz, 4H), 7.45 (s, 1H) ppm; ^{13}C NMR (101 MHz, D_2O): δ = 26.3, 38.8, 42.5, 43.4, 48.1, 48.3, 50.6, 51.6, 64.7, 68.2, 69.2, 116.4, 116.7, 121.3, 124.6, 124.9, 140.6, 142.3, 144.9, 147.7, 148.0, 150.4, 168.4, 170.8, 173.0, 176.4, 177.9 ppm;^{31}P NMR (162 MHz, D_2O): δ = 0.95, -3.83 ppm. HRMS (neg. ESI, MeOH): m/z calc.for $C_{56}H_{50}N_6O_{15}P_2$ [M-2H]$^+$: 554.1362, found: 554.1369.

Peptide tweezer 3:

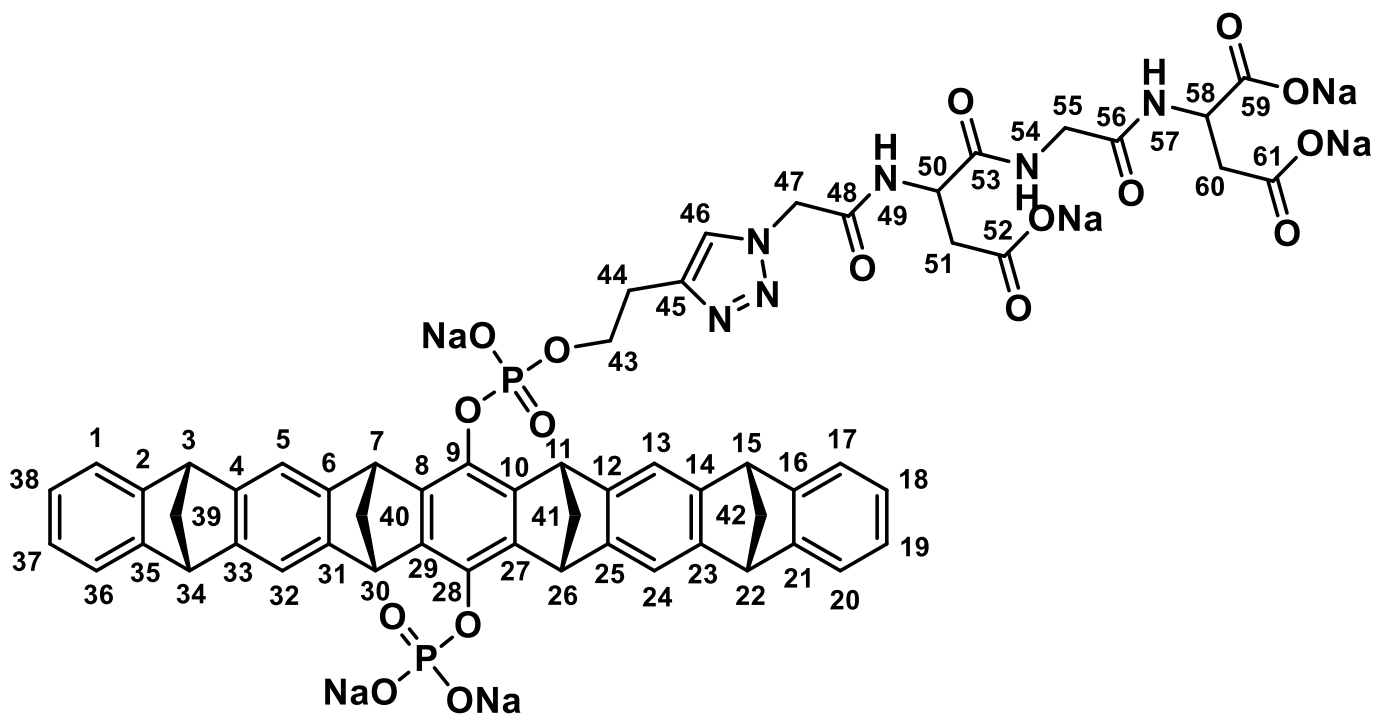

The phosphoric acid **3** was prepared from monobutinyl tweezer **1** (10 mg, 12.8 µmol, 1 eq.) and azido-peptide **N_3-Asp-Gly-Asp** (11 mg, 28.3 µmol, 2.2 eq.) using the one-pot click procedure. The crude product was purified by preparative HPLC column chromatography (H_2O/MeCN + 0.1 % TFA; gradient). After lyophilization, the free phosphoric acid was obtained as a colourless solid (8.9 mg, 7.63 µmol ,59 %). ^1H NMR (600 MHz, DMSO-*d6*): δ = 2.21 (d, *J* = 6.9 Hz, 2H), 2.29 (d, *J* = 10.8 Hz, 6H), 2.55 – 2.60 (m, 2H), 2.71 (ddd, *J* = 22.5, 16.6, 5.5 Hz, 2H), 2.94 (t, *J* = 6.4 Hz, 2H), 3.73 (dd, *J* = 5.6, 2.8 Hz, 2H), 4.10 (dd, *J* = 13.6, 6.0 Hz, 6H), 4.31 (d, *J* = 14.1 Hz, 4H), 4.55 (dd, *J* = 14.2, 6.4 Hz, 1H), 4.63 (td, *J* = 7.9, 5.3 Hz, 1H), 5.17 (q, *J* = 16.4 Hz, 2H), 6.72 – 6.82 (m, 4H), 7.02 – 7.09 (m, 4H), 7.11 (d, *J* = 9.4 Hz, 4H), 7.97 (s, 1H), 8.09 (d, *J* = 7.9 Hz, 1H), 8.30 (t, *J* = 5.9 Hz, 1H), 8.77 (d, *J* = 7.8 Hz, 1H) ppm; ^{13}C NMR (101 MHz, DMSO-*d6*): δ = 26.8, 36.0, 36.4, 41.9, 48.0, 48.5, 49.7, 50.3, 51.5, 65.7, 67.8, 69.0, 116.8, 121.5, 124.3, 124.5, 136.4, 141.0, 141.6, 142.8, 146.7, 147.0, 150.3, 165.7, 168.4, 170.5, 171.6, 171.7, 172.2 ppm; ^{31}P NMR (162 MHz, DMSO-*d6*): δ = -5.91, -5.43 ppm. HR MS (pos. ESI, MeOH): m/z calc. for $C_{58}H_{52}N_6O_{17}P_2$ [M+H]$^+$: 1167.2937, found: 1167.2937.
Deprotonation of the phosphoric acid **3** (8.9 mg, 7.63 µmol, 1 eq.) with aq. NaOH (458 µL, 45.8 µmol, 6 eq.). delivered the hexasodium salt **3** as a colourless solid in quantitative yield (9.9 mg, 7.63 µmol, quant.). ^1H-NMR (400 MHz, D_2O): δ = 2.24 (dd, *J* = 16.0, 8.5 Hz, 4H), 2.37 (s, 4H), 2.46 (d, *J* = 7.6 Hz, 2H), 2.54 (dd, *J* = 15.8, 9.2 Hz, 1H), 2.68 (dt, *J* = 15.8, 6.2 Hz, 2H), 2.78 (dd, *J* = 16.2, 5.1 Hz, 1H), 3.09 – 3.23 (m, 2H), 3.96 (d, *J* = 3.9 Hz, 2H), 4.13– 4.20 (m, 4H), 4.28 (s, 2H), 4.43 (dd, *J* = 9.1, 4.2 Hz, 1H), 4.55 (s, 2H), 4.75 (dd, *J* = 5.5, 3.7 Hz, 2H), 5.35 (s, 2H), 6.65 (s, 4H), 6.95 (t, *J* = 5.2 Hz, 2H), 7.10 (d, *J* = 6.1 Hz, 2H), 7.25 – 7.32 (m, 4H), 7.95 (s, 1H) ppm; ^{13}C NMR (151 MHz, D_2O): δ = 26.4, 38.9, 39.6, 42.5, 48.1, 48.3, 50.7, 51.9, 51.9, 53.1, 65.0, 68.3, 68.7, 69.6, 116.4, 116.9, 121.2, 121.5, 125.0, 125.5, 140.9, 142.4, 145.1, 147.9, 148.2, 151.1, 168.0, 170.3, 173.8, 177.6 ppm; ^{31}P NMR (162 MHz, D_2O): δ = 0.85, -3.98 ppm; HR MS (pos. ESI, MeOH): m/z calc. for $C_{58}H_{52}N_6O_{17}P_2$ [M+2H]$^+$: 584.1505, found: 584.1502.

Peptide tweezer 4:

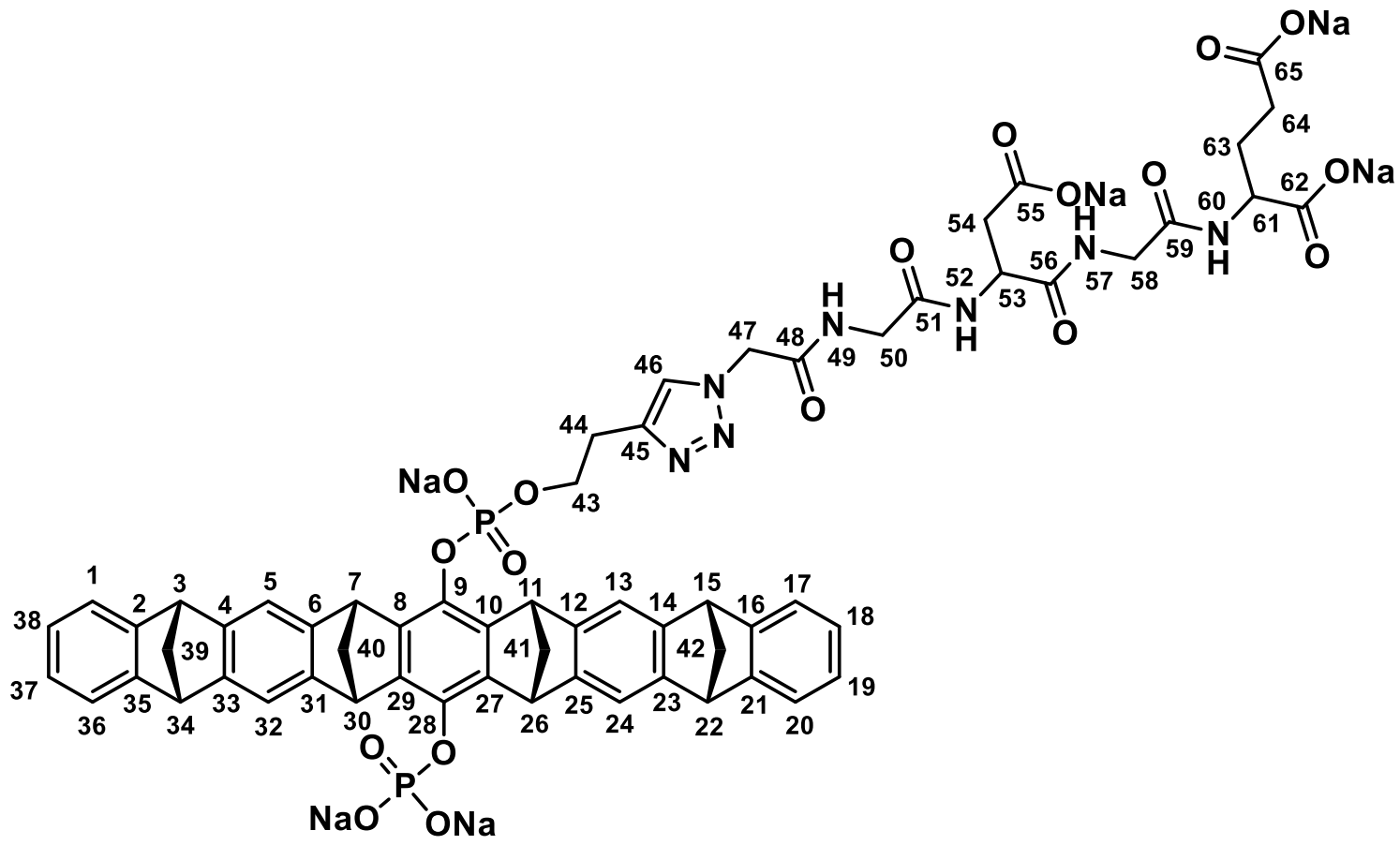

The phosphoric acid **4** was prepared from monobutinyl tweezer **1** (10 mg, 12.8 µmol, 1 eq.) and azido-peptide **N_3-Gly-Asp-Gly-Glu** (13 mg, 28.3 µmol, 2.2 eq.) using the one-pot click procedure. The crude product was purified by preparative HPLC column chromatography (H_2O/MeCN + 0.1 % TFA; gradient). After lyophilization, the free phosphoric acid was obtained as a colourless solid (9.1 mg, 6.64 µmol, 53 %). ^{1}H-NMR (400 MHz, DMSO-*d₆*): δ = 1.74 – 1.85 (m, 1H), 1.93 – 2.03 (m, 1H), 2.20 (d, *J* = 6.6 Hz, 2H), 2.23 – 2.36 (m, 8H), 2.56 (d, *J* = 7.9 Hz, 1H), 2.67 – 2.76 (m, 1H), 2.93 (t, *J* = 5.9 Hz, 2H), 3.68 – 3.73 (m, 2H), 3.84 (d, *J* = 3.8 Hz, 2H), 4.09 (s, 6H), 4.24 (dd, *J* = 13.3, 8.6 Hz, 1H), 4.32 (d, *J* = 7.3 Hz, 4H), 4.58 (dd, *J* = 12.9, 7.2 Hz, 1H), 5.19 (s, 2H), 6.68 – 6.84 (m, 4H), 6.99 – 7.17 (m, 8H), 7.93 (d, *J* = 7.8 Hz, 1H), 7.98 (s, 1H), 8.16 (t, *J* = 5.6 Hz, 1H), 8.41 (d, *J* = 7.5 Hz, 1H), 8.57 (t, *J* = 5.0 Hz, 1H) ppm; ^{13}C NMR (101 MHz, DMSO-d_6): δ = 26.3, 29.9, 36.1, 42.0, 42.1, 48.0, 49.6, 50.3, 51.1, 51.5, 65.5, 67.8, 69.0, 116.8, 121.5, 124.3, 124.5, 141.0, 141.5, 142.9, 146.7, 147.0, 150.3, 165.9, 168.6, 170.8, 171.9, 173.1, 173.8 ppm; ^{31}P-NMR (162 MHz, DMSO-d_6): δ = -5.43, -5.94 ppm; HR MS (pos. ESI, MeOH): m/z calc. for $C_{61}H_{57}N_7O_{18}P_2$ $[M+2H]^+$: 619.6690, found: 619.6691.
Deprotonation of the phosphoric acid **4** (6.8 mg, 5.5 µmol, 1 eq.) with aq. NaOH (330 µL, 33 µmol, 6 eq.) delivered the hexasodium salt **4** as a colourless solid in quantitative yield (7.5 mg, 5.47 µmol, 99.7% quant.).^{1}H NMR (600 MHz, D_2O): δ = 1.89 (dt, *J* = 14.9, 8.5 Hz, 1H), 2.02 – 2.10 (m, 1H), 2.17 – 2.27 (m, 6H), 2.32 – 2.39 (m, 4H), 2.44 (d, *J* = 7.2 Hz, 2H), 2.70 (ddd, *J* = 24.2, 16.2, 6.6 Hz, 2H), 3.27 (s, 2H), 3.93 (dd, *J* = 36.0, 16.9 Hz, 2H), 4.07 (d, *J* = 3.4 Hz, 2H), 4.14 (dd, *J* = 16.0, 7.1 Hz, 5H), 4.30 (s, 2H), 4.52 (s, 2H), 4.69 (dd, *J* = 8.1, 5.5 Hz, 1H), 5.06 (s, 2H), 6.83 (s, 4H), 7.06 – 6.98 (m, 2H), 7.08 – 7.14 (m, 2H), 7.21 – 7.30 (m, 4H), 7.34 (s, 1H) ppm;^{13}C NMR (151 MHz, D_2O): δ = 26.3, 28.4, 34.1, 38.7, 42.5, 42.6, 48.2, 50.7, 51.7, 51.8, 55.2, 65.0, 68.4, 68.9, 116.4, 116.9, 121.2, 121.5, 124.9, 125.0, 140.9, 142.3, 144.9, 147.9, 148.1, 151.0, 168.4, 170.6, 170.9, 171.1, 174.1, 177.7, 182.2, 183.2 ppm; ^{31}P NMR (162 MHz, D_2O): δ = 0.73, -3.89 ppm; HR MS (pos. ESI, MeOH): m/z calc. for $C_{61}H_{57}N_7O_{18}P_2$ $[M+2H]^+$: 619.6690, found: 619.6690.

2) Synthesis of dimeric Molecular Tweeezers with functional Linkers

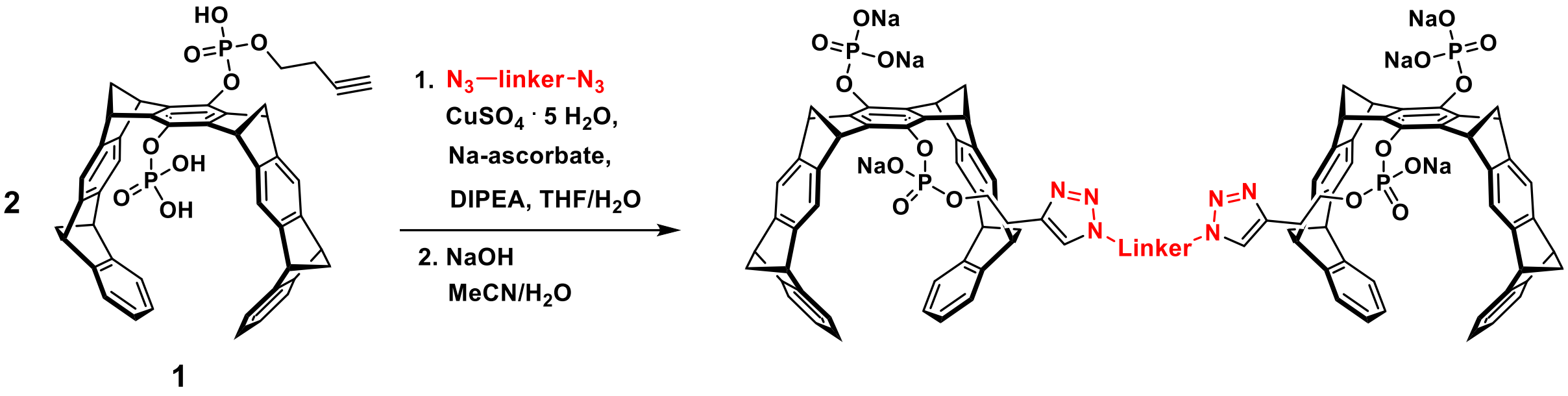

Figure S15: Iterative double click of 2 eq. butynyl MT 1 with 1 eq. diazidolinker followed by quantitative neutralization to water-soluble dimeric tweezer.

Dimeric tweezer 5:

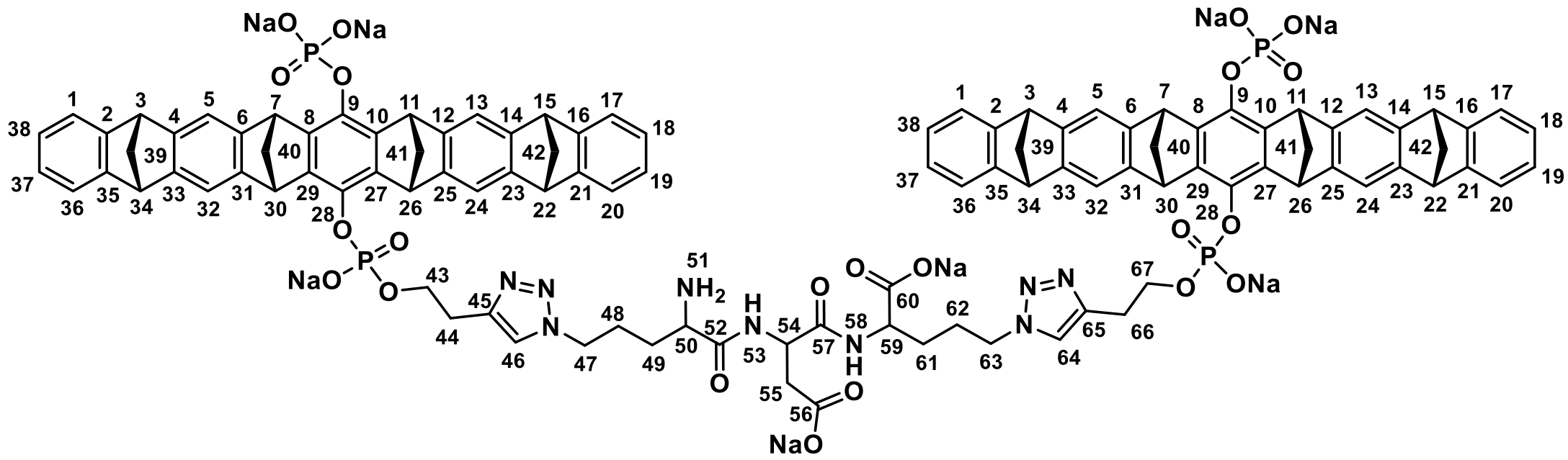

The phosphoric acid **5** was prepared from monobutinyl tweezer **1** (20 mg, 25.6 µmol, 2 eq.) and peptide linker **N_3-Orn-Asp-Orn-N_3** (5.31 mg, 12.8 µmol, 1 eq.) using the iterative click procedure. The crude product was purified by preparative HPLC column chromatography (H_2O/MeCN + 0.1 % TFA; gradient). After lyophilization, the free phosphoric acid was obtained as a colourless solid (6.6 mg, 3.2 µmol, 25 %).^{1}H NMR (600 MHz, DMSO-d_6): δ = 1.64 (dd, J = 14.0, 7.9 Hz, 1H), 1.67 – 1.75 (m, 2H,), 1.81 (dd, J = 13.6, 7.1 Hz, 1H), 1.85 – 1.96 (m, 3H), 1.97 – 2.03 (m, 1H), 2.17 (s, 4H), 2.21 – 2.34 (m, 12H), 2.56 – 2.62 (m, 1H), 2.66 – 2.82 (m, 4H), 2.87 – 2.95 (m, 1H), 3.78 – 3.85 (m, 1H), 3.91 – 3.95 (m, 2H), 4.09 (dd, J = 39.5, 11.4 Hz, 10H), 4.28 – 4.43 (m, 13H), 4.67 (dd, J = 12.3, 7.7 Hz, 1H), 6.68 – 6.83 (m, 8H), 6.97 – 7.07 (m, 8H), 7.08 – 7.15 (m, 8H), 7.95 – 8.10 (m, 2H), 8.29 (s, 2H), 8.35 (d, J = 7.4 Hz, 1H), 8.88 (d, J = 6.1 Hz, 1H) ppm; ^{13}C-NMR (151 MHz, DMSO-d_6): δ = 24.8, 26.5, 26.9, 28.0, 36.0, 48.0, 48.7, 49.9, 50.3, 51.4, 51.6, 65.1, 65.3, 67.8, 68.8, 116.7, 121.5, 122.7, 124.5, 141.03, 141.4, 143.4, 146.8, 146.9, 150.2, 168.3, 171.4, 173.1 ppm; ^{31}P NMR (162 MHz, DMSO-d_6): δ = -5.46, -6.14 ppm; HR MS (pos. ESI, MeOH): m/z calc. for $C_{106}H_{95}N_9O_{22}P_4$ [M+H]$^+$: 986.2860, found: 986.2870.
Deprotonation of the phosphoric acid **5** (6 mg, 2.88 µmol, 1 eq.) with aq. NaOH (23.2 µL, 23 µmol, 8 eq.) delivered the heptasodium salt **5** as a beige-coloured solid in quantitative yield (6.7 mg, 2.96 µmol, quant.). ^{1}H NMR (600 MHz, D_2O): δ = 1.28 (s, 4H), 1.79 (dt, J = 14.1, 6.9 Hz, 2H), 1.97 (s, 4H), 2.11 – 2.44 (m, 14H), 2.45 – 2.57 (m, 4H), 2.76 (ddd, J = 14.6, 13.3, 4.6 Hz, 2H), 3.17 (s, 1H), 3.50 – 3.77 (m, 2H), 3.86 – 4.17 (m, 10H), 4.34 (t, J = 40.7 Hz, 9H), 4.57 (s, 4H), 4.89 (d, J = 5.2 Hz, 1H), 6.38 – 7.51 (m, 26H) ppm; ^{13}C NMR (151 MHz, D_2O): δ = 26.5, 28.5, 29.2, 29.9, 34.1, 48.3, 49.9, 50.7, 53.4, 54.9, 61.6, 68.3, 69.6, 116.5, 119.0, 121.7, 124.6, 140.6, 142.1, 147.8, 148.3, 150.5, 162.5, 171.1, 172.3, 178.1 ppm; ^{31}P NMR (162 MHz, D_2O): δ = 0.99, -3.16 ppm; LR MS (pos. ESI, MeOH): m/z calc. for $C_{106}H_{95}N_9O_{22}P_4$ [M+2H]$^+$: 986.3, gefunden: 986.3.

Dimeric tweezer 6:

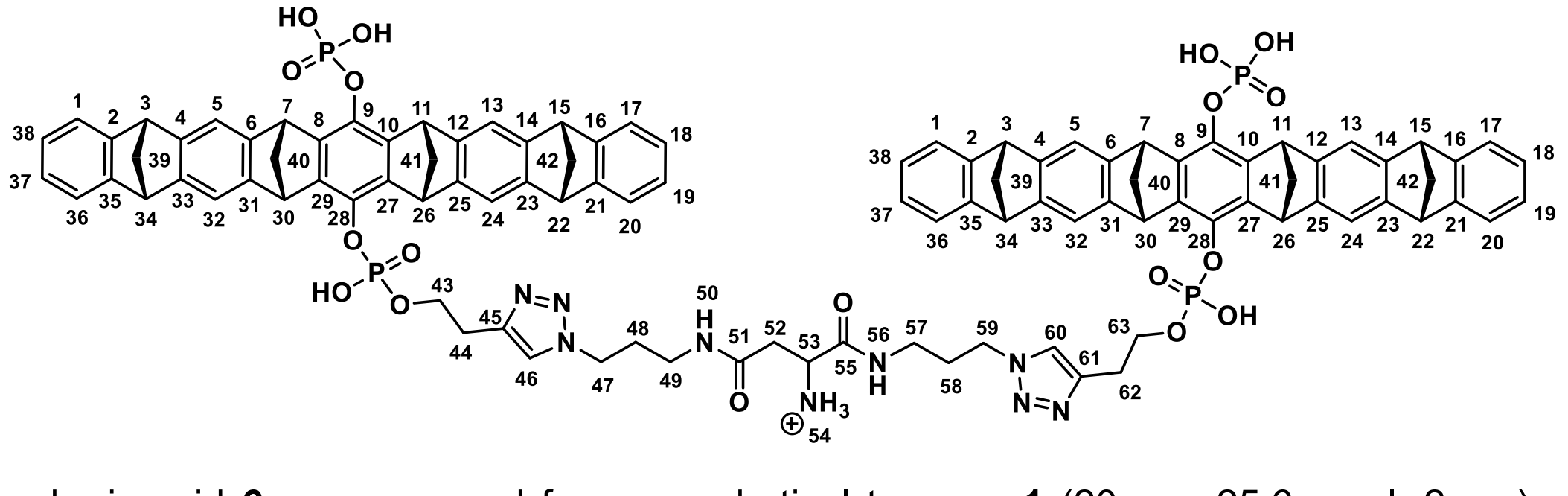

he phosphoric acid **6** was prepared from monobutinyl tweezer **1** (20 mg, 25.6 µmol, 2 eq.) and the cationic Asp peptide linker (5.28 mg, 12.8 µmol, 1 eq.) using the iterative click procedure. The crude product was purified by preparative HPLC column chromatography (H_2O/MeCN + 0.1 % TFA; gradient). After lyophilization, the free phosphoric acid was obtained as a colourless solid (9.9 mg, 8.93 µmol, 58 %).^{1}H NMR (600 MHz, DMSO-*d6*): δ = 1.92 – 2.04 (m, 4H), 2.17 (d, *J* = 6.0 Hz, 4H), 2.27 (dd, *J* = 15.6, 7.0 Hz, 12H), 2.65 – 2.91 (m, 6H), 3.11 (d, *J* = 4.2 Hz, 3H), 3.15 – 3.22 (m, 1H), 3.93 – 4.01 (m, 4H), 4.06 (d, *J* = 13.1 Hz, 9H), 4.32 (d, *J* = 10.3 Hz, 8H), 4.35 – 4.44 (m, 4H), 6.77 (s, 8H), 7.04 (dd, *J* = 31.6, 6.2 Hz, 8H), 7.10 (d, *J* = 7.5 Hz, 8H), 8.01 (d, *J* = 6.3 Hz, 2H), 8.19 (s, 3H), 8.43 (s, 1H), 8.58 (s, 1H) ppm; ^{13}C NMR (151 MHz, DMSO-*d6*): δ = 26.8, 29.5, 29.6, 35.9, 46.8, 47.0, 48.0, 49.5, 50.3, 65.4, 67.8, 68.9, 116.8, 121.5, 122.9, 124.5, 136.2, 141.0, 141.5, 143.2, 146.7, 146.9, 150.2, 168.0, 168.4 ppm;.^{31}P NMR (162 MHz, DMSO-*d6*): δ = -5.43, -5.87 ppm; HR MS (neg. ESI, MeOH): m/z calc. für $C_{102}H_{91}N_9O_{18}P_4$ [M-2H]$^+$: 926.2649, found: 926.2657.

Deprotonation of the phosphoric acid **6** (17 mg, 8.96 µmol, 1 eq.) with aq. NaOH (54 µL, 54 µmol, 6 eq.) delivered the hexasodium salt **6** as a colourless solid in quantitative yield (19.1 mg, 9.44 µmol, quant.). ^{1}H NMR (600 MHz, D_2O+DMSO-*d6*): δ = 2.01 (d, *J* = 5.8 Hz, 4H), 2.19 (d, *J* = 16.7 Hz, 8H), 2.27 – 2.45 (m, 6H), 2.85 (d, *J* = 60.2 Hz, 8H), 3.50 (s, 1H), 3.96 (dd, *J* = 29.5, 21.3 Hz, 12H), 4.07 (s, 4H), 4.34 – 4.46 (m, 8H), 6.73 (s, 8H), 6.95 (dd, *J* = 24.0, 15.2 Hz, 12H), 7.06 (d, *J* = 5.5 Hz, 4H), 7.86 (d, *J* = 1.4 Hz, 2H) ppm; ^{13}C NMR (151 MHz, D_2O+DMSO-*d6*): δ = 28.01, 30.3, 37.3, 41.7, 48.7, 49.1, 51.7, 53.2, 65.7, 69.1, 70.0, 117.8, 122.9, 125.0, 126.1, 137.4, 148.4, 149.3, 149.9, 152.0 ppm; ^{31}P NMR (243 MHz, D_2O+DMSO-*d6*): δ = 0.38, -4.10 ppm; HR MS (pos. ESI, MeOH): m/z calc. for $C_{102}H_{91}N_9O_{18}P_4$ [M+H]$^+$: 928.2805, found: 928.2808.

Dimeric bisphosponate tweezer 7:

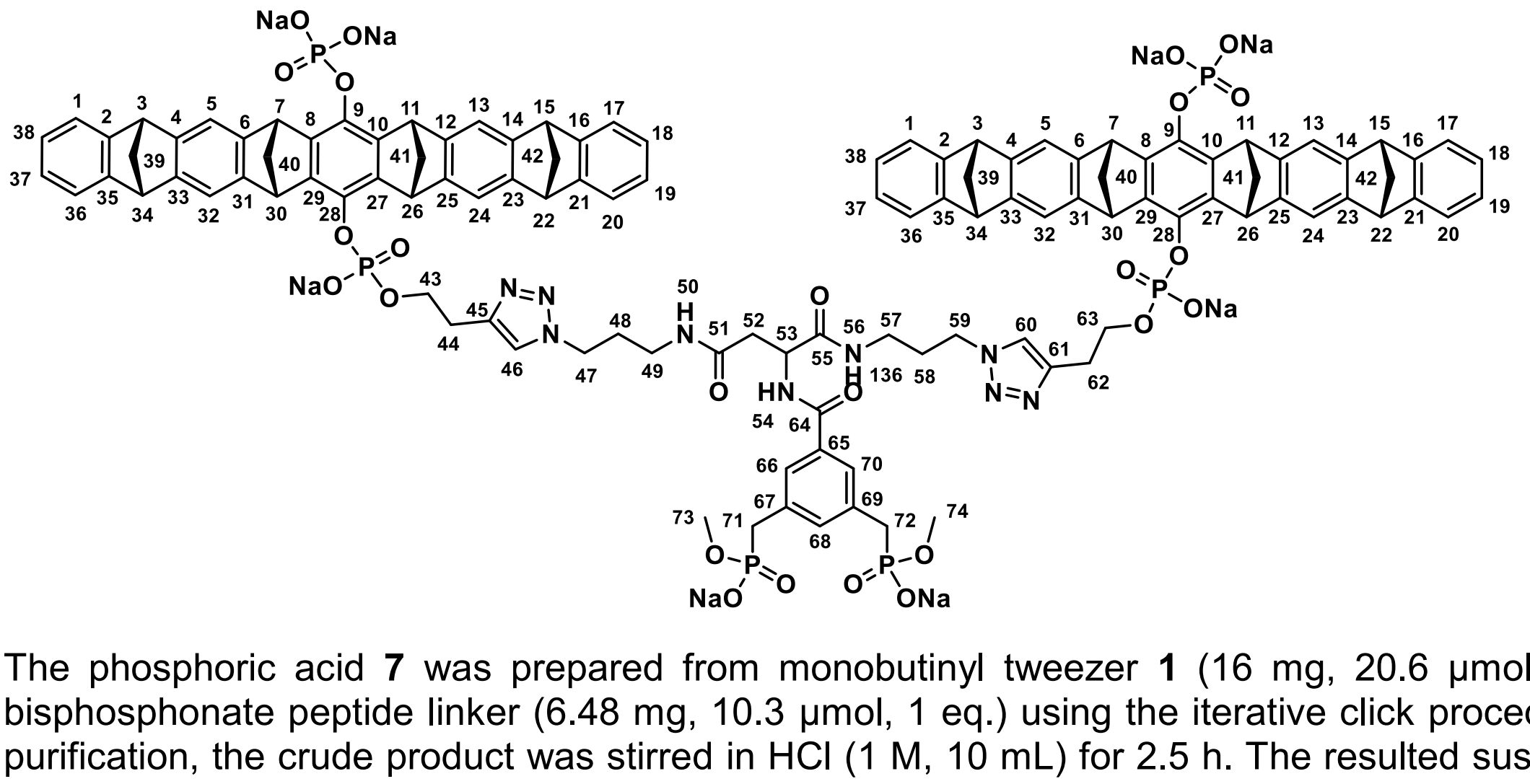

The phosphoric acid **7** was prepared from monobutinyl tweezer **1** (16 mg, 20.6 µmol, 2 eq.) and bisphosphonate peptide linker (6.48 mg, 10.3 µmol, 1 eq.) using the iterative click procedure. For the purification, the crude product was stirred in HCl (1 M, 10 mL) for 2.5 h. The resulted suspension was transferred into a falcon tube and was centrifuged for 15 min at 4400 rpm. After decanting the supernatant, this washing step was repeated three times with HCl (1 M, 3 x 10 mL), twice with distilled water (2x 10 mL) and twice with Diethylether (2 x 10 mL). The purified product was then dissolved in MeCN/H_2O and transferred back into a separate flask. After solvent removal under vacuum and overnight lyophilization, the free phosphoric acid was obtained as a beige-coloured solid (14.2 mg, 6.53 µmol, 64 %).^{1}H NMR (600 MHz, DMSO-*d6*): δ = 1.88 – 2.03 (m, 4H), 2.20 (d, *J* = 6.3 Hz, 4H), 2.28 (d, *J* = 13.7 Hz, 12H), 2.65 (ddd, *J* = 22.5, 14.6, 6.6 Hz, 2H), 2.93 (s, 4H), 3.10 (d, *J* = 21.0 Hz, 8H), 3.53 (d,

J = 10.7 Hz, 6H), 4.08 (s, 8H), 4.10 – 4.16 (m, 4H), 4.30 (d, *J* = 23.1 Hz, 8H), 4.36 (dd, *J* = 13.4, 6.3 Hz, 4H), 4.78 (dd, *J* = 13.8, 7.3 Hz, 1H), 6.73 – 6.80 (m, 8H), 7.02 – 7.08 (m, 8H), 7.10 (s, 8H), 7.34 (s, 1H), 7.65 (s, 2H), 7.95 – 8.10 (m, 3H), 8.15 (s, 1H), 8.49 (d, *J* = 7.5 Hz, 1H) ppm; ^{13}C NMR (151 MHz, DMSO-*d*$_6$): δ = 26.8, 29.9, 32.5, 33.4, 35.9, 37.6, 47.0, 48.0, 50.3, 50.9, 51.7, 65.8, 67.8, 69.0, 116.9, 121.5, 123.0, 124.5, 126.9, 133.3, 134.0, 135.9, 141.0, 141.6, 142.8, 146.7, 147.0, 150.3, 166.0, 169.7, 171.1 ppm; ^{31}P NMR (162 MHz, DMSO-*d*$_6$): δ = 24.08, -5.43, -5.88 ppm; HR MS (pos. ESI, MeOH): m/z calc. for $C_{113}H_{105}N_9O_{25}P_6$ [M+2H]$^+$: 1121.2642, found: 1121.2538.
Deprotonation of the phosphoric acid **7** (14.2 mg, 6.53 µmol, 1 Äq.) with aq. NaOH (52.2 µL, 52 µmol, 8 Äq.) delivered the octasodium salt **7** as a beige-coloured solid in quantitative yield (15.8 mg, 6.72 µmol, quant.).^{1}H NMR (400 MHz, D_2O+DMSO-*d*$_6$): δ = 1.82 – 2.08 (m, 8H), 2.18 (s, 8H), 2.31 (d, *J* = 4.8 Hz, 4H), 2.79 (dt, *J* = 59.3, 40.8 Hz, 14H), 3.29 (d, *J* = 10.3 Hz, 6H), 3.97 (s, 12H), 4.10 (d, *J* = 16.3 Hz, 8H), 4.47 (s, 4H), 4.72 (s, 1H), 6.73 (s, 8H), 6.89 – 7.10 (m, 16H), 7.22 (s, 1H), 7.42 (s, 2H), 7.89 (s, 2H) ppm; ^{13}C NMR (151 MHz, D_2O+DMSO-*d*$_6$): δ = 28.0, 30.5, 34.4, 35.3, 37.4, 38.7, 48.6, 49.0, 51.5, 52.5, 52.6, 65.5, 69.0, 69.9, 117.6, 122.8, 124.8, 126.0, 126.9, 134.2, 135.5, 137.3, 139.5, 141.4, 142.2, 143.0, 145.8, 147.7, 148.1, 149.2, 149.6, 150.1, 151.9,170.2, 171.9, 173.1 ppm; ^{31}P NMR (162 MHz, D_2O+DMSO-*d*$_6$): δ = 19.58, 2.42, -4.09 ppm; HR MS (neg. ESI, MeOH): m/z calc. for $C_{113}H_{105}N_9O_{25}P_6$ [M-2H]$^+$: 1086.2767, found: 1086.2760.

Dimeric NTA tweezer 8:

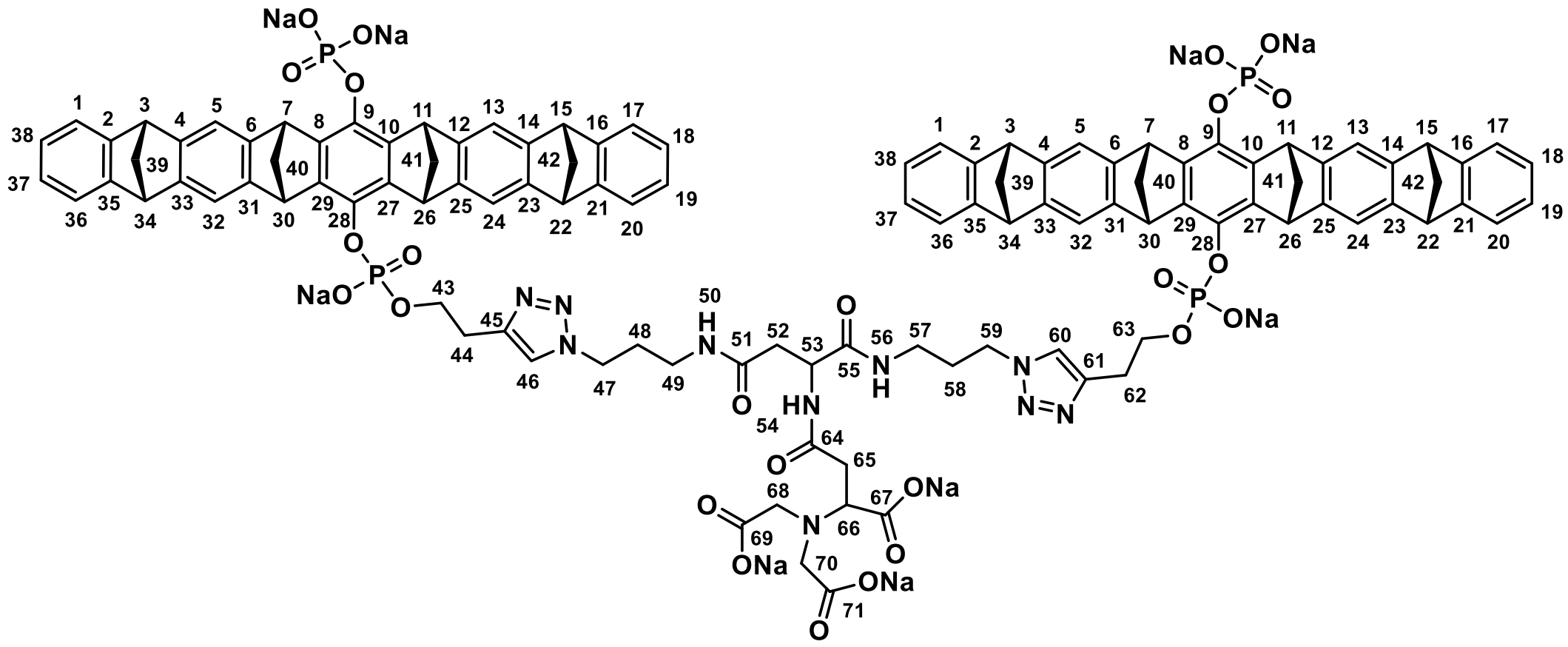

The phosphoric acid **8** was prepared from monobutinyl tweezer **1** (16 mg, 20.6 µmol, 2 eq.) and NTA peptide linker (6.11 mg, 10.3 µmol, 1 eq.) using the iterative click procedure. For the purification, the crude product was stirred in HCl (1 M, 10 mL) for 2.5 h. The resulted suspension was transferred into a falcon tube and was centrifuged for 15 min at 4400 rpm. After decanting the supernatant, this washing step was repeated three times with HCl (1 M, 3 x 10 mL), twice with distilled water (2x 10 mL) and twice with Diethylether (2 x 10 mL). The purified product was then dissolved in MeCN/H_2O and transferred back into a separate flask. After solvent removal under vacuum and overnight lyophilization, the free phosphoric acid was obtained as a colourless solid (17 mg, 8.15 µmol, 79 %).^{1}H NMR (600 MHz, DMSO-*d*$_6$): δ = 1.91 – 2.02 (m, 4H), 2.20 (d, *J* = 6.8 Hz, 4H), 2.29 (dd, *J* = 11.7, 8.0 Hz, 12H), 2.40 – 2.49 (m, 1H), 2.51 – 2.77 (m, 3H), 2.94 (t, *J* = 6.4 Hz, 4H), 3.09 (dd, *J* = 15.1, 5.7 Hz, 4H), 3.34 – 3.59 (m, 4H), 3.73 – 3.89 (m, 1H), 4.08 (s, 8H), 4.13 (dd, *J* = 13.8, 6.9 Hz, 4H), 4.27 – 4.40 (m, 12H), 4.52 – 4.65 (m, 1H), 6.74 – 6.80 (m, 8H), 7.02 – 7.13 (m, 16H), 7.79 – 7.97 (m, 1H), 8.03 (dd, *J* = 18.5, 4.2 Hz, 3H), 8.30 – 8.60 (m, 1H) ppm; ^{13}C NMR (151 MHz, DMSO-*d*$_6$): δ = 26.8, 30.0, 35.9, 37.6, 47.0, 48.0, 50.3, 52.4, 53.4, 61.4, 65.8, 67.8, 69.1, 116.8, 121.5, 123.0, 124.5, 135.9, 136.4, 141.0, 141.7, 146.6, 147.0, 150.3, 169.6, 170.8, 172.7, 172.9, 173.2 ppm; ^{31}P NMR (243 MHz, DMSO-*d*$_6$): δ = -5.43, -5.85 ppm; HR-MS (neg. ESI, MeOH): m/z calc. for $C_{110}H_{100}N_{10}O_{25}P_4$ [M-2H]$^+$: 1041.7849, found: 1041.7841.
Deprotonation of the phosphoric acid **8** (16.8 mg, 8.05 µmol, 1 eq.) with aq. NaOH (64.4 µL, 64 µmol, 9 eq.) delivered the nonasodium salt **8** as a colourless solid in quantitative yield (18 mg, 7.88 µmol, 98 %).^{1}H NMR (600 MHz, D_2O+DMSO-*d*$_6$): δ = 2.19 (d, *J* = 34.9 Hz, 4H), 2.32 (d, *J* = 5.3 Hz, 4H), 2.50 (d, *J* = 12.1 Hz, 8H), 2.57 – 2.73 (m, 6H), 2.82 – 2.97 (m, 2H), 3.01 – 3.51 (m, 12H), 3.70 (dd, *J* = 22.3, 15.2 Hz, 1H), 4.24 (s, 4H), 4.29 (d, *J* = 8.0 Hz, 8H), 4.35 (s, 4H), 4.51 (d, *J* = 7.1 Hz, 4H), 4.72 (s, 4H), 4.81 – 4.88 (m, 1H), 7.01 – 7.08 (m, 8H), 7.21 – 7.33 (m, 12H), 7.37 (d, *J* = 8.1 Hz, 4H), 8.20 (d, *J* = 13.0 Hz, 2H) ppm; ^{13}C NMR (151 MHz, D_2O+DMSO-*d*$_6$): δ = 29.2, 31.7, 38.8, 41.2, 50.2, 52.8, 58.7, 60.5, 66.8, 70.3, 71.2, 118.6, 119.2, 124.1, 126.3, 127.3, 138.5, 141.8, 142.8, 144.4, 147.1, 149.6, 150.4, 150.8, 153.2, 173.7, 174.7, 177.5, 180.6, 181.5 ppm; ^{31}P-NMR (243 MHz, D_2O+DMSO-*d*$_6$): δ = 2.69, -

4.10 ppm; HR MS (neg. ESI, MeOH): m/z calc. for $C_{110}H_{100}N_{10}O_{25}P_4$ [M-2H]$^+$: 1041.7849, found: 1041.7847.

Dimeric distilben-disulfonate tweezer 9:

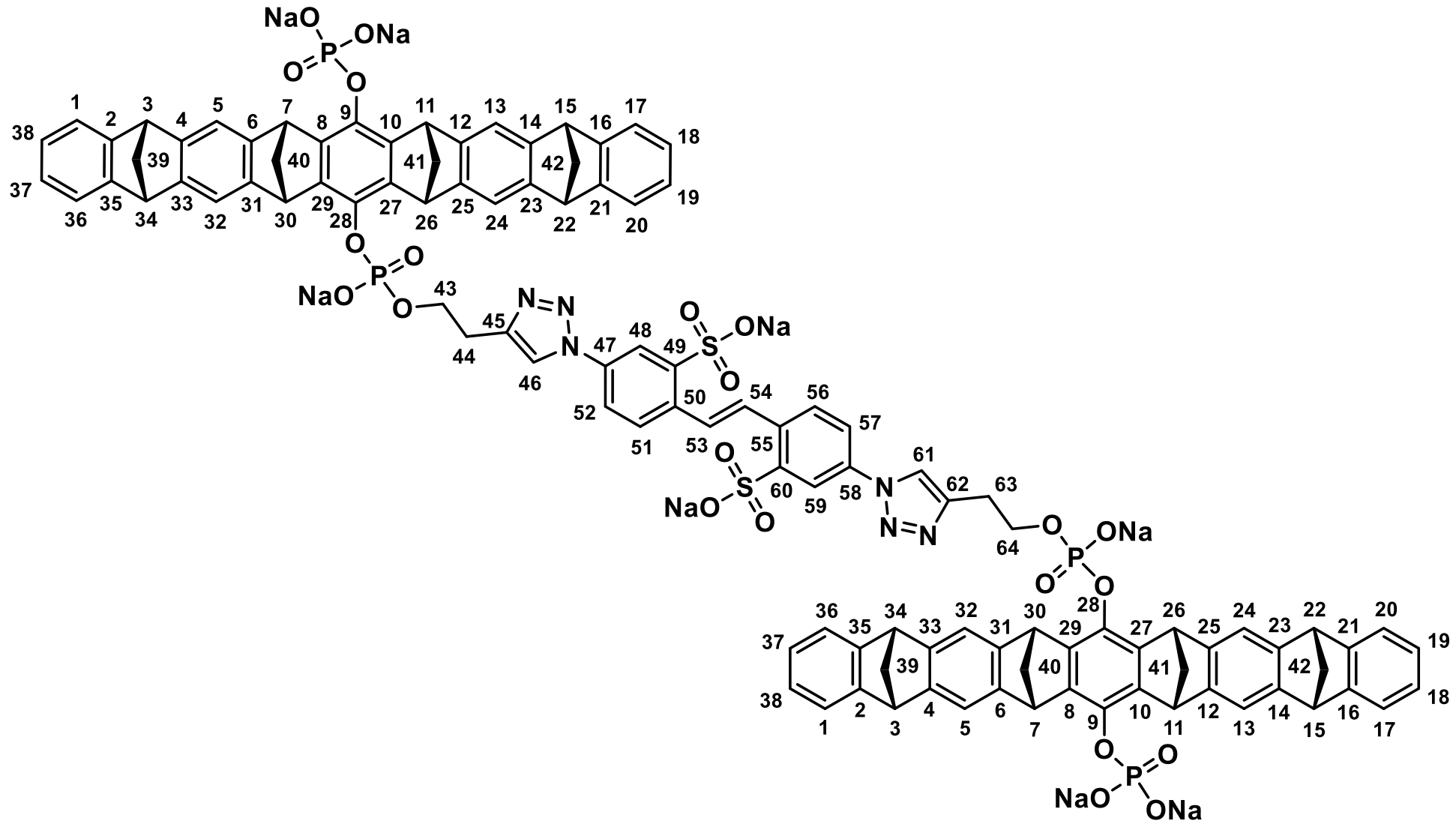

The phosphoric acid **9** was prepared from monobutinyl tweezer **1** (10 mg, 12.8 µmol, 2 eq.) and commercially available Disodium *4*,*4'*-diazidostilbene-2,2'-disulfonate *tetrahydrate* (3.45 mg, 6.4 µmol, 1 eq.) using the iterative click procedure. The whole synthesis and work-up was performed in the absence of light. The crude product was purified by preparative HPLC column chromatography (MeCN/H_2O/5% THF + 0.1% TFA). After lyophilization, the free phosphoric acid was obtained as a beige-coloured solid 7.2 mg (3.64 µmol, 57 %). ^{1}H NMR (600 MHz, DMSO-d_6): δ = 2.21 (d, *J* = 6.9 Hz, 4H), 2.29 (d, *J* = 5.0 Hz, 12H), 3.02 (t, *J* = 6.5 Hz, 4H), 4.09 (d, *J* = 12.8 Hz, 8H), 4.19 (dd, *J* = 13.9, 6.8 Hz, 4H), 4.31 (d, *J* = 14.3 Hz, 8H), 6.75 – 6.79 (m, 8H), 7.04 – 7.08 (m, 8H), 7.12 (d, *J* = 17.2 Hz, 8H), 7.81 – 7.99 (m, 4H), 8.22 – 8.39 (m, 4H), 8.77 (s, 2H) ppm;^{13}C NMR (151 MHz, DMSO-d_6): δ = 26.8, 48.0, 50.3, 65.6, 67.8, 69.0, 116.8, 118.5, 120.1, 121.1, 121.5, 124.5, 127.0, 127.6, 134.8, 135.9, 136.4, 141.0, 141.7, 144.3, 146.6, 147.1, 150.3 ppm; ^{31}P NMR (162 MHz, DMSO-d_6): δ = -5.36, -5.69 ppm; HR MS (pos. ESI, MeOH): m/z calc. for $C_{106}H_{82}N_6O_{22}P_4S_2$ [M+2H]$^+$: 990.7026, found: 990.7010.
Deprotonation of the phosphoric acid **9** (9.9 mg, 8.93 µmol, 1 eq.) with aq. NaOH (283 µL, 28.3 µmol, 8 eq.) delivered the octasodium salt **9** as a beige-coloured solid in quantitative yield (8.3 mg, 3.85 µmol, quant.).^{1}H NMR (600 MHz, D_2O+DMSO-d_6): δ = 1.76 (s, 4H), 2.25 (s, 12H), 3.05 (s, 4H), 3.98 (d, *J* = 13.4 Hz, 12H), 4.15 (s, 4H), 4.51 (d, *J* = 14.5 Hz, 4H, H-26, H-30), 6.73 (s, 8H), 6.89 – 7.12 (m, 16H), 7.79 (s, 4H), 8.09 (s, 2H), 8.28 (s, 2H), 8.74 (s, 2H) ppm, ^{13}C NMR (151 MHz, D_2O+DMSO-d_6): δ = 27.6, 47.9, 50.5, 63.5, 67.8, 68.5, 116.0, 118.5, 120.4, 121.6, 124.8, 127.4, 127.9, 134.6, 135.1, 137.4, 140.2, 141.1, 146.5, 148.5, 150.8 ppm; ^{31}P NMR (243 MHz, D_2O+DMSO-d_6): δ = 5.91, -4.65 ppm; HR MS (neg. ESI, MeOH): m/z calc. for $C_{106}H_{82}N_6O_{22}P_4S_2$ [M-2H]$^+$: 988.6880, found: 988.6871.

Dimeric diphenyl-disulfone tweezer 11:

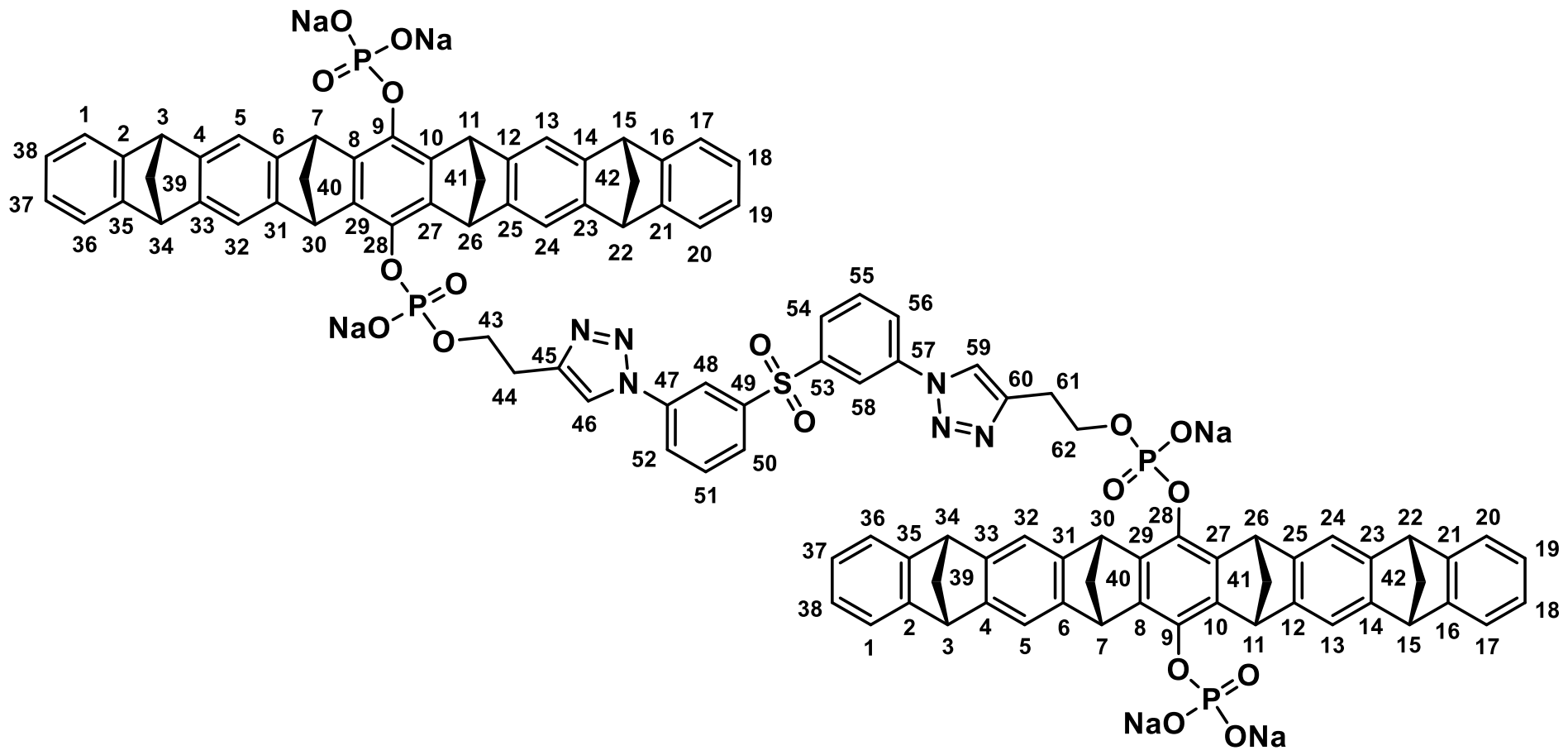

The phosphoric acid **11** was prepared from monobutinyl tweezer **1** (10 mg, 12.8 µmol, 2 eq.) and commercially available 3,3-Diazidodiphenyl sulfone (1.93 mg, 6.4 µmol, 1 eq.) using the iterative click procedure. The crude product was purified by preparative HPLC column chromatography (MeCN/H_2O/5% THF + 0.1% TFA). After lyophilization, the free phosphoric acid was obtained as a colourless solid (6.6 mg, 3.55 µmol, 55 %). ^{1}H NMR (600 MHz, DMSO-d_6): δ = 2.15 (d, J = 6.7 Hz, 4H), 2.21 – 2.29 (m, 12H), 3.07 (t, J = 6.5 Hz, 4H), 4.04 (d, J = 21.2 Hz, 8H), 4.20 – 4.33 (m, 12H), 6.72 – 6.78 (m, 8H), 7.01 – 7.09 (m, 16H), 7.88 (t, J = 8.1 Hz, 2H), 8.16 (d, J = 8.1 Hz, 2H), 8.26 – 8.33 (m, 2H), 8.59 (s, 2H), 8.97 (s, 2H) ppm; ^{13}C NMR (151 MHz, DMSO-d_6): δ = 26.8, 48.0, 50.3, 65.4, 67.8, 69.0, 116.8, 118.4, 121.5, 121.7, 124.4, 125.1, 127.5, 131.9, 136.0, 136.4, 137.4, 140.9, 141.6, 142.2, 144.8, 146.6, 146.9, 150.2 ppm; ^{31}P NMR (243 MHz, DMSO-d_6): δ = -5.46, -5.92 ppm; HR MS (neg. ESI, MeOH): m/z calc. for $C_{104}H_{80}N_6O_{18}P_4S$ [M-2H]$^+$: 927.7044, found: 927.7043.

Deprotonation of the phosphoric acid **11** (6.5 mg, 3.50 µmol, 1 eq.) with aq. NaOH (210 µL, 21.0 µmol, 6 eq.) delivered the hexasodium salt 11 as a colourless solid in quantitative yield (6.8 mg, 3.42 µmol, 98%).^{1}H NMR (400 MHz, D_2O+DMSO-d_6): δ = 1.93 (d, J = 5.0 Hz, 4H), 2.13 (d, J = 17.4 Hz, 8H), 2.36 (s, 4H), 3.02 (s, 4H), 3.89 (d, J = 27.8 Hz, 8H), 4.14 (d, J = 18.1 Hz, 8H), 4.58 (s, 4H), 6.71 (s, 8H), 6.99 (dd, J = 35.0, 29.4 Hz, 16H), 7.84 (t, J = 7.9 Hz, 2H), 7.94 (d, J = 7.1 Hz, 2H), 8.23 (d, J = 7.5 Hz, 2H), 8.39 (s, 2H), 8.87 (s, 2H) ppm; ^{13}C NMR (151 MHz, D_2O+DMSO-d_6): δ = 29.2, 47.9, 50.5, 63.9, 67.8, 68.8, 116.1, 118.6, 121.7, 122.0, 124.7, 125.5, 127.5, 132.5, 137.7, 140.4, 141.3, 142.2, 146.4, 146.5, 148.62, 150.8 ppm; ^{31}P NMR (162 MHz, D_2O+DMSO-d_6): δ = 0.97, -3.14 ppm; HR MS (neg. ESI, MeOH): m/z calc. for $C_{104}H_{80}N_6O_{18}P_4S$ [M-2H]$^+$: 927.7044, found: 927.7039.

NMR spectra of all new dimeric tweezers (^1H, ^{13}C and ^{31}P)

A Peptide Tweezers 2-4

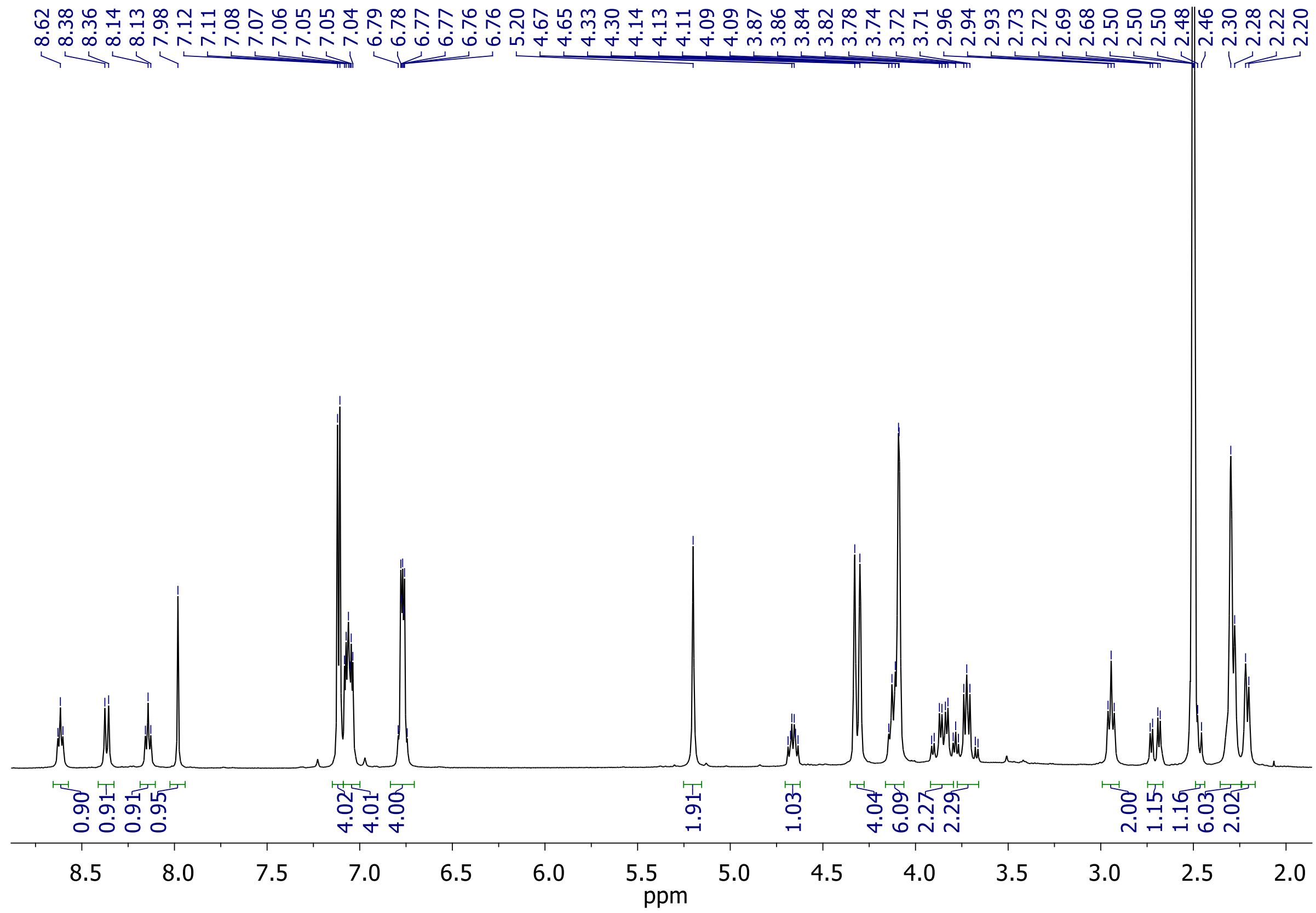

^1H NMR spectrum of peptide tweezer **2** (400 MHz, DMSO-d_6, RT)

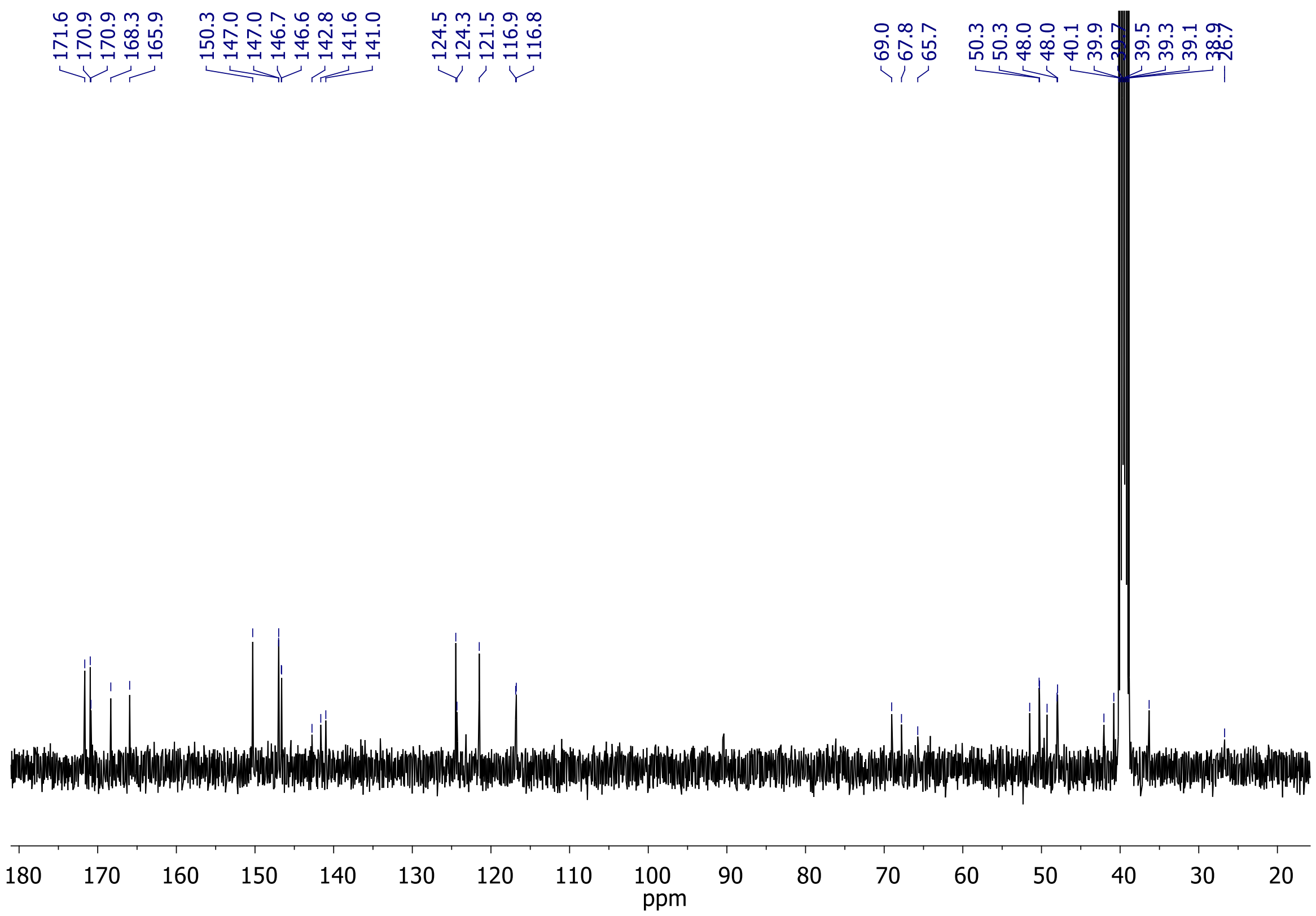

^{13}C NMR spectrum of peptide tweezer **2** (101 MHz, DMSO-d_6, RT)

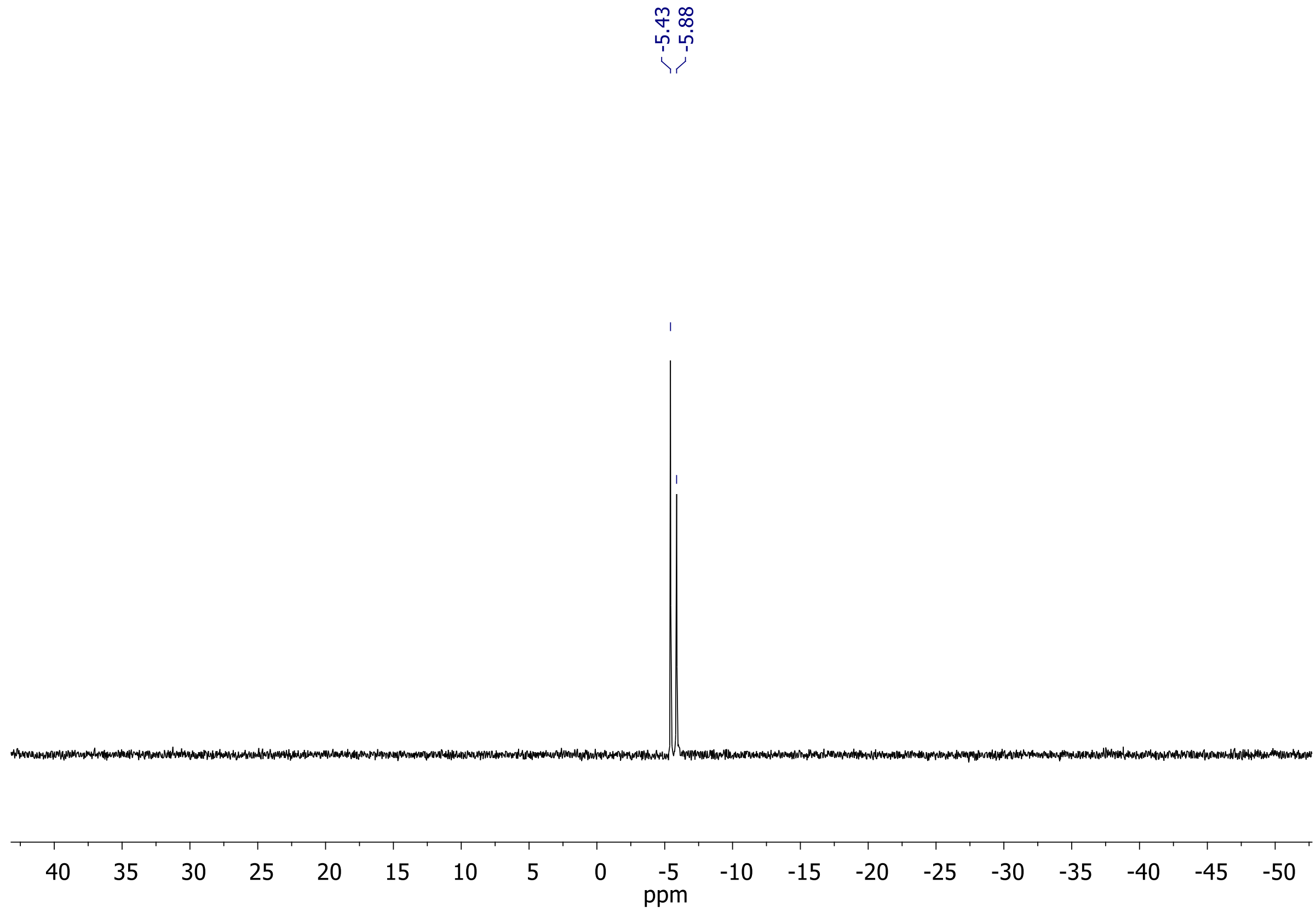

^{31}P NMR spectrum of peptide tweezer **2** (162 MHz, DMSO-d_6, RT)

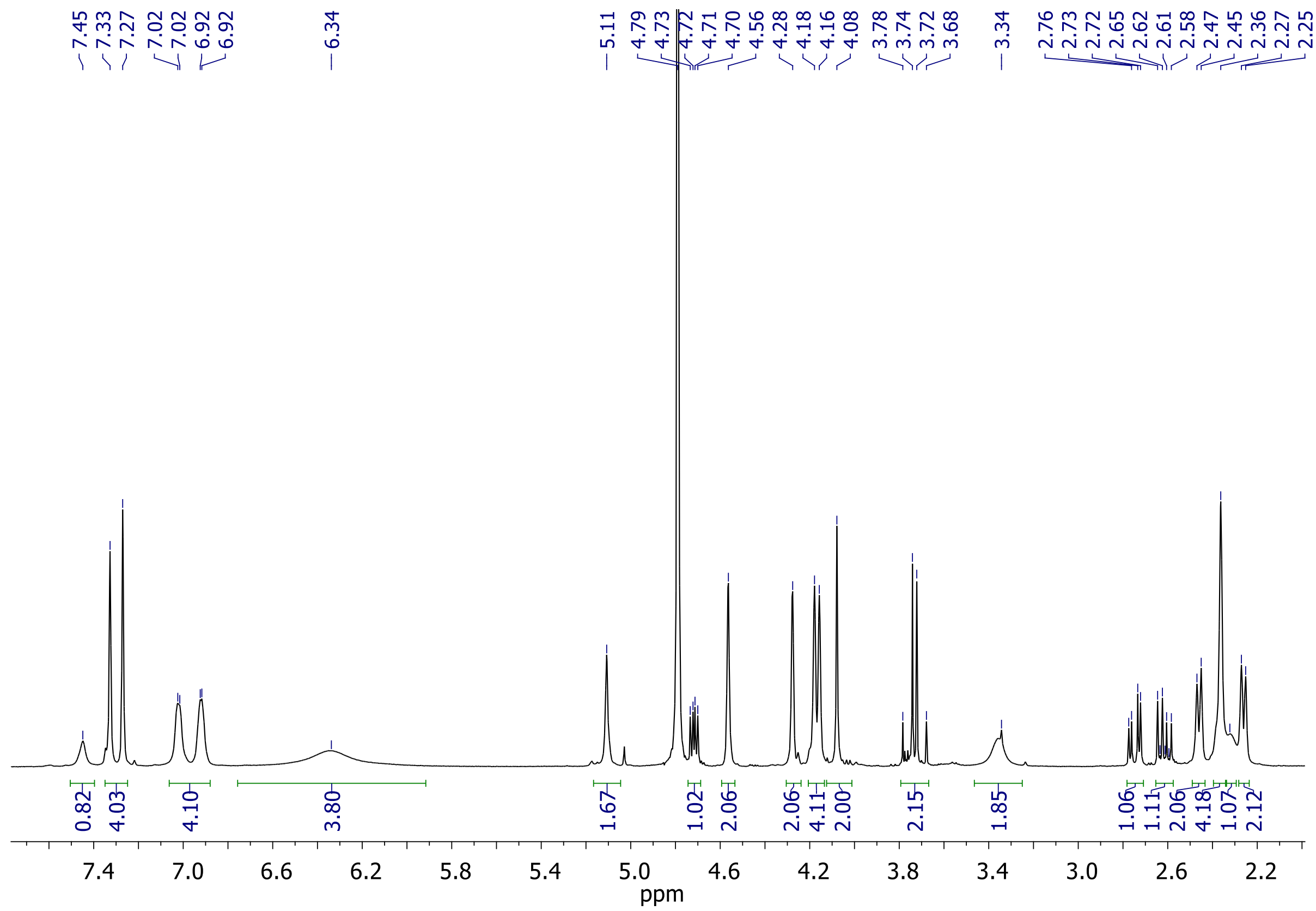

^{1}H NMR spectrum of peptide tweezer **2** (Na salt) (400 MHz, D_2O, RT)

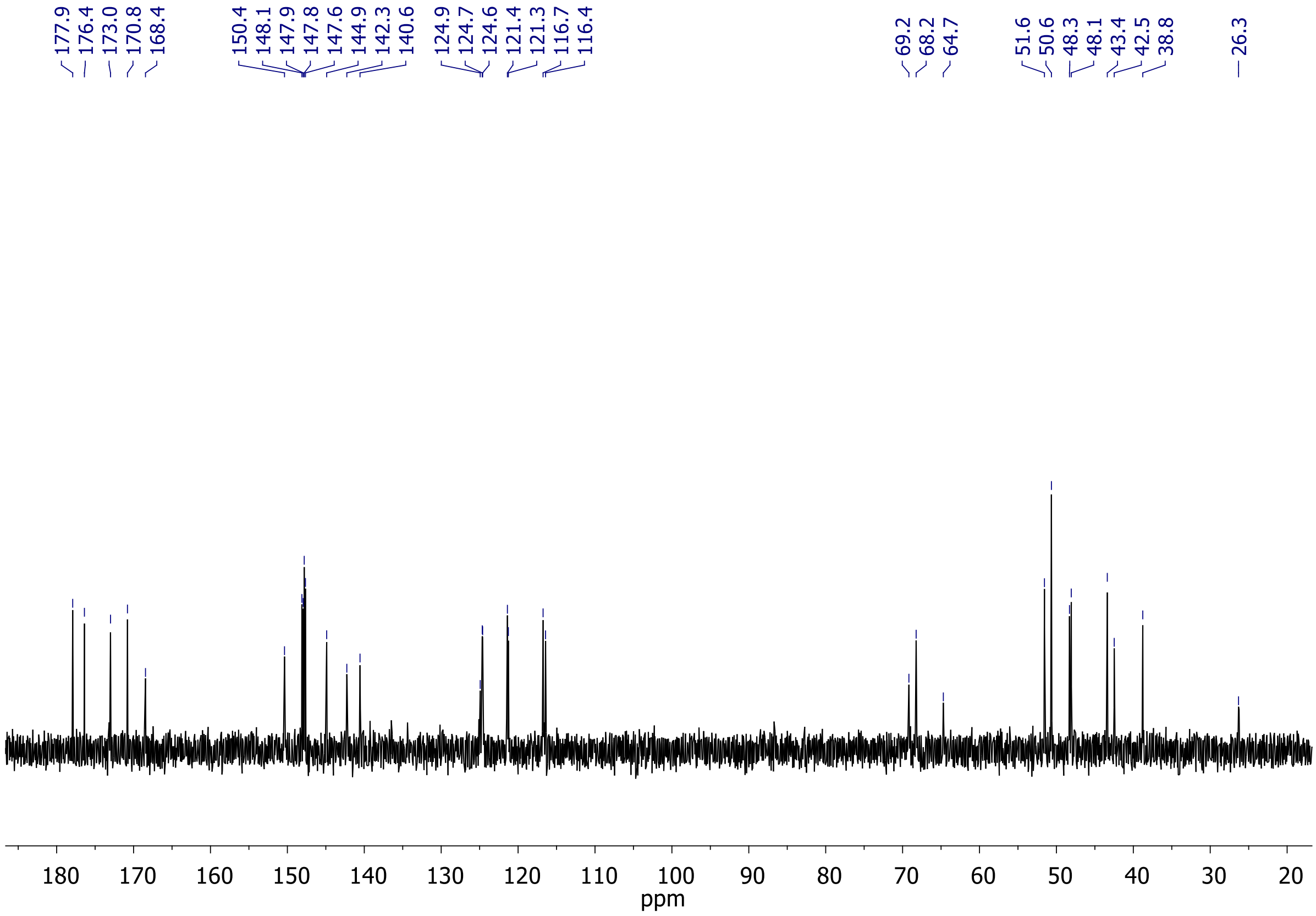

^{13}C NMR spectrum of peptide tweezer **2** (Na salt) (101 MHz, D_2O, RT)

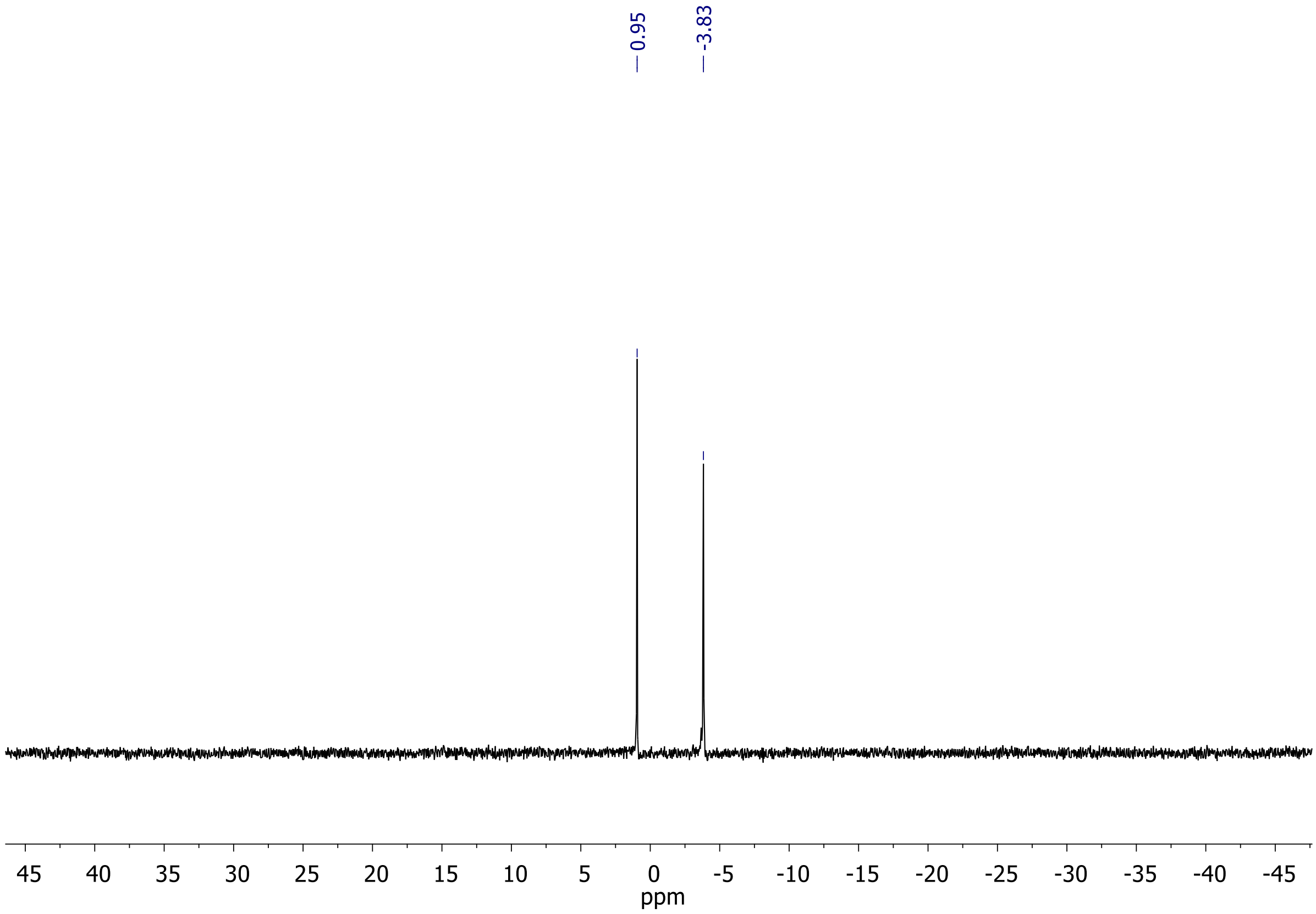

^{31}P NMR spectrum of peptide tweezer **2** (Na salt) (162 MHz, D_2O, RT)

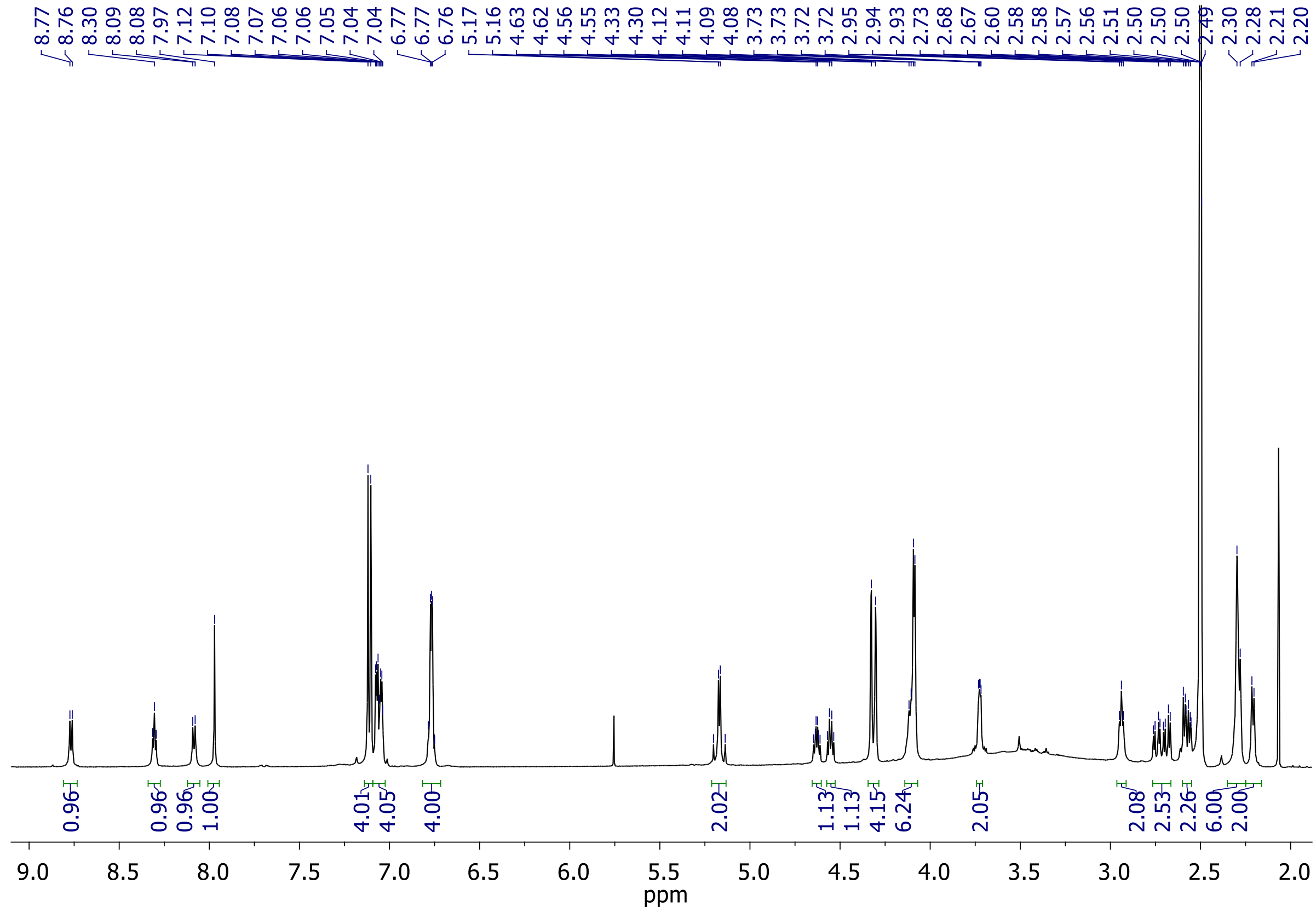

^{1}H NMR spectrum of peptide tweezer **3** (600 MHz, DMSO-d_6, RT)

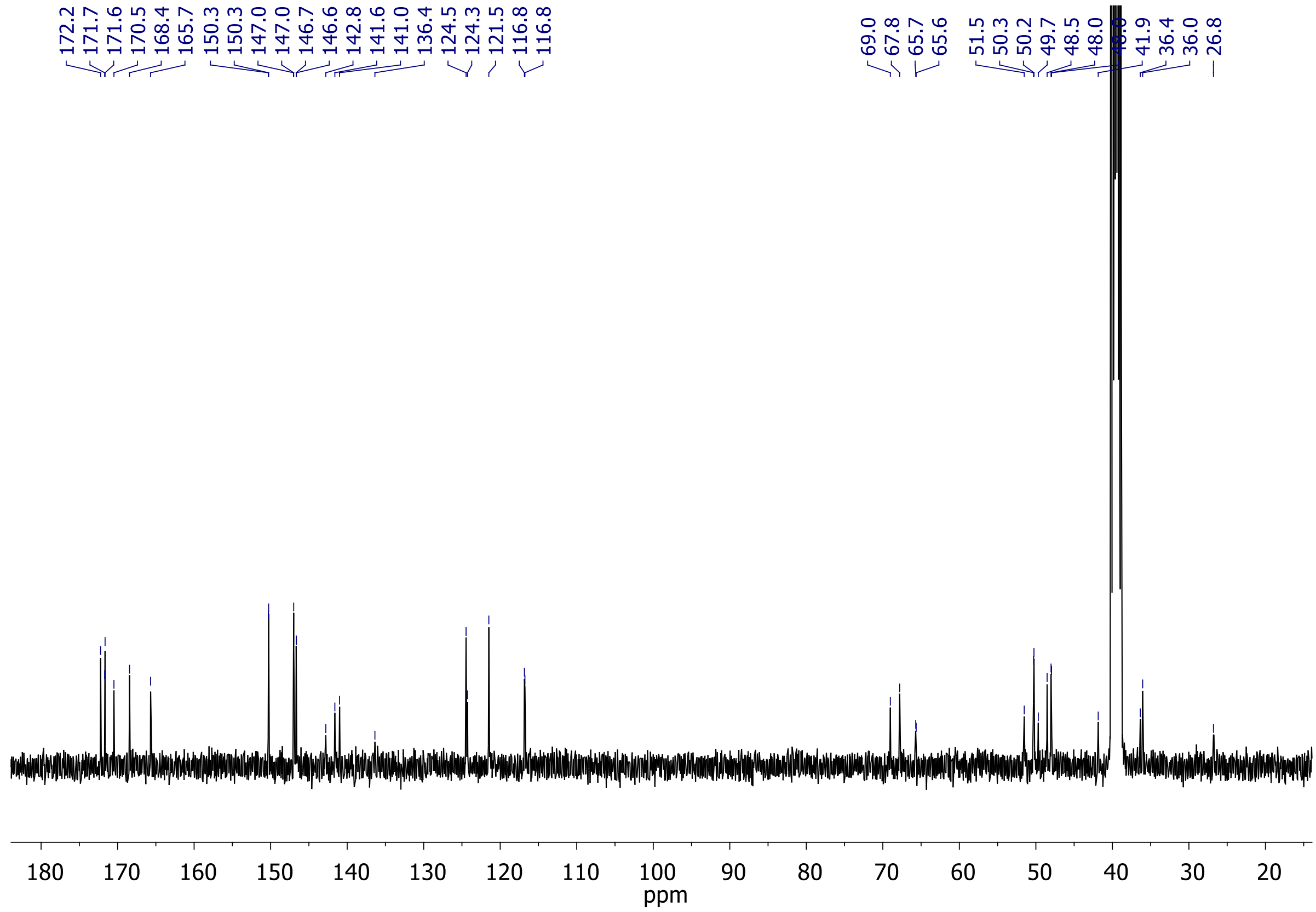

^{13}C NMR spectrum of peptide tweezer **3** (101 MHz, DMSO-d_6, RT)

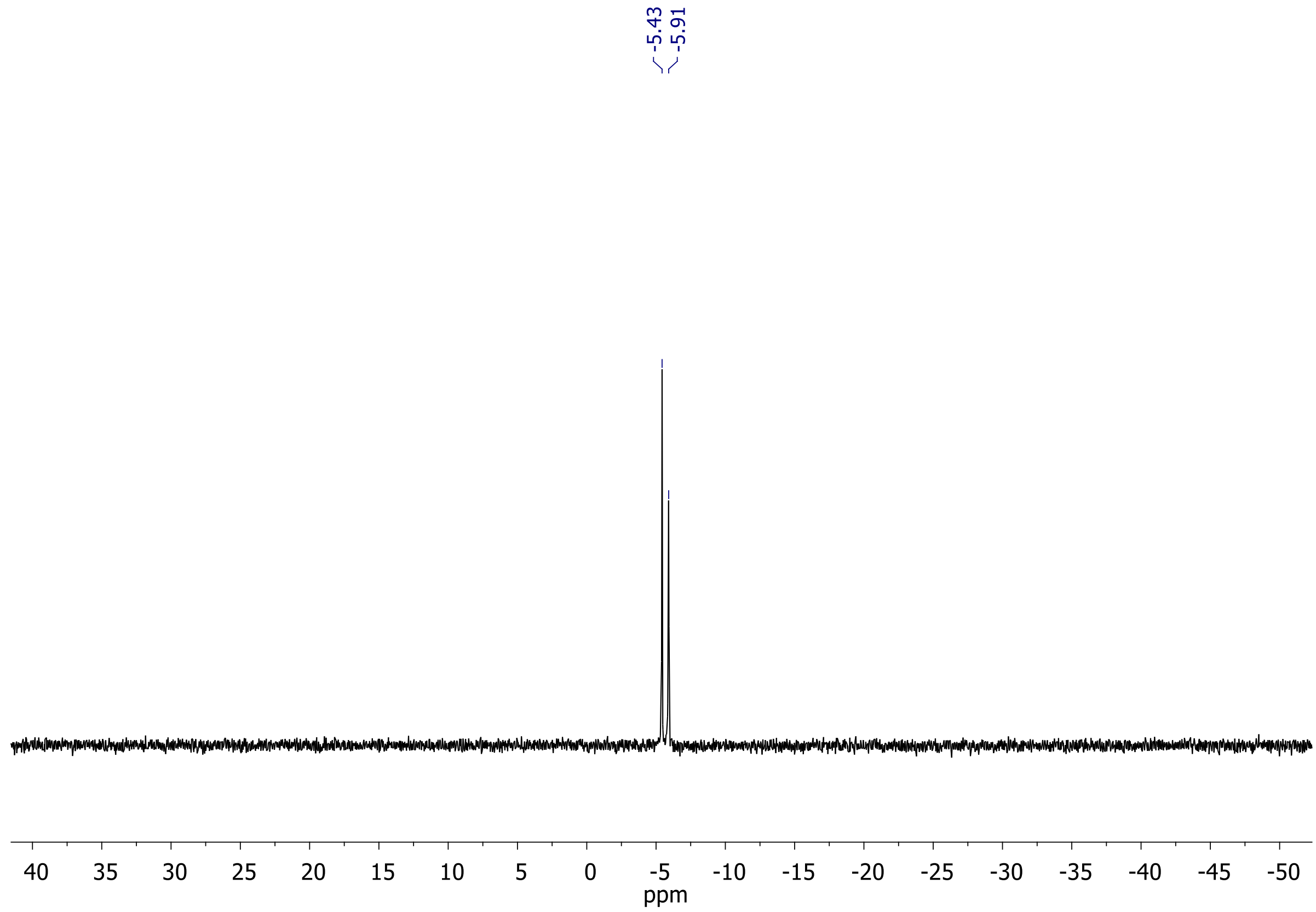

^{31}P NMR spectrum of peptide tweezer **3** (162 MHz, DMSO-d_6, RT)

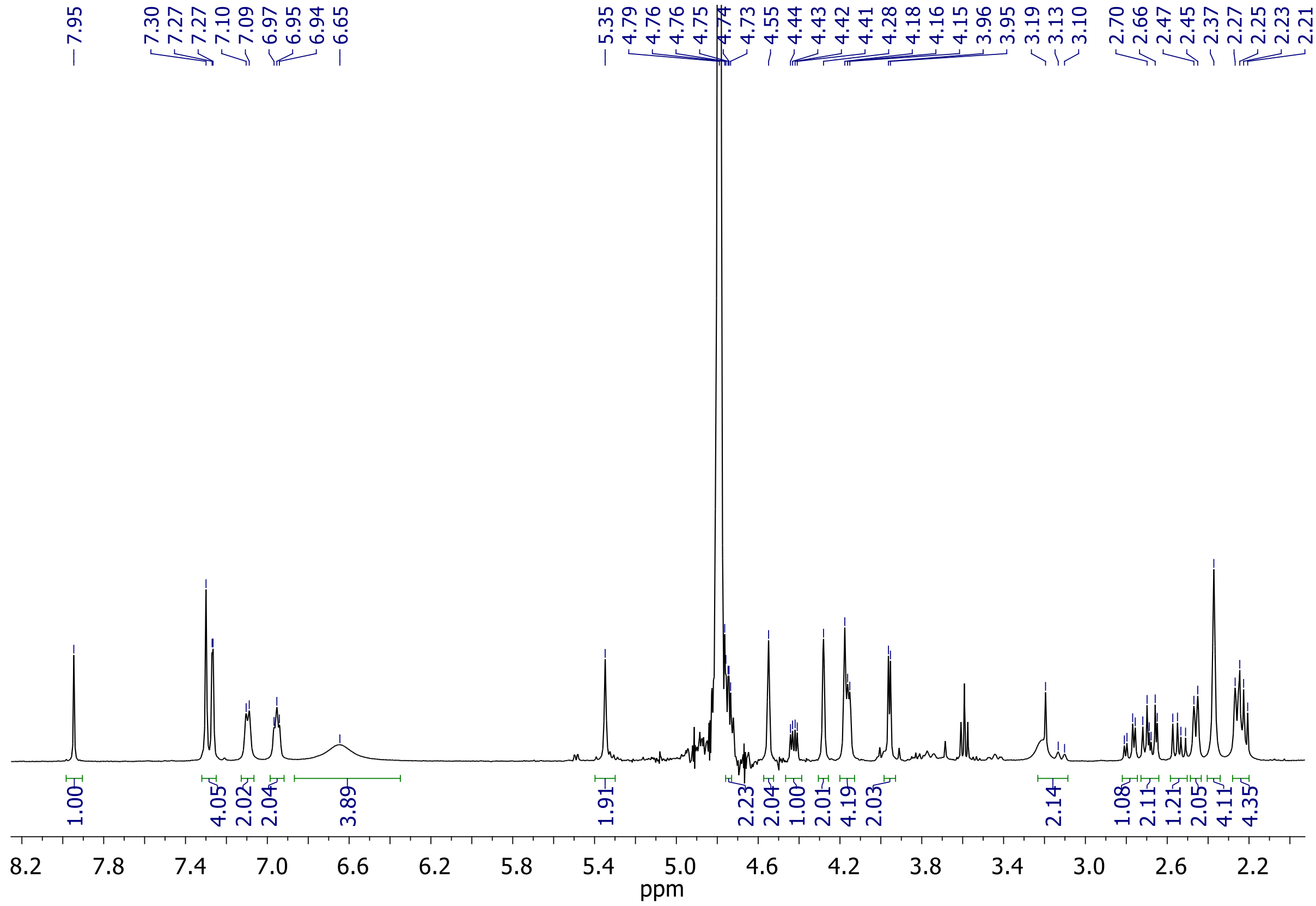

^{1}H NMR spectrum of peptide tweezer **3** (Na salt) (400 MHz, D_2O, RT)

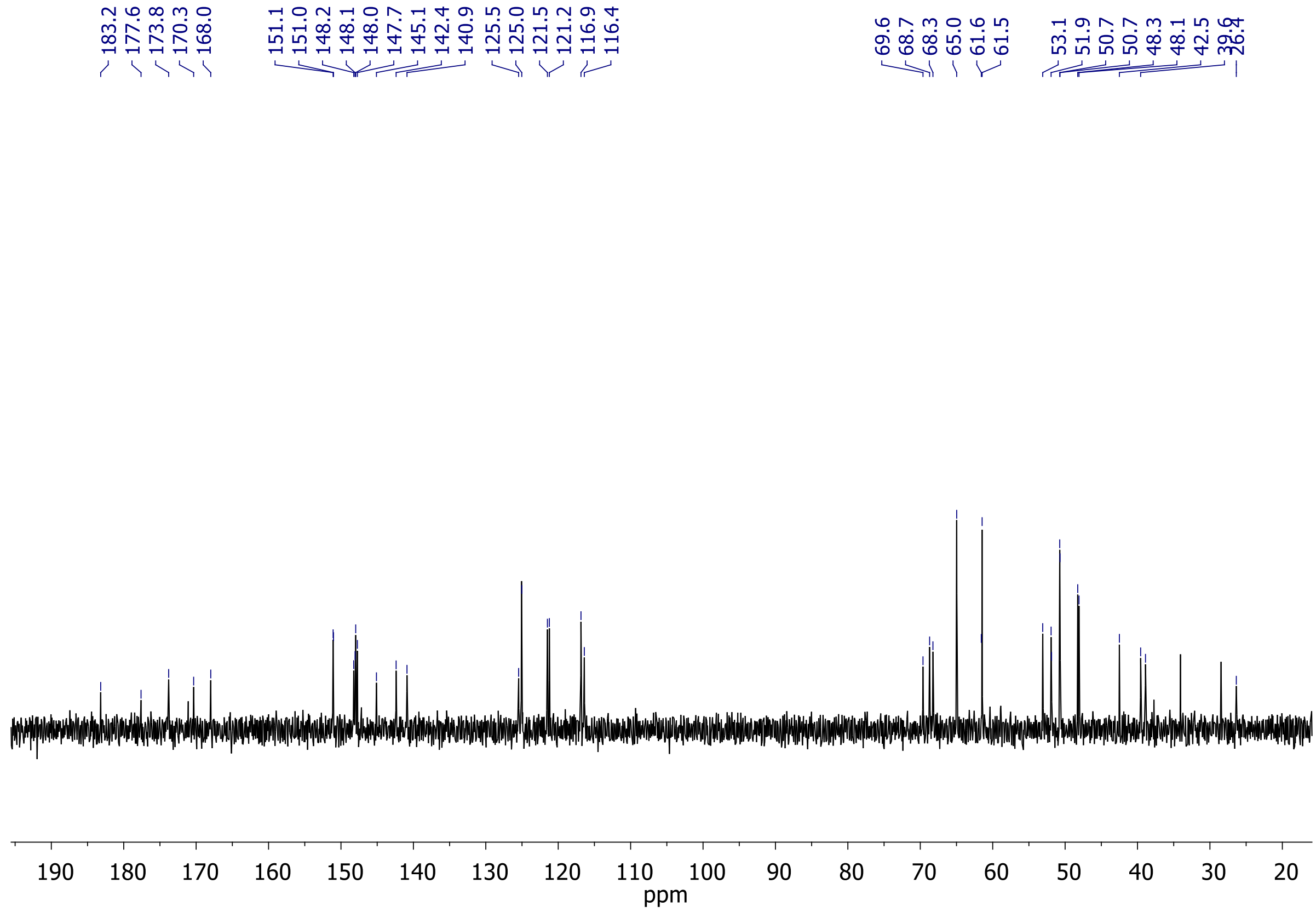

^{13}C NMR spectrum of peptide tweezer **3** (Na salt) (151 MHz, D_2O, RT)

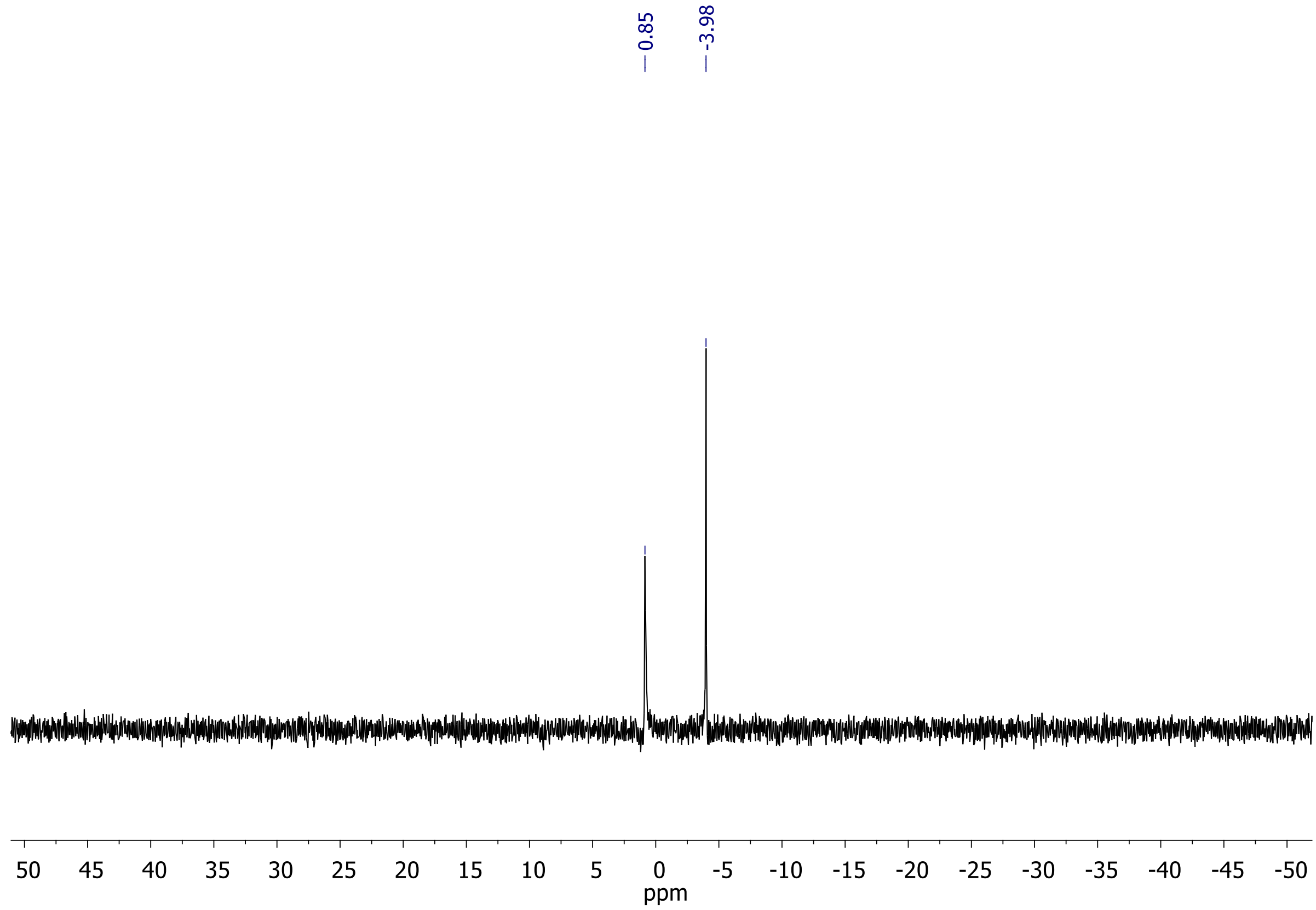

^{31}P NMR spectrum of peptide tweezer **3** (Na salt) (162 MHz, D_2O, RT)

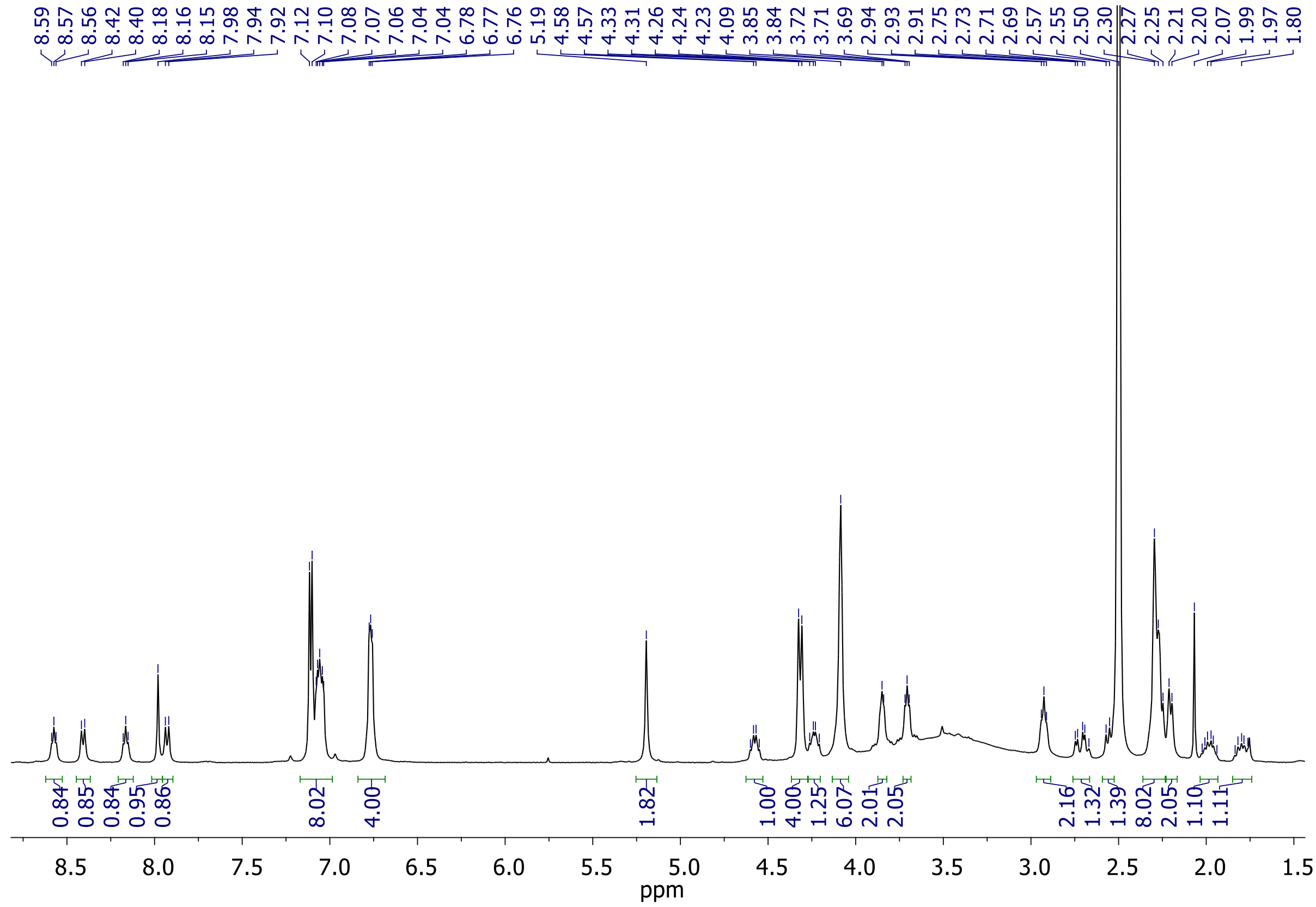

^{1}H NMR spectrum of peptide tweezer **4** (400 MHz, DMSO-d_6, RT)

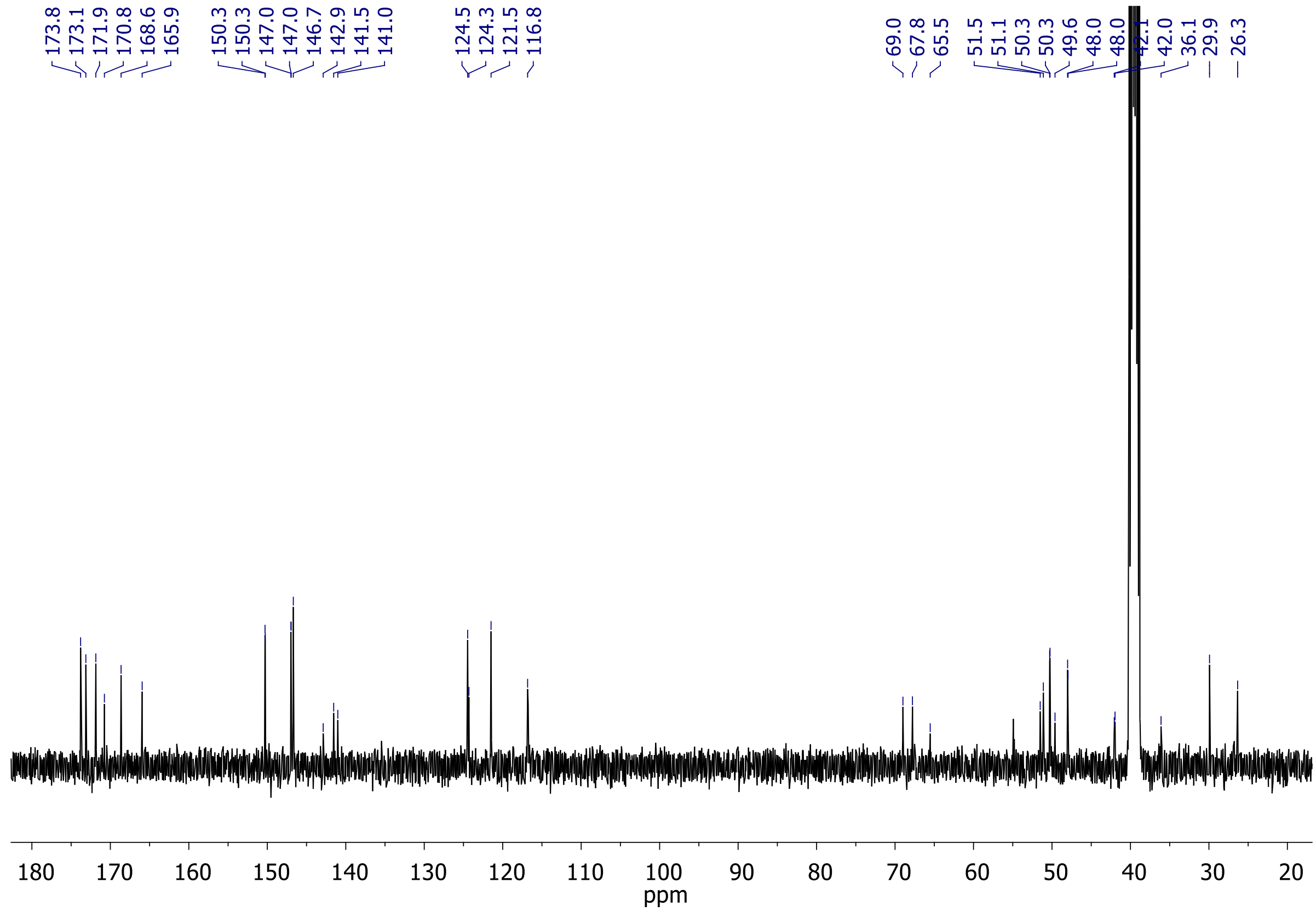

^{13}C NMR spectrum of peptide tweezer **4** (101 MHz, DMSO-d_6, RT)

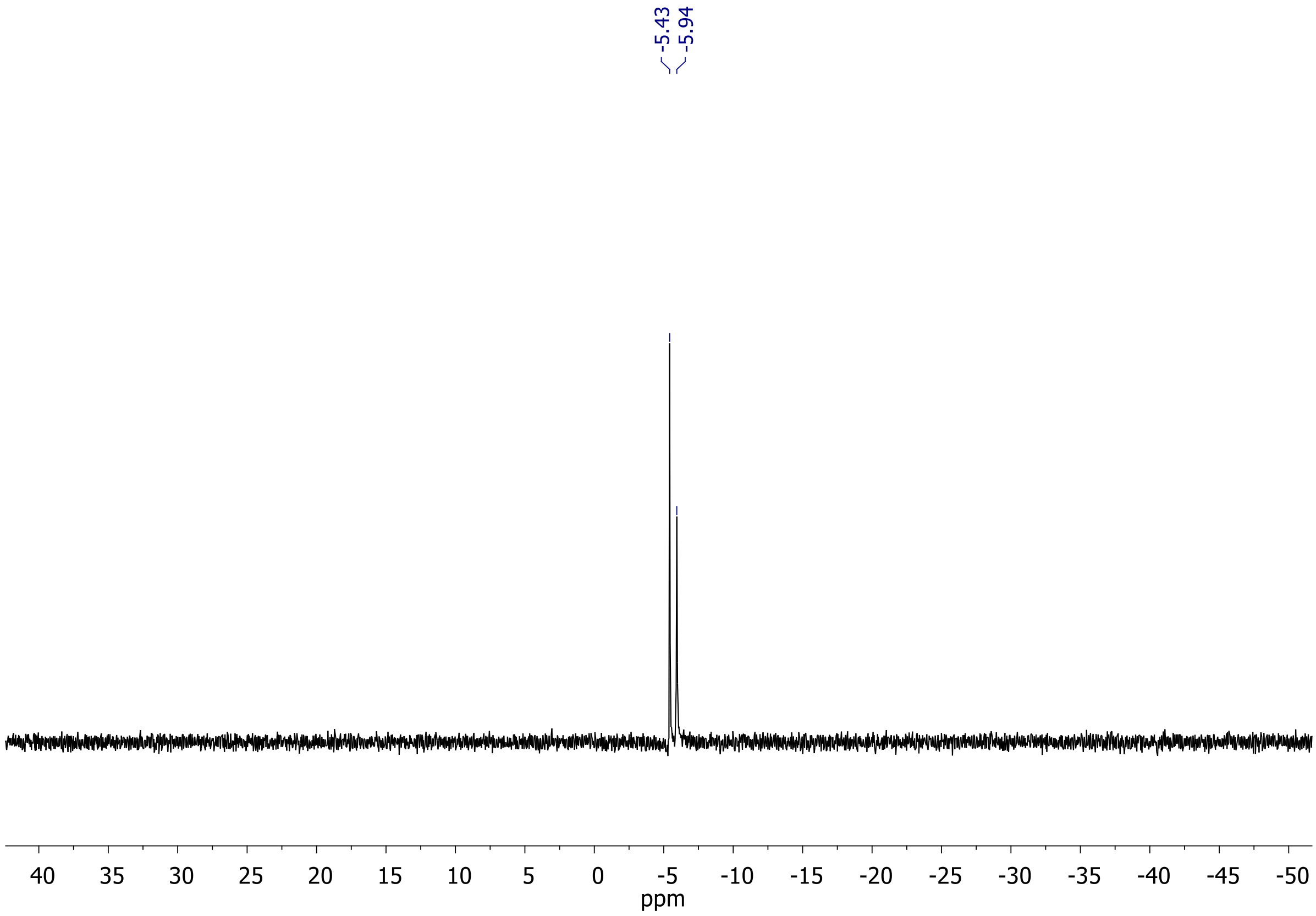

^{31}P NMR spectrum of peptide tweezer **4** (162 MHz, DMSO-d_6, RT)

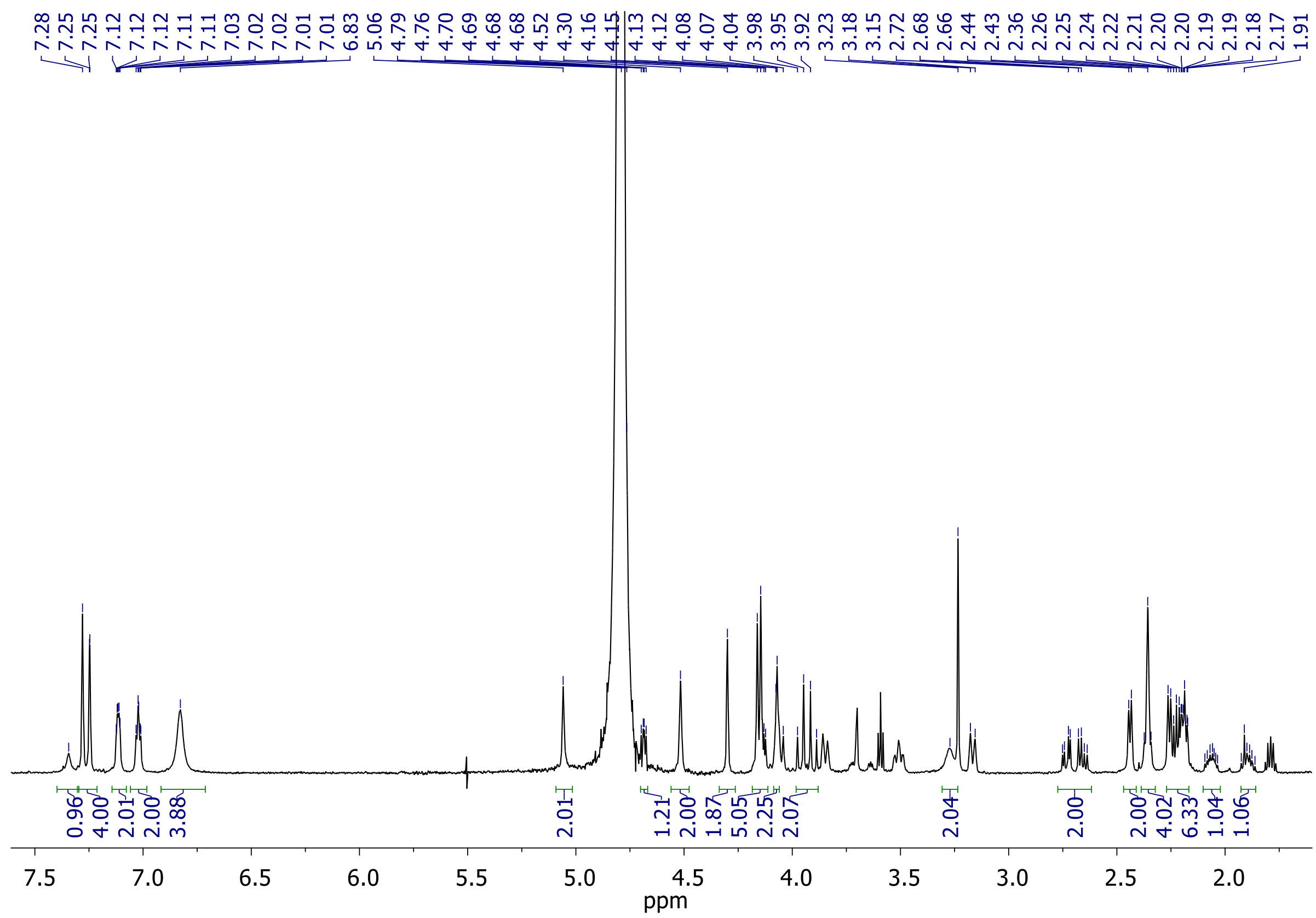

^1H NMR spectrum of peptide tweezer **4** (Na salt) (600 MHz, D_2O, RT)

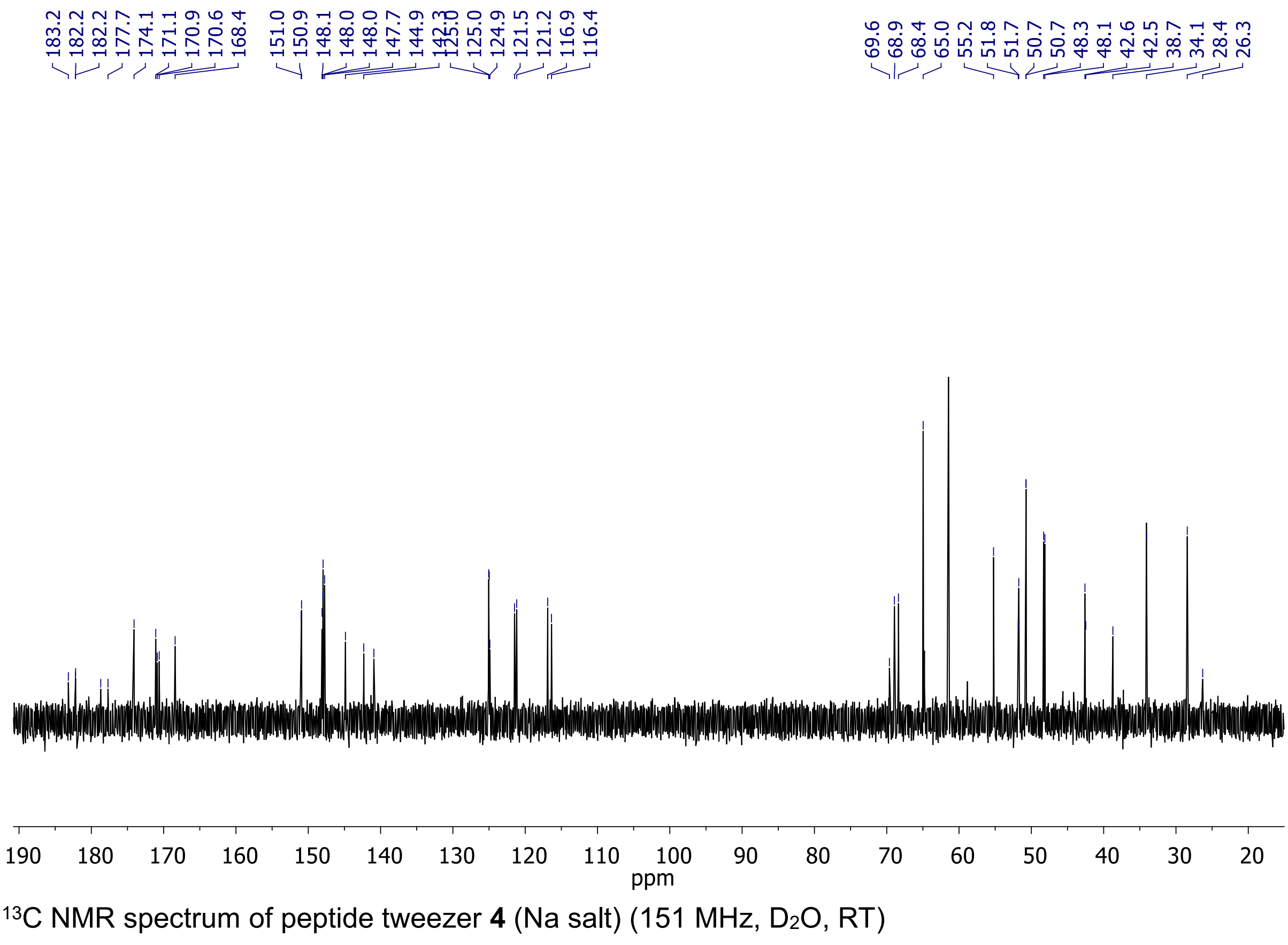

^{13}C NMR spectrum of peptide tweezer **4** (Na salt) (151 MHz, D_2O, RT)

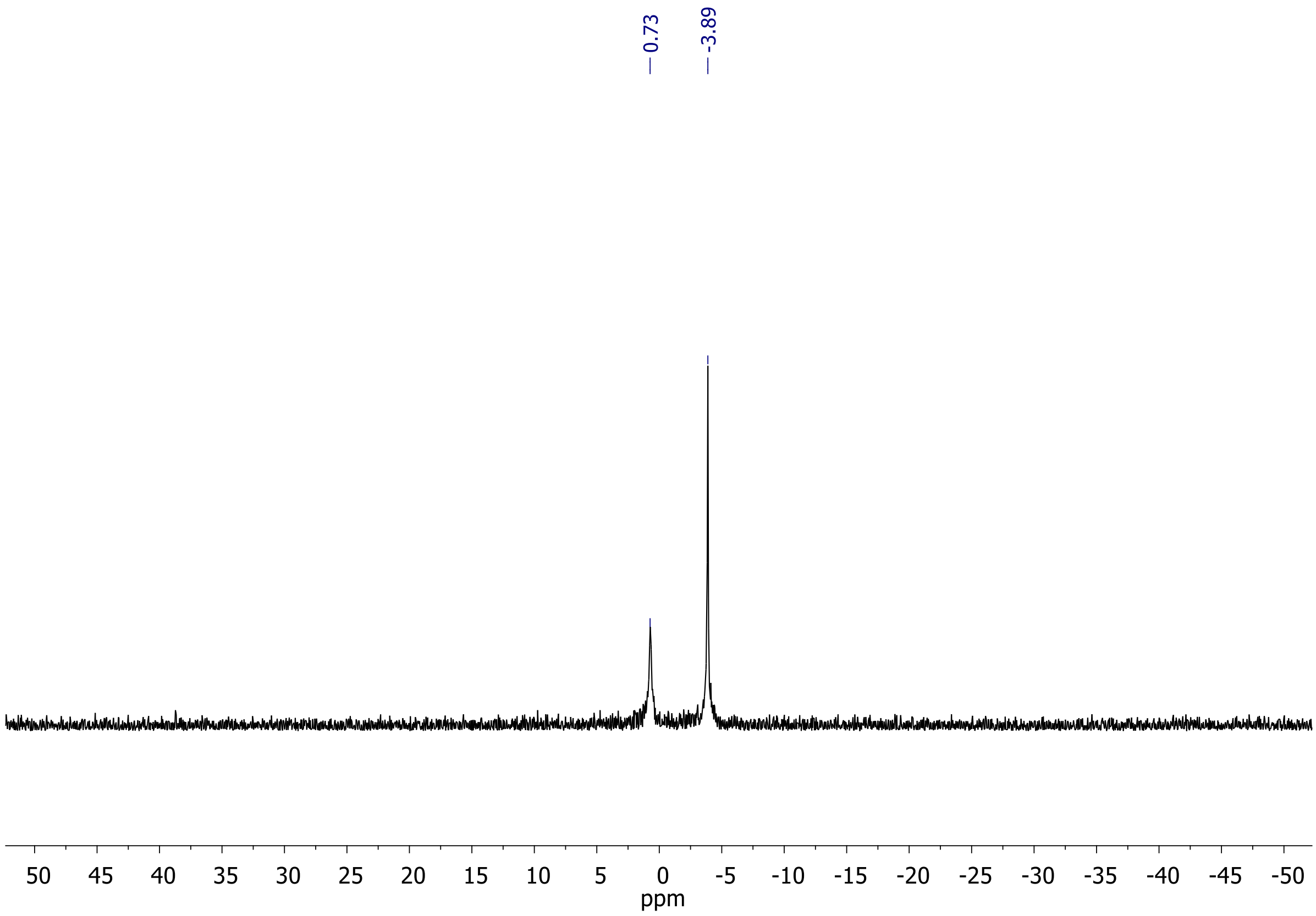

NMR spectrum of peptide tweezer **4** (Na salt) (162 MHz, D_2O, RT)

B Dimeric tweezers with functional linkers 5-11

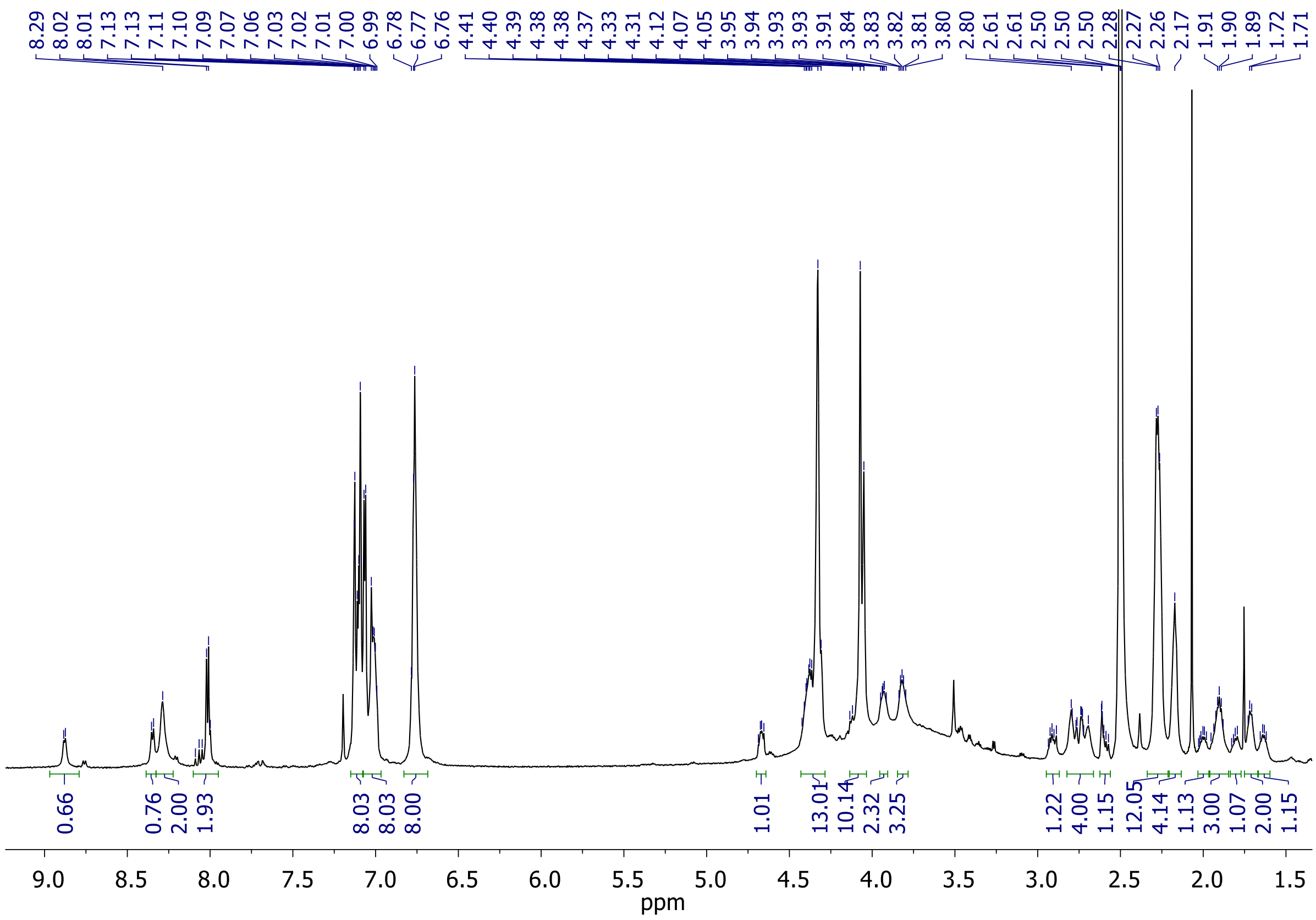

^{1}H NMR spectrum of anionic tweezer **5** (600 MHz, DMSO-d_6, RT)

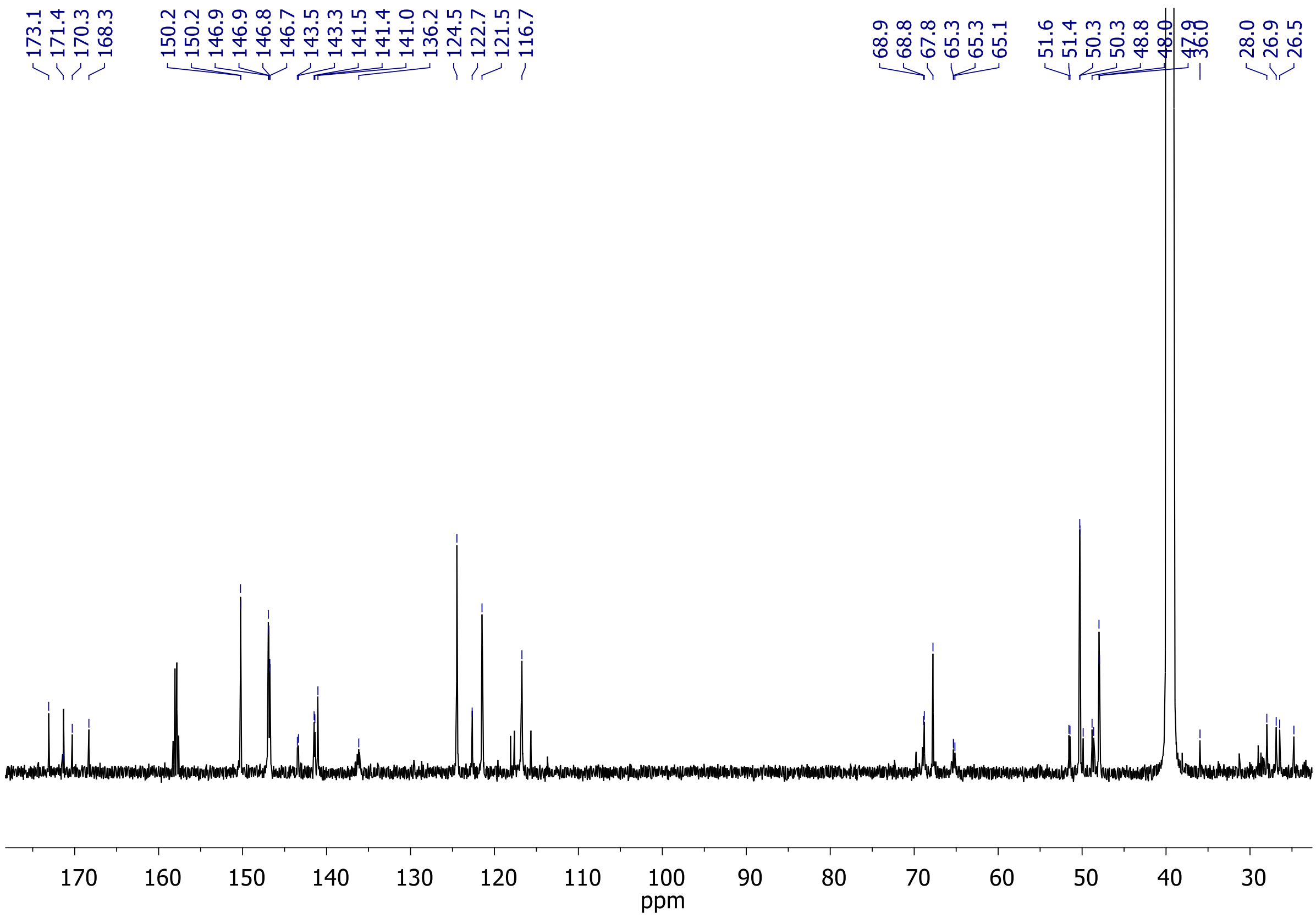

^{13}C NMR spectrum of anionic tweezer **5** (151 MHz, DMSO-d_6, RT)

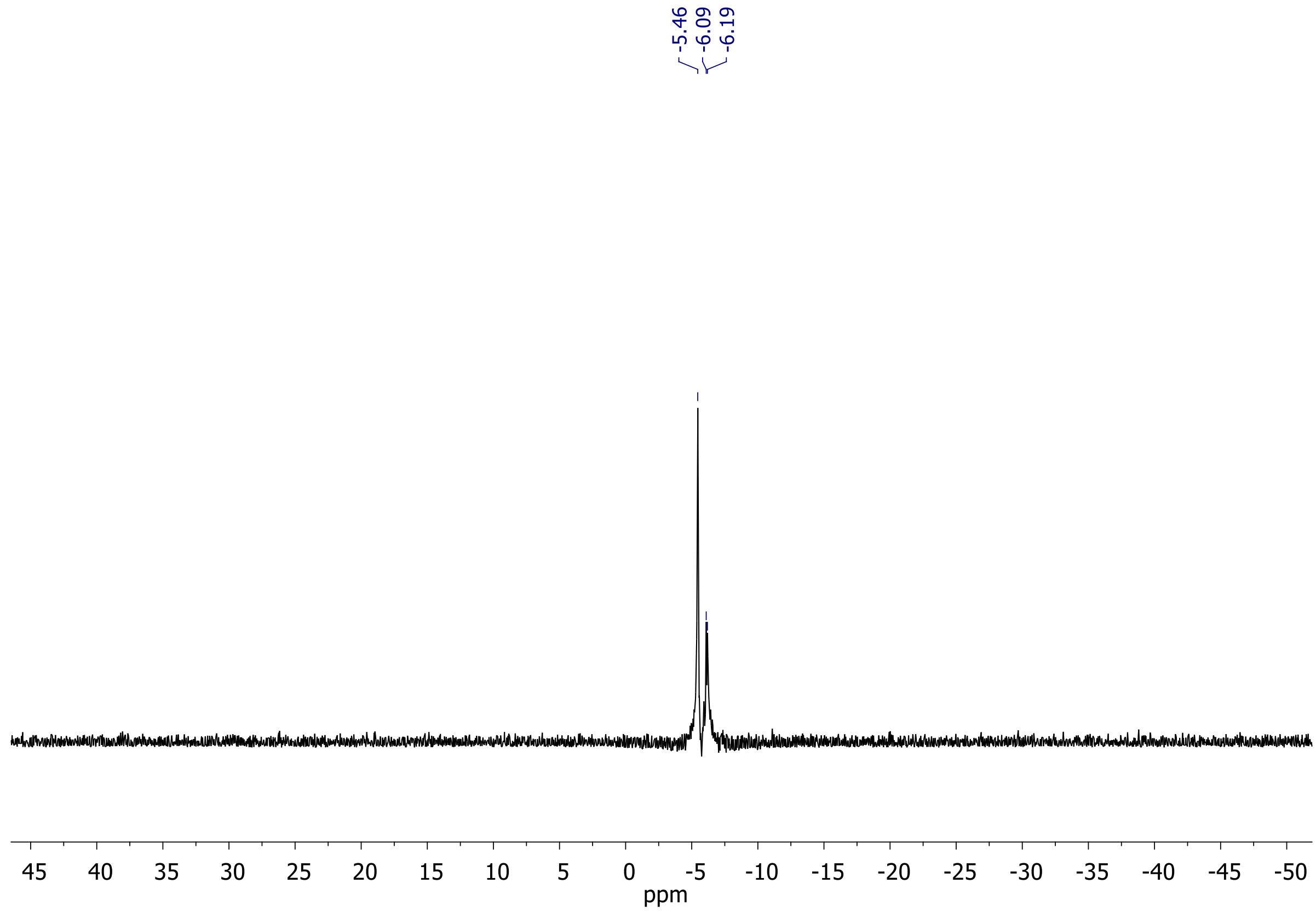

^{31}P NMR spectrum of anionic tweezer **5** (162 MHz, DMSO-d_6, RT)

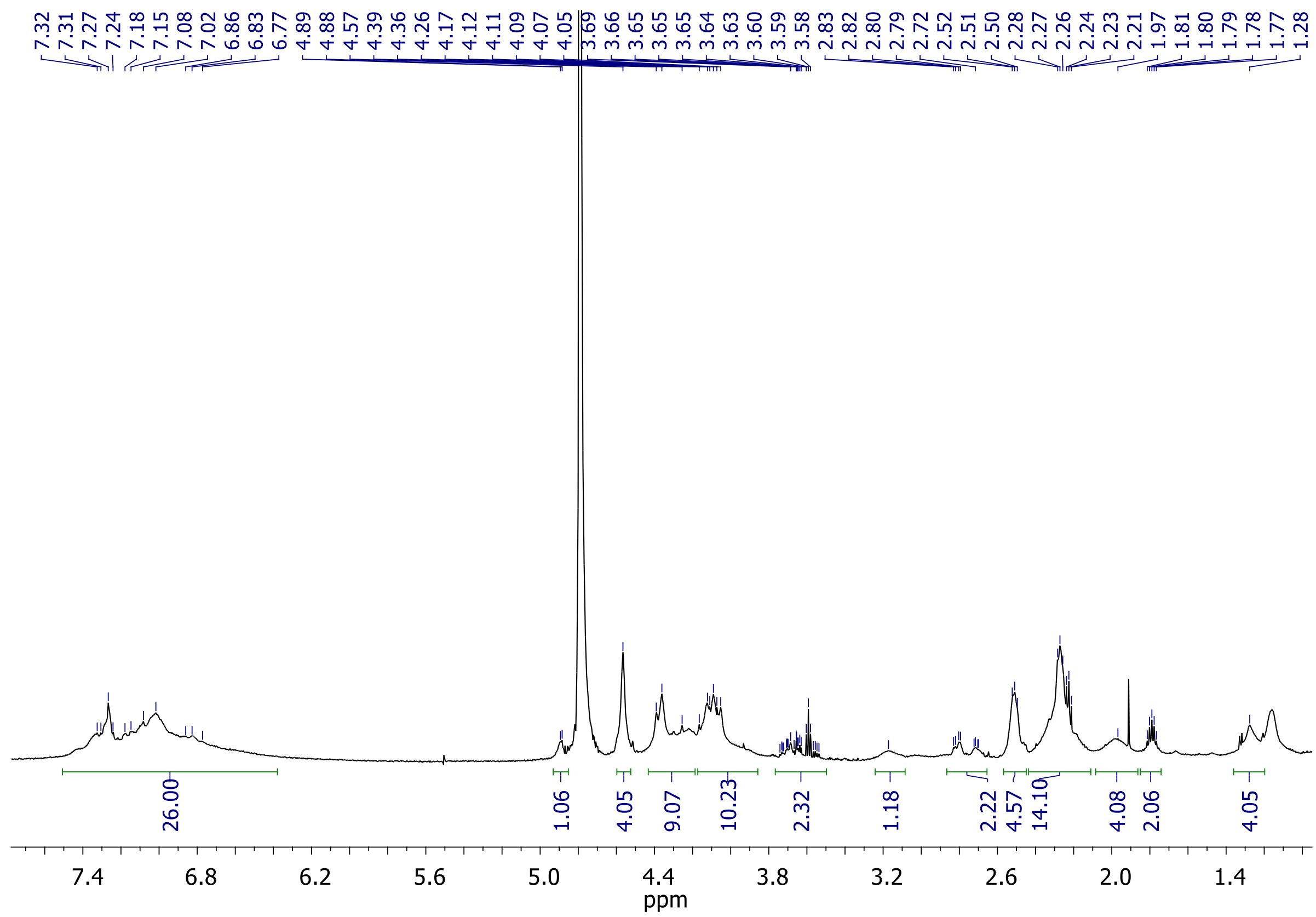

^{1}H NMR spectrum of anionic tweezer **5** (Na salt) (600 MHz, D_2O, RT)

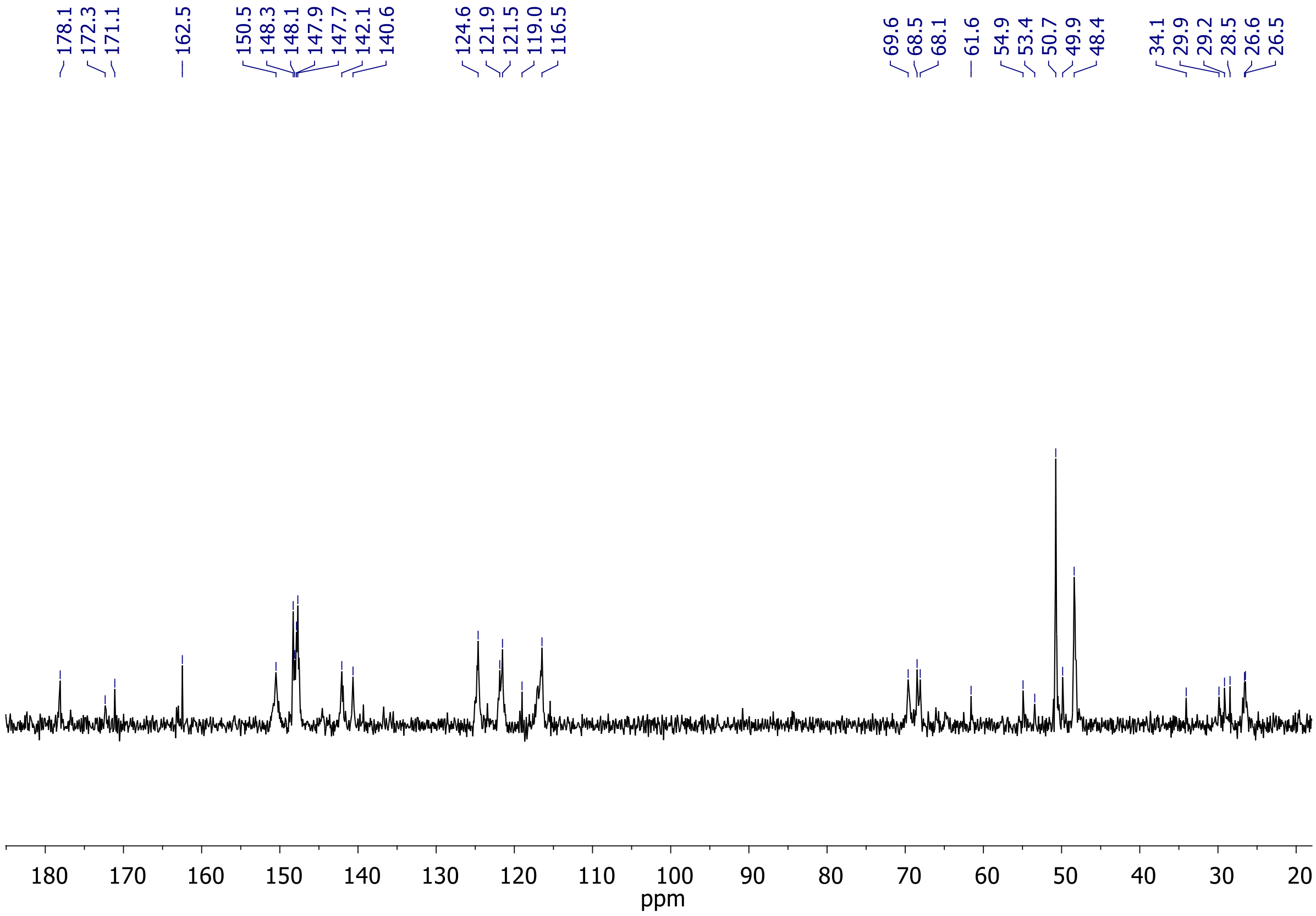

^{13}C NMR spectrum of anionic tweezer **5** (Na salt) (151 MHz, D_2O, RT)

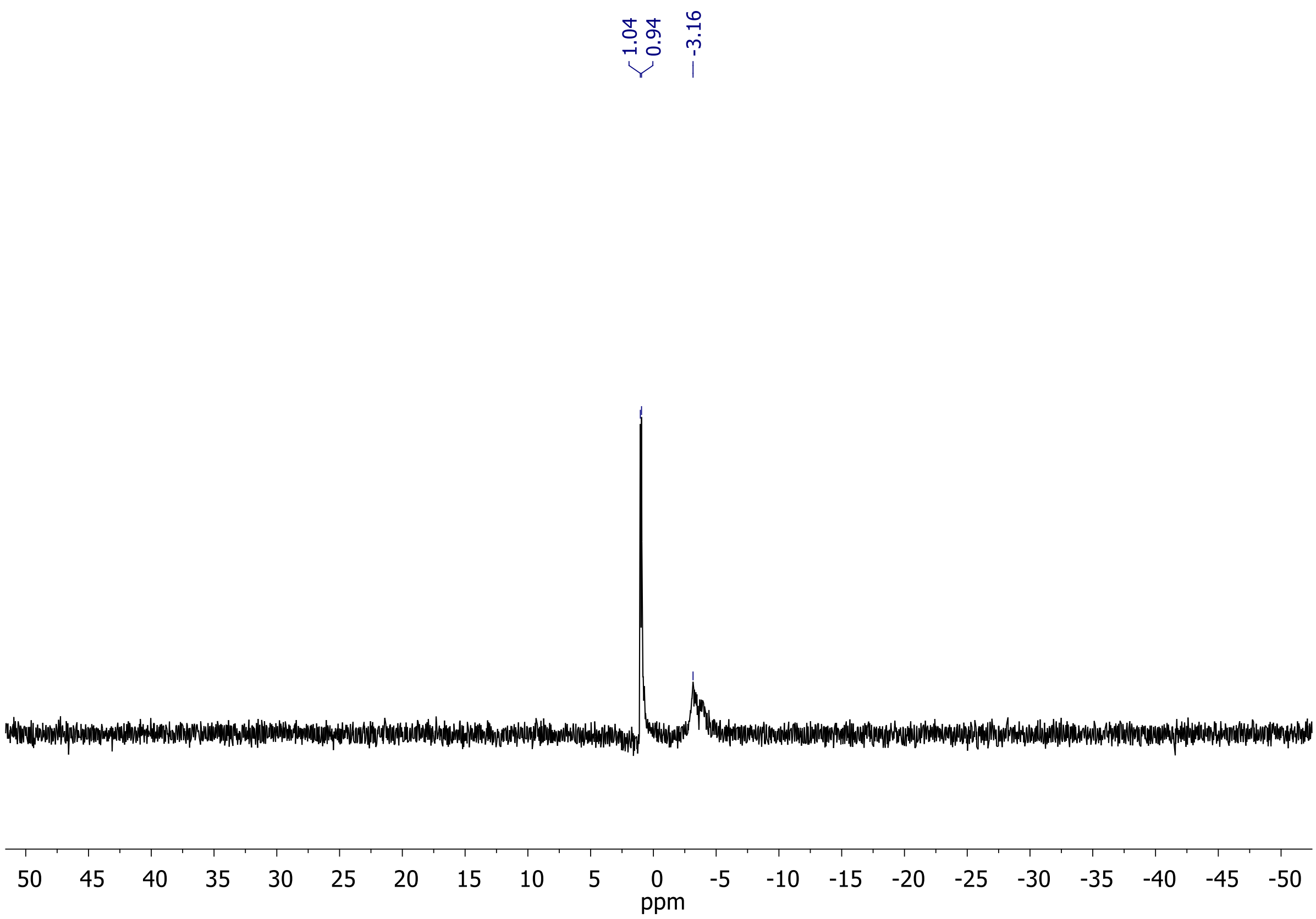

^{31}P NMR spectrum of anionic tweezer **5** (Na salt) (162 MHz, D_2O, RT)

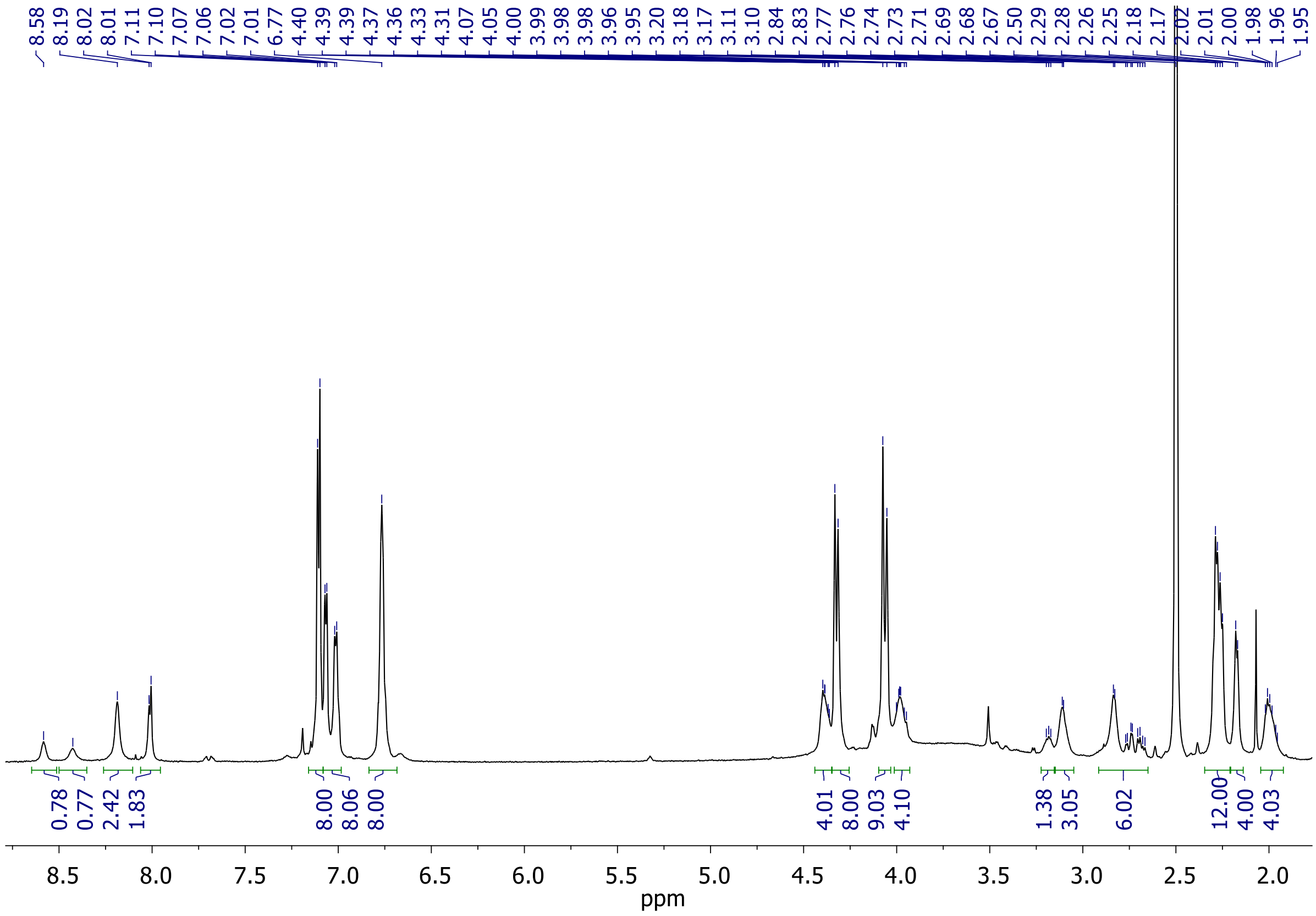

^{1}H NMR spectrum of cationic tweezer **6** (TFA salt) (600 MHz, DMSO-d_6, RT)

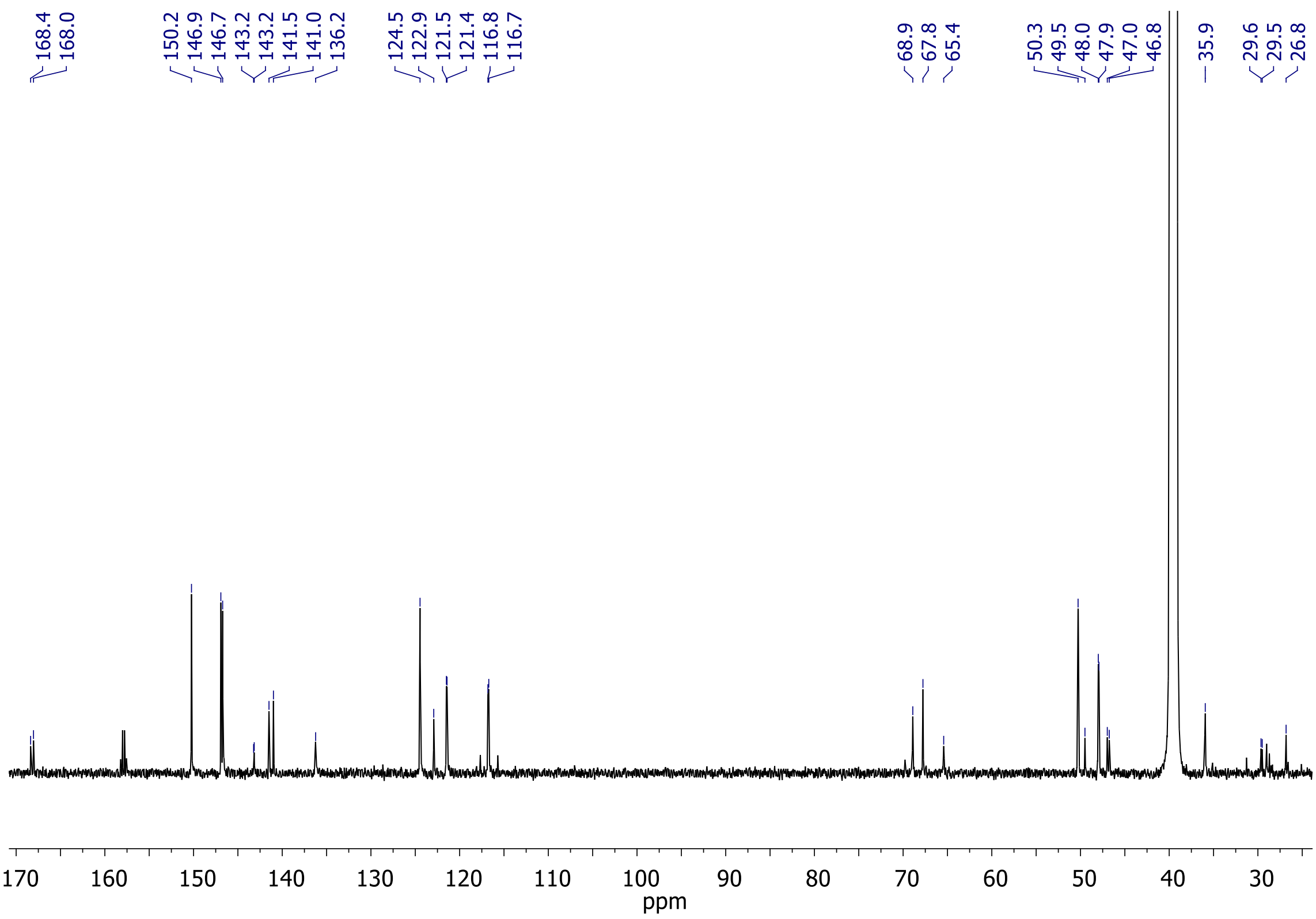

^{13}C NMR spectrum of cationic tweezer **6** (TFA salt) (151 MHz, DMSO-d_6, RT)

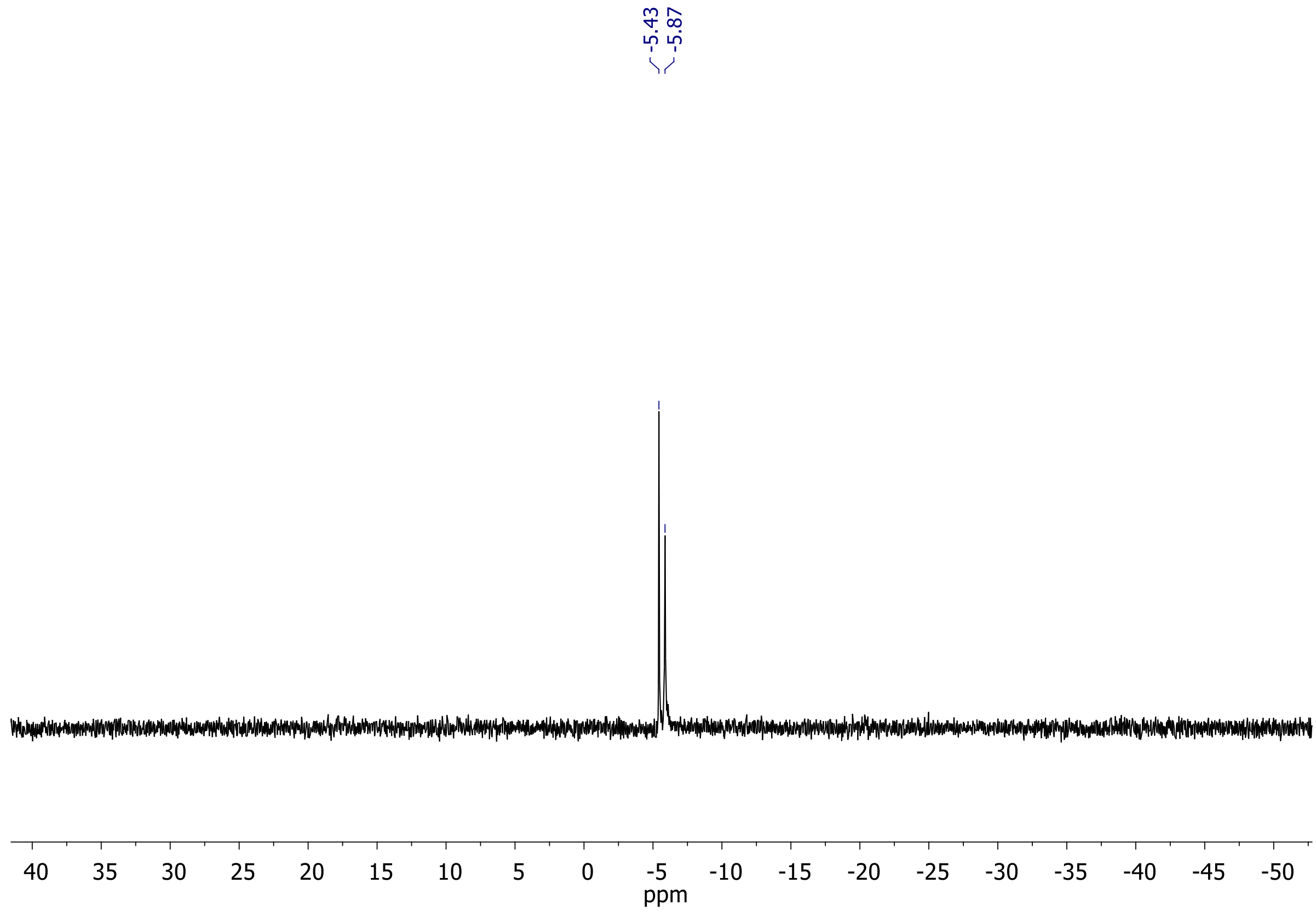

^{31}P NMR spectrum of cationic tweezer **6** (TFA salt) (162 MHz, DMSO-d_6, RT)

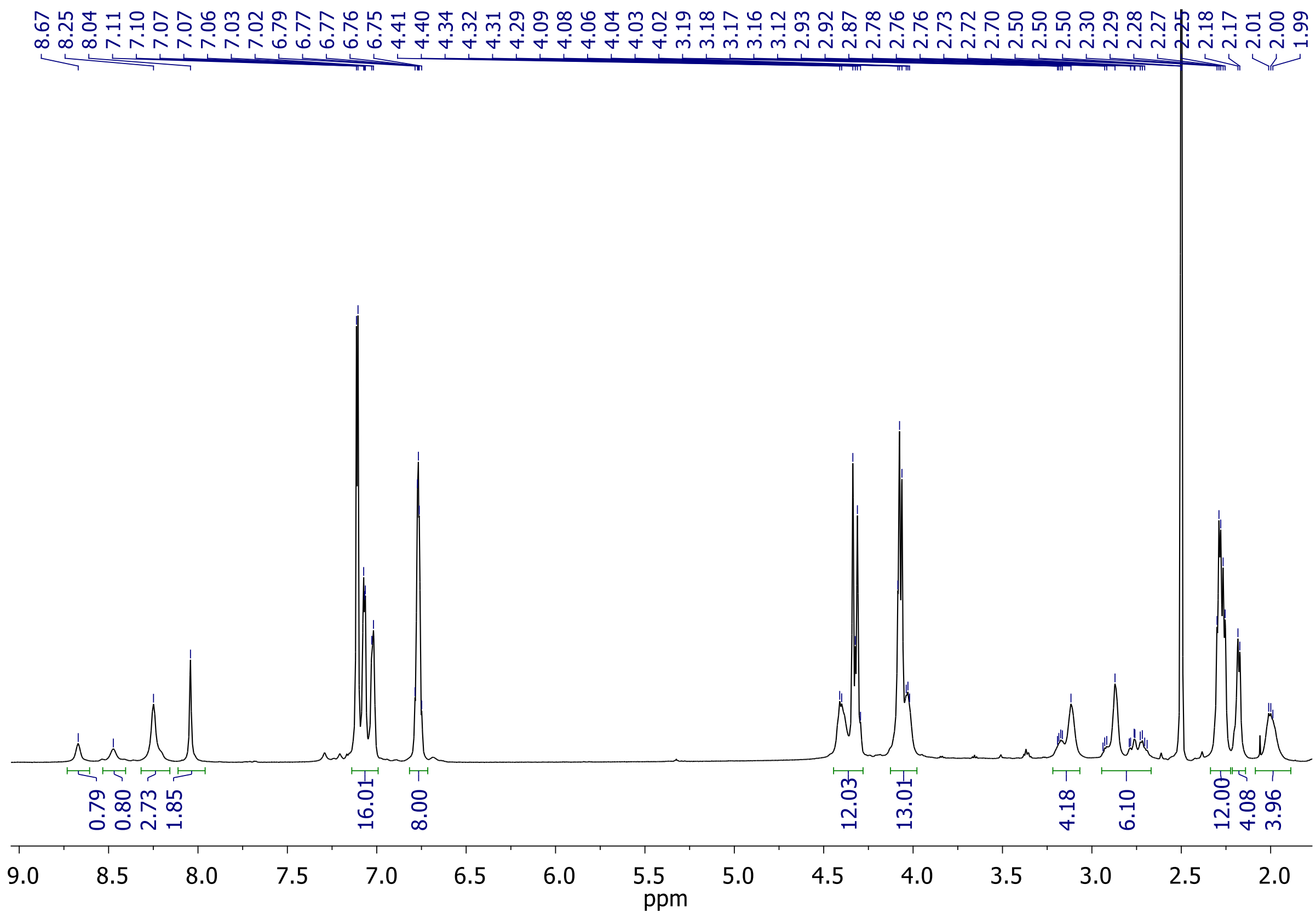

^{1}H NMR spectrum of cationic tweezer **6** (HCl salt) (600 MHz, DMSO-d_6, RT)

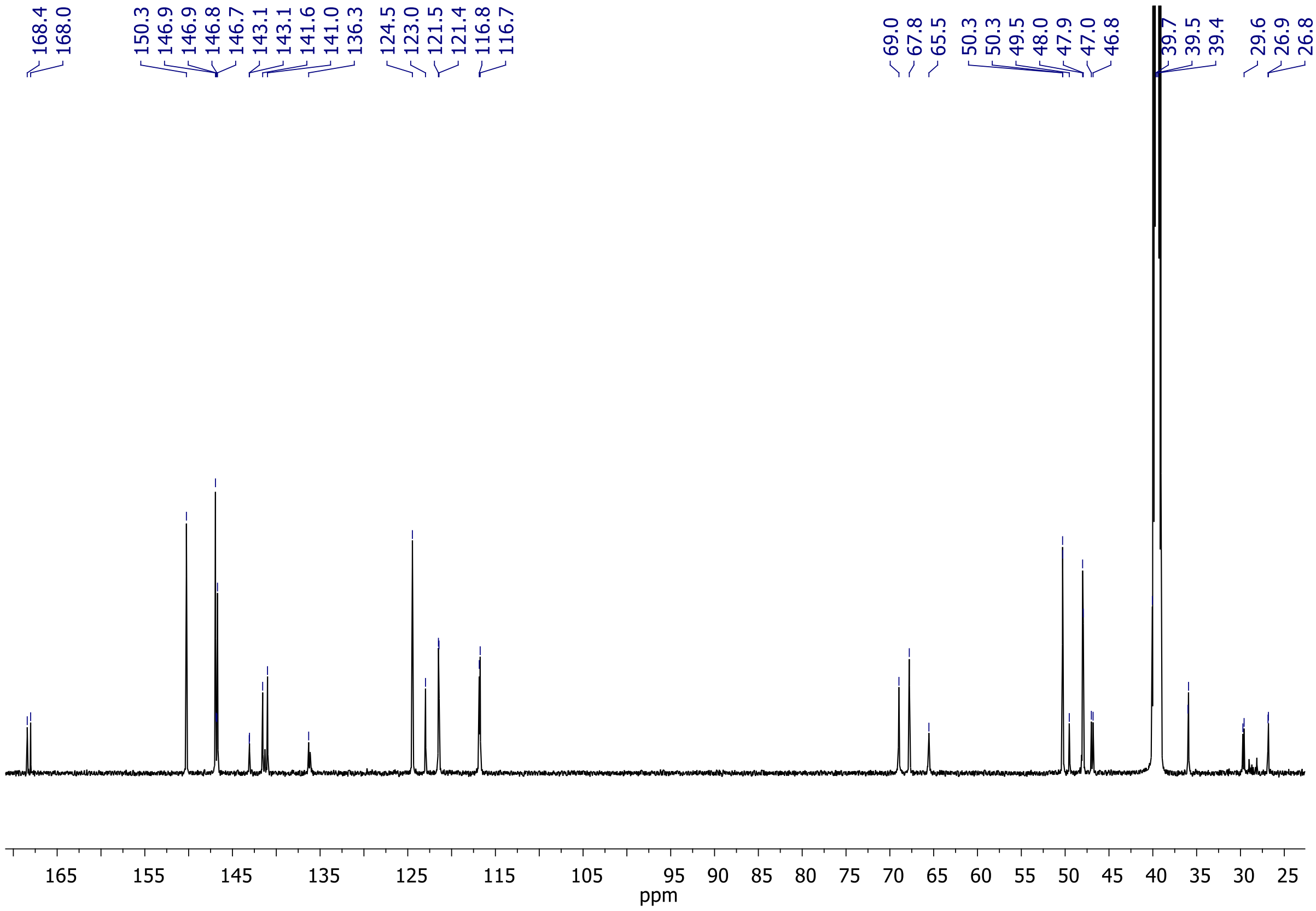

^{13}C NMR spectrum of cationic tweezer **6** (HCl salt) (150 MHz, DMSO-d_6, RT)

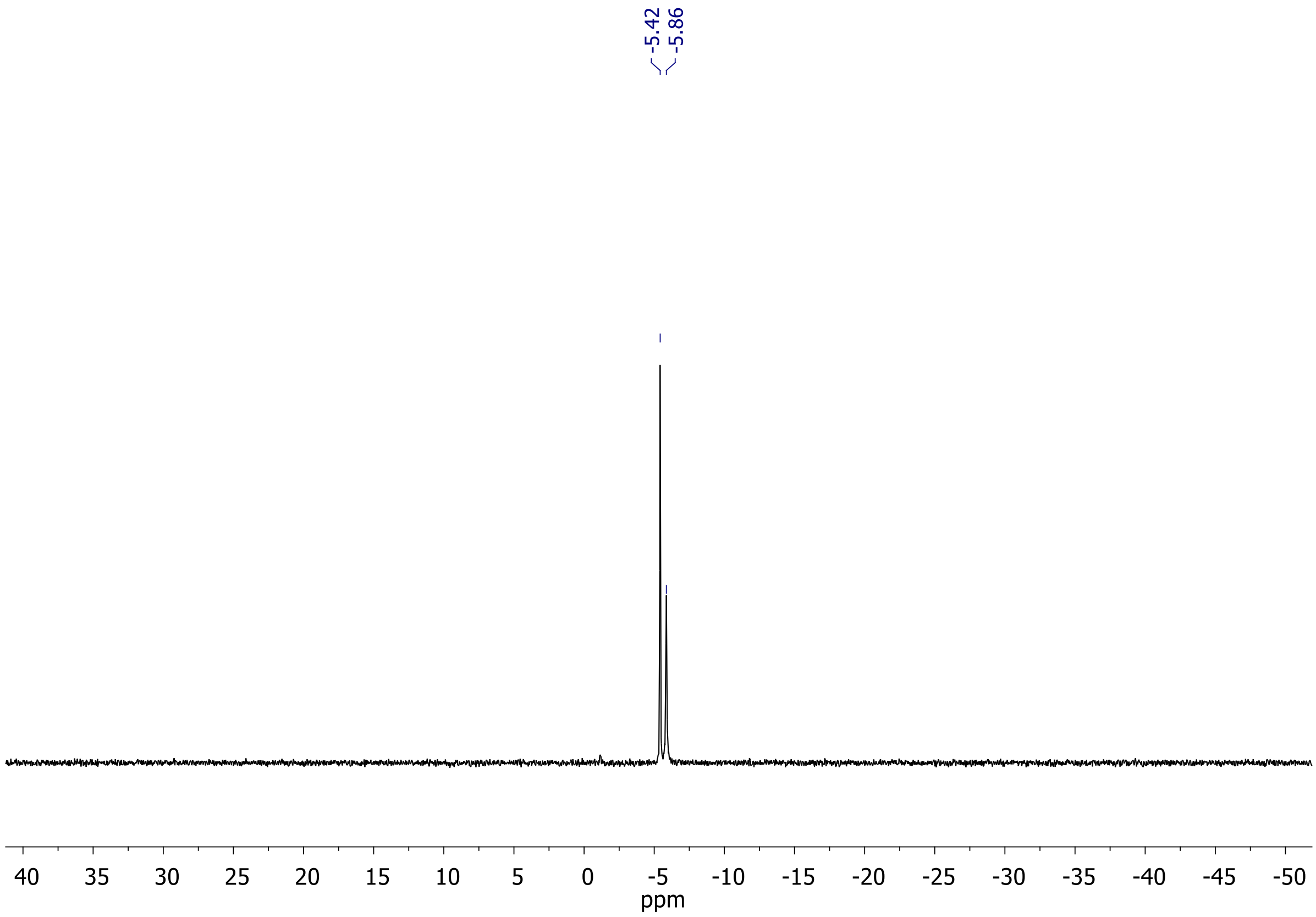

^{31}P NMR spectrum of cationic tweezer **6** (HCl salt) (162 MHz, DMSO-d_6, RT)

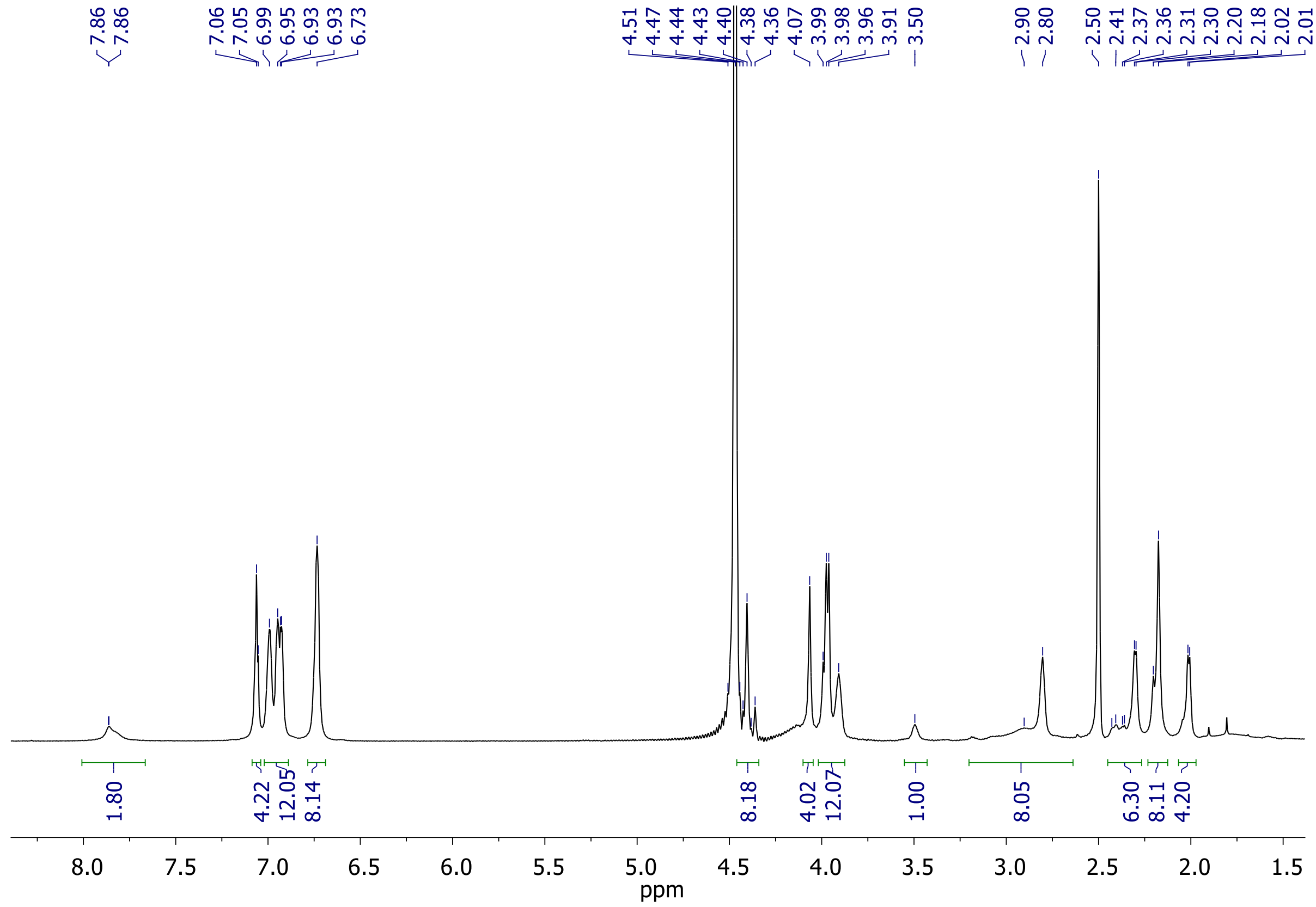

^{1}H NMR spectrum of cationic tweezer **6** (Na salt) (600 MHz, D_2O+DMSO-d_6, RT)

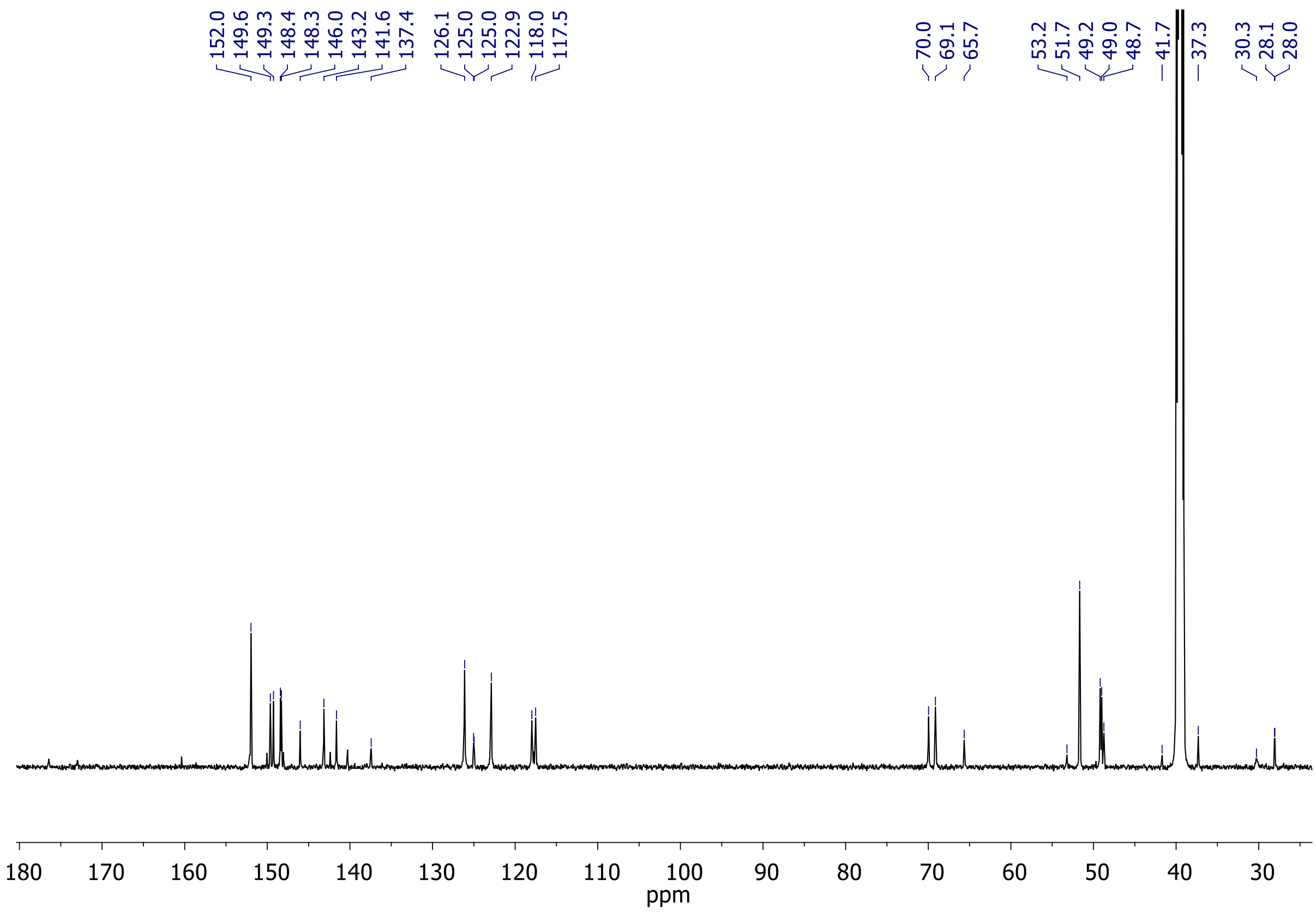

^{13}C NMR spectrum of cationic tweezer **6** (Na salt) (151 MHz, D_2O+DMSO-d_6, RT)

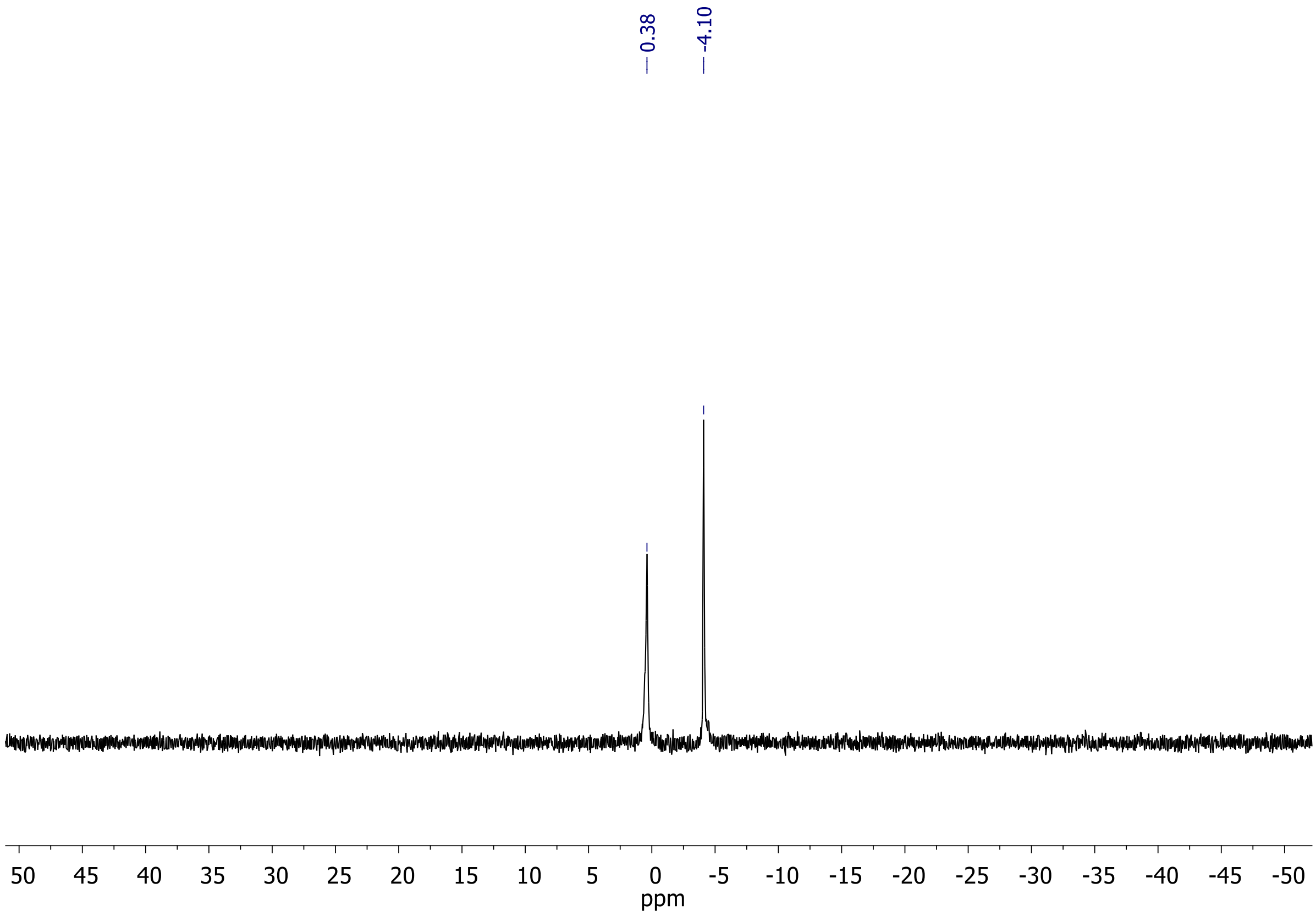

^{31}P NMR spectrum of cationic tweezer **6** (Na salt) (243 MHz, D_2O+DMSO-d_6, RT)

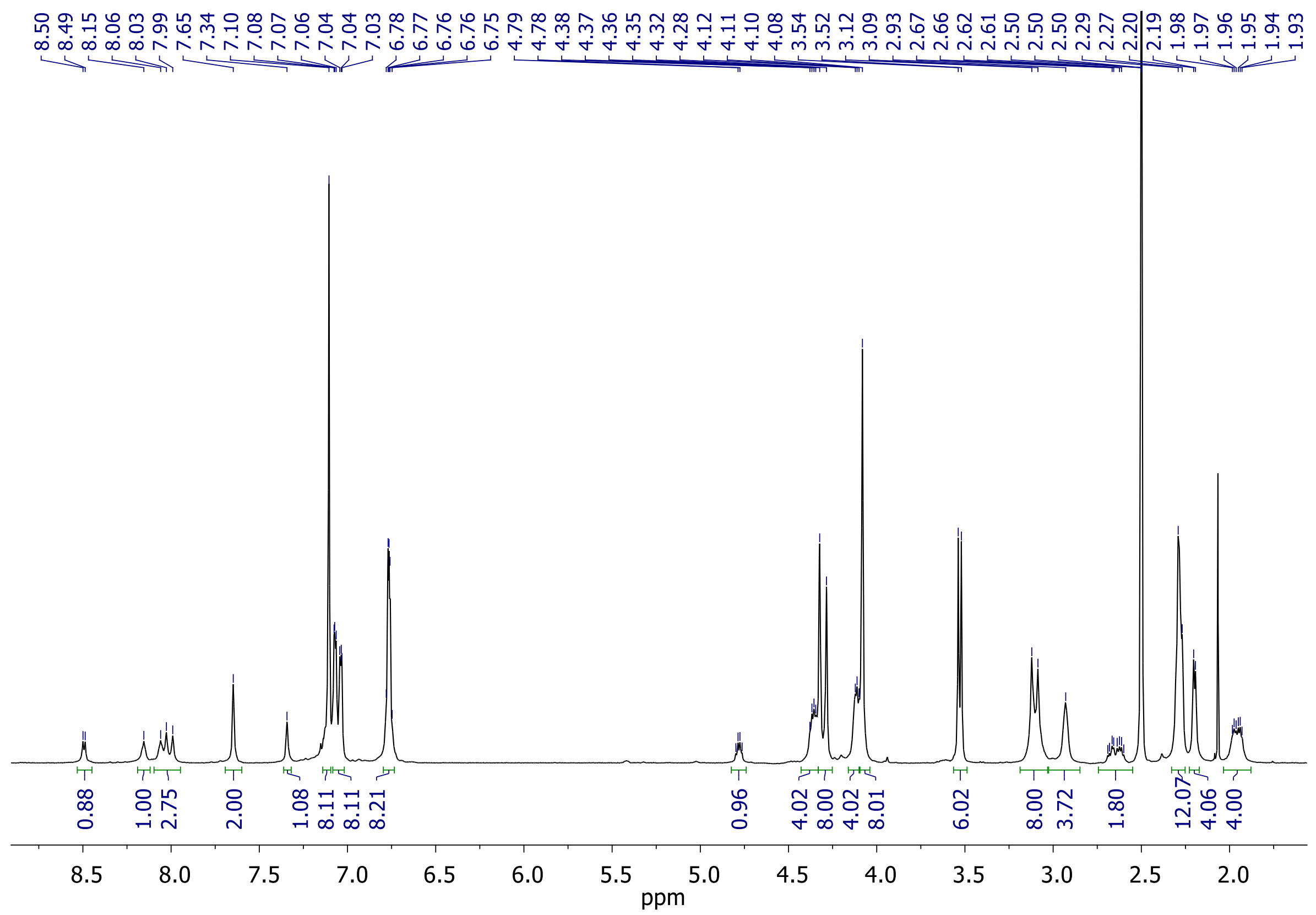

^{1}H NMR spectrum of bisphosphonate tweezer **7** (600 MHz, DMSO-d_6, RT)

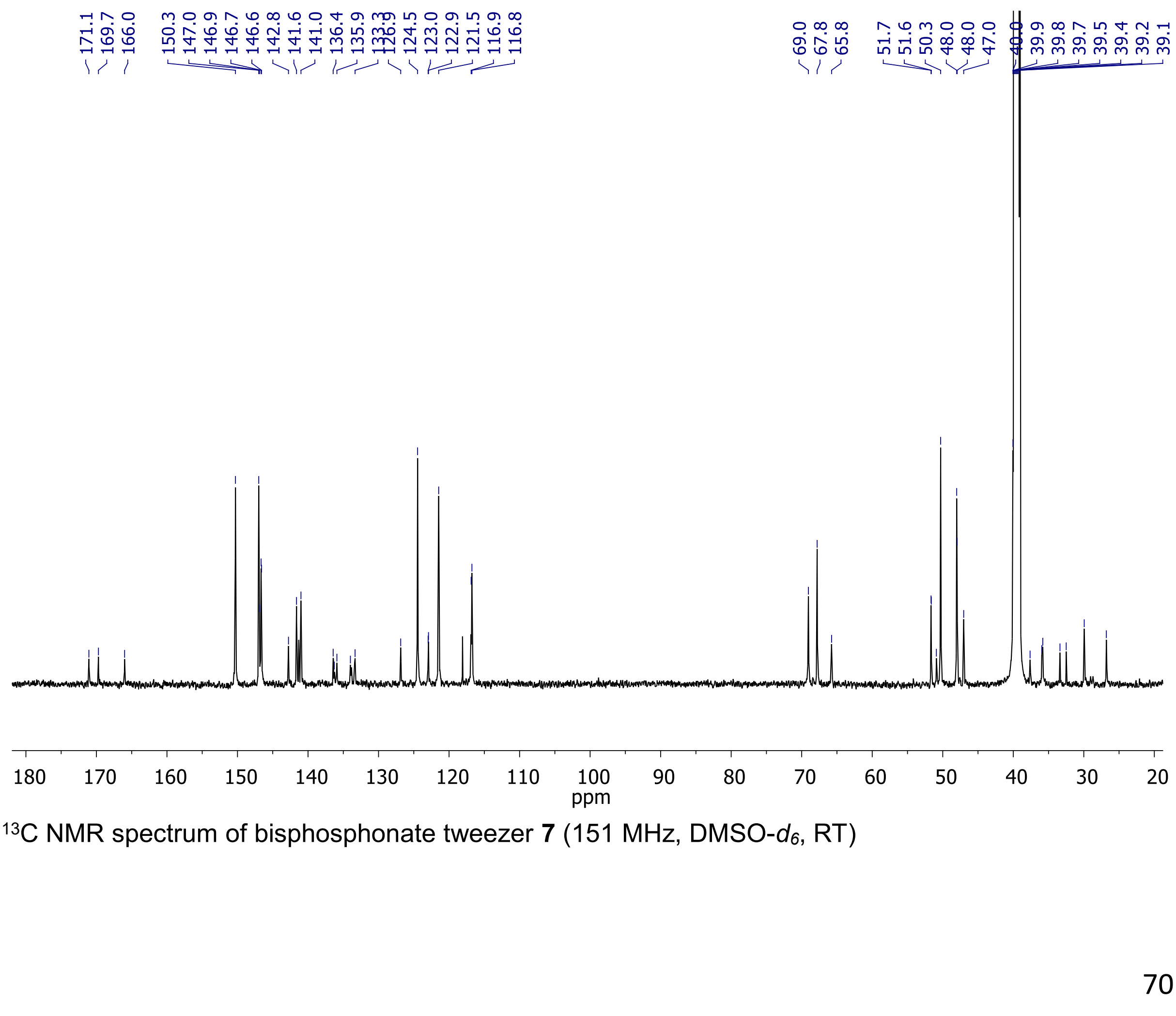

^{13}C NMR spectrum of bisphosphonate tweezer **7** (151 MHz, DMSO-d_6, RT)

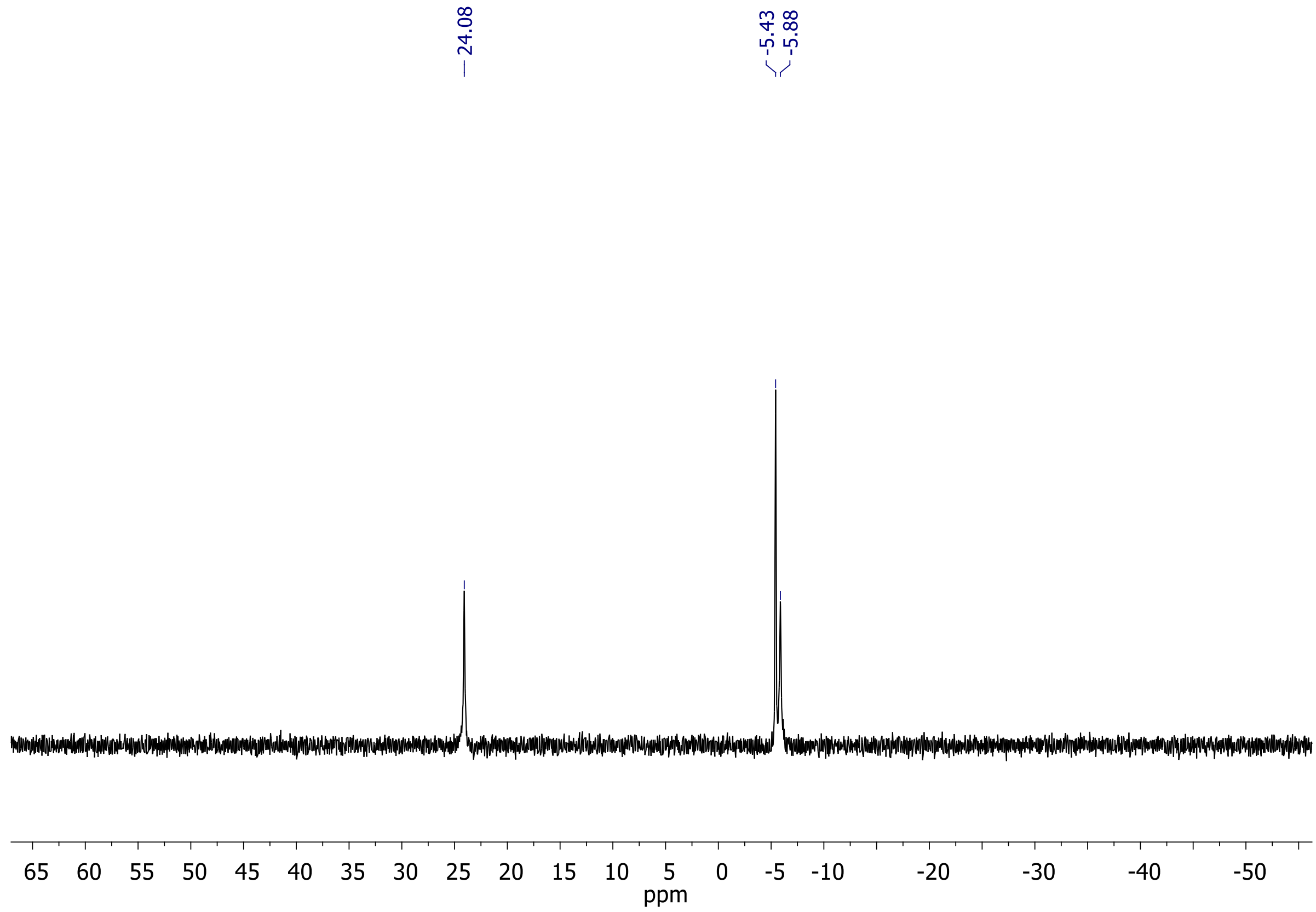

^{31}P NMR spectrum of bisphosphonate tweezer **7** (162 MHz, DMSO-d_6, RT)

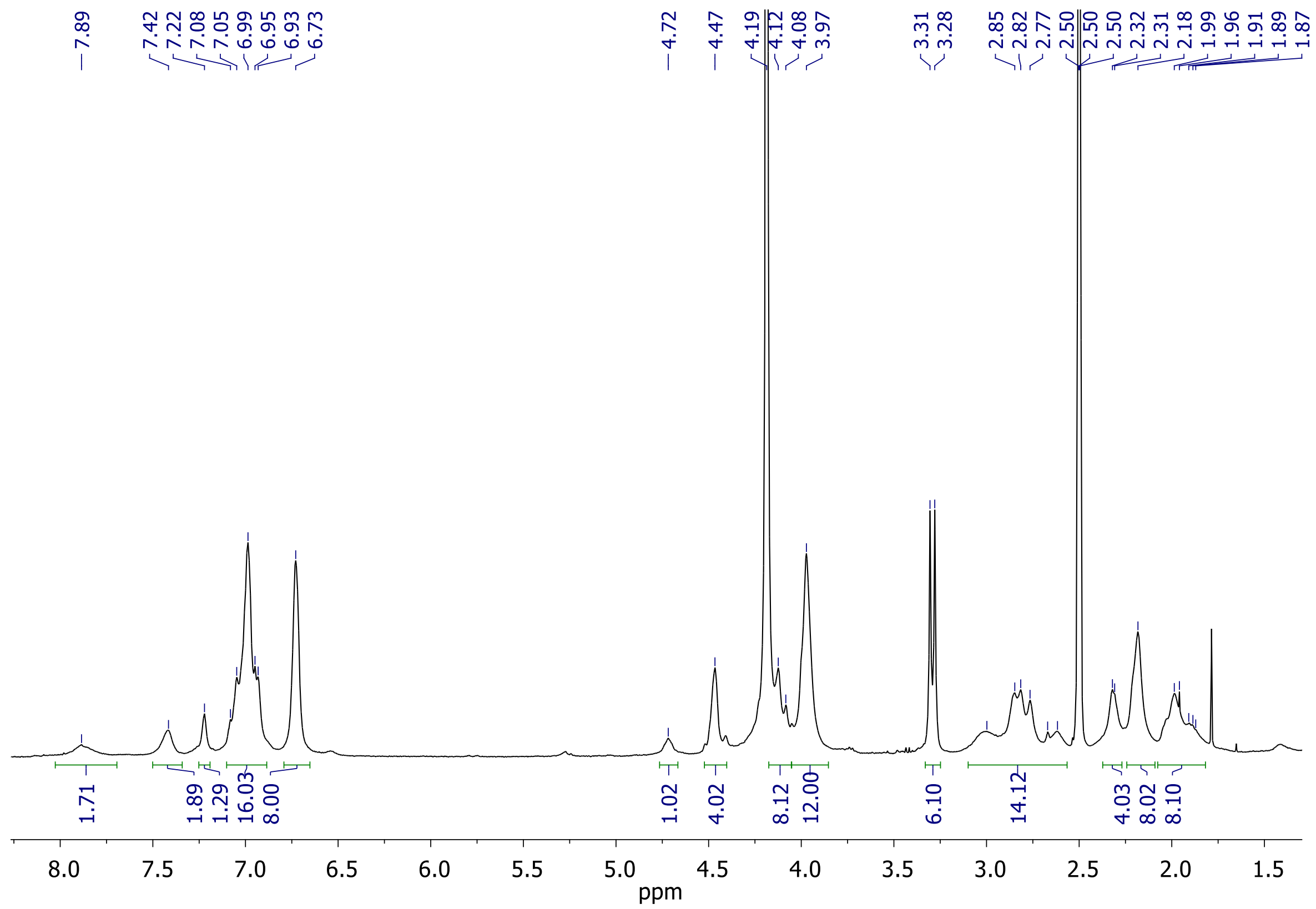

^{1}H NMR spectrum of bisphosphonate tweezer **7** (Na salt) (300 MHz, D_2O+DMSO-d_6, RT)

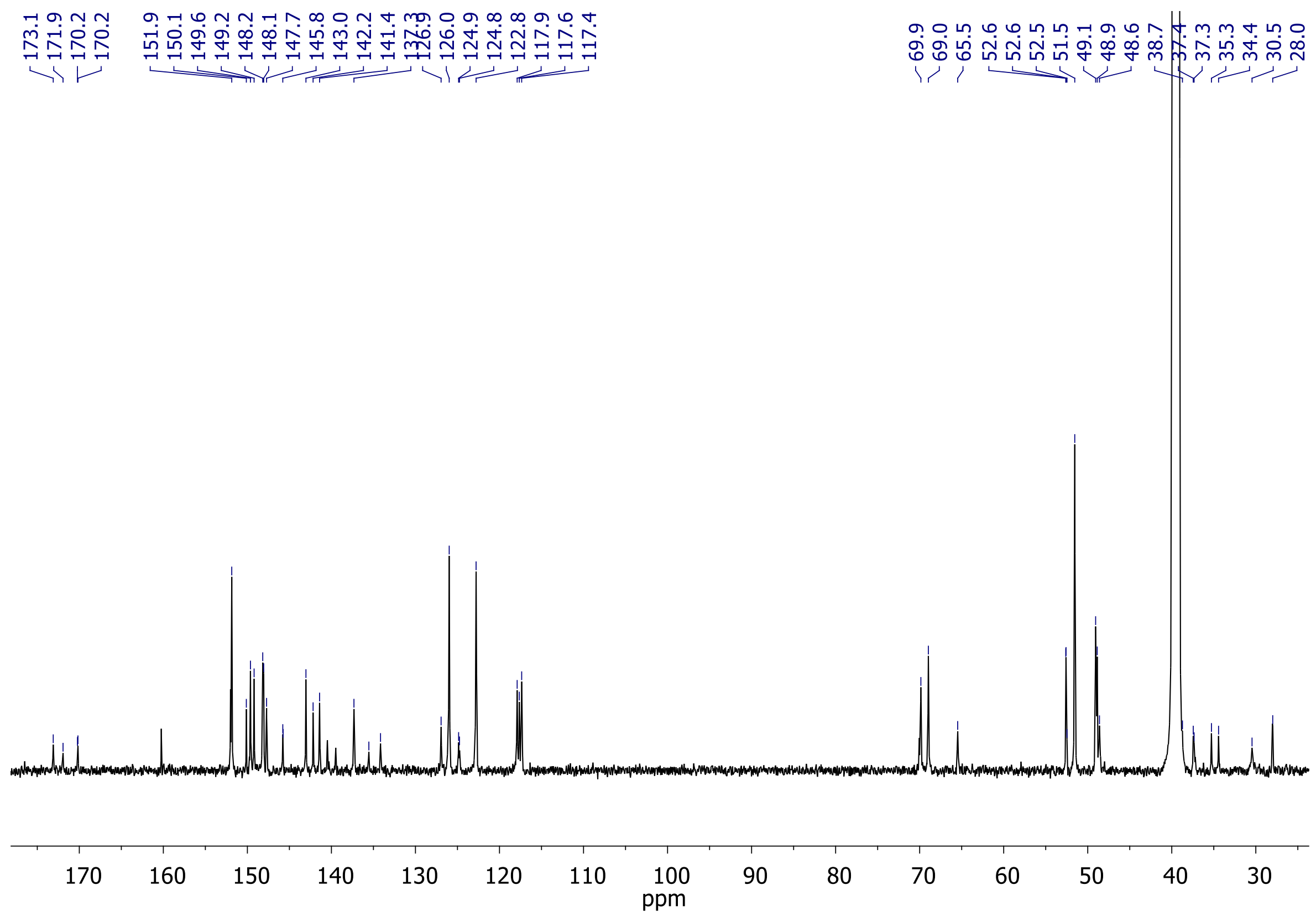

^{13}C NMR spectrum of bisphosphonate tweezer **7** (Na salt) (151 MHz, D_2O+DMSO-d_6, RT)

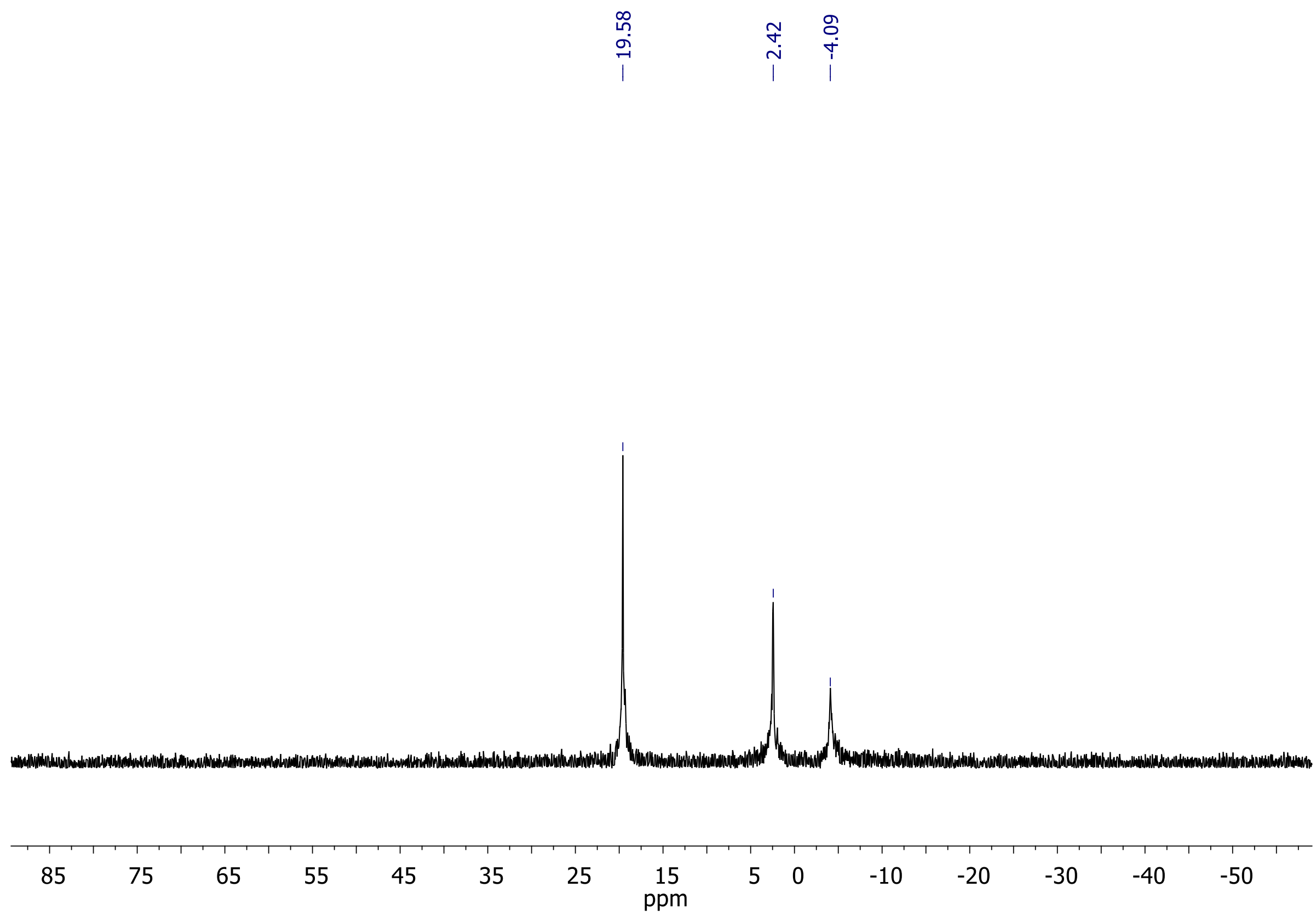

^{31}P NMR spectrum of bisphosphonate tweezer **7** (Na salt) (162 MHz, D_2O+DMSO-d_6, RT)

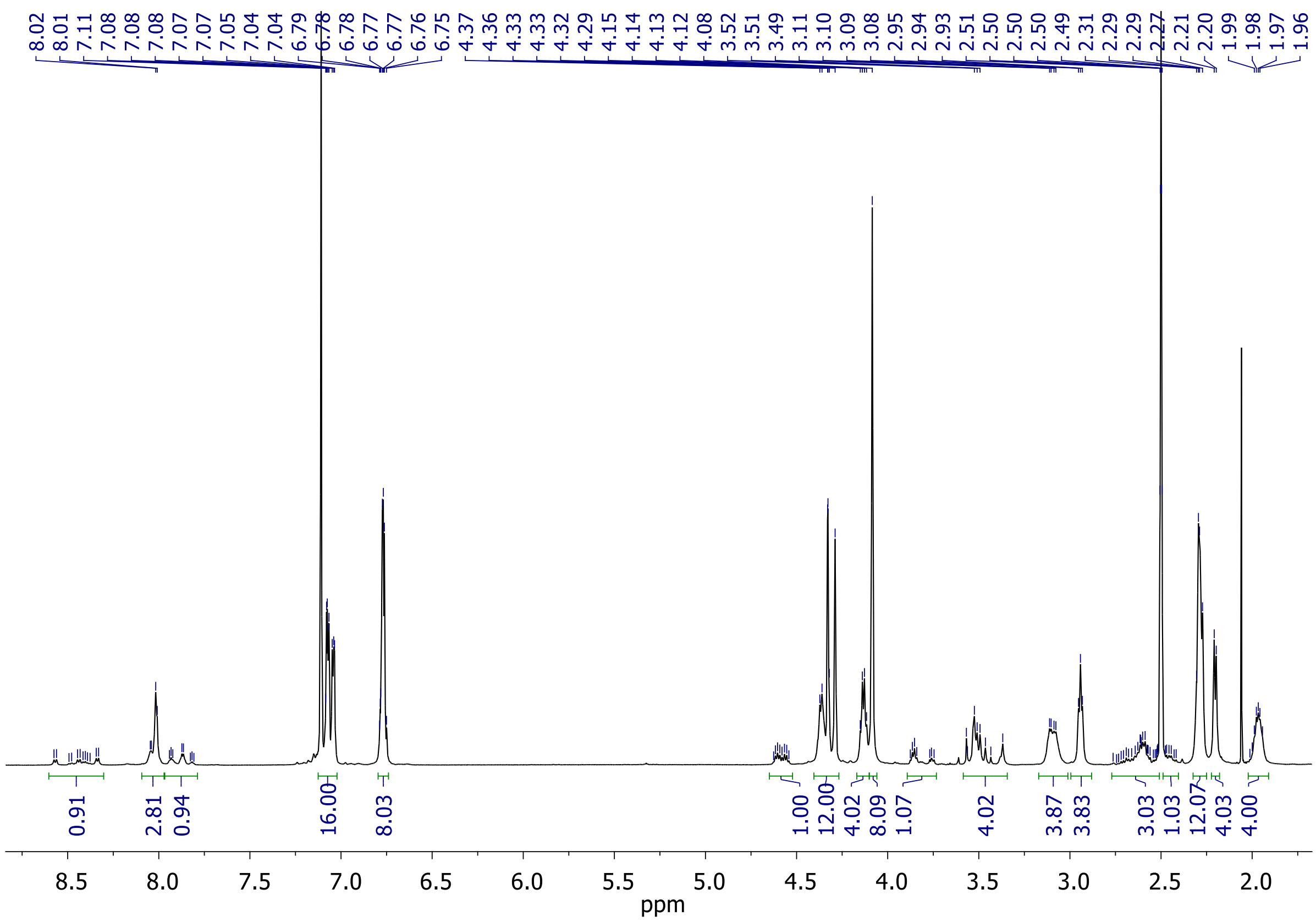

^{1}H NMR spectrum of NTA tweezer **8** (600 MHz, DMSO-d_6, RT)

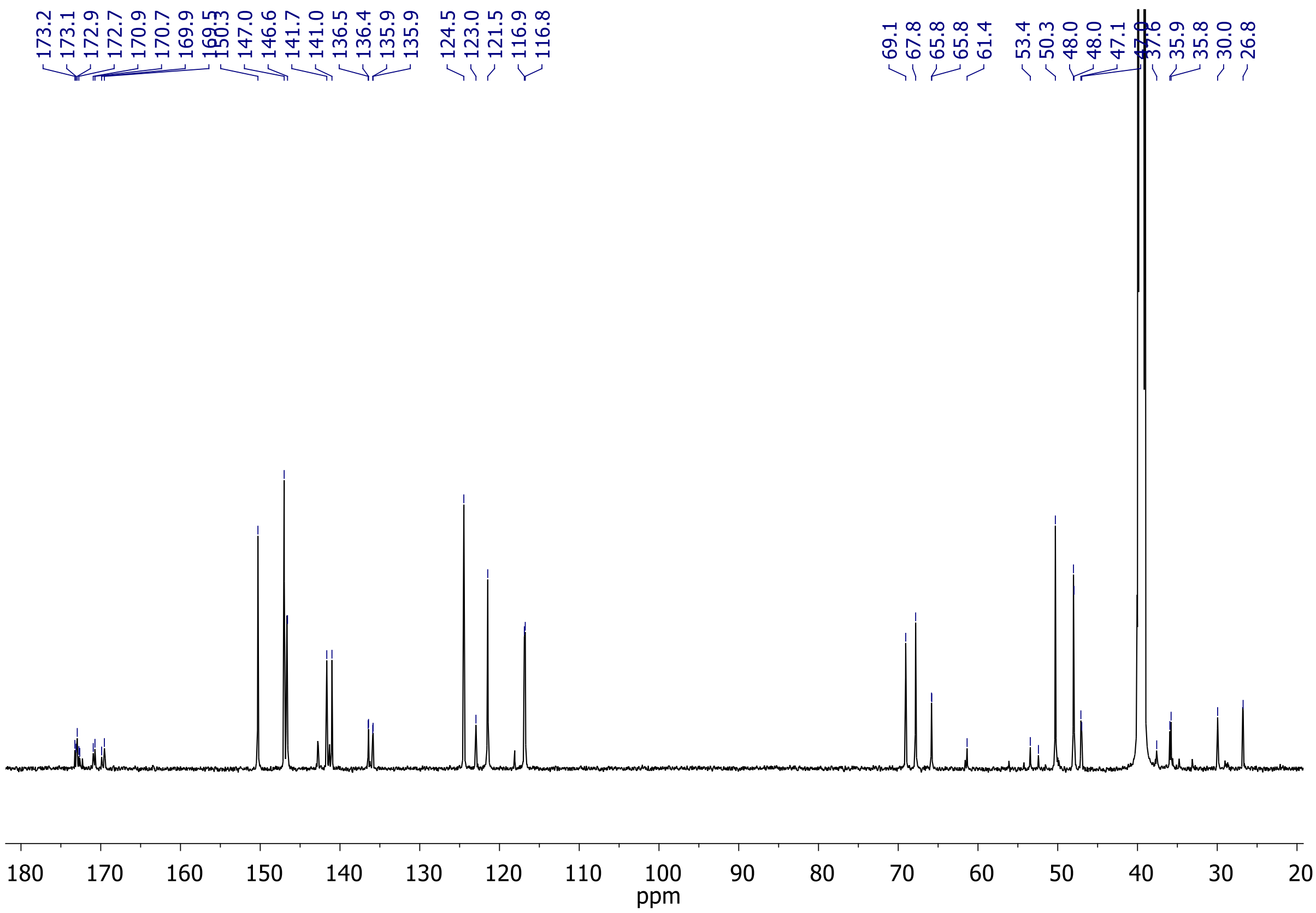

^{13}C NMR spectrum of NTA tweezer **8** (151 MHz, DMSO-d_6, RT)

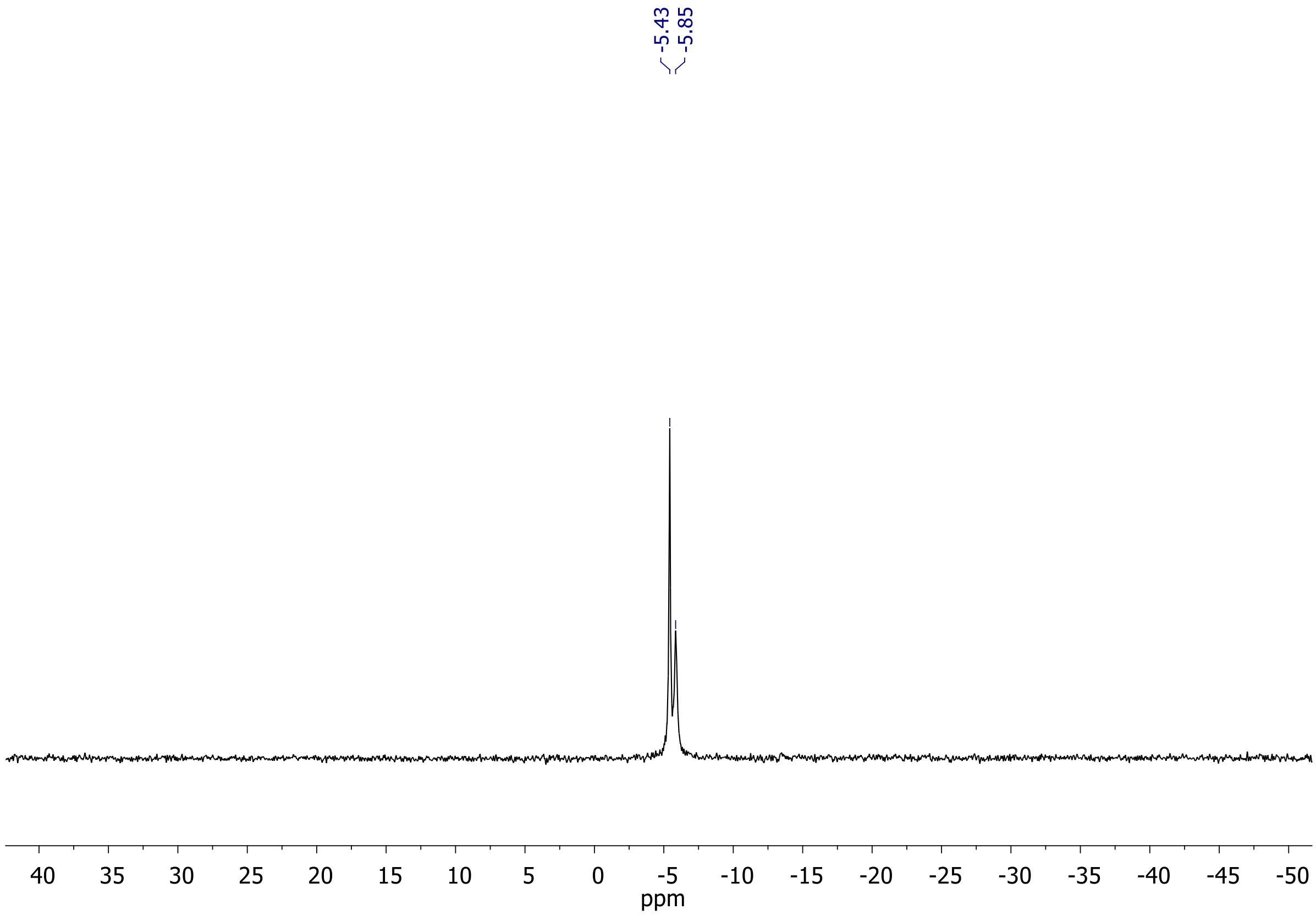

^{31}P NMR spectrum of NTA tweezer **8** (243 MHz, DMSO-*d*$_6$, RT)

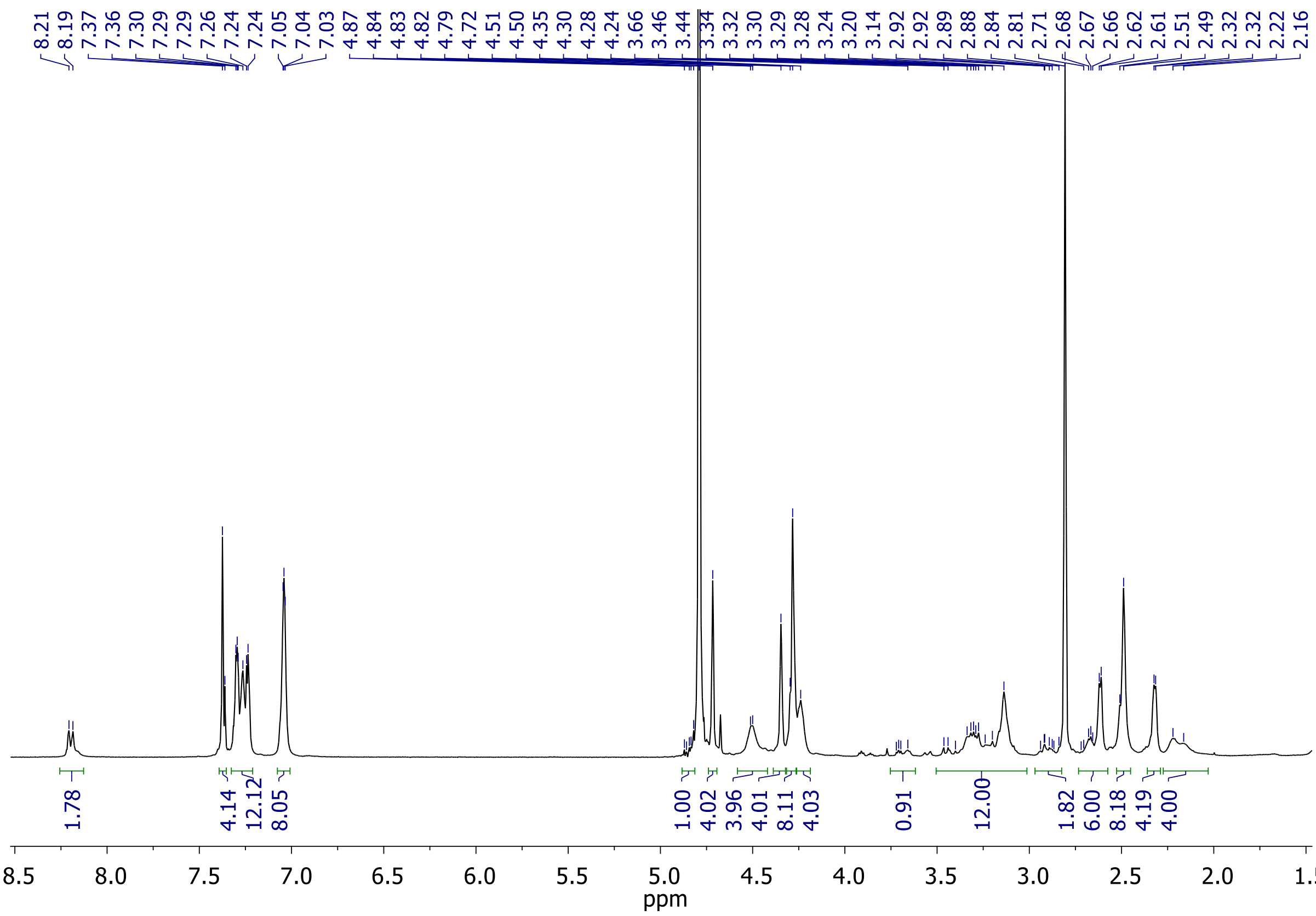

^{1}H NMR spectrum of NTA tweezer **8** (Na salt) (600 MHz, D_2O+DMSO-*d*$_6$, RT)

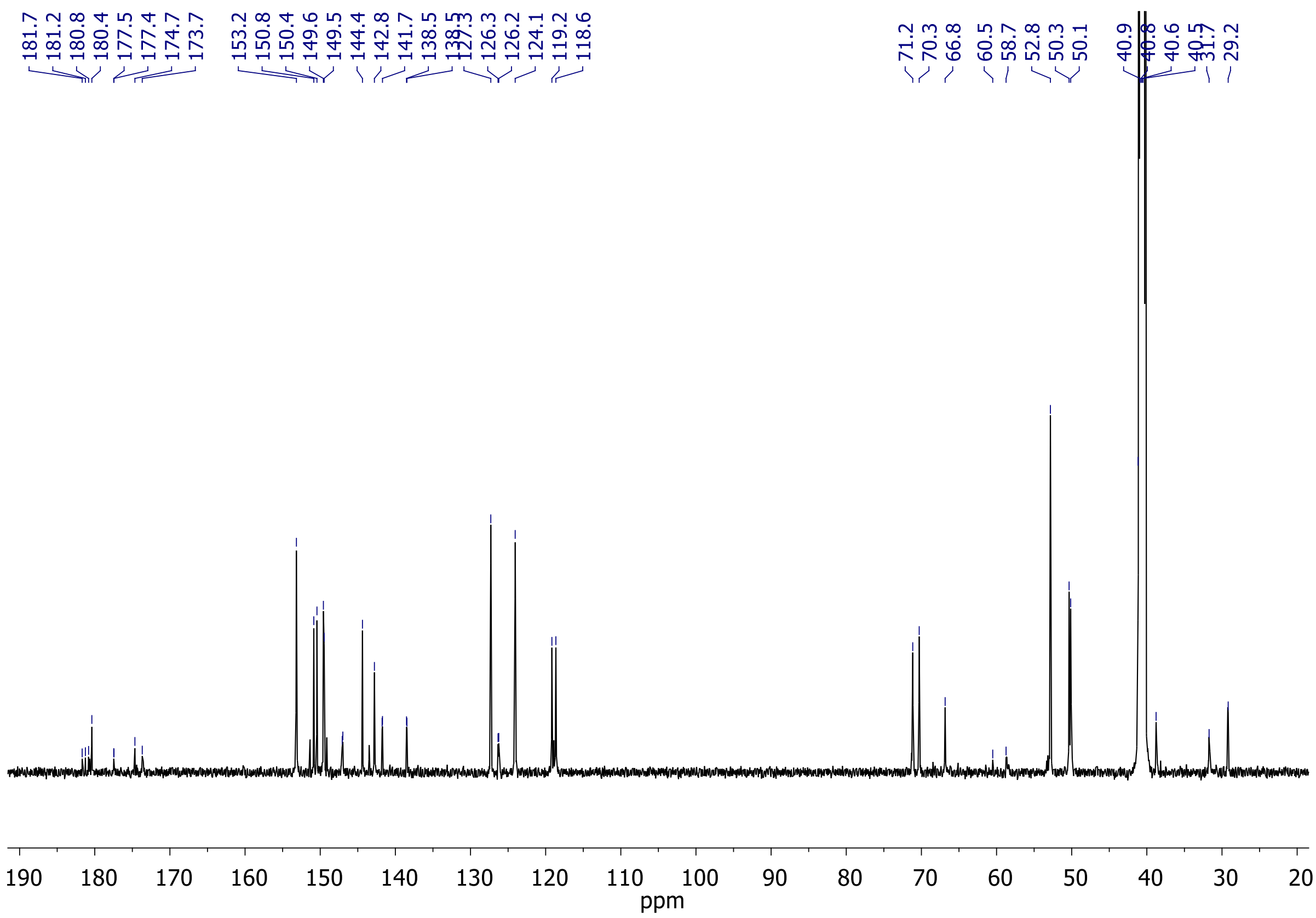

^{13}C NMR spectrum of NTA tweezer **8** (Na salt) (151 MHz, D_2O+DMSO-d_6, RT)

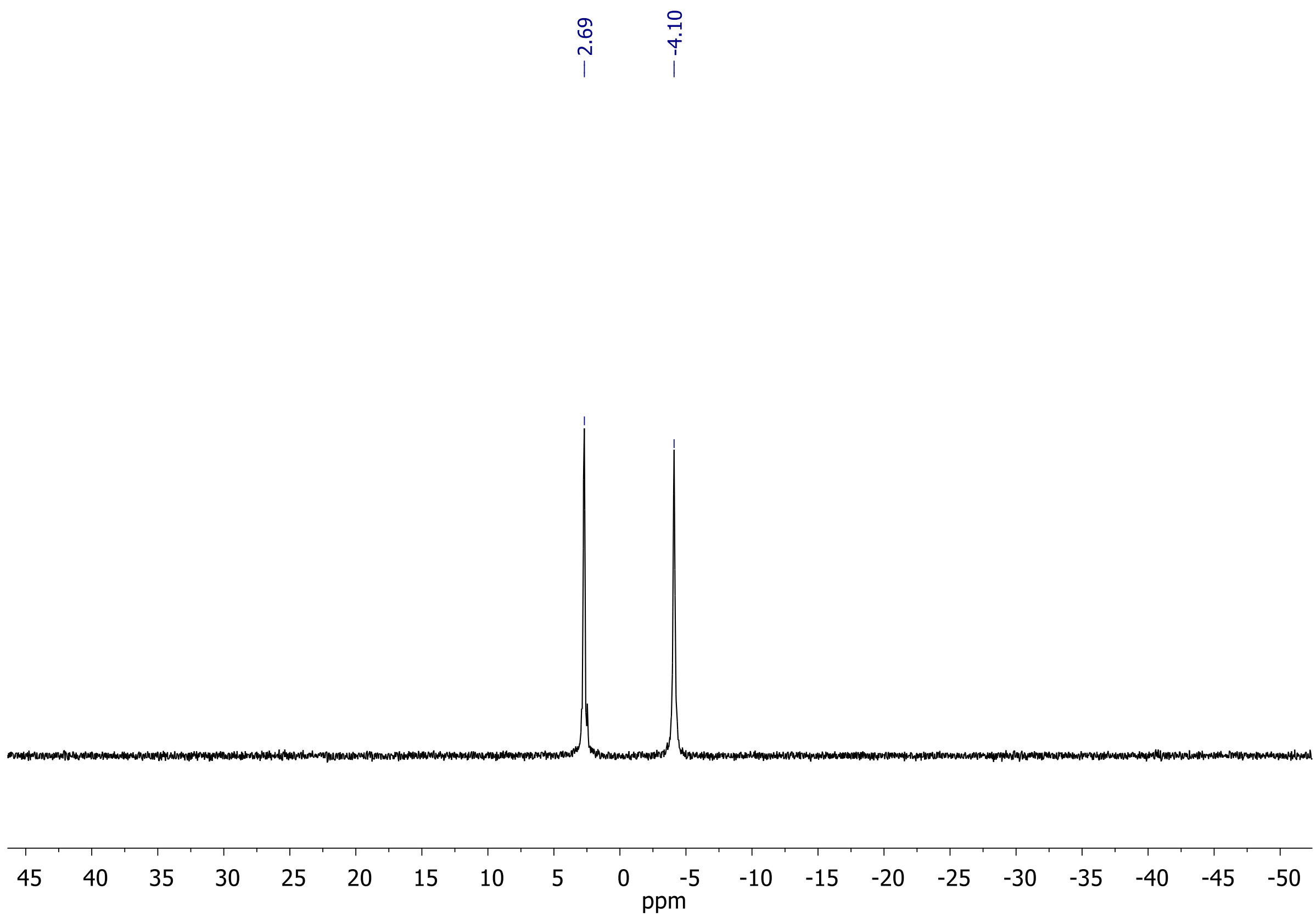

^{31}P NMR spectrum of NTA tweezer **8** (Na salt) (243 MHz, D_2O+DMSO-d_6, RT)

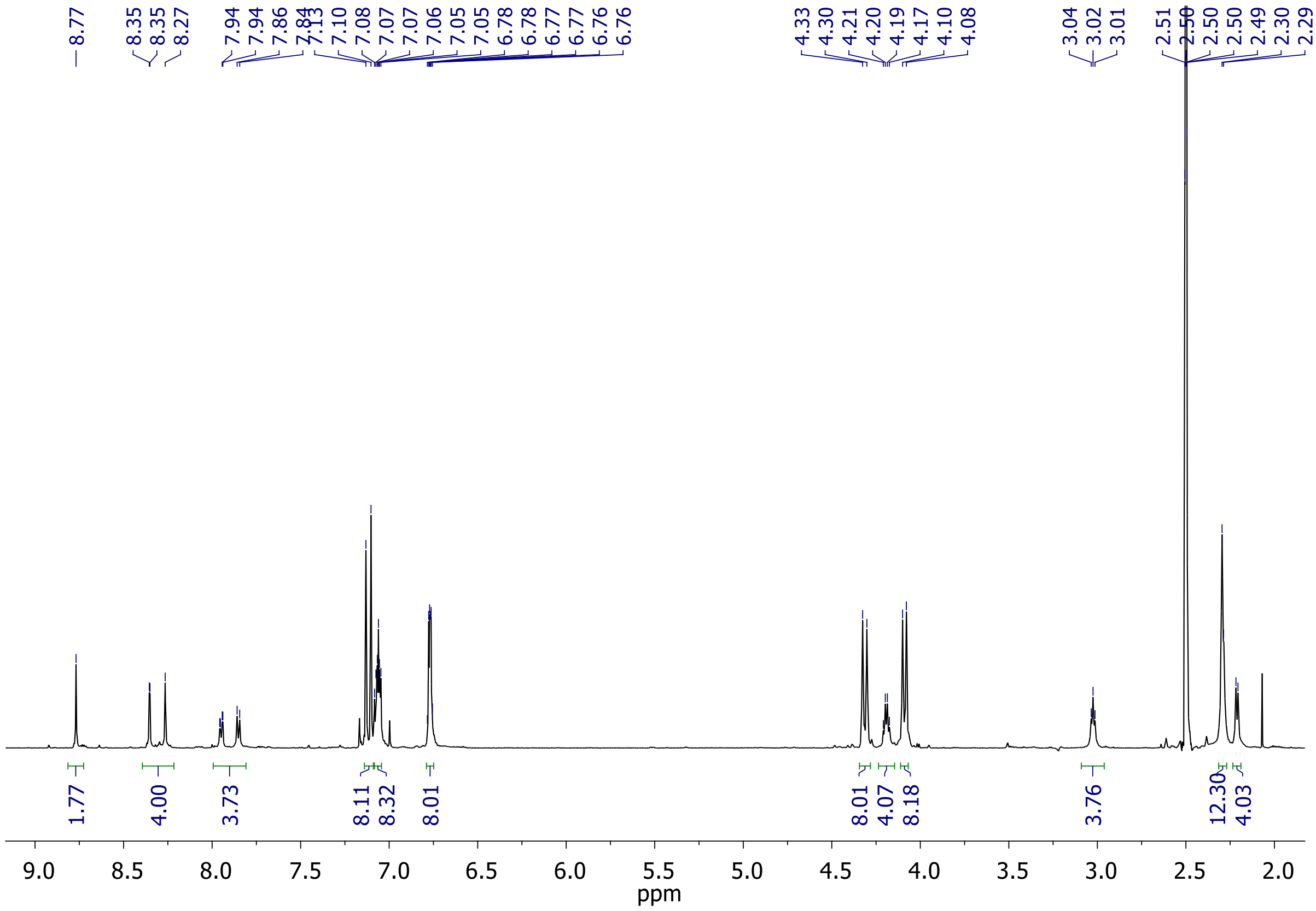

^{1}H NMR spectrum of stilbene disulfonate tweezer **9** (600 MHz, DMSO-d_6, RT)

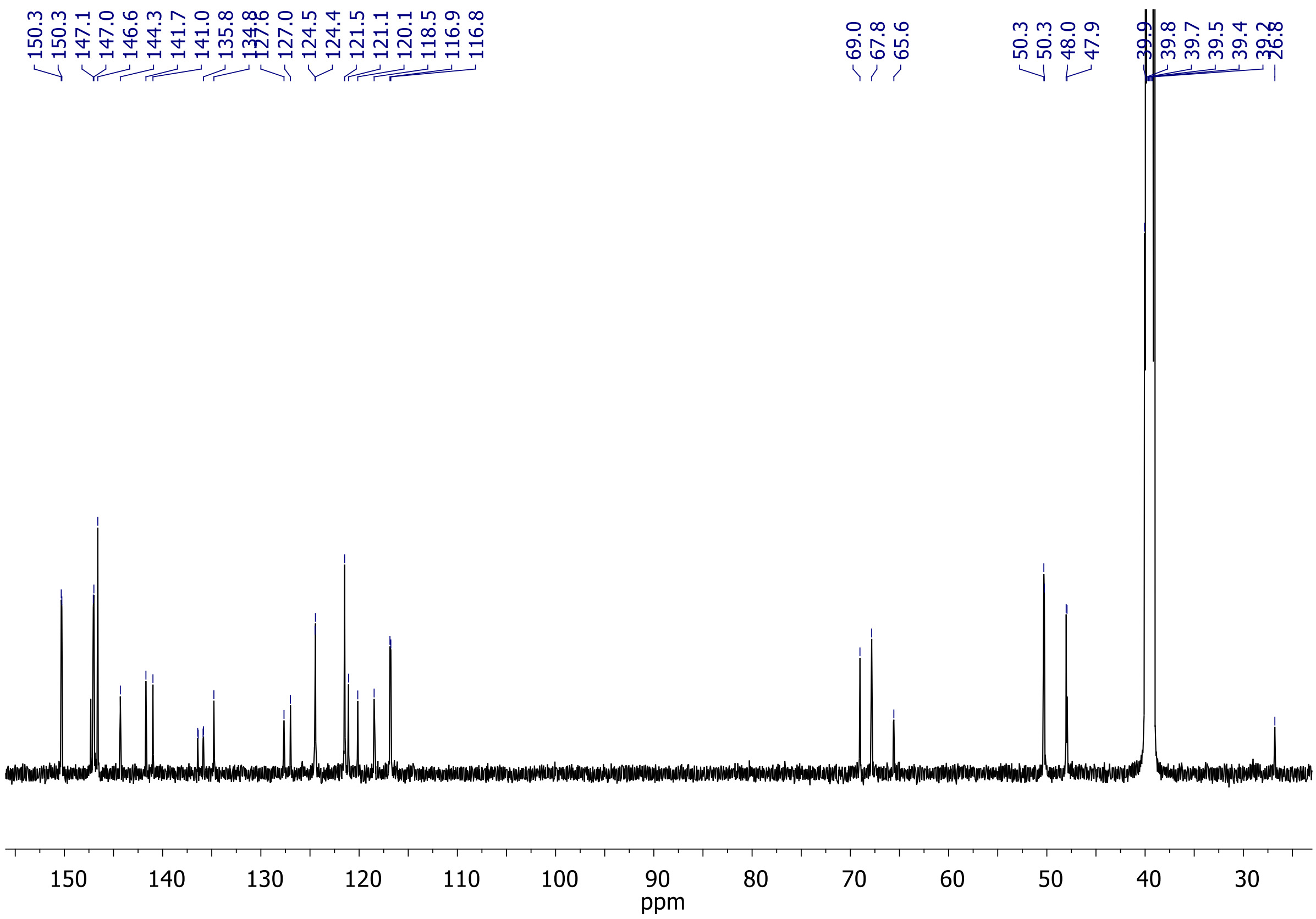

^{13}C NMR spectrum of stilbene disulfonate tweezer **9** (151 MHz, DMSO-d_6, RT)

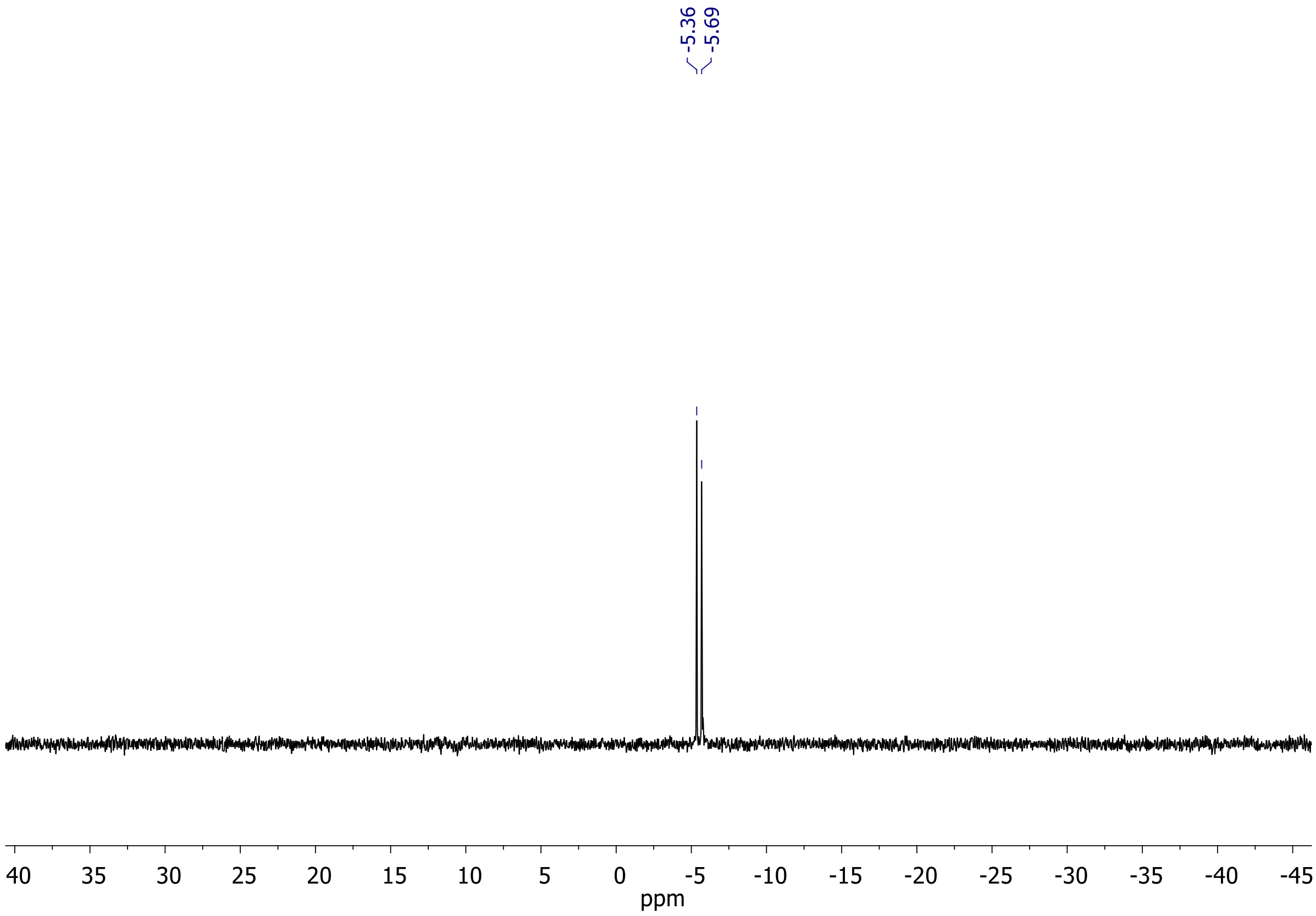

^{31}P NMR spectrum of stilbene disulfonate tweezer **9** (162 MHz, DMSO-d_6, RT)

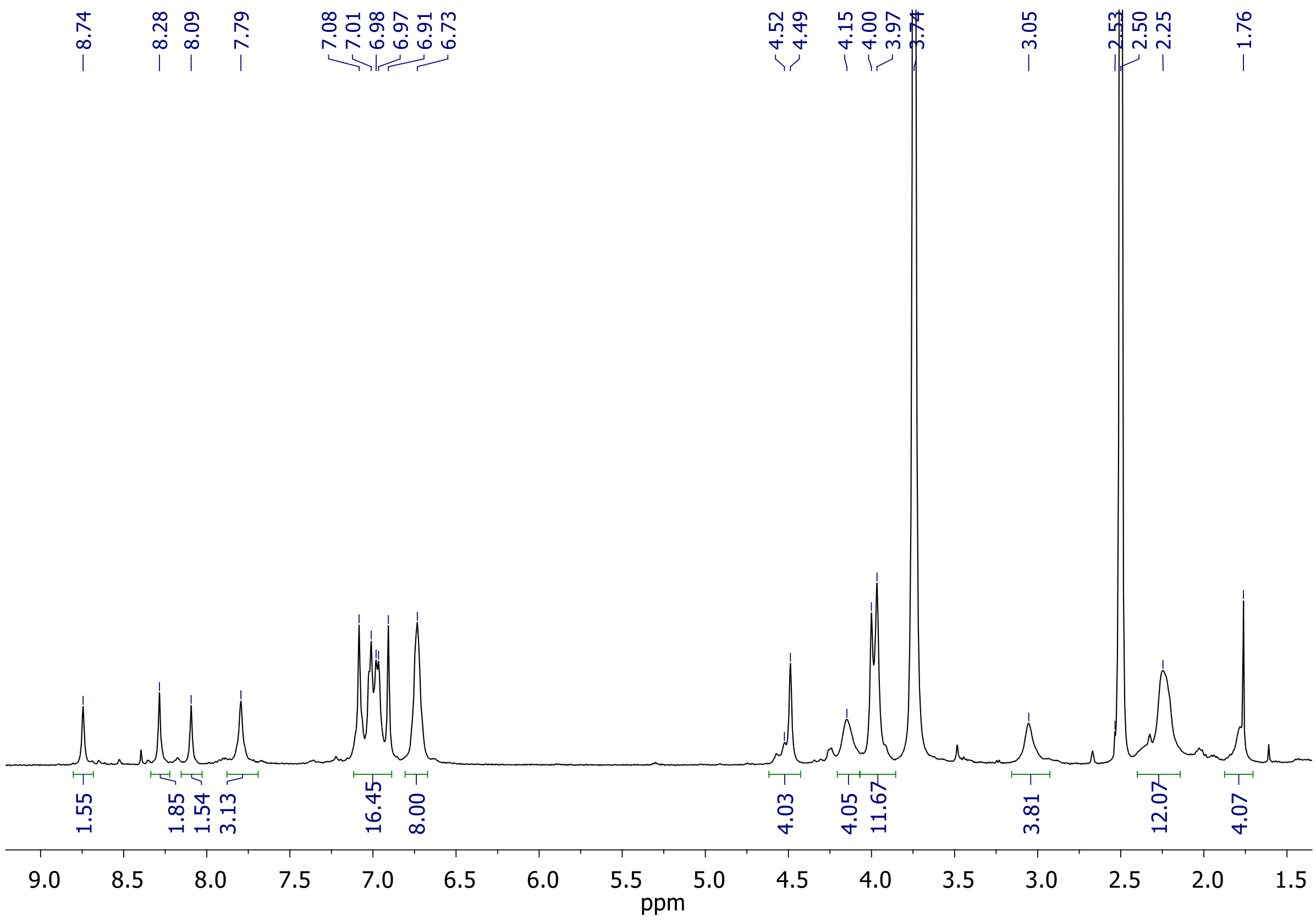

^{1}H NMR spectrum of stilbene disulfonate tweezer **9** (Na salt) (600 MHz, D_2O+DMSO-d_6, RT)

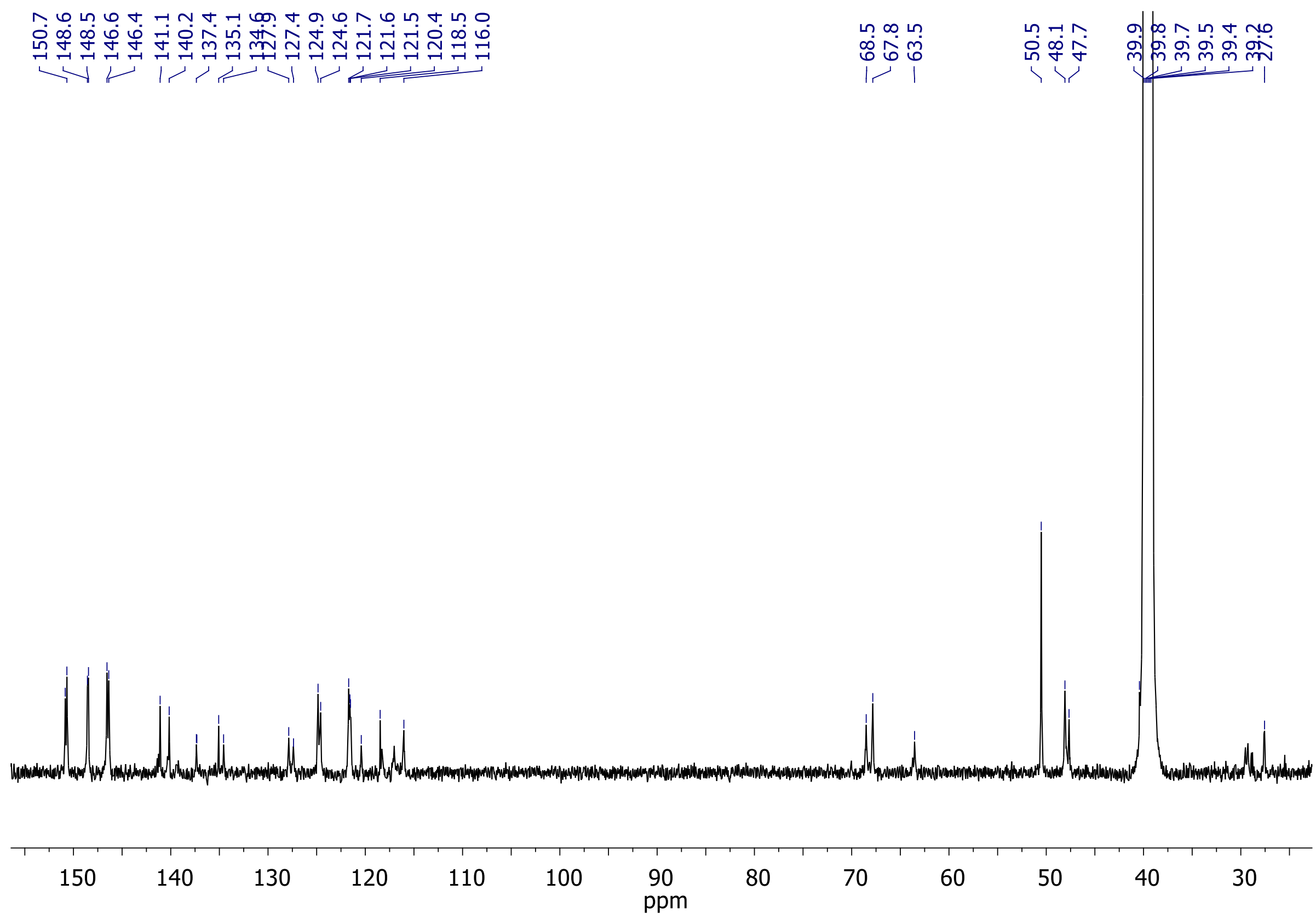

^{13}C NMR spectrum of stilbene disulfonate tweezer **9** (Na salt) (151 MHz, D_2O+DMSO-d_6, RT)

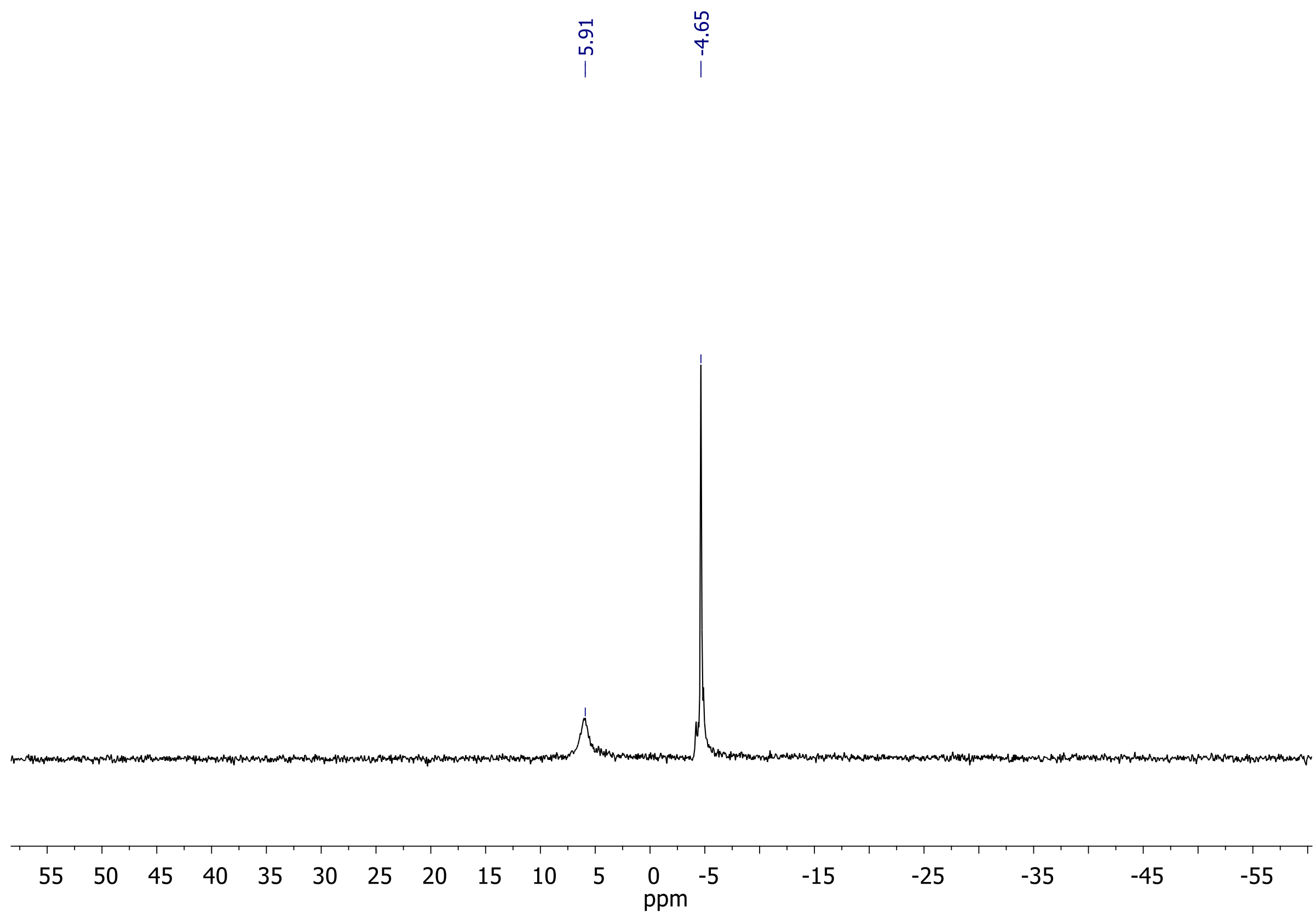

^{31}P NMR spectrum of stilbene disulfonate tweezer **9** (Na salt) (243 MHz, D_2O+DMSO-d_6, RT)

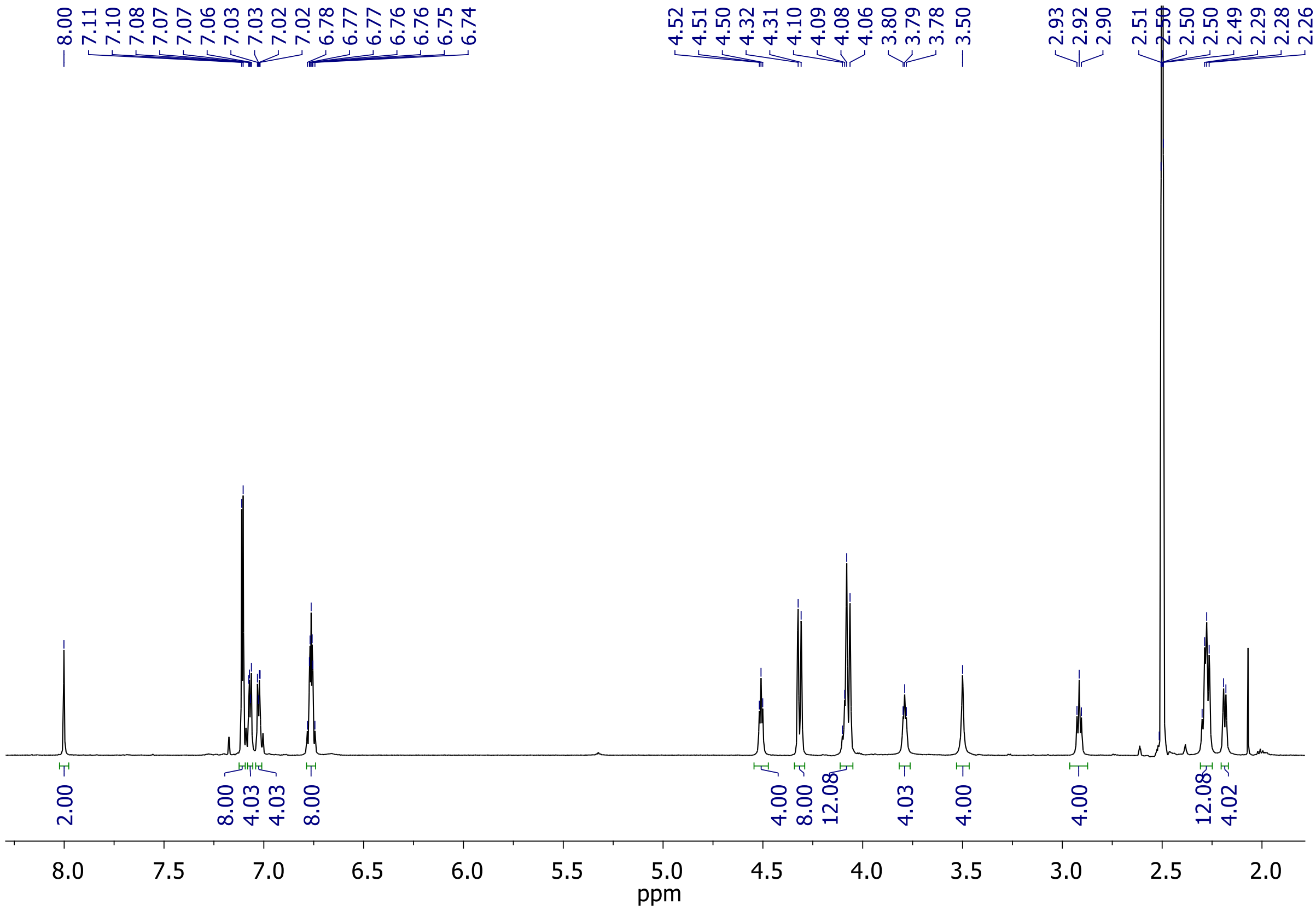

^1H NMR spectrum of PEG tweezer **10** (600 MHz, DMSO-d_6, RT)

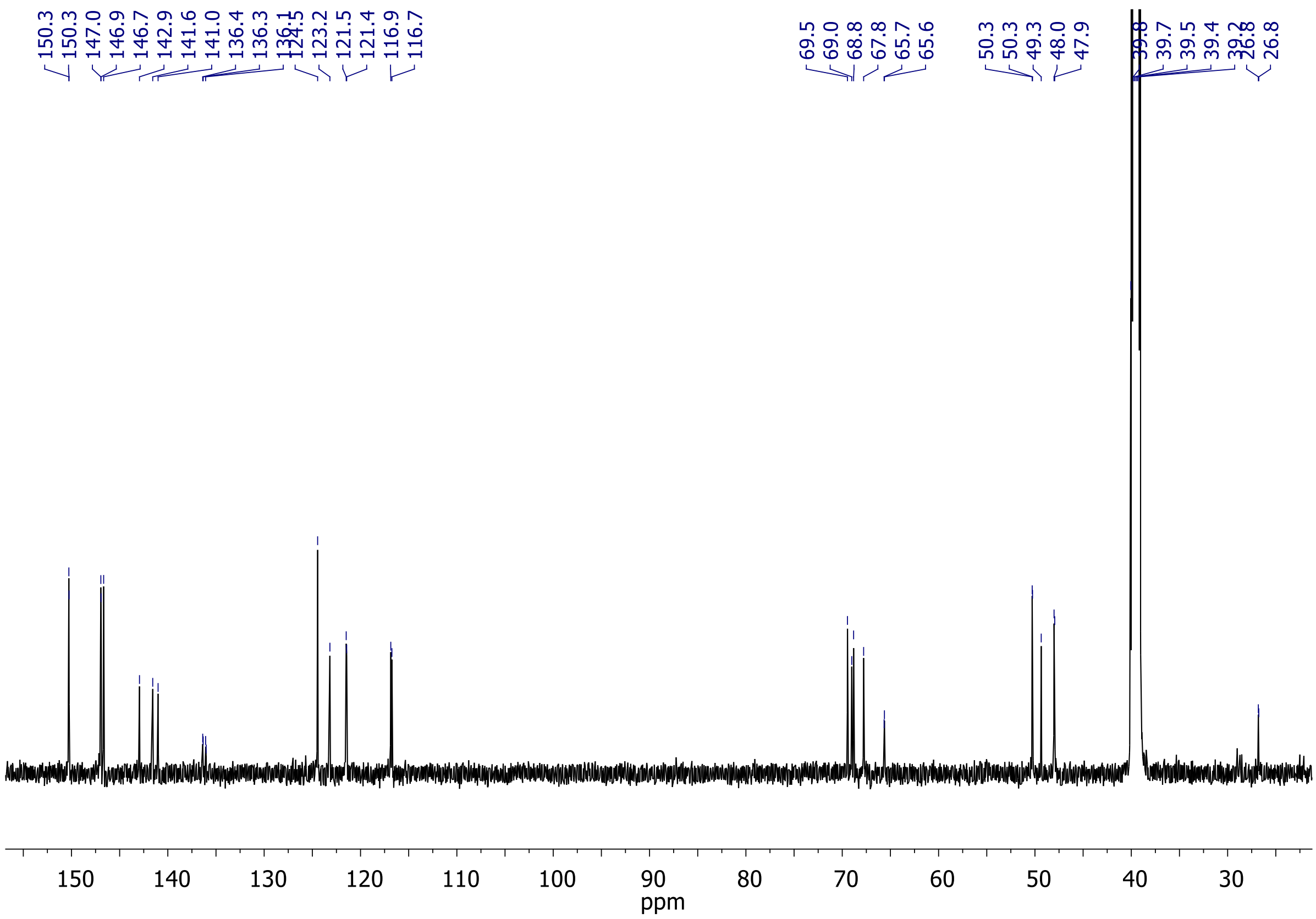

^{13}C NMR spectrum of PEG tweezer **10** (151 MHz, DMSO-d_6, RT)

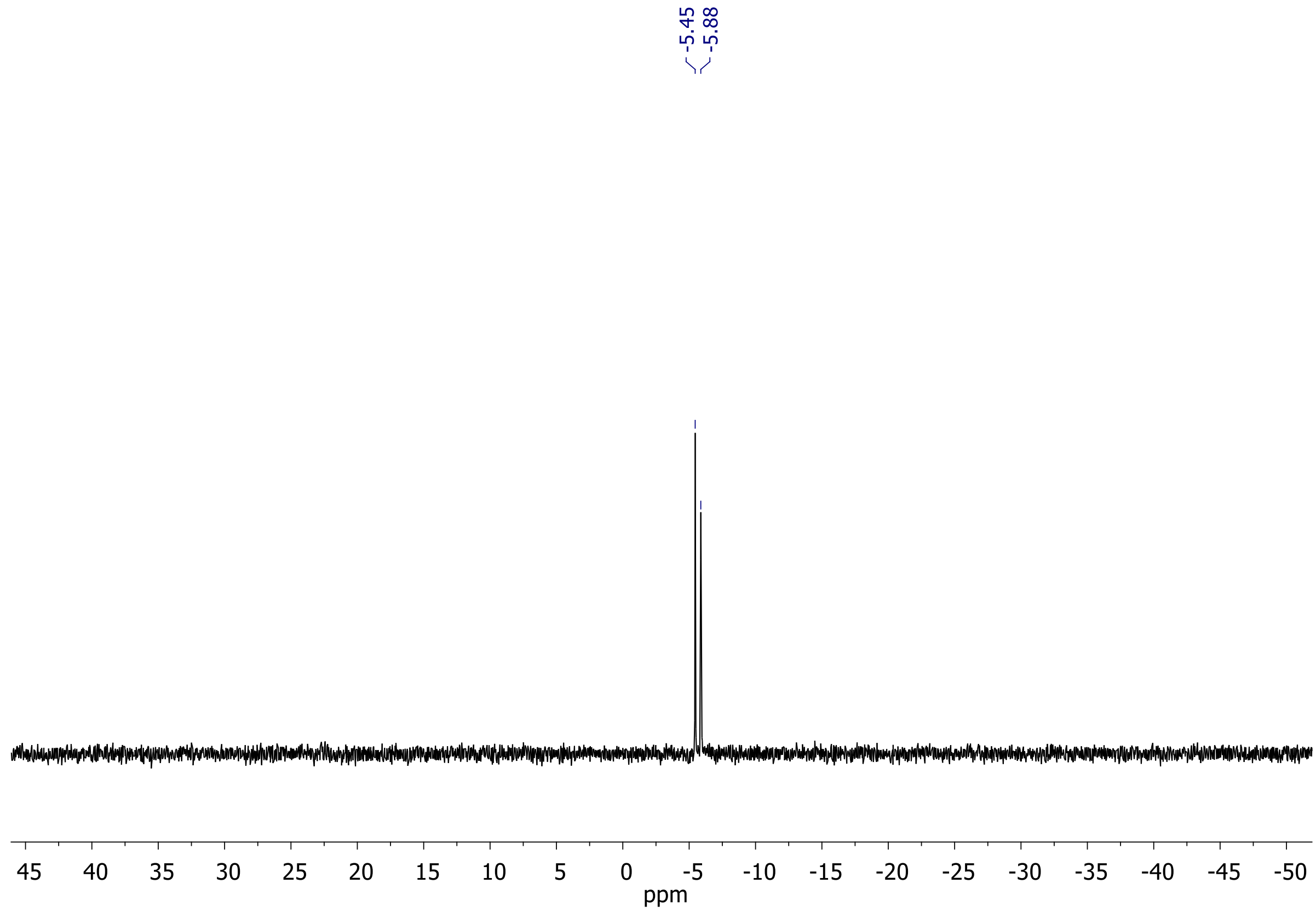

^{31}P NMR spectrum of PEG tweezer **10** (162 MHz, DMSO-d_6, RT)

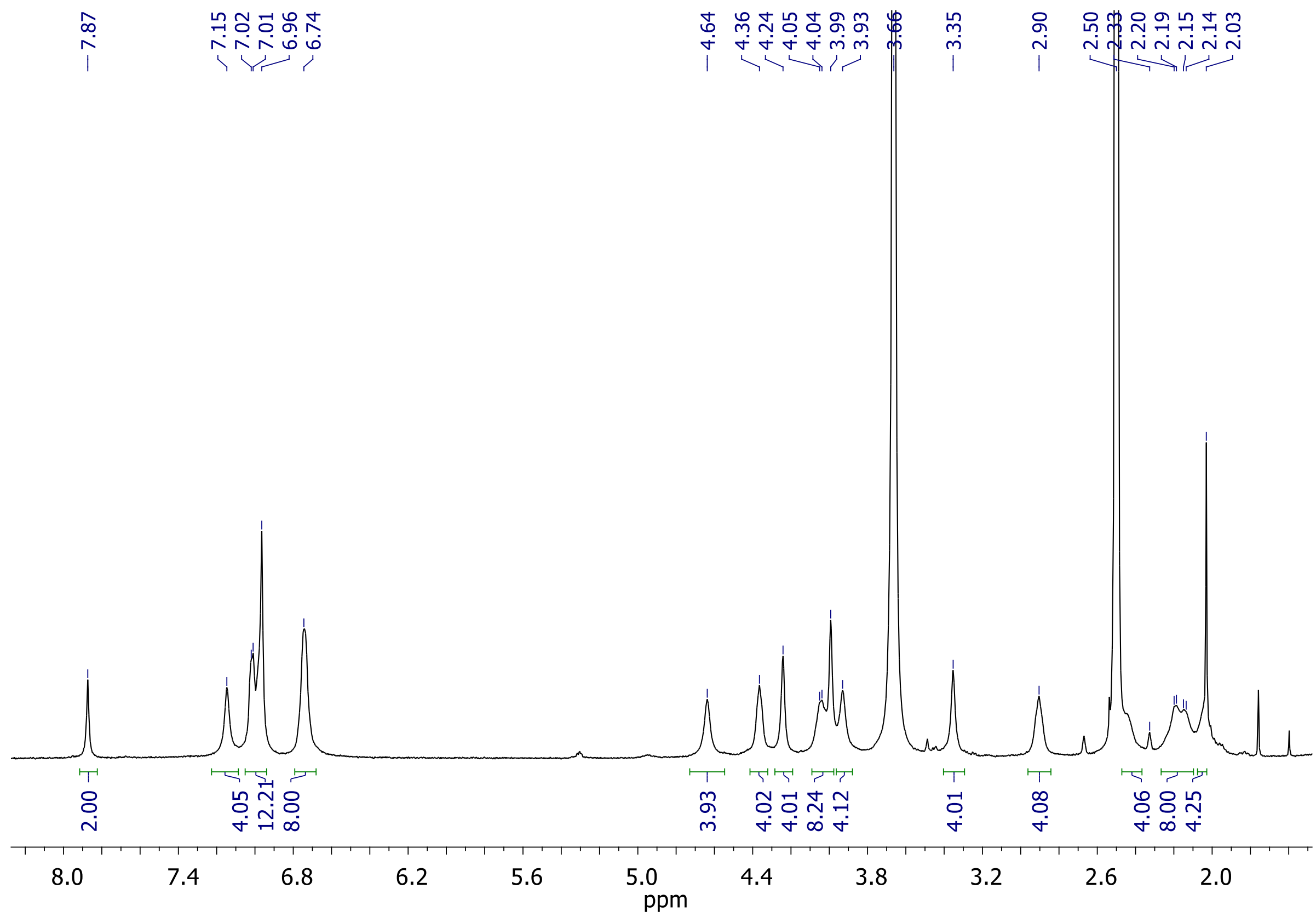

^{1}H NMR spectrum of PEG tweezer **10** (Na salt) (400 MHz, D_2O+DMSO-d_6, RT)

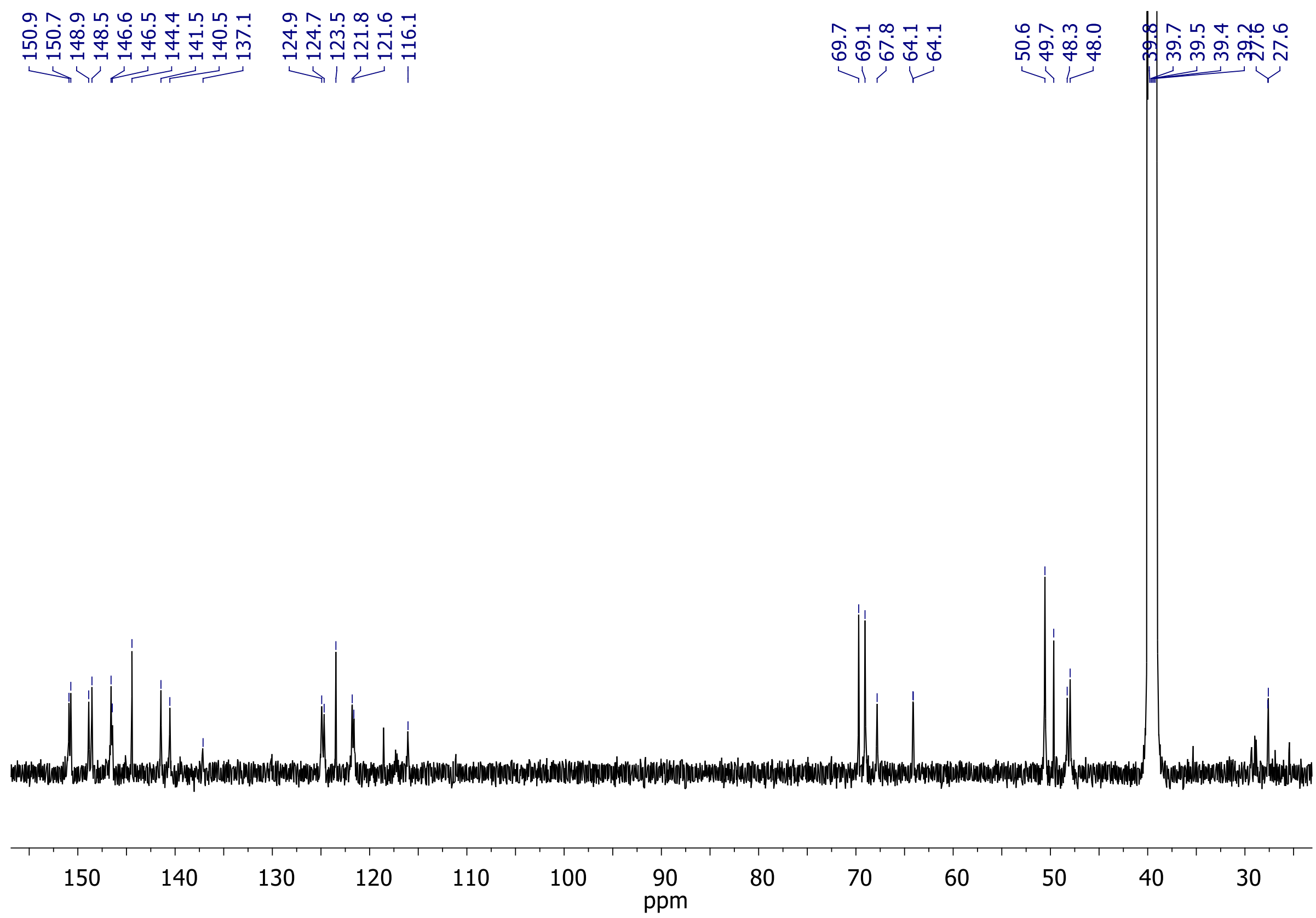

^{13}C NMR spectrum of PEG tweezer **10** (Na salt) (151 MHz, D_2O+DMSO-d_6, RT)

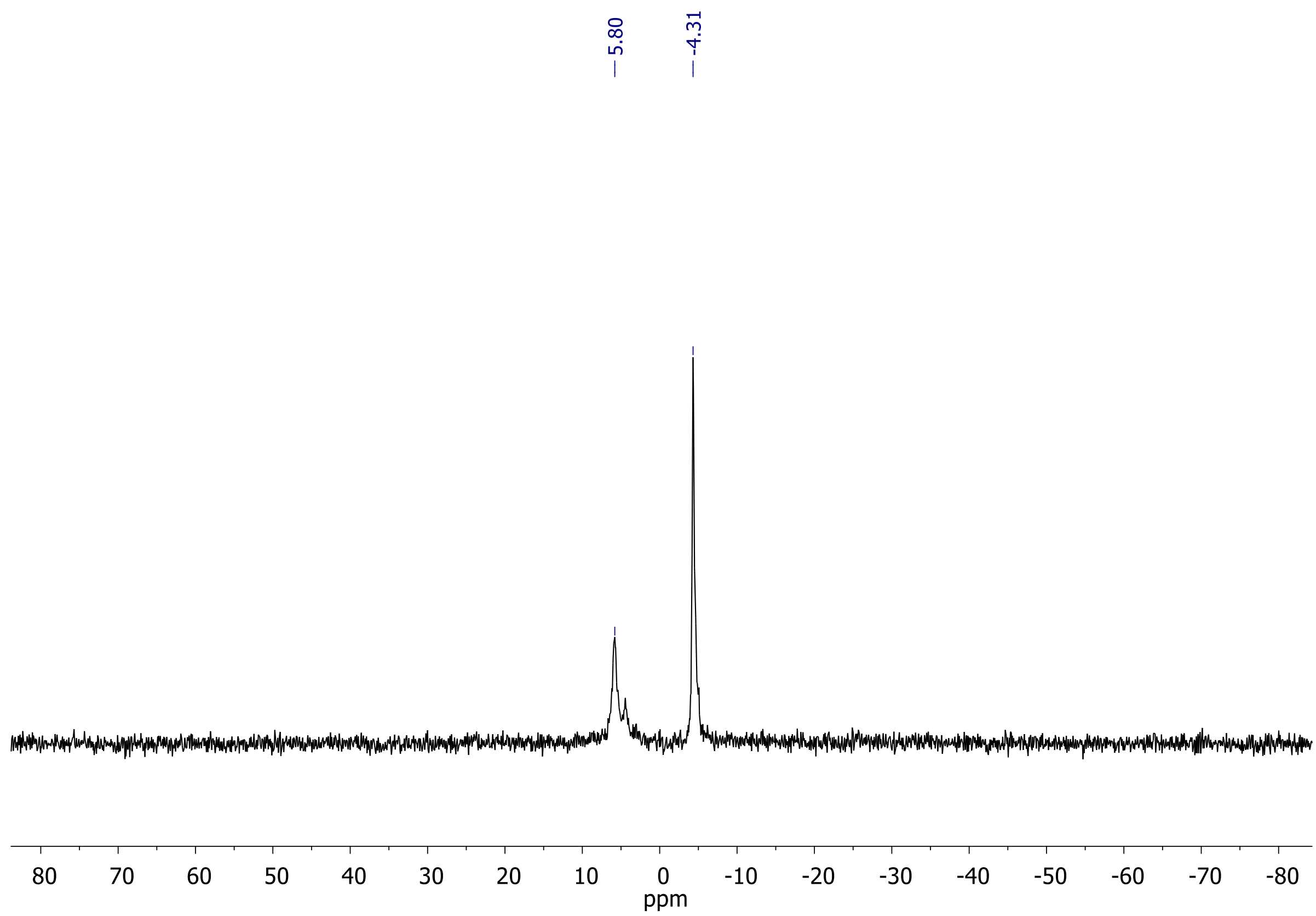

^{31}P NMR spectrum of PEG tweezer **10** (Na salt) (243 MHz, D_2O+DMSO-d_6, RT)

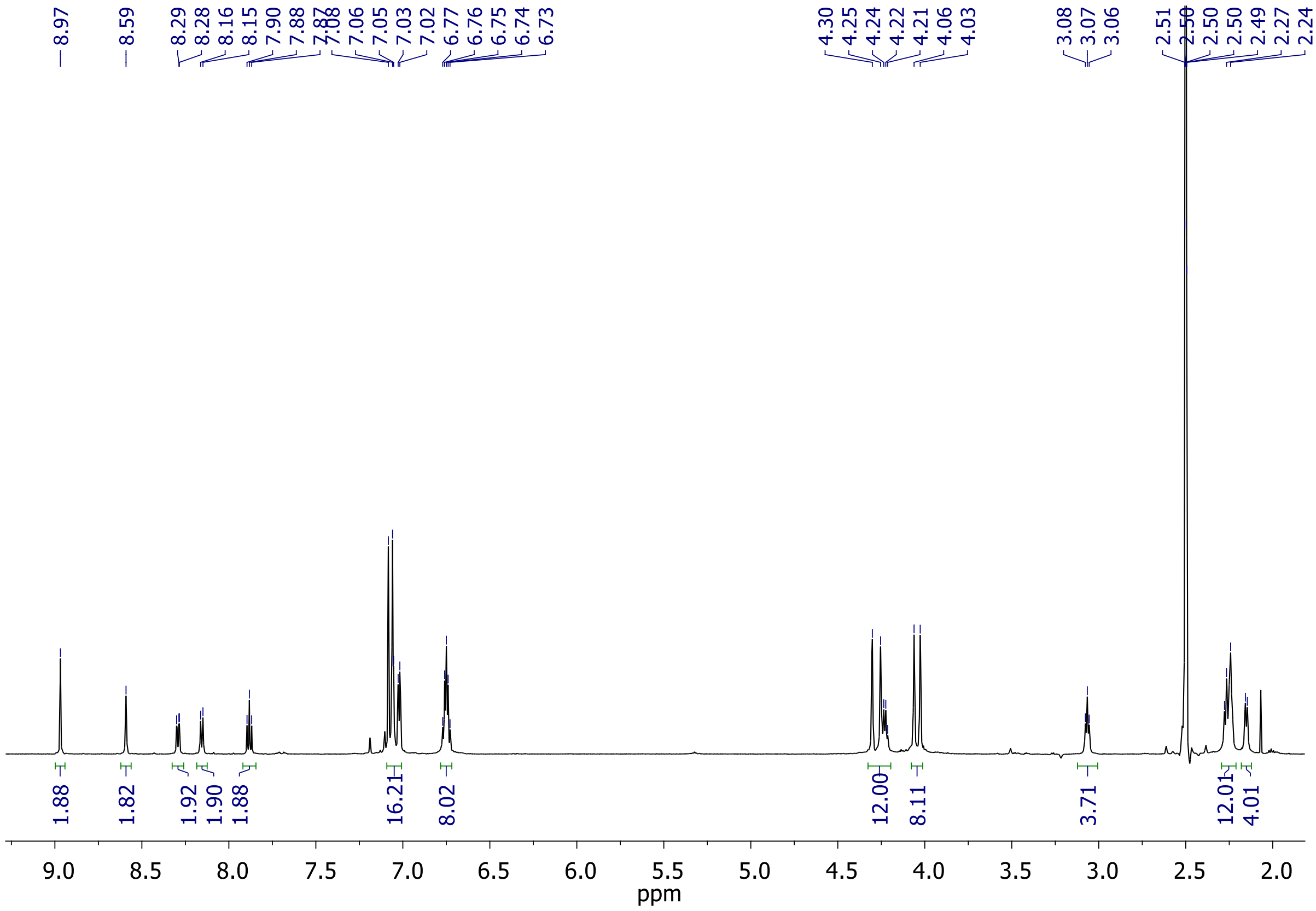

^{1}H NMR spectrum of diphenylsulfone tweezer **11** (600 MHz, DMSO-d_6, RT)

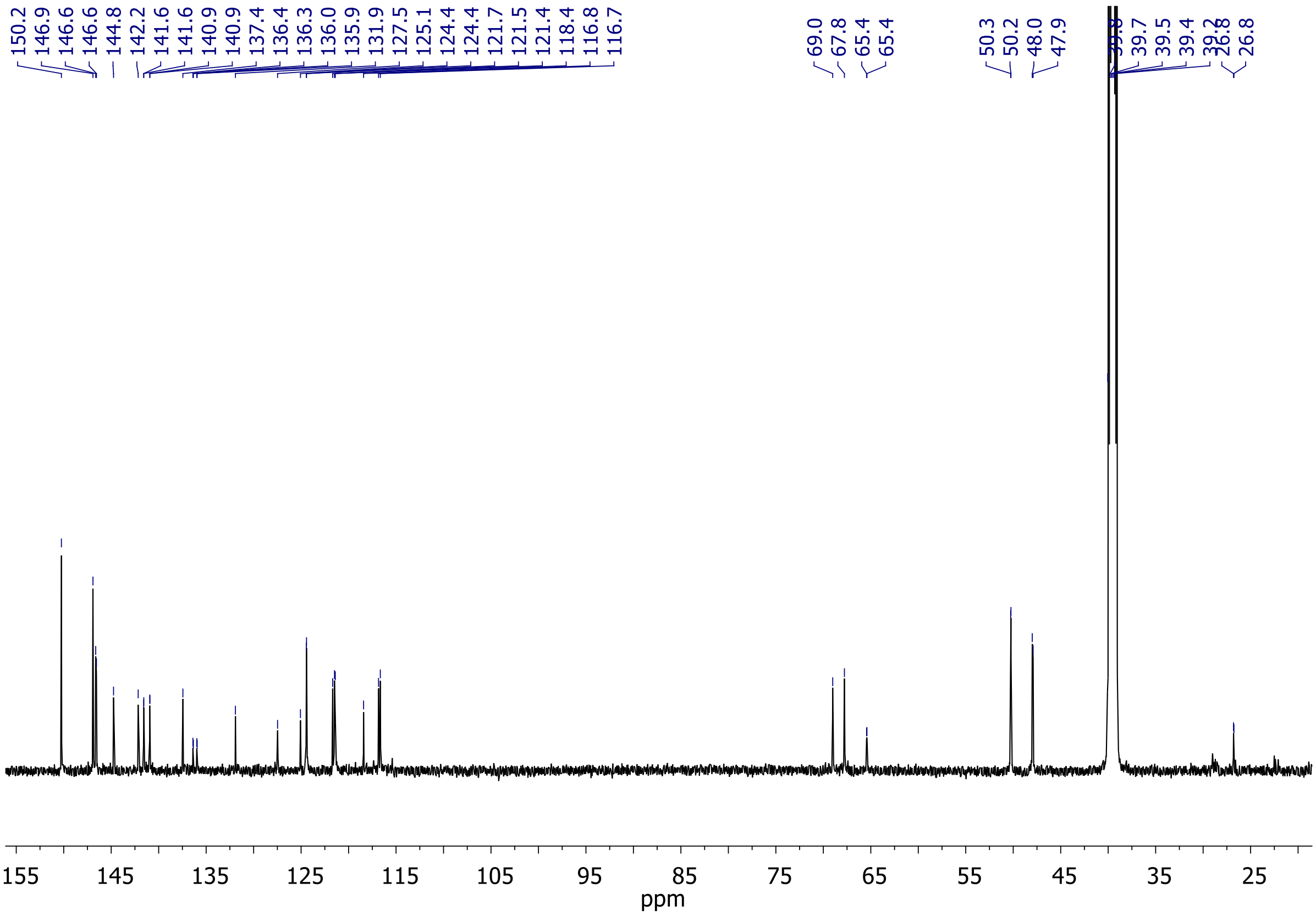

^{13}C NMR spectrum of diphenylsulfone tweezer **11** (151 MHz, DMSO-d_6, RT)

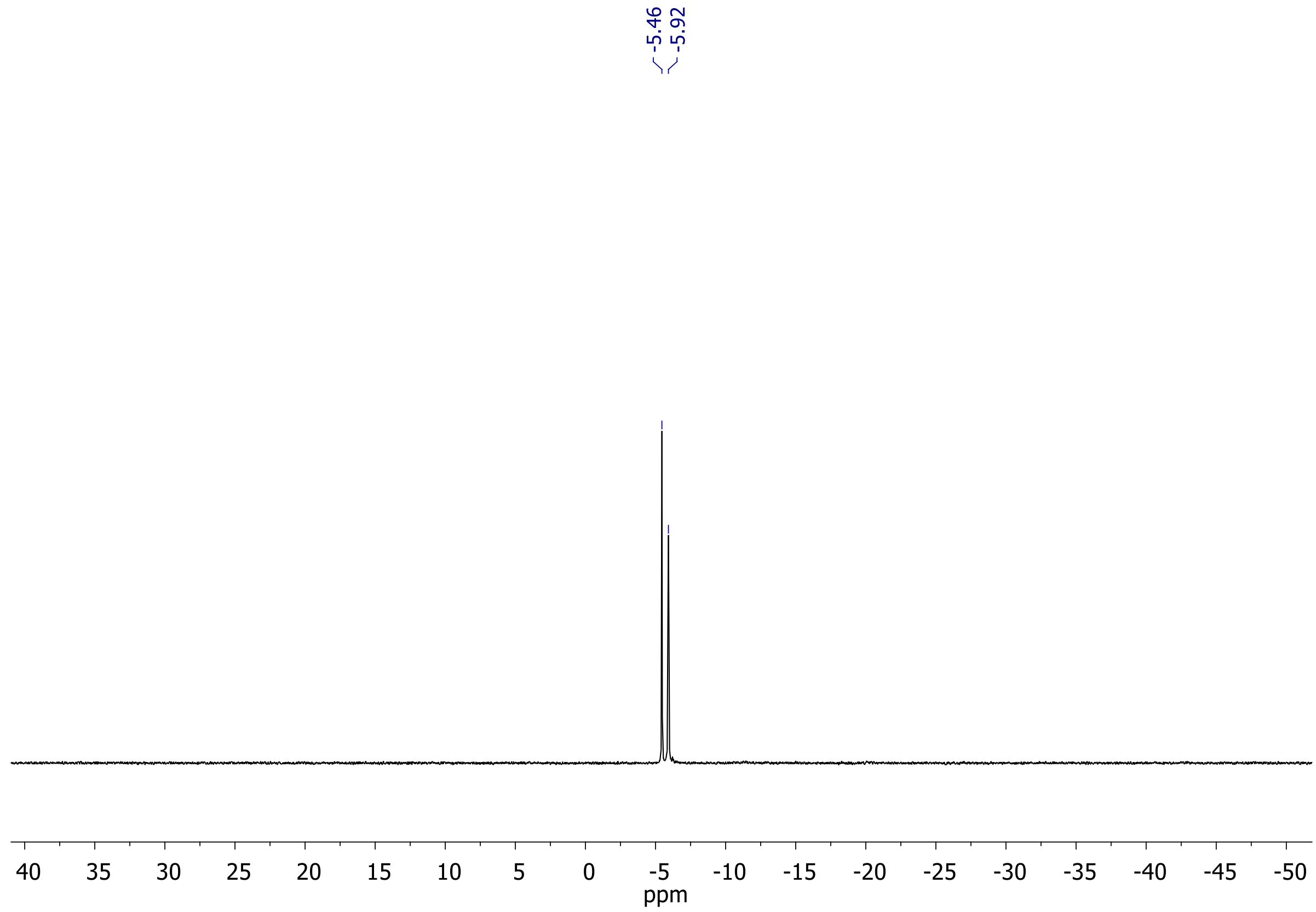

^{31}P NMR spectrum of diphenylsulfone tweezer **11** (243 MHz, DMSO-d_6, RT)

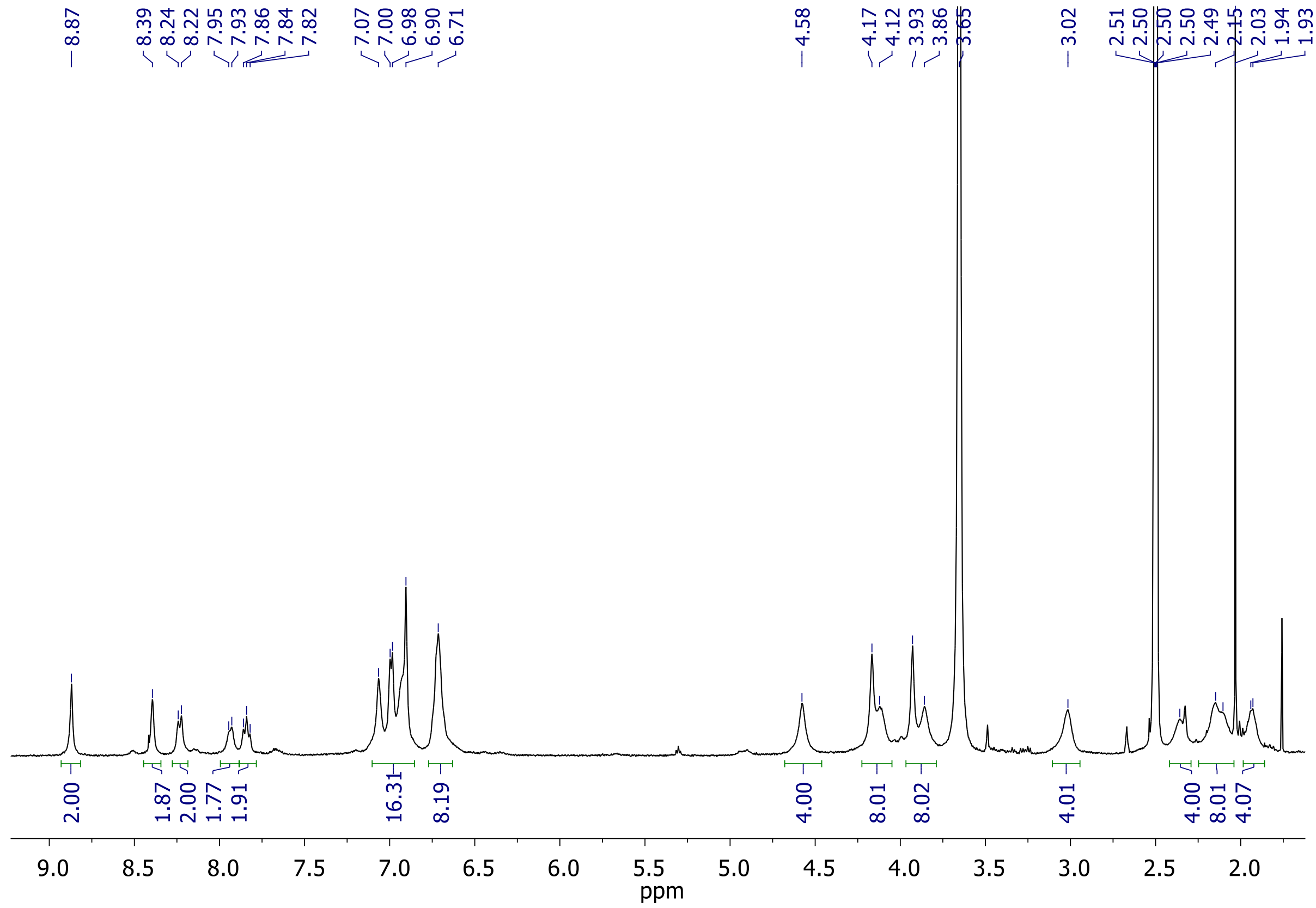

^{1}H NMR spectrum of diphenylsulfone tweezer **11** (Na salt) (400 MHz, D_2O+DMSO-d_6, RT)

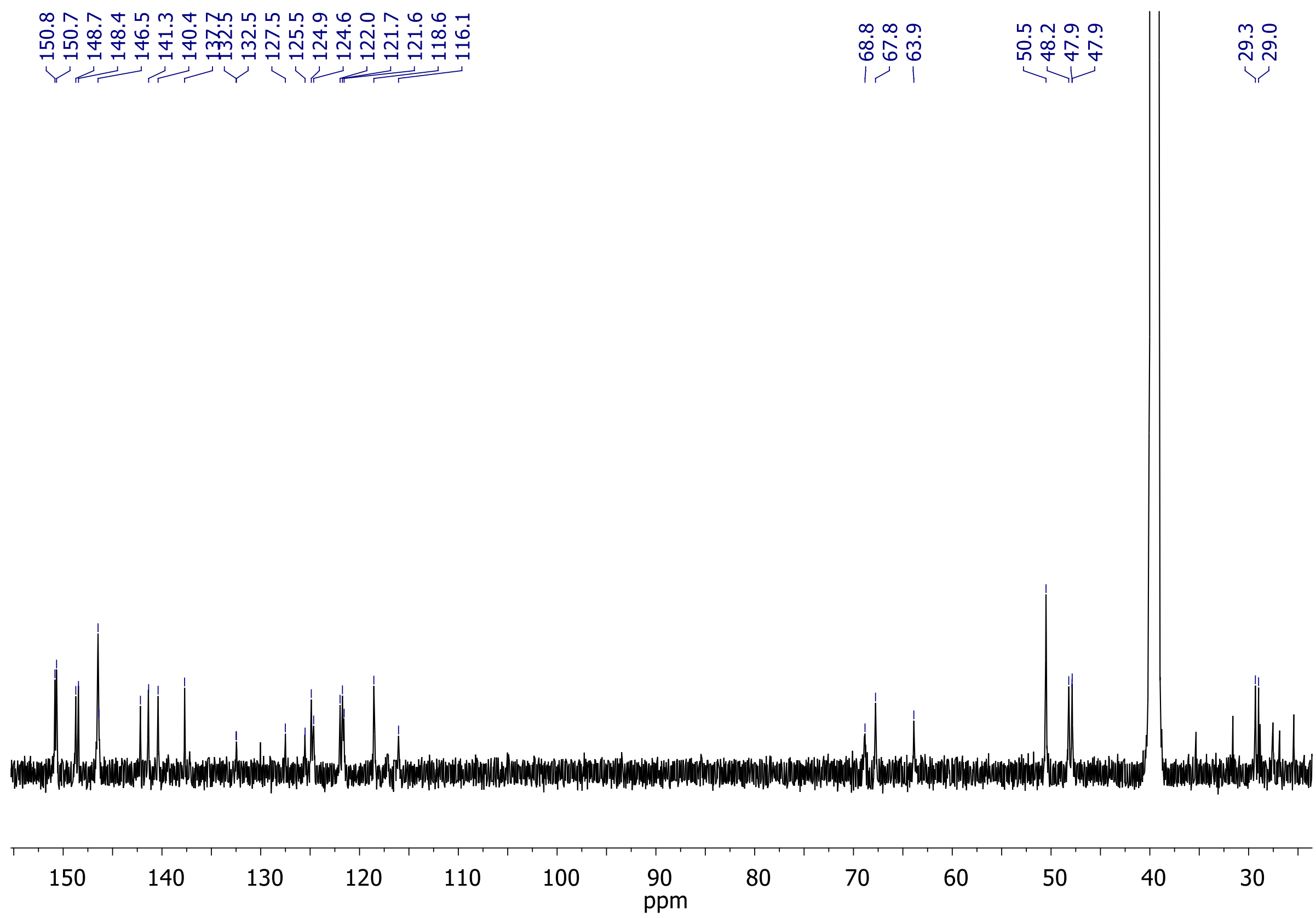

^{13}C NMR spectrum of diphenylsulfone tweezer **11** (Na salt) (151 MHz, D_2O+DMSO-d_6, RT)

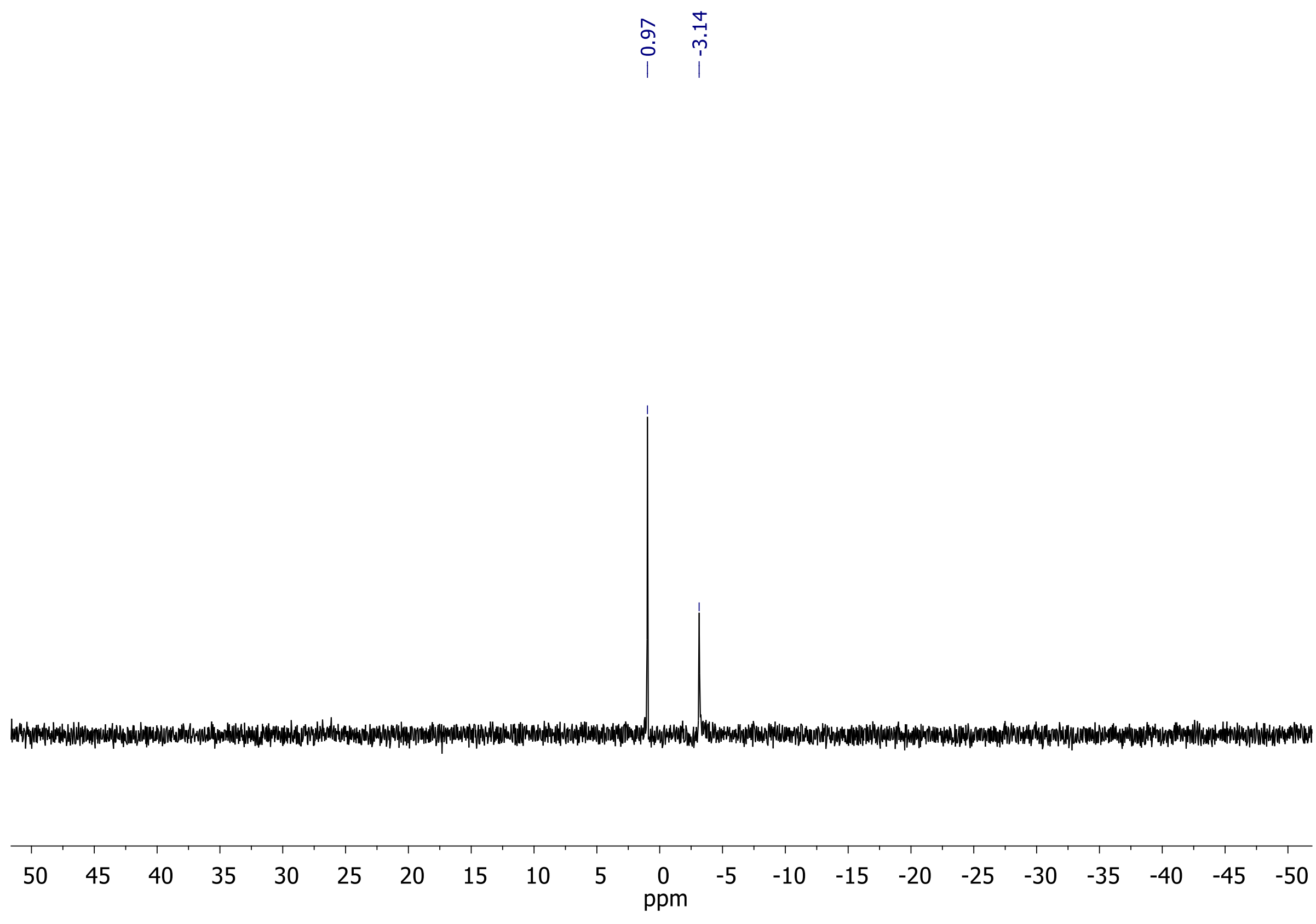

^{31}P NMR spectrum of diphenylsulfone tweezer **11** (Na salt) (162 MHz, D_2O+DMSO-d_6, RT)

References

[1] M. Fokkens, T. Schrader, F.-G. Klärner, *J. Am. Chem. Soc.* **2005**, *127*, 14415-14421.
[2] P. Talbiersky, F. Bastkowski, F.-G. Klärner, T. Schrader, *J. Am. Chem. Soc.* **2008**, *130*, 9824-9828.
[3] C. Heid, A. Sowislok, T. Schaller, F. Niemeyer, F.-G. Klärner, T. Schrader, *Chem. Eur. J.* **2018**, *24,* 11332.
[4] J. Neblik, A. Kirupakaran, C. Beuck, J. Mieres-Perez, F. Niemeyer, M.-H. Le, U. Telgheder, J. F. Schmuck, A. Dudziak, P. Bayer, E. Sanchez-Garcia, S. Westermann, T. Schrader, *J. Am. Chem. Soc.* **2023**, *145*(28), 15251-15264.
